# *CircVAPA* promotes small cell lung cancer progression by modulating the *miR-377-3p* and *miR-494-3p*/IGF1R/AKT axis

**DOI:** 10.1186/s12943-022-01595-9

**Published:** 2022-06-06

**Authors:** Jinghan Hua, Xiaolin Wang, Liying Ma, Jingxin Li, Guozhen Cao, Shaobo Zhang, Wenchu Lin

**Affiliations:** 1 High Magnetic Field Laboratory, Hefei Institutes of Physical Science, Chinese Academy of Sciences, Hefei, 230031 Anhui, China; 2 University of Science and Technology of China, Hefei, 230026 Anhui, China; 3 Key Laboratory of High Magnetic Field and Ion Beam Physical Biology, Hefei Institutes of Physical Science, Chinese Academy of Sciences, Hefei, 230031 Anhui, China; 4 High Magnetic Field Laboratory of Anhui Province, Hefei, 230031 Anhui, China; 5 Department of Pathology and Pathophysiology, School of Basic Medicine, Anhui Medical University, Hefei, 230031 China

**Keywords:** *CircVAPA*, SCLC, Progression, *miR-377-3p*, *miR-494-3p*, IGF1R, AKT, BMS-536924

## Abstract

**Background:**

Multiple lines of evidence have demonstrated that circular RNAs (circRNAs) play oncogenic or tumor-suppressive roles in various human cancers. Nevertheless, the biological functions of circRNAs in small cell lung cancer (SCLC) are still elusive.

**Methods:**

*CircVAPA* (annotated as hsa_circ_0006990) was identified by mining the circRNA profiling dataset of six paired SCLC tissues and the RNA-seq data of serum samples from 36 SCLC patients and 118 healthy controls. The *circVAPA* expression level was evaluated using quantitative real-time PCR in SCLC cells and tissues. Cell viability, colony formation, cell cycle and apoptosis analysis assays and in vivo tumorigenesis were used to reveal the biological roles of *circVAPA*. The underlying mechanism of *circVAPA* was investigated by Western blot, RNA pulldown, RNA immunoprecipitation, dual-luciferase reporter assay and rescue experiments.

**Results:**

We revealed that *circVAPA*, derived from exons 2-4 of the vesicle-associated membrane protein-associated protein A (VAPA) gene, exhibited higher expression levels in SCLC cell lines, clinical tissues, and serum from SCLC patients than the controls, and facilitated SCLC progression in vitro and in vivo*.* Mechanistically, *circVAPA* activated the phosphoinositide 3-kinase (PI3K)/protein kinase B (AKT) signaling pathway by modulating the *miR-377-3p* and *miR-494-3p*/insulin-like growth factor 1 receptor (IGF1R) axis to accelerate SCLC progression. Furthermore, *circVAPA* depletion markedly enhanced the inhibitory effects of BMS-536924, an IGF1R kinase inhibitor in cellular and xenograft mouse models.

**Conclusions:**

*CircVAPA* promotes SCLC progression via the *miR-377-3p* and *miR-494-3p*/IGF1R/AKT axis. We hope to develop clinical protocols of combinations of *circVAPA* inhibition and BMS-536924 addition for treating SCLC with *circVAPA* upregulation.

**Supplementary Information:**

The online version contains supplementary material available at 10.1186/s12943-022-01595-9.

## Background

Small cell lung cancer (SCLC) is a highly aggressive and lethal subtype of lung cancer, characterized by bi-allelic inactivation of tumor suppressors RB1 and p53 [1–3]. Despite the significant advances achieved in chemotherapy, SCLC patients almost recur within one year, resulting in a quite poor prognosis and its 5-year survival rate less than 7% [4–7]. Therefore, it is urgent to elucidate the underlying mechanisms of SCLC progression and identify the potential biomarkers and therapeutic targets in SCLC.

Circular RNAs (circRNAs) are back-spliced from precursor mRNA (pre-mRNA) formed with exons or introns, in which a downstream splice site is joined with an upstream splice site [8–12]. Reverse complementary sequences such as *Alu* elements in the flanking region of circularized exons and RNA binding proteins (RBPs) are the major factors contributing to circRNA biogenesis [13–17]. Due to its covalent closed loop structure and lack of exposed terminal ends, circRNAs are resistant to the degradation by exonucleases and highly stable in blood and other body fluids [10, 18]. Accumulating evidence has demonstrated that the dysregulated circRNAs are associated with specific hallmarks of cancer, including sustaining proliferative signaling, evading growth suppressors, activating invasion, and metastasis [10, 18–25]. For instance, *circPTPRA* acts as a competitive endogenous RNA (ceRNA) against *miR-96-5p* to erase the repressive role of RASSF8, consequently suppressing epithelial-mesenchymal transition and metastasis of non-small cell lung carcinoma (NSCLC) [22]. *CircURI1* behaves as a decoy of heterogeneous nuclear ribonucleoprotein M (hnRNPM) to modulate alternative splicing of *VEGFA*, thereby inhibiting gastric cancer metastasis [20]. Several circRNAs with translational effects are also involved in tumorigenesis [10, 18, 25]. For example, circular RNA E-cadherin undergoes translation to encode a unique E-cadherin protein variant (C-E-Cad), promoting glioblastoma tumorigenicity by activating the EGFR–STAT3 signaling [25]. However, the biological functions and underlying mechanisms of circRNAs in SCLC occurrence and development remain unclear.

The insulin-like growth factor 1 receptor (IGF1R) exerts essential functions in transmitting signals through the IGF1/IGF1R signaling axis-mediated pathway, which belongs to the tyrosine kinase receptor family [26–28]. Aberrant regulation of the IGF1R signaling pathway has been recognized as a well-established therapeutic target in a variety of human malignancies, including SCLC [29, 30]. BMS-536924, an ATP-competitive IGF1R inhibitor, can suppress the IGF1R-induced phosphorylation of phosphoinositide 3-kinase (PI3K)/ protein kinase B (AKT), consequently repressing cancer cell proliferation and differentiation [31–34].

To clarify the functions of circRNAs in SCLC tumorigenesis, bioinformatics analysis of differentially expressed circRNAs in SCLC tissues and serum from SCLC patients identified *circVAPA* as an up-regulated circular RNA. Loss- and gain- of function experiments revealed that *circVAPA* accelerated cell cycle progression, cell proliferation, and colony formation in SCLC. Mechanically, we found that *circVAPA* served as a ceRNA against *miR-377-3p* and *miR-494-3p* to weaken their inhibitory effects on *IGF1R* mRNA expression, thus promoting SCLC progression by activating the PI3K/AKT pathway.

## Materials and methods

### Clinical SCLC samples

All SCLC clinical samples and the corresponding non-tumor tissues were collected from Anhui Provincial Hospital. Written informed consent was obtained from all patients for this study. All fresh samples were immediately frozen in liquid nitrogen after removing from the operation and stored in liquid nitrogen for further investigation.

### Cell lines and reagents

All human SCLC cell lines (H69, DMS79, H82, DMS273, H446, and H526) and non-small cell lung cancer (NSCLC) cell lines (HCC827 and PC9) were maintained under standard culture conditions with RPMI-1640 media supplemented with 10% fetal bovine serum (FBS) and 1% penicillin/streptomycin (PS) at 37 °C in a humidified atmosphere with 5% CO_2_. BMS-536924 was purchased from Glpbio (Cat NO. GC17773) and kept as a stock solution of 100 nM in dimethyl sulfoxide (DMSO).

### RNA preparation and quantitative real-time PCR

The nuclear and cytoplasmic fractions were isolated as described previously [20]. Total RNA from whole-cell lysates or the cytoplasmic and nuclear fractions were extracted using TRIzol (Thermo Scientific) according to the manufacturer’s instructions. Complementary DNA (cDNA) was synthesized using the Transcriptor First Strand cDNA Synthesis Kit (Roche). *18S rRNA* was used for miRNAs template normalization, and *β-actin* was used as an internal standard for *circVAPA* and mRNAs. Real-time quantitative PCR (RT-qPCR) was performed using ChamQ SYBR qPCR Master Mix (Vazyme) on a Roche Light Cycler 96 Real-Time PCR System. Oligonucleotide sequences for primers using in RT-qPCR were listed in Table S5.

### Fluorescence in situ hybridization (FISH)

The Cy3-labeled probe against the back-spliced junction in *circVAPA* was synthesized by RiboBio (Guangzhou, China). FISH was performed using a FISH kit (RiboBio) following the manufacturer's guidelines. The images were visualized using an Olympus SpinSR10 confocal microscope.

### Quantification of RNA copy number per cell

Quantification of RNA copy number per cell was carried out as previously described with some modifications [35]. The DNA fragments corresponding to *circVAPA*, *IGF1R*, *miR-377-3p* and *miR-494-3p* were amplified with cDNA and the amount of the purified product were used to plot standard curves through real-time PCR. R squares for Spearman’s correlation coefficient, and P values were calculated by Spearman’s correlation test. Total RNA was extracted from 10^5^ DMS273 and H82 cells, respectively, and cDNA was subsequently synthesized. The copy numbers per cell in each cell line were calculated based on the specific number of cells and the Ct value using the standard curve.

### Plasmid construction and cell transfection

The short hairpin RNA (shRNA) oligonucleotides targeting the junction site of *circVAPA* were inserted into the pLKO.1 vector (Sigma). Then the constructs were packaged into lentivirus, which were used to infect SCLC cells. The cells were subsequently selected using puromycin resistance for one week. The surviving cells were regarded as stable *circVAPA* knockdown cells. For *circVAPA* overexpression, the second, third and fourth exons of VAPA gene and the endogenous flanking sequence including the complementary *Alu* element pairs were inserted into the backbone vector of pcDNA3. Transfection was carried out using Effectene Transfection Reagent (QIAGEN) according to the manufacturer’s protocol. The *circVAPA* level was assessed using RT-qPCR. Oligonucleotide sequences for primers used in plasmid construction, short interfering RNAs (siRNAs), and shRNA were listed in Table S5. The pGL-3 basic luciferase reporter vector and pRL-TK renilla luciferase vector were purchased from Promega.

### Cell viability assay

SCLC cells (3 × 10^3^/well) were seeded into 96-well plates. After transfection or drug treatment for 48 h, these cells were analyzed using the CellTiter-Glo luminescent assay according to the manufacturer’s instructions [36]. The multi-label plate reader (Envision PerkinElmer) was used to detect the luminescence signals.

### Cell cycle and apoptosis analysis

SCLC cells were cultured in 6-well plates at a concentration of 2 × 10^5^ cells per well. For cell cycle analysis, 48 h after transfection, SCLC cells were fixed in 80% ethanol at -20 °C overnight, followed by the staining with PI/RNase staining buffer (BD Biosciences). Cells were measured for cell cycle distribution by flow cytometry, and the cell-cycle profiles were further analyzed using ModFit software (Verity Software House). For cell apoptosis assay, apoptotic cells were determined as previously described [36]. Cells were stained with FITC Annexin V and PI using the FITC Annexin V Apoptosis Detection Kit (BD Pharmingen) according to the manufacturer’s instructions.

### Cell colony formation assay

For adherent cells, 1.5 × 10^3^ DMS273 cells 48 h after transfection were plated into 6-well plates in triplicates for each condition and then cultured for 3 weeks. Then the colonies were fixed with methanol for 30 min, followed by staining with 1.5% crystal violet for 10 min at room temperature. For suspension cells, 48 h after transfection, 10^3^ cells in 1 ml of RPMI1640 containing 10% (v/v) FBS and 0.33% (w/v) agarose were overlaid onto bottom agar consisting of 1 ml of RPMI-1640 containing 10% (v/v) FBS and 0.5% (w/v) agarose in a 6-well culture plate. Then the cells were cultured for 3 weeks. The colonies were photographed and analyzed by Image J.

### Western blot analysis and antibodies

Western blot analysis was performed as described previously [36]. The following antibodies were used in this study: anti-AKT (CST, #9272), anti-phosphorylated-AKT (Ser-473) (CST, #9271), anti-IGF1R (Proteintech, #20,254-1-AP), anti-phosphorylated-S6 Ribosomal Protein (Ser235/236) (CST, #4858), anti-S6 Ribosomal Protein (5G10) (CST, #2217), anti-PARP (CST, #9542), anti-p21 (Proteintech, 10,355-1-AP), anti-β-actin (TransGen, HC201).

### RNA pull-down assay with a biotinylated *circVAPA* probe

RNA pull-down was conducted as previously described [35]. Briefly, we designed a biotin-labeled 30nt probe against the back-spliced junction of *circVAPA* to specifically pull down *circVAPA* and its intracellular RNA-RNA complex. A biotin-labeled probe with scrambled sequence was set as a negative control. 10^7^ cells were cross-linked in ice-cold PBS buffer with 1% formaldehyde for 10 min. Upon PBS buffer removal, these cells were lysed in RNA immunoprecipitation (RIP) buffer on ice for 30 min. After sonication, the cell supernatant was harvested and divided into two equal parts for subsequent RNA pull-down after centrifugation. The biotin-labeled and control probes were incubated with the respective cell lysate for 4 h at 4 °C with gentle rotation. Identical blocked M280 Streptavidin magnetic Dynabeads (Invitrogen) were added to the above lysates and further rotated for 4 h at 4 °C. After washing with RIP buffer and RIP buffer supplemented with 500 mM NaCl, the bound RNA was isolated using TRIzol and used for RNA detection by RT-qPCR assay.

### RNA immunoprecipitation

10^7^ cells were cross-linked in ice-cold PBS buffer with 1% formaldehyde for 10 min. Then they were harvested and lysed in RIP lysis buffer, incubated with Dynabeads protein G (Invitrogen) conjugated with anti-IgG (CST, #2729) or anti-AGO2 (Sigma, SAB4200085), and rotated at 4 °C overnight. The immunoprecipitated RNAs were extracted by TRIzol reagent and further detected by RT-qPCR with specific primers.

### Dual-luciferase reporter assay

The recombinant luciferase reporter plasmids were inserted with the potential *miR-377-3p* and *miR-494-3p* binding site sequences in *circVAPA* and the 3’ UTR of *IGF1R*. When the 293T cells grew to 80% confluency, the cells were co-transfected with *miR-377-3p* and *miR-494-3p* mimics or inhibitors, luciferase reporter plasmids (*circVAPA*-WT, *circVAPA*-Mut, *IGF1R*-WT, *IGF1R*-Mut), and pRL-TK renilla luciferase vector. Activities of firefly luciferase (FL) and renilla luciferase (RL) were measured after transfection for 48 h using the Dual-Luciferase® Reporter Assay System, and the relative ratio of the FL/RL was used to reveal the interactions between *miR-377-3p*, *miR-494-3p* and *circVAPA*, *IGF1R*.

### Small cell lung cancer xenograft mouse models

Animal experiments were carried out according to a protocol approved by the Ethics Committee of Hefei Institutes of Physical Science, China Academy of Sciences [36]. Four-week-old nude mice were injected subcutaneously with 10^7^ stably transfected sh-*circVAPA* or sh-NC in DMS273 cells suspended in an equal volume of Matrigel on the both left and right flanks (n = 5 per group). When tumors volume reached 100-200 mm^3^, 0.5% carboxymethyl cellulose sodium (CMC-Na) or BMS-536924 (100 mg/kg) was administered daily by gavage for 16 consecutive days. The width and length of the tumor were measured every day for 5 weeks, and the tumor size was calculated according to the formula: volume (mm^3^) = (length × width^2^)/2. Thirty days after injection, mice were sacrificed, and tumors were harvested.

### Histological and immunohistochemical analyses

3 paired clinical SCLC tissues and harvested xenograft tumor tissues were fixed in 4% paraformaldehyde for 24 h and embedded in paraffin. The embedded tissues were cut into 4 μm thick sections and then processed for hematoxylin and eosin (H&E) staining and immunohistochemistry (IHC) staining (anti-IGF1R, CST, #14,534; anti-phosphorylated-AKT, Affinity, #AF0016; anti-Ki67, CST, #12,202).

### CircRNA RNase R and Actinomycin D treatments

For RNase R treatment, 2 μg total RNA was incubated with or without 20 U RNase R at 37 °C for 30 min. For actinomycin D treatment, 2 mg/ml actinomycin D was added to the culture medium to block RNA transcription at indicated time points. After treatment, RT-qPCR was used to assess the expression levels of *circVAPA* and *VAPA* mRNA.

### Statistical analysis

Statistical analysis was carried out using GraphPad Prism 6.0.1 (GraphPad Software). Data were listed mean ± SD of at least three independent experiments. Differences between groups were analyzed using Student’s t-test. A P-value of < 0.05 was considered to be significant.

## Results

### Identification of *circVAPA* in SCLC

**Fig. 1:**
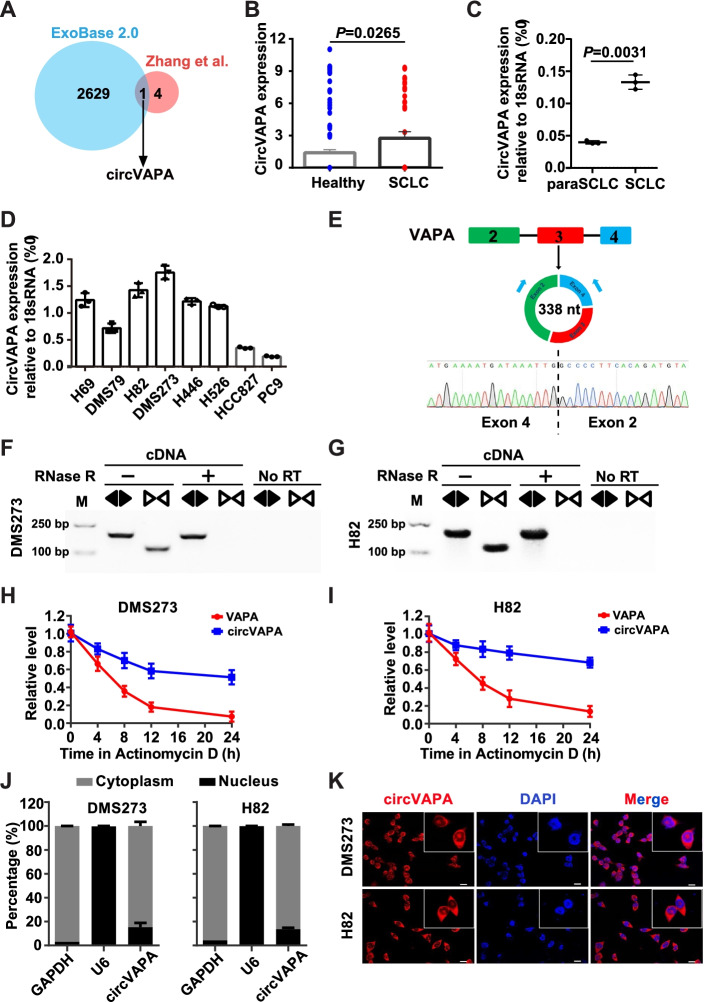
Expression and characterization of *circVAPA* in SCLC. **a** Venn diagram revealing the overlap of upregulated circRNAs based on circRNA profiling of SCLC tissues from Zhang et al. [38] and the RNA-seq data of serum samples of SCLC from exoRBase 2.0. **b** Box plots illustrating *circVAPA* expression in 36 serum samples from SCLC patients versus 118 serum samples from healthy controls. **c** The levels of *circVAPA* expression in 3 paired SCLC and matched adjacent normal tissues were detected by RT-qPCR. **d** RT-qPCR analysis of relative expression of *circVAPA* in six SCLC cell lines (H69, DMS79, H82, DMS273, H446, and H526) and two NSCLC cell lines (HCC827 and PC9). **e** The upper schematic illustration demonstrated the circularization of exons 2-4 of VAPA gene forms *circVAPA* by “head-to-tail” junction. The presence of *circVAPA* was validated by RT-PCR followed by Sanger sequencing, and the black dotted line indicates the putative back-splice site. **f and g** PCR products with divergent primers (*circVAPA*) or convergent primers (*VAPA* mRNA) in cDNA of DMS273 cells (**f**) and H82 cells (**g**), treated with or without RNase R, as assessed by the agarose gel electrophoresis. No RT, no reverse transcription. **h and i** The relative expression of *circVAPA* and *VAPA* mRNA in SCLC cells were measured after actinomycin D treatment for 0 h, 4 h, 8 h, 12 h, 16 h, 20 h, and 24 h. **j** RT-qPCR analysis of *circVAPA* in the nuclear and cytoplasmic fractions of SCLC cells. (*GAPDH* is mainly expressed in the cytoplasm and *U6* in the nucleus). **k** The subcellular localization of *circVAPA* in DMS273 and H82 cells performed with FISH. Nuclei was stained blue (DAPI) and *circVAPA* was stained red (Cy3). Scale bar, 20 μm. (All data are presented as the mean ± SD; *P < 0.05; **P < 0.01; ***P < 0.001 by two-tailed Student’s t-test). Three independent assays were performed in the above assays

Integrative analysis of the previously reported upregulated circRNAs based on circRNA profiling of six paired SCLC tissues and the RNA-seq data of serum samples from 36 SCLC patients and 118 healthy controls [37, 38] identified *circVAPA* as a significantly upregulated circRNA (Fig. 1A, B and Table S1, S2, S3). Then, we analyzed the expression of *circVAPA* in both lung cancer cell lines and human primary SCLC tissues. The endogenous *circVAPA* was significantly elevated in 3-paired SCLC tissues compared to the corresponding non-tumor controls (paraSCLC) (Fig. 1C). Similarly, *circVAPA* exhibited remarkably higher expressions in SCLC cell lines than NSCLC cell lines (Fig. 1D), suggesting that *circVAPA* is a significantly upregulated circRNA in SCLC meriting further investigation. Two SCLC cell lines (DMS273 and H82) were chosen for subsequent experiments due to the highest *circVAPA* expression levels among six SCLC cell lines (Fig. 1D). *CircVAPA* (annotated as hsa_circ_0006990 in circBase (http://www.circbase.org/) with 338 nucleotides (nt) in length, was back-spliced from exon 2-4 of the VAMP-associated protein A (VAPA) gene (Fig. 1E), which is located on human chromosome 18p11.22 [39, 40]. The putative back-spliced junction fragment of *circVAPA* was verified by PCR amplification with divergent primers from complementary DNA (cDNA) of SCLC cell lines and confirmed by Sanger sequencing (Fig. 1E). RNase R exonuclease assay examined by RT-qPCR verified that *circVAPA* was resistant to digestion (Fig. 1F, G, and Fig. S1A), consistent with the characteristics of circRNAs [41, 42]. To further evaluate the stability of *circVAPA*, actinomycin D (an inhibitor of transcription) treatment assay revealed that *circVAPA* was more stable than *VAPA* mRNA in SCLC cells (Fig. 1H, I). The function of non-coding RNA, including circRNA, is tightly and closely related to its subcellular location pattern [9, 11, 43]. FISH assay with a probe targeting the back-spliced junction of *circVAPA* and RT-qPCR analysis of nuclear and cytoplasmic RNAs revealed the predominately cytoplasmic enrichment of *circVAPA* in both DMS273 and H82 cells (Fig. 1J, K). Finally, ~ 770 and ~ 500 *circVAPA* copies per cell were determined in DMS273 and H82 cells, respectively (Fig. S1B, C). Taking above results together, our findings demonstrated that *circVAPA* was a cytoplasmic circRNA and upregulated in SCLC.

### *CircVAPA* promotes SCLC progression in vitro

**Fig. 2:**
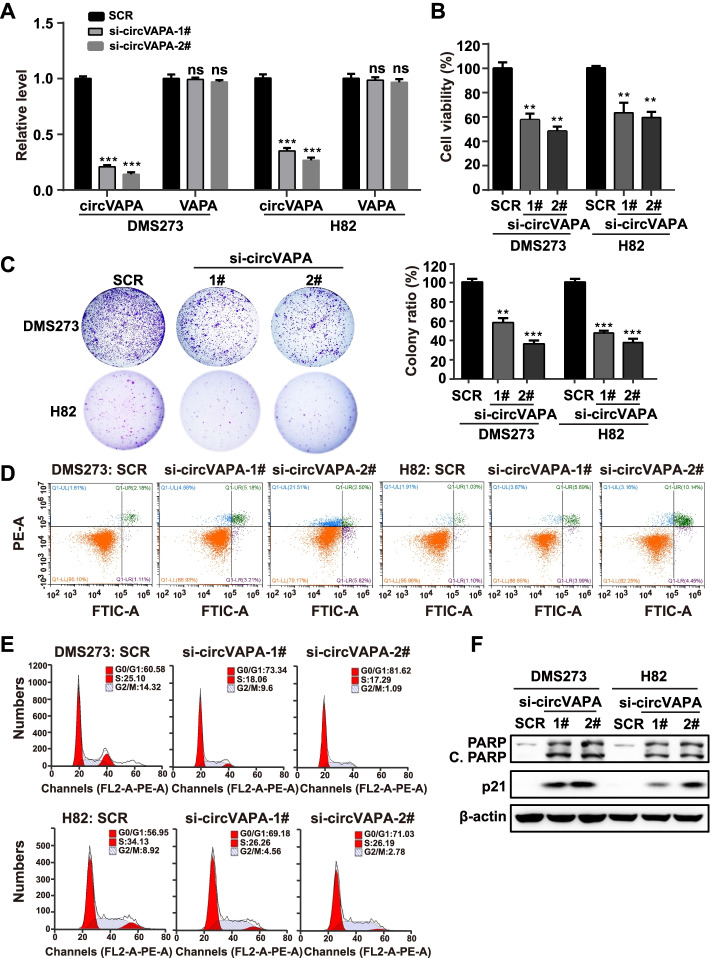
Silencing *circVAPA* suppresses cell viability, induces apoptosis of SCLC cells, and inhibits cell cycle progression in vitro. **a** RT-qPCR analysis of *circVAPA* and *VAPA* mRNA expression in SCLC cells treated with the corresponding siRNA. SCR, siRNA with scrambled sequences; si-*circVAPA* 1# and si-*circVAPA* 2#, two siRNAs specifically against the junction site of *circVAPA*. **b** Cell viability was assessed by the CellTiter-Glo assay. **c** Colony formation assay (DMS273) and soft agar colony formation assay (H82) were used to assess cell survival in SCLC cells transfected with the indicated siRNAs. **d** The apoptosis rate was analyzed by flow cytometry after depleting *circVAPA* in SCLC cells. **e** Effects on cell cycle progression analyzed by flow cytometry after downregulation of *circVAPA*. **f** The expressions of apoptosis-related protein (cleaved-PARP) and cycle-related protein (p21) were detected in SCLC cells transfected with the indicated siRNA by Western blot. β-actin was used as an internal reference. C.PARP, cleaved PARP. (All data are presented as the mean ± SD, ns, no significance; *P < 0.05; **P < 0.01; ***P < 0.001 by two-tailed Student’s t-test). Three independent assays were performed in the above assays

The aberrant expression of *circVAPA* in SCLC tissues prompted further research into the role of *circVAPA* in SCLC progression. RNA interference is a practical approach to investigating the biological functions of non-coding RNA (ncRNA) of interest. Two independent siRNAs targeting the back-spliced junction site of *circVAPA* resulted in the effective silence of *circVAPA* in SCLC cells, whereas no significant changes on *VAPA* mRNA (Fig. 2A and Fig. S2A). The CellTiter-Glo luminescent assay revealed that *circVAPA* knockdown with either siRNA decreased the SCLC cell viability (Fig. 2B). Subsequently, siRNA-mediated *circVAPA* inhibition resulted in the reduction in colony formation of SCLC cells (Fig. 2C). Flow cytometry analysis demonstrated that the depletion of *circVAPA* led to cell cycle G0/G1 arrest in SCLC cells and increased the proportion of apoptotic SCLC cells (Fig. 2D, E and Fig. S2B). Meanwhile, the p21 and cleaved PARP protein levels examined by western blot were robustly elevated upon *circVAPA* knockdown in SCLC cells (Fig. 2F).

**Fig. 3:**
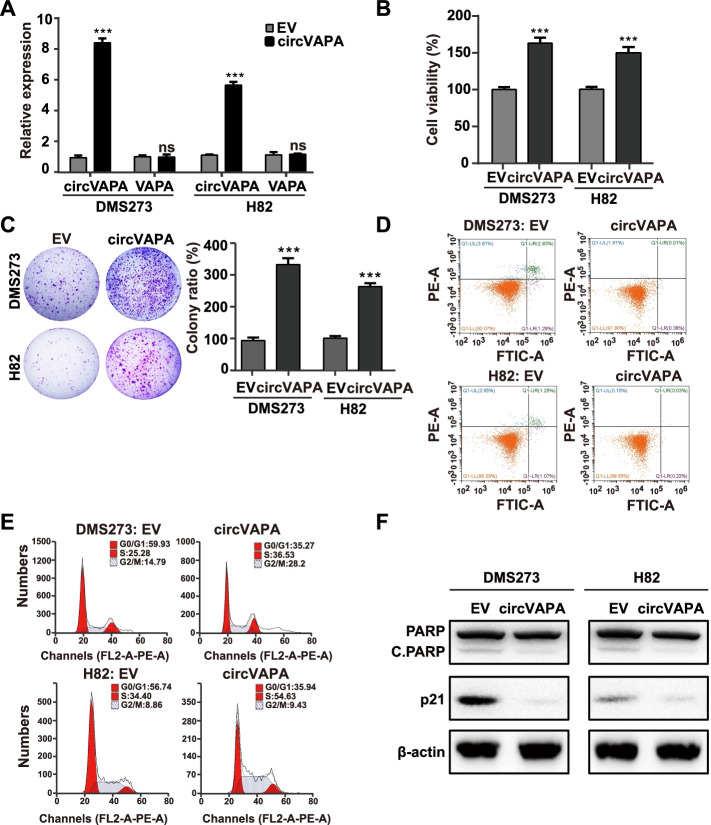
Overexpressing *circVAPA* elevates cell viability, reduces apoptosis of SCLC cells, and accelerates cell cycle progression in vitro. **a** RT-qPCR analysis of *circVAPA* and *VAPA* mRNA expression upon *circVAPA* overexpression. EV, the empty vector; *circVAPA*, the *circVAPA* overexpression plasmid. **b** Cell viability was assessed by the CellTiter-Glo assay. **c** Colony formation assay (DMS273) and soft agar colony formation assay (H82) were used to assess cell survival in SCLC cells transfected with indicated plasmid. **d** The apoptosis rate was analyzed by flow cytometry after overexpressing *circVAPA* in SCLC cells. **e** Effects on cell cycle progression analyzed by flow cytometry after *circVAPA* overexpression. **f** The expressions of apoptosis-related protein (cleaved-PARP) and cycle-related protein (p21) were detected by Western blot. β-actin was used as an internal reference. C.PARP, cleaved PARP. (All data are presented as the mean ± SD; ns, no significance; *P < 0.05; **P < 0.01; ***P < 0.001 by two-tailed Student’s t-test). Three independent assays were performed in the above assays

Furthermore, we generated the construction of overexpressing *circVAPA* (OE-*circVAPA*) with its endogenous flanking sequences including complementary *Alu* element pairs to gain the function of the ectopic *circVAPA*. As depicted in Fig. 3A, OE-*circVAPA* dramatically increased the *circVAPA* expressions and unchanged the *VAPA* mRNA levels in SCLC cells. In contrast to the effect of *circVAPA* silencing, overexpression of *circVAPA* contributed to the increase in cell viability, colony formation, and cell cycle progression, and the decrease in the proportion of apoptotic cells and the protein levels of p21 and cleaved PARP in SCLC cells (Fig. 3B-F, and Fig. S2C). Collectively, our results concluded that *circVAPA* promoted SCLC progression in vitro.

### *CircVAPA* functions as a sponge for *miR-377-3p* and *miR-494-3p*

**Fig. 4:**
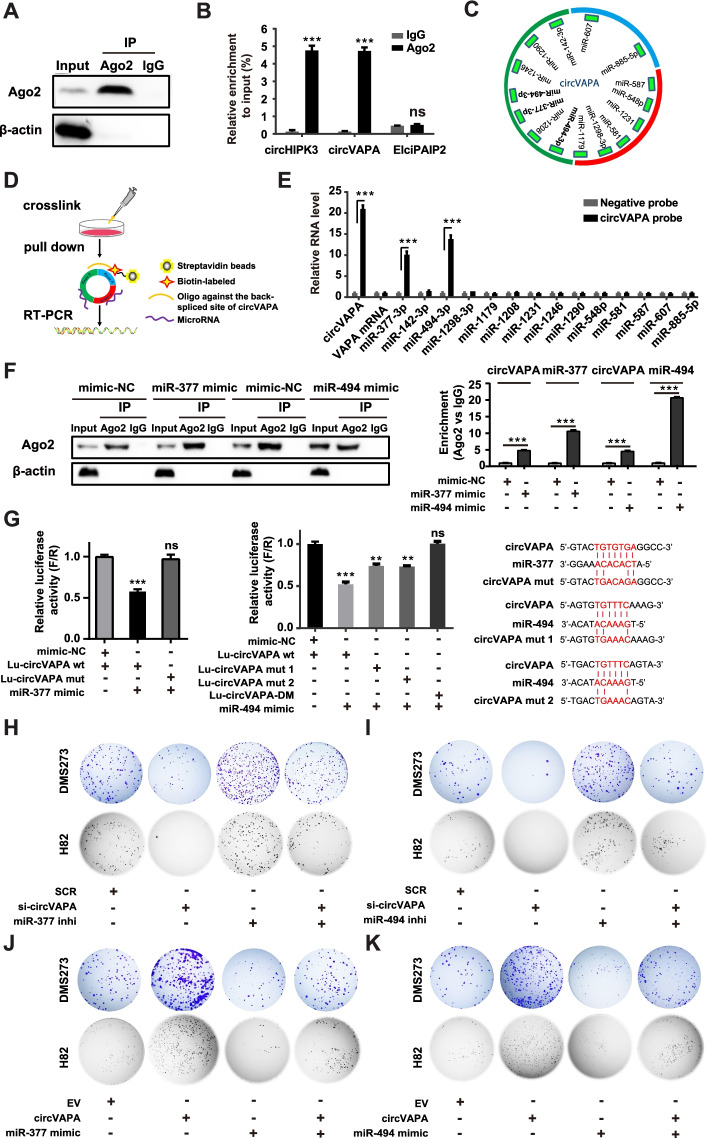
*circVAPA* functions as a sponge for *miR-377-3p* and *miR-494-3p*. **a** Anti-Ago2 RNA IP assay for detecting *circVAPA* in DMS273 cells. Anti-IgG was used as a negative control. **b** Anti-Ago2 RIP was performed in DMS273 cells. *CircHIPK3* was a positive control, while *EIciPAIP2* was a negative control. **c** Predicted putative binding miRNAs of *circVAPA* with CircInteractome. **d** Illustration of the experimental procedure for the *circVAPA* pull-down assay with biotinylated antisense oligonucleotides. **e** RNA pull-down efficiency of *circVAPA* in DMS273 cells. The enrichment of the *circVAPA* probe or negative probe was detected by RT-qPCR assay to analyze potential miRNAs associated with *circVAPA*. Negative probe, a biotin-labeled oligonucleotide with scrambled sequences; *circVAPA* probe, a biotin-labeled oligonucleotide with antisense sequences targeting the *circVAPA* junction site. **f** Anti-Ago2 RIP was performed in DMS273 cells transiently overexpressing mimics NC/*miR-377-3p*/*miR-494-3p* to detect RNA enrichment in IP complexes. Anti-IgG was used as a negative control. The expression of *circVAPA* and *miR-377-3p*/*miR-494-3p* were detected by RT-qPCR. **g** The relative luciferase activities were detected in 293T cells after co-transfection with Lu-*circVAPA*-WT or Lu-*circVAPA*-Mut and NC or *miR-377-3p*/*miR-494-3p* mimics, respectively (left). Lu-*circVAPA*-DM represents Lu-*circVAPA*-Mut 1 & 2, which contains two mutation sites. The firefly luciferase activities were measured and normalized to renilla luciferase activities (F/R). Wild-type (WT) and mutated-type (MuT) sequences of the putative binding sites between *circVAPA* and *miR-377-3p*/*miR-494-3p* were listed (right). **h–k** Colony formation assay of DMS273 cells and H82 cells transiently transfected with siRNAs, plasmids, miRNA inhibitors or mimics as indicated. *miR-377* inhi, *miR-377* inhibitor; *miR-494* inhi, *miR-494* inhibitor. SCR, siRNA with scrambled sequences; si-*circVAPA*, the co-transfection of two independent siRNAs target *circVAPA*; EV, the empty vector; *circVAPA*, the *circVAPA* overexpression plasmid; *miR-377* mimic/*miR-494* mimic, transiently overexpressing *miR-377-3p*/*miR-494-3p*, respectively; *miR-377* inhibitor/*miR-494* inhibitor, transiently suppressing *miR-377-3p*/*miR-494-3p*, respectively. (All data are presented as the mean ± SD; ns, no significance; *P < 0.05; **P < 0.01; ***P < 0.001 by two-tailed Student’s t-test). Three independent assays were performed in the above assays. *** *miR-377*/*miR-494* in this article represents *miR-377-3p*/*miR-494-3p*, respectively

Up to now, the well-characterized mechanism for cytoplasmic circRNAs is to sequester miRNA to regulate target gene expression [11, 44]. Considering that *circVAPA* is preferentially localized within the cytoplasm, we speculated that *circVAPA* participated in SCLC progression through a ceRNA mechanism. Since Ago2 is an essential mediator of circRNA-miRNA interaction [44, 45], Ago2 RNA immunoprecipitation (RIP) was conducted to validate the binding of *circVAPA* in DMS273 cells (Fig. 4A). RIP assay confirmed the direct binding of Ago2 to *circVAPA* and *circHIPK3* (a positive control), but not *EIciPAIP2* (a negative control) (Fig. 4B) [46, 47]. We then employed CircInteractome (https://circinteractome.nia.nih.gov/), a web tool for exploring the interaction between circRNAs and miRNAs [48], to predict the putative miRNA binding sites of *circVAPA*. As illustrated in Fig. 4C, 14 putative miRNA binding sites were predicted in *circVAPA* sequences, among which there are two potential binding sites for *miR-494-3p*. Afterward, a biotin-labeled oligonucleotide probe antisense to the junction site of *circVAPA* was synthesized and applied to perform RNA pull-down assay to further evidence the possible miRNA-*circVAPA* interactions (Fig. 4D). The antisense probe was able to effectively and precisely capture the endogenous *circVAPA*, as well as co-pulldown *miR-377-3p* and *miR-494-3p* compared to the control probe, but no other 12 predicted miRNAs (Fig. 4E). Moreover, RT-qPCR analysis of Ago2 RIP demonstrated that *circVAPA* was much more enriched after the overexpression of either *miR-377-3p* or *miR-494-3p* with the corresponding mimic in DMS273 cells (Fig. 4F). Furthermore, we constructed luciferase reporter gene plasmids where either the linear sequence or the sequence with the mutation of the putative binding sites for *miR-377-3p* or *miR-494-3p* of *circVAPA* was fused to the 3’ UTR of luciferase. Dual-luciferase reporter assay verified the direct binding of *circVAPA* to *miR-377-3p/miR-494-3p* in 293T cells (Fig. 4G). Notably, two putative *miR-494-3p* binding sites in *circVAPA* are both required for their interactions (Fig. 4G). Upon siRNA-mediated *circVAPA* knockdown, the expression levels for both *miR-377-3p* and *miR-494-3p* were markedly increased in DMS273 and H82 cells, while *circVAPA* overexpression caused the decreased levels of *miR-377-3p* and *miR-494-3p* (Fig. S3A). Importantly, we showed that suppressing *miR-377-3p* or *miR-494-3p* in *circVAPA*-depleted cells could rescue the inhibitory roles of *circVAPA* knockdown on cell viability and colony formation in DMS273 and H82 cells (Fig. 4H, I and Fig. S3G, H). On the contrary, *miR-377-3p* or *miR-494-3p* overexpression eliminated the promotive effects of *circVAPA* overexpression on cell viability and colony formation of DMS273 and H82 cells (Fig. 4J, K and Fig. S3I, J). These results indicated that *circVAPA* served as a molecular sponge for *miR-377-3p* and *miR-494-3p* in SCLC cells.

### IGF1R is a functional target of *miR-377-3p* and *miR-494-3p*

**Fig. 5:**
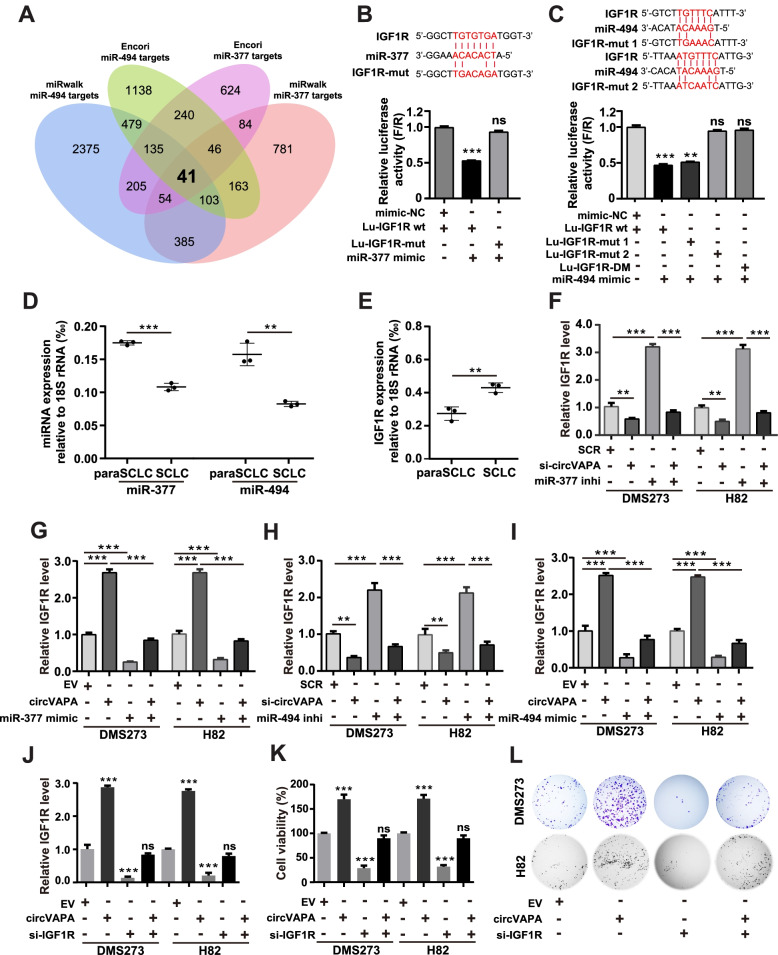
*circVAPA* facilitates SCLC cell viability via the *miR-377-3p* & *miR-494-3p*/IGF1R/AKT axis. **a** Two databases (miRwalk and ENCORI) were used to predict the potential target mRNAs of *miR-377-3p* and *miR-494-3p*. The Venn diagram shows the number of overlapping miRNAs. **b and c** Dual-luciferase reporter assay was performed to validate the interaction of *IGF1R* and *miR-377-3p* (**b**) or *miR-494-3p* (**c**). 293T cells were co-transfected with a dual-luciferase reporter vector containing the putative binding sites/ mutated sequences for *IGF1R* and *miR-377-3p*/*miR-494-3p*, *miR-377-3p*/*miR-494-3p* mimics or negative control mimics (mimic-NC), respectively. Lu-*IGF1R*-DM represents Lu-*IGF1R*-Mut 1 & 2, which contains two mutation sites. **d-e** RT-qPCR analysis of *miR-377-3p*/*miR-494-3p* (**d**) and *IGF1R* (**e**) in 3-paired SCLC and paraSCLC samples. *18S rRNA* was used as the internal control. **f-j** RT-qPCR analysis of *IGF1R* expression in SCLC cells transiently transfected with siRNAs or plasmids as indicated. **k and l** Cell viability (**k**) and colony formation (**l**) assays of SCLC cells transiently transfected with siRNAs, plasmids, miRNA inhibitors or mimics as indicated. SCR, siRNA with scrambled sequences; si-*circVAPA*, the co-transfection of two independent siRNAs target *circVAPA*; EV, the empty vector; *circVAPA*, the *circVAPA* overexpression plasmid; *miR-377 mimic*/*miR-494 mimic*, transiently overexpressing *miR-377-3p*/*miR-494-3p*, respectively; *miR-377 inhibitor*/*miR-494 inhibitor*, transiently suppressing *miR-377-3p*/*miR-494-3p*, respectively; si-*IGF1R*, an independent siRNA targeting *IGF1R*. (All data are presented as the mean ± SD; ns, no significance; *P < 0.05; **P < 0.01; ***P < 0.001 by two-tailed Student’s t-test). Three independent assays were performed in the above assays. *** *miR-377*/*miR-494* represents *miR-377-3p*/*miR-494-3p*, respectively

**Fig. 6:**
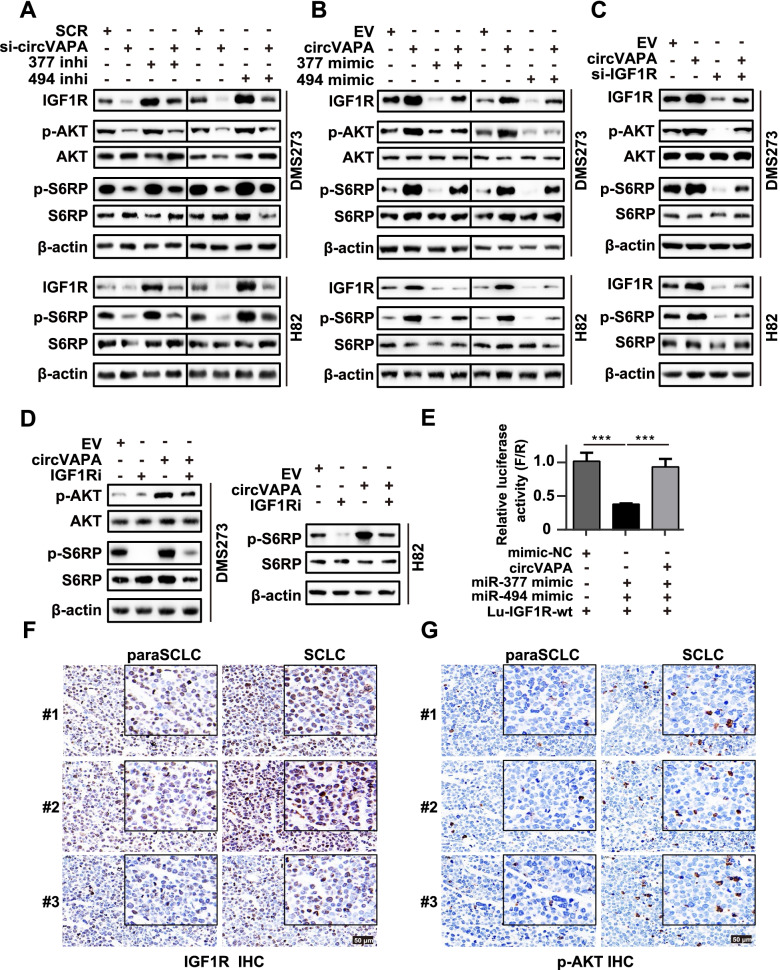
*circVAPA* promotes the progression of SCLC through the *miR-377-3p* and *miR-494-3p*/IGF1R/AKT axis. **a and b** Western blot analysis of the effects of *circVAPA* or *miR-377-3p*/*miR-494-3p* on IGF1R, AKT, and its downstream protein expression in SCLC cells. 377 inhi, *miR-377* inhibitor, 494 inhi, *miR-494* inhibitor; 377 mimic, *miR-377* mimic; 494 mimic, *miR-494* mimic. **c** Western blot analysis of the effect of overexpressing *circVAPA* or silencing *IGF1R* on AKT and its downstream protein expression in SCLC cells. **d** Western blot analysis of the effect of overexpressing *circVAPA* or IGF1R inhibitor (drug BMS-536924) on AKT and its downstream protein expression in SCLC cells. **e** The relative luciferase activities were detected in 293T cells after co-transfection with Lu-*IGF1R*-WT and mimic-NC or the *circVAPA* overexpression plasmid or *miR-377-3p*/*miR-494-3p* mimics, respectively. The firefly luciferase activities were measured and normalized to renilla luciferase activities (F/R). **f-g** Representative images of immunohistochemistry analysis of IGF1R (**f**) and p-AKT (**g**) in three independent SCLC cases. Scale bar, 50 μm. SCR, siRNA with scrambled sequences; si-*circVAPA*, the co-transfection of two independent siRNAs target *circVAPA*; EV, the empty vector; *circVAPA*, the *circVAPA* overexpression plasmid; *miR-377* mimic/*miR-494* mimic, transiently overexpressing *miR-377-3p*/*miR-494-3p*, respectively; *miR-377* inhibitor/*miR-494* inhibitor, transiently suppressing *miR-377-3p*/*miR-494-3p*, respectively; si-*IGF1R*, an independent siRNA targeting *IGF1R*; IGF1Ri, the addition of IGF1R inhibitor (drug BMS-536924). (All data are presented as the mean ± SD; ns, no significance; *P < 0.05; **P < 0.01; ***P < 0.001 by two-tailed Student’s t-test). Three independent assays were performed in the above assays. *** *miR-377*/*miR-494* in this article represents *miR-377-3p*/*miR-494-3p*, respectively

Since *circVAPA* could behave as a ceRNA against *miR-377-3p* and *miR-494-3p*, we set out to identify the downstream targets of miRNAs. The potential targets of *miR-377-3p* and *miR-494-3p* were predicted based on two databases (miRWalk (http://mirwalk.umm.uni-heidelberg.de/) and ENCORI (https://rna.sysu.edu.cn/encori/rriPathways.php)) [49, 50], revealing that *miR-377-3p* and *miR-494-3p* shared 41 common candidate genes (Fig. 5A, Table S4). Integrative analysis of significantly upregulated target genes in SCLC from the dataset (GSE149507) and the 41 common candidates ultimately focused on ten potential target genes. IGF1R, as an indispensable player in SCLC [28–30], was further confirmed as the only common target of *miR-377-3p* and *miR-494-3p*, evidenced by that the *IGF1R* mRNA level was significantly decreased or increased upon the transfection of *miR-377-3p/miR-494-3p* mimics or inhibitors, respectively (Fig. S4). We further unveiled that *miR-377-3p*/*miR-494-3p* combined with *IGF1R* mRNA wild type at the molecular level, but not with *IGF1R* mRNA carrying mutations in the putative binding sites for *miR-377-3p/miR-494-3p* in 293T cells by dual-luciferase reporter assay (Fig. 5B, C). Notably, there are 2 putative binding sites (Positions 2199–2204, 5648–5654) for *miR-494-3p* in the 3’ UTR of *IGF1R*, and only the site 2 rescued *miR-494-3p*-induced inhibitory effect on luciferase activity (Fig. 5C). Next, we examined the *miR-377-3p*/*miR-494-3p* and *IGF1R* expression in SCLC clinical samples and found that *miR-377-3p*/*miR-494-3p* were both down-regulated in SCLC compared to paraSCLC, whereas the *IGF1R* mRNA showed the opposite trend (Fig. 5D, E). Furthermore, silencing *miR-377-3p*/*miR-494-3p* with their corresponding inhibitors robustly increased the IGF1R expression at mRNA and protein levels in DMS273 and H82 cells (Fig. 5F, H and Fig. 6A and Fig. S5B). Conversely, overexpression of *miR-377-3p* and *miR-494-3p* with their mimics significantly reduced the mRNA and protein levels of IGF1R in SCLC cells (Fig. 5G, I and Fig. 6B and Fig. S5B). These results together indicated that IGF1R was the direct and functional target of *miR-377-3p* and *miR-494-3p*.

### *CircVAPA* facilitates SCLC cell viability via the *miR-377-3p* &* miR-494-3p*/IGF1R/AKT axis

To test whether *circVAPA* behaved as a ceRNA targeting *miR-377-3p* and *miR-494-3p* to regulate the expression level of *IGF1R*, dual-luciferase reporter assay was carried out to assess the interaction between *circVAPA*, *miR-377-3p* & *miR-494-3p*, and *IGF1R*. The ectopic *circVAPA* overexpression reversed the suppression of luciferase activities caused by *miR-377-3p* and *miR-494-3p* overexpression, demonstrating that *circVAPA* served as a ceRNA against *miR-377-3p* and *miR-494-3p* to relieve the inhibition of *IGF1R* expression in 293T cells (Fig. 6E). We also evaluated the copies per cell for *miR-377-3p*, *miR-494-3p* and *IGF1R* mRNA in DMS273 and H82 cells, and found that the average molecular ratio is approximately 4.5:1:9 (Fig. S5C-F). IGF1R has been reported to activate the PI3K/AKT signaling [51]. We wondered whether *circVAPA* regulated the PI3K/AKT signaling pathway via the *miR-377-3p* & *miR-494-3p*/*IGF1R* axis to promote SCLC progression. First, we showed that the *miR-377-3p* or *miR-494-3p* inhibitors could rescue the decrease in the mRNA and protein levels of IGF1R and its downstream targets, phosphorylated AKT (p-AKT) and phosphorylated S6 ribosomal protein (p-S6RP) expression upon *circVAPA* silencing in SCLC cells (Fig. 5F, H and Fig. 6A). Notably, the p-AKT level was too low to be detected by western blot in H82 cells according to our previously reported study [36]. Conversely, the promotive effects of *circVAPA* on the *IGF1R* mRNA, IGF1R protein, p-AKT, and p-S6RP protein expression could be abolished by the *miR-377-3p* or *miR-494-3p* mimics (Fig. 5G, I and Fig. 6B). Importantly, IGF1R has been demonstrated as a potential target, and inhibition of IGF1R in SCLC cells displays a promising antitumor effect [29, 30, 51]. IGF1R inhibition with either siRNA or BMS-536924, an ATP-competitive IGF1R inhibitor, could recover the *circVAPA*-induced promoting phenotype of cell viability, colony formation, *IGF1R* mRNA, and the protein levels of IGF1R, p-AKT and p-S6RP (Fig. 5J-L, Fig. 6C, D and Fig. S6A-D). The IGF1R and p-AKT protein levels examined by IHC staining, are both upregulated in SCLC compared to paraSCLC (Fig. 6F, G). Taking these results together, *circVAPA* facilitated SCLC cell viability and colony formation by activating the IGF1R/AKT axis by sequestering *miR-377-3p* and *miR-494-3p*.

### *CircVAPA* facilitates SCLC proliferation through regulating IGF1R in vivo and in vitro

**Fig. 7:**
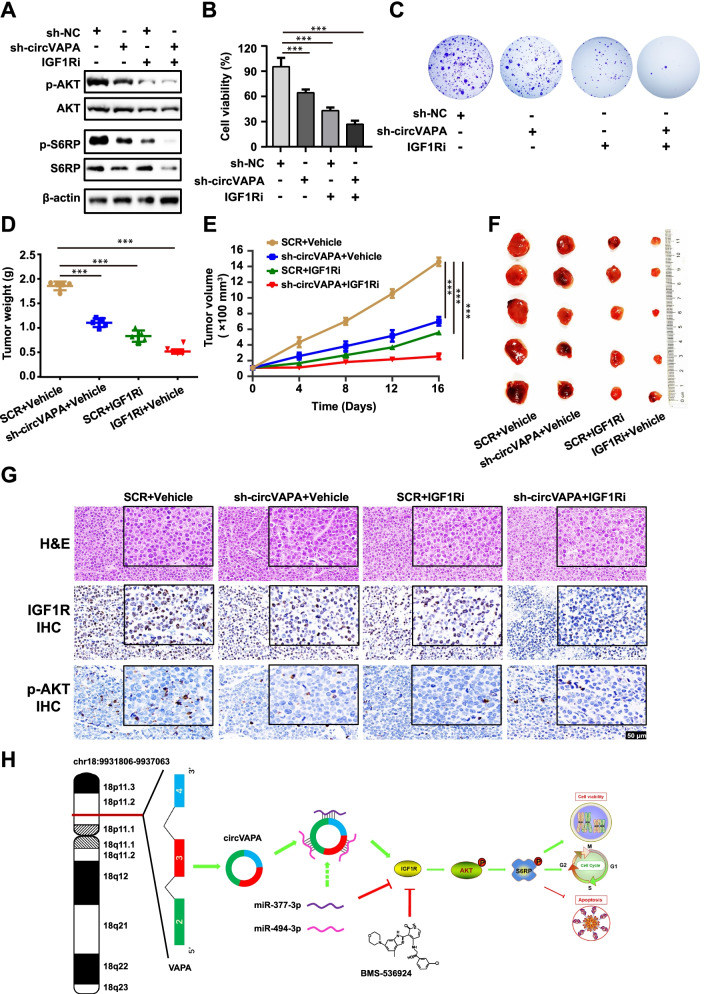
*CircVAPA* facilitates SCLC proliferation through regulating IGF1R in vivo and in vitro. **a** Western blot analysis of the effect of SCLC stable cell line with *circVAPA* knockdown or the control with or without IGF1R inhibitor (drug BMS-536924) on AKT and its downstream protein expression. **b-c** Cell viability (**b**) and colony formation (**c**) assays of the SCLC stable cell line with *circVAPA* knockdown or the control with or without IGF1R inhibitor (drug BMS-536924). **d-f** Therapeutic efficacy of *circVAPA* depletion and IGF1R inhibitor (drug BMS-536924) as single-agents or in combination in vivo (*n* = 5 for each group). Tumor weights (**d**), tumor volume curves (**e**), and tumor photos (**f**) of xenograft tumors treated with *circVAPA* depletion and IGF1R inhibitor alone or in combination. **g** Immunohistochemistry analysis of IGF1R and p-AKT in tumors. Scale bar, 50 μm. **h** Model patterns of *circVAPA*/*miR-377-3p* & *miR-494-3p*/IGF1R/AKT axis. Vehicle, negative control cells for silencing *circVAPA*; sh-*circVAPA*, stable cell line with lentivirus shRNA to knockdown *circVAPA*; IGF1Ri, the addition of IGF1R inhibitor (drug BMS-536924). (All data are presented as the mean ± SD; ns, no significance; *P < 0.05; **P < 0.01; ***P < 0.001 by two-tailed Student’s t-test). Three independent assays were performed in the above assays

BMS-536924, a small molecule inhibitor targeting IGF1R, has been confirmed to suppress IGF1R phosphorylation and block IGF1R-mediated activation of AKT signaling cascades [33]. Addition of BMS-536924 could attenuate the expression of p-AKT and p-S6RP protein, but this negative regulation of AKT signaling cascade was diminished upon *circVAPA* overexpression (Fig. 6D). Moreover, we established a DMS273 stable cell line with lentivirus shRNA to silence *circVAPA* and confirmed the effective knockdown efficiency of *circVAPA* (Fig. S6E). Treatment with BMS-536924 addition or *circVAPA* inhibition displayed a moderate impact on the reduction of AKT signaling cascade, whereas a combination treatment with both exhibited the maximal repressive effect (Fig. 7A and Fig. S6B). In support of the western blot results, the BMS-536924 addition alone or *circVAPA* silencing displayed an appreciable effect on blocking the cell viability and colony formation of DMS273 and H82 (Fig. 7B, C and Fig. S6C, D). However, the combination of BMS-536924 treatment and *circVAPA* depletion achieved the maximal inhibitory effects on cell viability and colony formation in DMS273 and H82 (Fig. 7B, C and Fig. S6C, D).

To explore the biological functions of *circVAPA* in SCLC in vivo, we then performed the subcutaneous injection of *circVAPA* knockdown and control DMS273 cells into nude mice to investigate the in vivo roles of *circVAPA*. Cells with stable *circVAPA* knockdown formed significantly smaller tumors’ size, volume, and weight than the control (Fig. 7D, E, F and Fig. S6E). IHC staining demonstrated that the levels of Ki67, IGF1R, p-AKT were significantly decreased in the tumors derived from cells with stable *circVAPA* knockdown compared to the control (Fig. 7G and Fig. S6F). The IGF1R inhibitor BMS-536924 exhibited the enhancement in the *circVAPA*-silencing-caused suppressive effects on cell viability, colony formation, p-AKT, and p-S6RP in vitro and tumors’ size, volume, and weight in vivo, suggesting that BMS-536924 and *circVAPA* depletion might achieve a potential synergistic effect on the treatment of SCLC (Fig. 7A-G and Fig. S6A-D, F). These results indicated that *circVAPA* promoted SCLC proliferation by targeting IGF1R in vivo.

## Discussion

SCLC is an aggressive malignancy with high mortality and poor prognosis [2, 6]. Even though a significant improvement in chemotherapy efficacy, the clinical outcome of SCLC patients remains poor, mainly due to recurrence and drug resistance [1, 2, 6]. Therefore, it is essential to identify novel biomarkers and effective therapeutic targets for SCLC. Emerging shreds of evidence uncover that circRNAs are dysregulated in diverse human cancers, and these aberrantly expressed circRNAs may be associated with the oncogenesis and progression of multiple cancers [10, 11]. Nevertheless, researches on the role and molecular mechanism of circRNAs in SCLC are still in its infancy.

Previous studies have revealed that *circVAPA* played an oncogenic role in colorectal and breast cancer [52, 53]. Li et al. discovered that *circVAPA* facilitated colorectal cancer progression by sponging *miR-101* [52]. Additionally, we have also found that RNA pull-down with the probe against the back-spliced junction of *circVAPA* displayed the enrichment of *miR-101* and *miR-101* did not affect the IGF1R/PI3K/AKT signaling pathway in SCLC (Fig. S3D-F). Zhou’s team reported that *miR-130a-5p* suppressed breast cancer cell migration and invasion, and *circVAPA* served as a sponge for *miR-130a-5p* [53]. However, no detailed studies on the role of *circVAPA* in SCLC were performed. With a series of molecular, cellular and biochemical experiments, we propose a working model (Fig. 7H) that *circVAPA* promotes SCLC progression in vitro and in vivo by modulating the *miR-377-3p* and *miR-494-3p*/IGF1R/AKT axis, expanding the knowledge about circRNAs in SCLC.

It has been widely accepted that most circRNAs could act as a ceRNA to regulate mRNA expression via competitively adsorbing miRNAs [23, 44, 46]. Herein, we verified that *circVAPA* could be enriched in the Ago2 RIP fraction, which is necessary for *circVAPA* to act as a miRNA sponge. We subsequently predicted that potential miRNAs might bind to *circVAPA* and confirmed the interaction between *circVAPA* and *miR-377-3p*/*miR-494-3p* by various approaches, such as RNA pull-down assay, Ago2 RIP assay in *circVAPA* over-expressed SCLC cells, and dual-luciferase reporter assay.

As miRNAs exert their regulatory functions by targeting downstream mRNAs, we explored *miR-377-3p*/ *miR-494-3p* downstream mRNA. *miR-377* and *miR-494* have been found to be related to human cancer and exerted important roles in human cancer by their respective target mRNAs [54, 55]. For example, Li found that *miR-377* expression was significantly downregulated in esophageal squamous cell carcinoma (ESCC), and *miR-377* expression was positively correlated with ESCC patient survival [54]. Moreover, *miR-377* inhibits the initiation and progression of esophageal squamous cell carcinoma through the negative regulation of CD133 and VEGF [54]. Additionally, *miR-494* suppresses gastrointestinal stromal tumor (GIST) cell proliferation via targeting KIT, a critical regulatory protein in the development and progression of GIST [55]. In this study, we have explored the common downstream targets of *miR-377-3p*/*miR-494-3p* using the miRWalk and ENCORI prediction tools [49, 50]. Of note, *IGF1R* was predicted to be a potential mRNA target in the downstream pathway of *miR-377-3p*/*miR-494-3p*. Then we utilized dual-luciferase reporter assay based on the putative binding sites of *miR-377-3p*/*miR-494-3p* on *IGF1R*, which verified that *IGF1R* was the common target of *miR-377-3p/miR-494-3p*.

Numerous circRNAs are significantly associated with clinicopathological characteristics of cancer by regulating the PI3K/AKT signaling pathway [51]. Given that IGF1R plays vital roles in PI3K-AKT signaling cascades [29, 30, 51], we speculated that the mechanism of action of *circVAPA* might affect the PI3K-AKT signaling cascades. As a result, we aimed to investigate whether IGF1R and its downstream PI3K-AKT signaling cascade could be activated by *circVAPA* altered. The effect of *circVAPA* knockdown on IGF1R and the PI3K-AKT signaling cascades in SCLC cells could be reversed by co-transfection of *miR-377-3p*/*miR-494-3p* inhibitors. On the contrary, the impact of *circVAPA* over-expression on IGF1R and the PI3K-AKT signaling cascades in SCLC cells could be rescued by co-transfection of *miR-377-3p*/*miR-494-3p* mimics or IGF1R inhibition. These in vitro experiments revealed that *circVAPA* might act as a molecular sponge to relieve the suppressive effects of *miR-377-3p*/*miR-494-3p* on their downstream target IGF1R.

The IGF1/IGF1R signaling axis-mediated pathway has been implicated in the tumorigenesis and development of multiple malignancies, and IGF1R inhibitor emerged as a potential anticancer agent [27, 28]. We revealed that overexpressing *circVAPA* could recover the reduction in IGF1R activity and eliminate the PI3K-AKT signaling cascades caused by BMS-536924 stimulation in SCLC. Moreover, BMS-536924 could block IGF1R activity and downstream signaling cascades, and this negative regulation could be further enhanced by knocking down *circVAPA* in vitro. Furthermore, the combination of *circVAPA* inhibition and BMS-536924 addition exhibited a better therapeutic efficacy in vivo than *circVAPA* silencing or BMS-536924 alone.

In conclusion, our study demonstrated that *circVAPA* might serve as an oncogenic circRNA and promote the progression of SCLC. Mechanistically, *circVAPA* acted as a sponge for *miR-377-3p*/*miR-494-3p* to elevate the IGF1R expression to activate the PI3K/AKT signaling pathway. Additionally, the combination of *circVAPA* inhibition and BMS-536924 displayed a more potent antitumor effect in SCLC. We hope to develop clinical protocols of combinations of *circVAPA* inhibition and BMS-536924 addition for treating SCLC with *circVAPA* upregulation.

## Conclusions

In summary, our work may provide novel insights into the mechanisms involved in SCLC progression, as well as a promising biomarker for SCLC. We advocate that the *circVAPA*/*miR-377-3p* and *miR-494-3p*/IGF1R/AKT axis may serve as a potent therapeutic target in SCLC.

## Supplementary Information

**Additional file 1: Figure S1.** (a) The relative expression of *circVAPA*, *CircHIPK3 *and* 18S* *rRNA* in SCLC cells were detected by RT-qPCR after RNase R treatment. *CircHIPK3* was a positive control, while *18S* *rRNA *was a negative control. (b-c) Quantification of *circVAPA* copy numbers in DMS273 and H82 cell lines. The red and blue dots indicate the Ct value and the amount of RNA in DMS273 and H82 SCLC cells, respectively. More experimental details are provided in the Methods section. (d) Prediction of the potential translation ability of *circVAPA*. An interaction model shows that *circVAPA *harbors potential internal ribosome entry site (IRES), but not open reading frame (ORF), which is analyzed by the circRNADb and ORFfinder databases. (All data are presented as the mean ± SD; ns, no significance; ***P < 0.001 by two-tailed Student’s t-test). **Figure S2.** (a) Schematic diagram of knocking down *circVAPA *using two independent siRNAs target *circVAPA *junction. (b-c) The apoptosis rate was analyzed by flow cytometry after downregulation (b) or overexpression (c) of *circVAPA in *SCLC cells. (All data are presented as the mean ± SD; ***P < 0.001 by two-tailed Student’s t-test). Three independent assays were performed in the above assays. **Figure S3.** (a) The effect of *circVAPA* on *miR-377-3p* and *miR-494-3p *expression levels in SCLC cells were detected by RT-qPCR. (b and c) RT-qPCR analysis of *miR-377-3p* (b) and *miR-494-3p* (c) expression in SCLC cells transiently transfected with the corresponding mimic, respectively. (d) After RNA pull-down by biotin-labeled *circVAPA* probe or negative probe, the *miR-101* enrichment level in DMS273 cells was detected by RT-qPCR. (e) RT-qPCR analysis of *miR-101* expression in SCLC cells transiently transfected with the corresponding mimic. (f) Western blot analysis of the effect of *miR-101 *on IGF1R, AKT, and its downstream protein expression in DMS273 (left) and H82 (right) SCLC cells. β-actin was used as an internal reference. (g-j) Cell viability analysis of SCLC cells transiently transfected with siRNAs, plasmids, miRNA inhibitors or mimics as indicated. SCR, siRNA with scrambled sequences; si-*circVAPA*, the co-transfection of two independent siRNAs target *circVAPA*; EV, the empty vector; *circVAPA*, the *circVAPA* overexpression plasmid; *miR-377* mimic/*miR-494* mimic, transiently overexpressing *miR-377-3p*/*miR-494-3p*, respectively; *miR-377* inhibitor/*miR-494* inhibitor, transiently suppressing *miR-377-3p*/*miR-494-3p*, respectively. (All data are presented as the mean ± SD; N.S., not significant; *P < 0.05; **P < 0.01; ***P <0.001 by two-tailed Student’s t-test). Three independent assays were performed in the above assays. **Figure S4.** (a-d) RT-qPCR analysis of common candidate targets of *miR-377-3p* & *miR-494-3p *in SCLC cells transiently transfected with the corresponding mimics or inhibitors. The common targets of *miR-377-3p* and *miR-494-3p* were predicted by miRWalk and ENCORI, and they were further in comparison with upregulated genes in the GEO dataset GSE149507. Overlapped genes matching the condition where |fold change| > 1 and *p*-value < 0.001 were selected. NC, negative control cells for overexpressing or suppressing *miR-377-3p*/*miR-494-3p*; *miR-377* mimic/*miR-494* mimic, transiently overexpressing *miR-377-3p*/*miR-494-3p*, respectively; *miR-377* inhibitor/*miR-494* inhibitor, transiently suppressing *miR-377-3p*/*miR-494-3p*, respectively. (All data are presented as the mean ± SD; **P < 0.01; ***P <0.001 by two-tailed Student’s t-test). Three independent assays were performed in the above assays. **Figure S5.** (a) RT-qPCR analysis of *miR-377-3p*/*miR-494-3p* expression in DMS273 cells transiently transfecting the indicated mimics. (b) RT-qPCR analysis of the effects of *miR-377-3p*/*miR-494-3p* on *IGF1R* in SCLC cells. (c-f) Quantification of *miR-377-3p*, *miR-494-3p,* and *IGF1R* copy numbers in DMS273 and H82 cell lines. The red and blue dots indicate the Ct value and the amount of RNA in DMS273 and H82 SCLC cells, respectively. More experimental details are provided in the Methods section. NC, negative control cells for overexpressing or suppressing *miR-377-3p*/*miR-494-3p*; *miR-377 *mimic/*miR-494* mimic, transiently overexpressing *miR-377-3p*/*miR-494-3p*, respectively; *miR-377* inhibitor/*miR-494* inhibitor, transiently suppressing *miR-377-3p*/*miR-494-3p*, respectively. (All data are presented as the mean ± SD; ***P < 0.001 by two-tailed Student’s t-test). Three independent assays were performed in the above assays. **Figure S6.** (a) Cell viability analysis of the effect of *circVAPA* overexpression or IGF1R inhibitor (drug BMS-536924) on SCLC cells. (b) Western blot analysis of the effects of *circVAPA* depletion or IGF1R inhibitor (drug BMS-536924) addition on S6RP and p-S6RP protein expression in H82 cells. (c and d) Cell viability (c) and colony formation (d) analysis of the H82 cells with *circVAPA* knockdown or the control with or without IGF1R inhibitor. (e) RT-qPCR analysis of *circVAPA* and *VAPA* mRNA expression in stable DMS273 cells transfected with the lentivirus-shRNA. Vector was the negative control for knocking down *circVAPA*. (f) Immunohistochemistry analysis of ki67 in tumors. Scale bar, 50 μm. si-*circVAPA*, the co-transfection of two independent siRNAs target *circVAPA*; IGF1Ri, the addition of IGF1R inhibitor (drug BMS-536924). (All data are presented as the mean ± SD; ns, no significance; ***P < 0.001 by two-tailed Student’s t-test). Three independent assays were performed in the above assays.

**Additional file 2: Table S1.** The differentially expressed circRNAs between serum samples from 36 SCLC patients and that from 118 healthy controls.

**Additional file 3: Table S2.** The upregulated circRNAs previously reported based on circRNA profiling of six paired SCLC tissues.

**Additional file 4: Table S3.** The expression of *circVAPA* in serum samples.

**Additional file 5: Table S4.** Common candidate targets of *miR-377-3p* and *miR-494-3p*.

**Additonal file 6: Table S5.** Oligos used in the study.

## Data Availability

Quantitative data that support this study are available within this article and its supplementary files. All other data that support the findings of this study are available from the corresponding author on reasonable request.

## Abbreviations

AKT: Protein kinase B
cDNA: Complementary DNA
CeRNA: Competitive endogenous RNA
CircRNA: Circular RNA
IGF1R: Insulin-like growth factor 1 receptor
ncRNA: Non-coding RNA
NSCLC: Non-small cell lung cancer
OE-*circVAPA*: Overexpressing *circVAPA*
PI3K: Phosphoinositide 3-kinase
Pre-mRNA: Precursor mRNA
RBP: RNA binding protein
RT-qPCR: Real-time quantitative PCR
S6RP: S6 ribosomal protein
SCLC: Small cell lung cancer
shRNA: Short hairpin RNA
siRNA: Short interfering RNA
VAPA: VAMP (Vesicle-associated membrane protein)-associated protein A
