# Supplementary figures and images for: CircVAPA promotes small cell lung cancer progression by modulating the miR-377-3p and miR-494-3p/IGF1R/AKT axis
Source: Mol Cancer. 2022 Jun 6;21:123. doi: 10.1186/s12943-022-01595-9 (PMC9172052; doi:10.1186/s12943-022-01595-9)

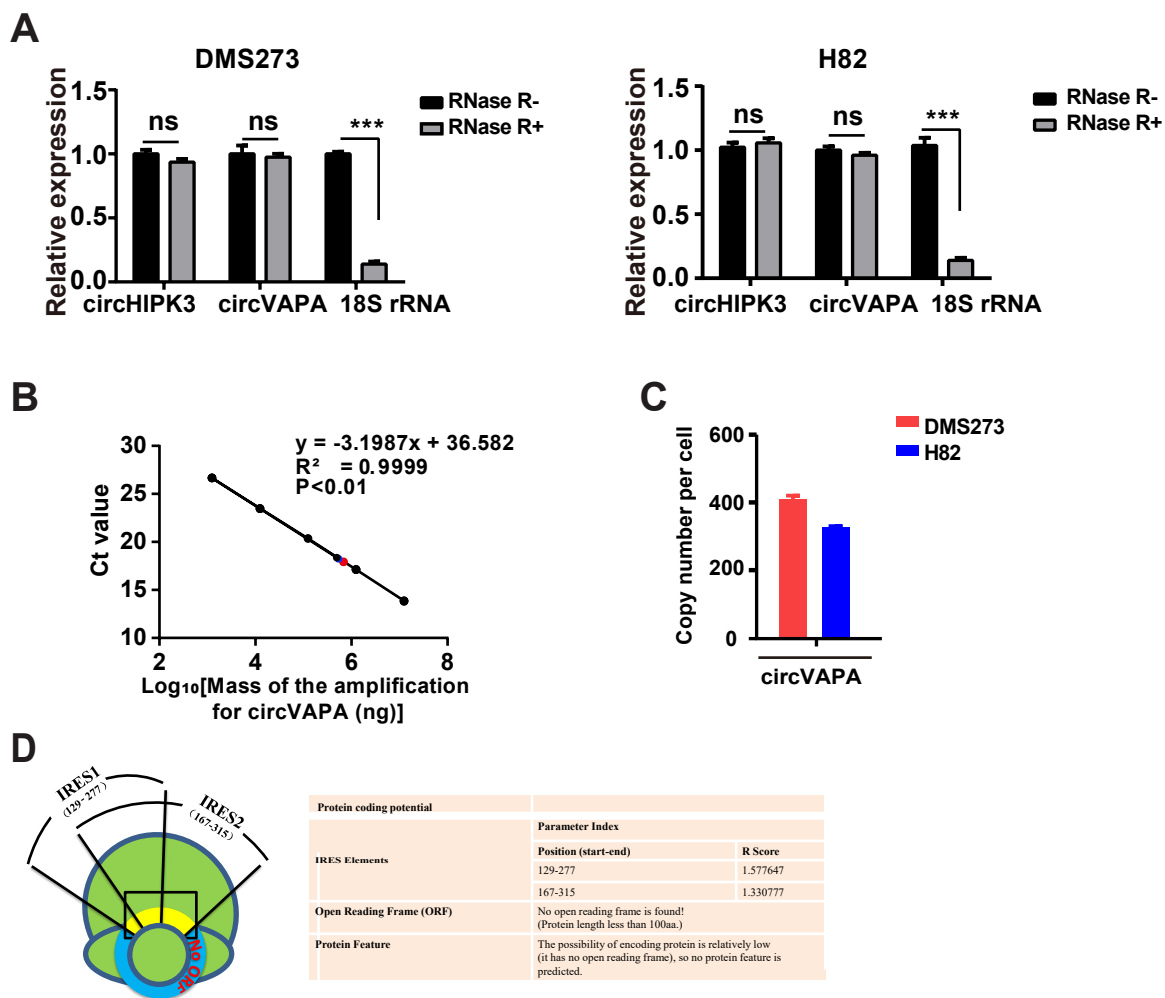

**Figure S1.**

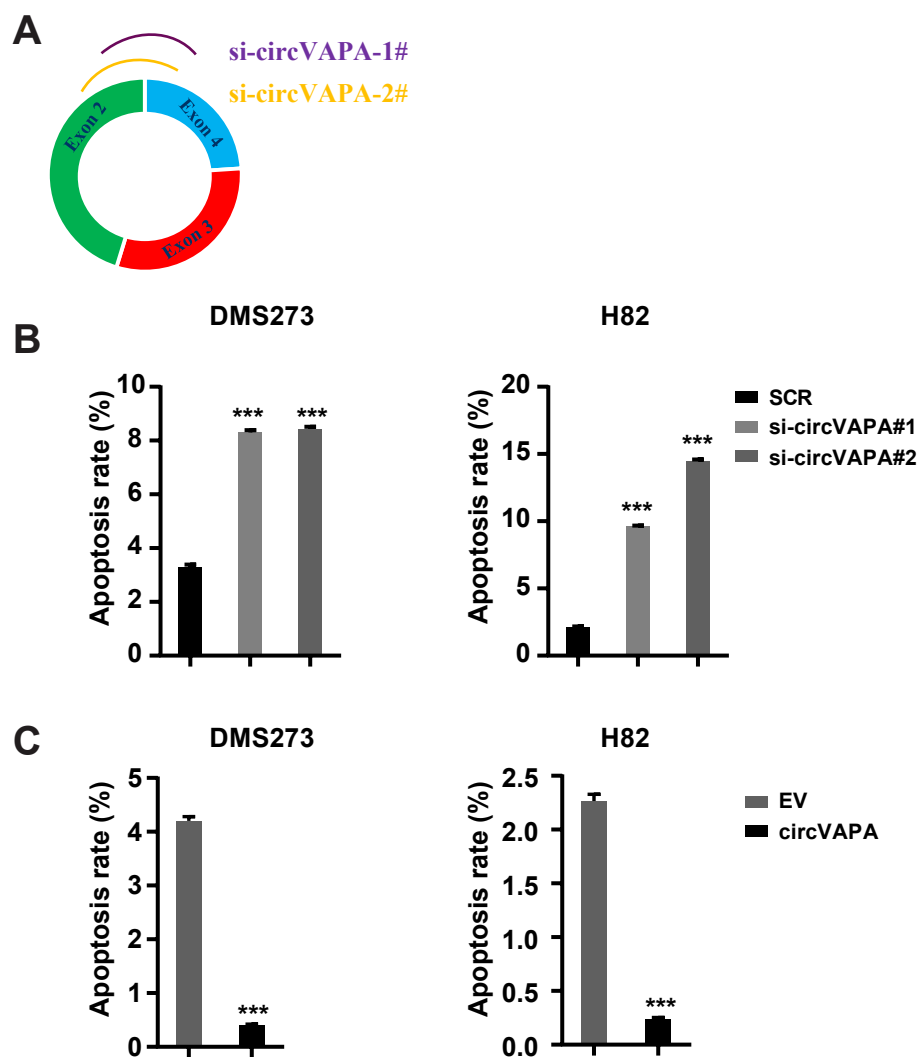

**Figure S2.**

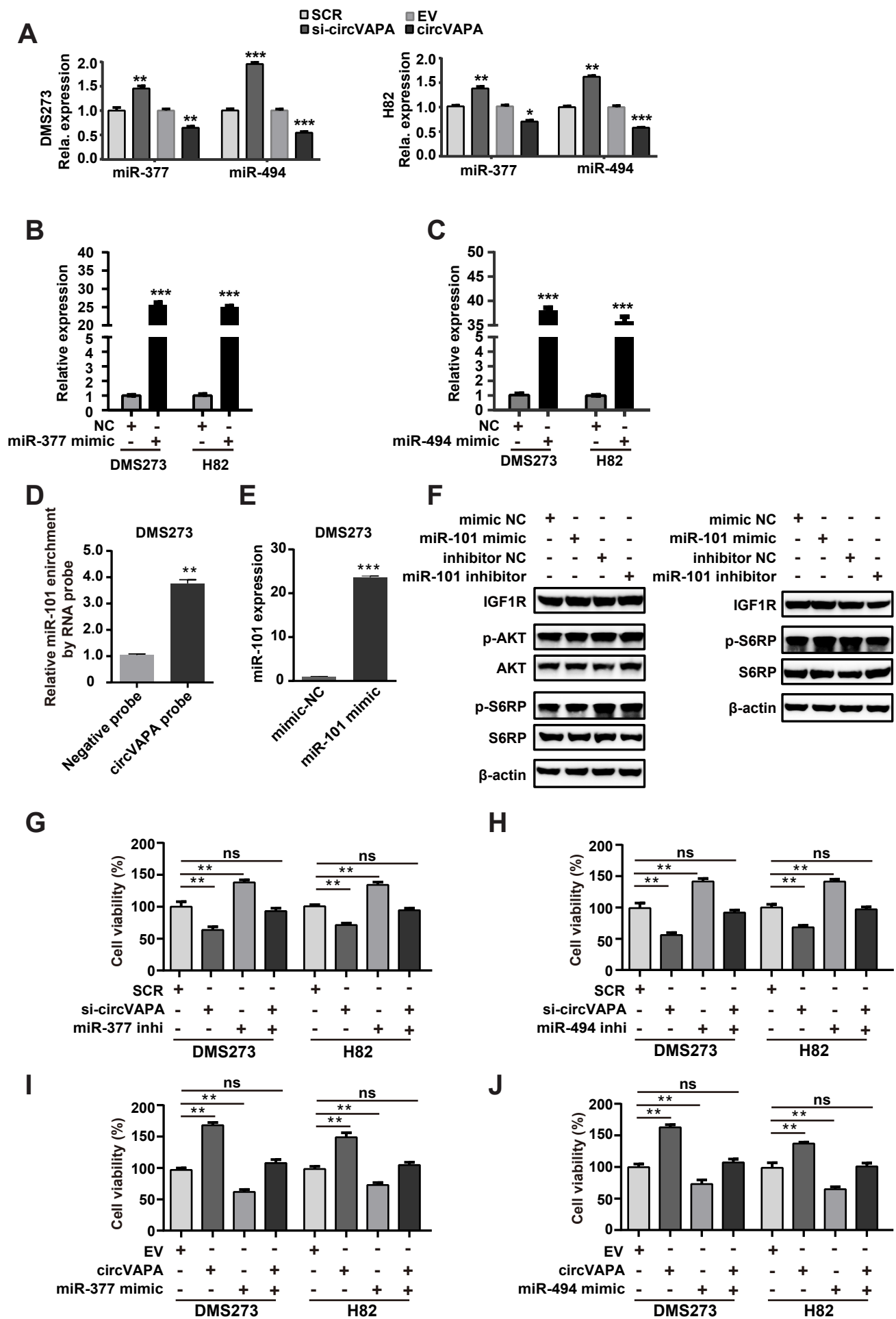

**Figure S3.**

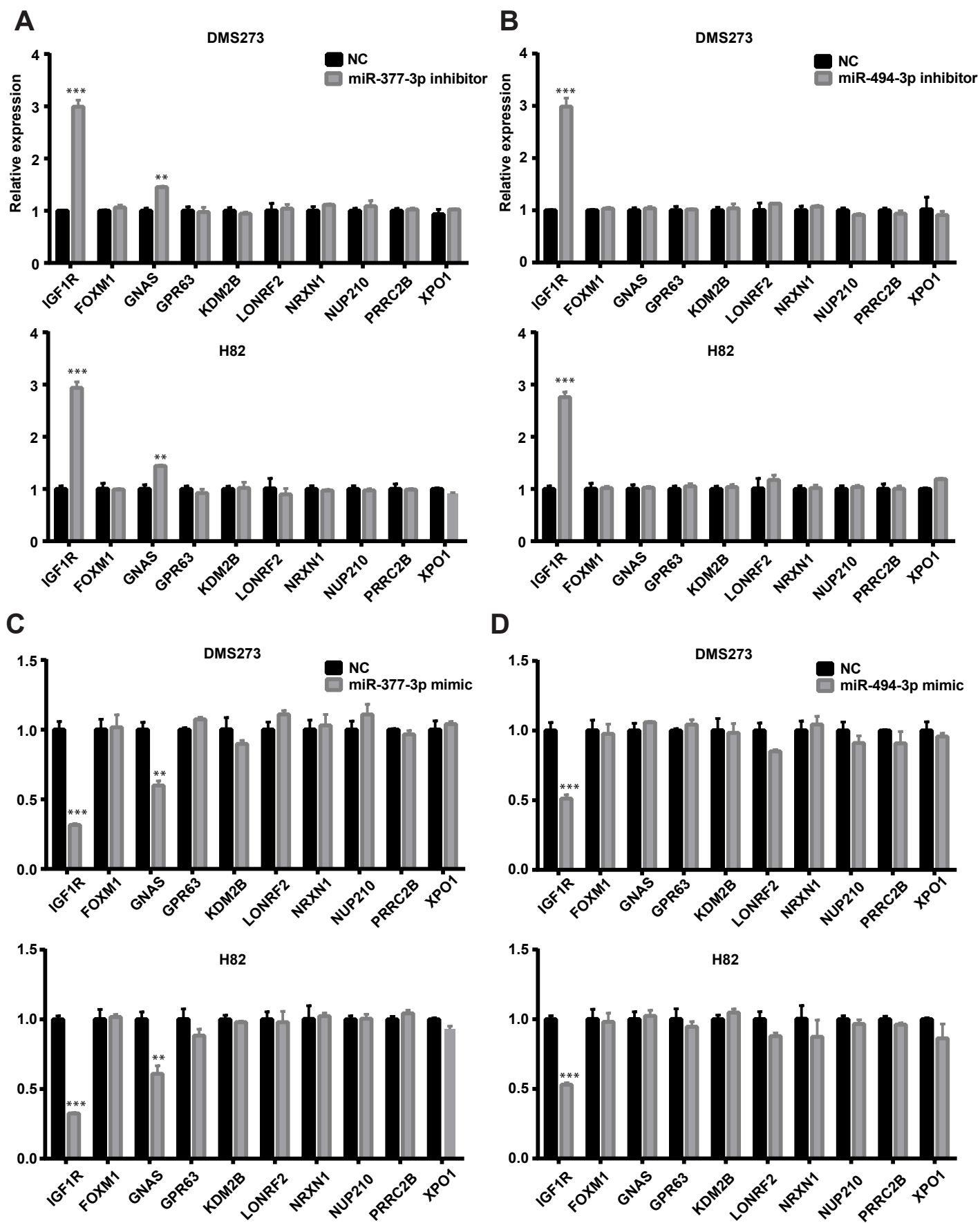

Figure S4.

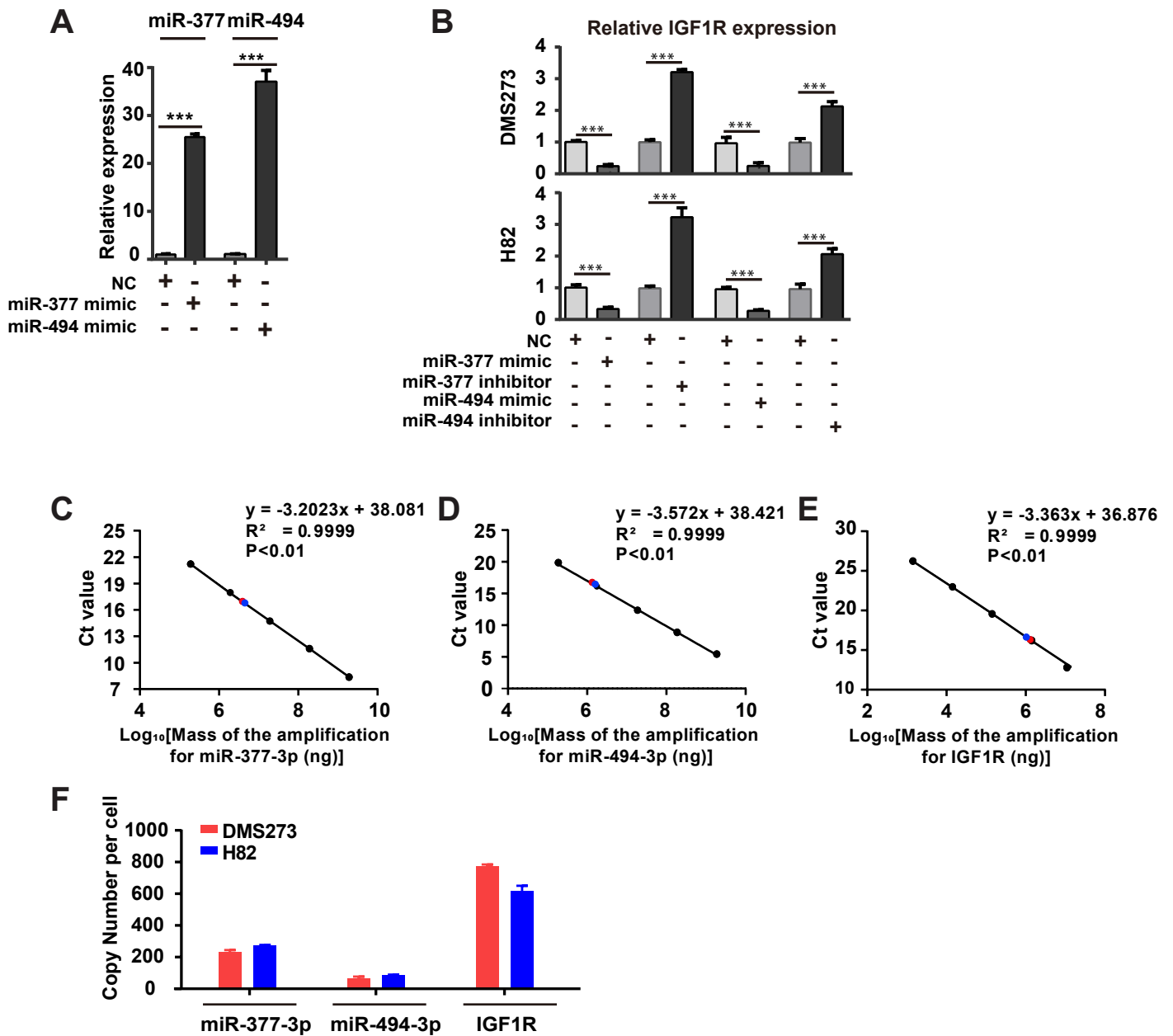

**Figure S5.**

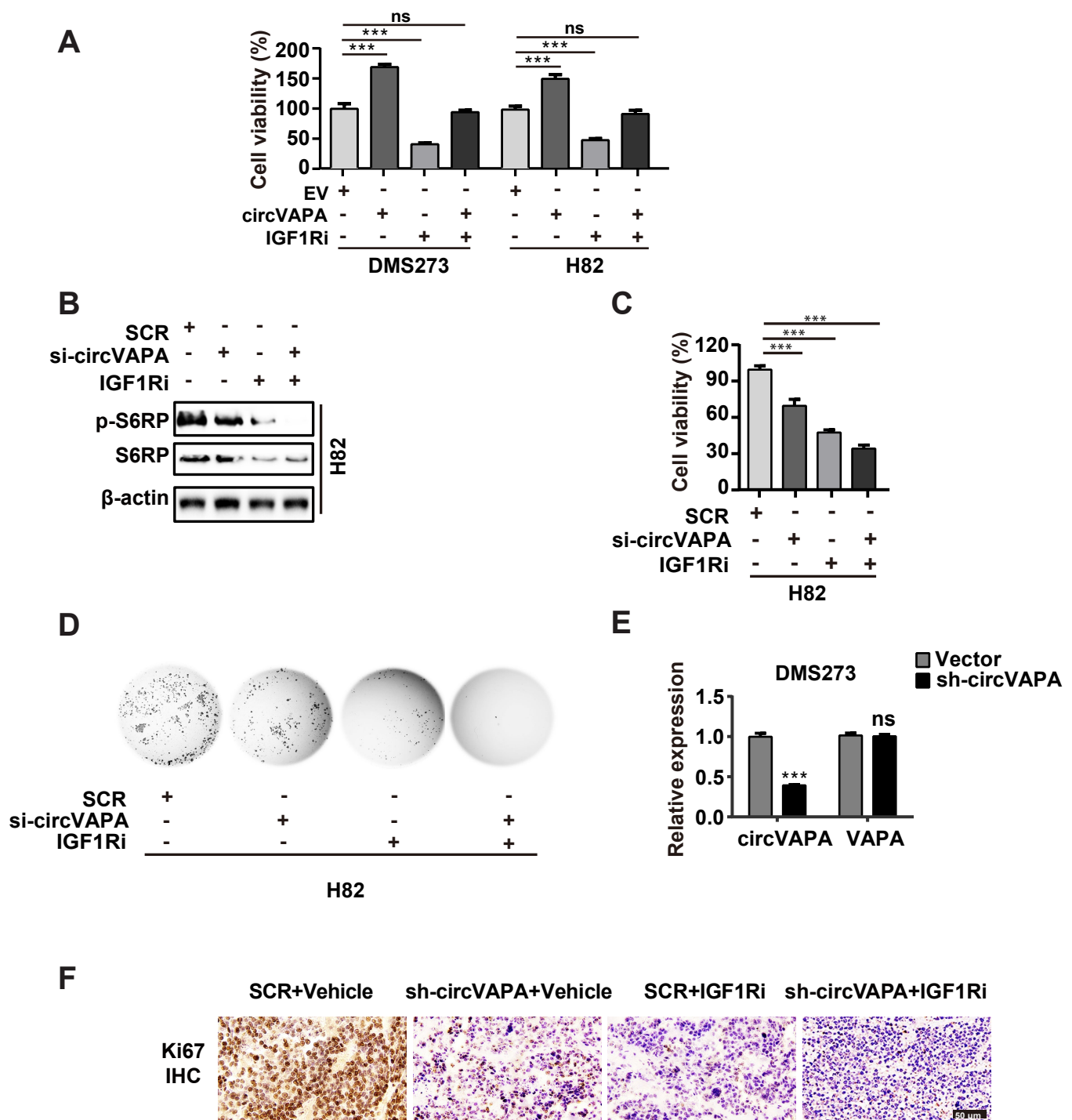

**Figure S6.**

Supplement: Supplementary file 1 — Additional file 1: Figure S1. (a) The relative expression of circVAPA, CircHIPK3 and 18S rRNA in SCLC cells were detected by RT-qPCR after RNase R treatment. CircHIPK3 was a positive control, while 18S rRNA was a negative control. (b-c) Quantification of circVAPA copy numbers in DMS273 and H82 cell lines. The red and blue dots indicate the Ct value and the amount of RNA in DMS273 and H82 SCLC cells, respectively. More experimental details are provided in the Methods section. (d) Prediction of the potential translation ability of circVAPA. An interaction model shows that circVAPA harbors potential internal ribosome entry site (IRES), but not open reading frame (ORF), which is analyzed by the circRNADb and ORFfinder databases. (All data are presented as the mean ± SD; ns, no significance; ***P < 0.001 by two-tailed Student’s t-test). Figure S2. (a) Schematic diagram of knocking down circVAPA using two independent siRNAs target circVAPA junction. (b-c) The apoptosis rate was analyzed by flow cytometry after downregulation (b) or overexpression (c) of circVAPA in SCLC cells. (All data are presented as the mean ± SD; ***P < 0.001 by two-tailed Student’s t-test). Three independent assays were performed in the above assays. Figure S3. (a) The effect of circVAPA on miR-377-3p and miR-494-3p expression levels in SCLC cells were detected by RT-qPCR. (b and c) RT-qPCR analysis of miR-377-3p (b) and miR-494-3p (c) expression in SCLC cells transiently transfected with the corresponding mimic, respectively. (d) After RNA pull-down by biotin-labeled circVAPA probe or negative probe, the miR-101 enrichment level in DMS273 cells was detected by RT-qPCR. (e) RT-qPCR analysis of miR-101 expression in SCLC cells transiently transfected with the corresponding mimic. (f) Western blot analysis of the effect of miR-101 on IGF1R, AKT, and its downstream protein expression in DMS273 (left) and H82 (right) SCLC cells. β-actin was used as an internal reference. (g-j) Cell viability analysis of [file 12943_2022_1595_MOESM1_ESM.pdf]
