# Supplementary material for: CircVAPA promotes small cell lung cancer progression by modulating the miR-377-3p and miR-494-3p/IGF1R/AKT axis
Source: Mol Cancer. 2022 Jun 6;21:123. doi: 10.1186/s12943-022-01595-9 (PMC9172052; doi:10.1186/s12943-022-01595-9)
Supplement: Supplementary file 2 — Additional file 2: Table S1. The differentially expressed circRNAs between serum samples from 36 SCLC patients and that from 118 healthy controls. [file 12943_2022_1595_MOESM2_ESM.docx]

**Table S1. The differentially expressed circRNAs between serum samples from 36 SCLC patients and that from 118 healthy controls.**

| **circRNA ID** | **circBase ID** | **Healthy** | **SCLC** | **Fold change** | **p value** |
| --- | --- | --- | --- | --- | --- |
| exo_circ_64043 | hsa_circ_0078510 | 0.032103 | 1.411917 | 43.98073562 | 4.6E-06 |
| exo_circ_00820 | hsa_circ_0020250 | 0.026993 | 1.036223 | 38.38786246 | 2.11E-05 |
| exo_circ_09376 | hsa_circ_0025725 | 0.023131 | 0.886223 | 38.31341257 | 0.000115 |
| exo_circ_22576 | hsa_circ_0004018 | 0.037448 | 1.429466 | 38.17201513 | 9.56E-07 |
| exo_circ_23863 | hsa_circ_0006379 | 0.025637 | 0.900457 | 35.12288022 | 8.1E-05 |
| exo_circ_10257 | hsa_circ_0026640 | 0.024999 | 0.84096 | 33.63942946 | 0.000137 |
| exo_circ_18884 | hsa_circ_0008636 | 0.027408 | 0.851981 | 31.084648 | 0.000482 |
| exo_circ_67994 | hsa_circ_0001773 | 0.023131 | 0.710295 | 30.70763351 | 0.000713 |
| exo_circ_05103 | hsa_circ_0021386 | 0.036081 | 1.044685 | 28.95401018 | 8.9E-05 |
| exo_circ_53172 | hsa_circ_0066474 | 0.040823 | 1.031672 | 25.27183313 | 0.000135 |
| exo_circ_59711 | hsa_circ_0002171 | 0.024485 | 0.618554 | 25.26214626 | 0.000772 |
| exo_circ_25815 | hsa_circ_0006413 | 0.024999 | 0.622452 | 24.89886991 | 0.001749 |
| exo_circ_76103 | hsa_circ_0086338 | 0.06424 | 1.562853 | 24.32849319 | 2.12E-06 |
| exo_circ_56913 | hsa_circ_0003201 | 0.03248 | 0.779174 | 23.98969799 | 0.000205 |
| exo_circ_44667 | hsa_circ_0058779 | 0.055863 | 1.283759 | 22.98056367 | 2.97E-05 |
| exo_circ_40598 | hsa_circ_0063716 | 0.074224 | 1.69991 | 22.9025151 | 9.31E-08 |
| exo_circ_21557 | hsa_circ_0039929 | 0.048214 | 1.103313 | 22.88380294 | 3.49E-05 |
| exo_circ_28538 | hsa_circ_0050512 | 0.04334 | 0.991699 | 22.88167292 | 5.62E-05 |
| exo_circ_74255 | hsa_circ_0088372 | 0.045005 | 1.009795 | 22.43736879 | 0.000163 |
| exo_circ_20840 | hsa_circ_0004947 | 0.028229 | 0.631978 | 22.38778621 | 0.000983 |
| exo_circ_07658 | hsa_circ_0028098 | 0.025637 | 0.566406 | 22.09299455 | 0.003837 |
| exo_circ_11374 | hsa_circ_0008405 | 0.02601 | 0.562158 | 21.61308618 | 0.003292 |
| exo_circ_22566 | hsa_circ_0003147 | 0.047382 | 1.021431 | 21.55745341 | 0.000177 |
| exo_circ_18671 | hsa_circ_0035875 | 0.029125 | 0.625383 | 21.47272719 | 0.000792 |
| exo_circ_40169 | hsa_circ_0062936 | 0.050247 | 1.053698 | 20.97042328 | 0.000227 |
| exo_circ_71911 | hsa_circ_0083766 | 0.054552 | 1.136465 | 20.83288272 | 2.08E-05 |
| exo_circ_59198 | hsa_circ_0074570 | 0.027907 | 0.580691 | 20.80776821 | 0.003697 |
| exo_circ_19055 | hsa_circ_0036174 | 0.027408 | 0.563564 | 20.56171267 | 0.004642 |
| exo_circ_29141 | hsa_circ_0007623 | 0.039798 | 0.791149 | 19.87935229 | 0.000952 |
| exo_circ_62046 | hsa_circ_0073360 | 0.031755 | 0.614566 | 19.35349315 | 0.003743 |
| exo_circ_30757 | hsa_circ_0014569 | 0.022571 | 0.433386 | 19.20125413 | 0.017308 |
| exo_circ_39619 | hsa_circ_0007969 | 0.03248 | 0.61585 | 18.96116193 | 0.006416 |
| exo_circ_74994 | hsa_circ_0086375 | 0.025883 | 0.476581 | 18.41314231 | 0.007121 |
| exo_circ_70368 | hsa_circ_0081314 | 0.060806 | 1.114249 | 18.32456111 | 0.0001 |
| exo_circ_07390 | hsa_circ_0004627 | 0.061705 | 1.127172 | 18.26702369 | 9.44E-05 |
| exo_circ_35831 | hsa_circ_0004478 | 0.06534 | 1.181128 | 18.07669598 | 0.000104 |
| exo_circ_05642 | hsa_circ_0020948 | 0.027907 | 0.502486 | 18.00546629 | 0.006209 |
| exo_circ_19742 | hsa_circ_0036799 | 0.040822 | 0.73439 | 17.99019175 | 0.000444 |
| exo_circ_74798 | hsa_circ_0089273 | 0.040478 | 0.724787 | 17.90584695 | 0.000541 |
| exo_circ_77979 | hsa_circ_0089952 | 0.03216 | 0.57226 | 17.79389569 | 0.001421 |
| exo_circ_52505 | hsa_circ_0003927 | 0.03604 | 0.640874 | 17.78236855 | 0.001387 |
| exo_circ_20478 | hsa_circ_0038349 | 0.031269 | 0.554558 | 17.73505289 | 0.00462 |
| exo_circ_25534 | hsa_circ_0045768 | 0.027907 | 0.490029 | 17.55908309 | 0.018772 |
| exo_circ_18316 | hsa_circ_0035474 | 0.069952 | 1.221389 | 17.46032158 | 7.95E-05 |
| exo_circ_32017 | hsa_circ_0015664 | 0.025109 | 0.435944 | 17.3617328 | 0.018722 |
| exo_circ_36318 | hsa_circ_0012975 | 0.024105 | 0.413022 | 17.13430315 | 0.018792 |
| exo_circ_27506 | hsa_circ_0004188 | 0.063925 | 1.085291 | 16.97744481 | 0.000333 |
| exo_circ_73826 | hsa_circ_0087906 | 0.031093 | 0.522798 | 16.81377377 | 0.005011 |
| exo_circ_74779 | hsa_circ_0089252 | 0.02601 | 0.436899 | 16.79732901 | 0.01921 |
| exo_circ_09579 | hsa_circ_0025825 | 0.044933 | 0.749459 | 16.6793347 | 0.001295 |
| exo_circ_15766 | hsa_circ_0032407 | 0.049423 | 0.818904 | 16.56939576 | 0.001211 |
| exo_circ_69072 | hsa_circ_0080242 | 0.048855 | 0.806738 | 16.51288461 | 0.000679 |
| exo_circ_37831 | hsa_circ_0060153 | 0.03484 | 0.572997 | 16.4465392 | 0.004698 |
| exo_circ_11935 | hsa_circ_0029642 | 0.039495 | 0.647988 | 16.40694436 | 0.004657 |
| exo_circ_38728 | hsa_circ_0061052 | 0.021601 | 0.351786 | 16.28553895 | 0.023212 |
| exo_circ_22129 | hsa_circ_0040773 | 0.038396 | 0.624774 | 16.27197482 | 0.004746 |
| exo_circ_14356 | hsa_circ_0031645 | 0.053325 | 0.866692 | 16.25308261 | 0.00018 |
| exo_circ_67558 | hsa_circ_0006013 | 0.073986 | 1.199356 | 16.21068781 | 4.93E-05 |
| exo_circ_23899 | hsa_circ_0008956 | 0.054881 | 0.884529 | 16.11708592 | 0.001414 |
| exo_circ_31126 | hsa_circ_0007398 | 0.026242 | 0.422829 | 16.11246926 | 0.020096 |
| exo_circ_76233 | hsa_circ_0087226 | 0.070117 | 1.123405 | 16.02176075 | 4.68E-05 |
| exo_circ_66542 | hsa_circ_0005058 | 0.048369 | 0.774305 | 16.00817618 | 0.001373 |
| exo_circ_58715 | hsa_circ_0074081 | 0.094239 | 1.494728 | 15.86109499 | 1.25E-05 |
| exo_circ_27588 | hsa_circ_0000862 | 0.039864 | 0.620188 | 15.55766239 | 0.005089 |
| exo_circ_28691 | hsa_circ_0002427 | 0.026501 | 0.411044 | 15.5104082 | 0.021487 |
| exo_circ_64481 | hsa_circ_0075854 | 0.034256 | 0.529784 | 15.46545601 | 0.005414 |
| exo_circ_39476 | hsa_circ_0061776 | 0.117048 | 1.803213 | 15.40570322 | 1.24E-06 |
| exo_circ_73011 | hsa_circ_0003399 | 0.037021 | 0.569178 | 15.37466738 | 0.005419 |
| exo_circ_15758 | hsa_circ_0032384 | 0.042064 | 0.644459 | 15.32102164 | 0.002036 |
| exo_circ_25814 | hsa_circ_0046447 | 0.02601 | 0.398012 | 15.30223916 | 0.020793 |
| exo_circ_61404 | hsa_circ_0001497 | 0.037328 | 0.570989 | 15.29655159 | 0.005895 |
| exo_circ_08897 | hsa_circ_0029592 | 0.028845 | 0.440115 | 15.25808686 | 0.021771 |
| exo_circ_12846 | hsa_circ_0030222 | 0.040187 | 0.612352 | 15.23758512 | 0.005413 |
| exo_circ_78691 | hsa_circ_0091013 | 0.031873 | 0.484621 | 15.20452651 | 0.009512 |
| exo_circ_56256 | hsa_circ_0069561 | 0.052171 | 0.79008 | 15.14395893 | 0.000614 |
| exo_circ_12201 | hsa_circ_0029834 | 0.056034 | 0.845783 | 15.09402621 | 0.001756 |
| exo_circ_74050 | hsa_circ_0088135 | 0.02487 | 0.372763 | 14.98824095 | 0.022843 |
| exo_circ_25848 | hsa_circ_0046573 | 0.031203 | 0.466199 | 14.94060919 | 0.005865 |
| exo_circ_58615 | hsa_circ_0074009 | 0.078882 | 1.173323 | 14.87438278 | 0.000211 |
| exo_circ_20099 | hsa_circ_0009128 | 0.096365 | 1.42801 | 14.81869681 | 5.83E-06 |
| exo_circ_58080 | hsa_circ_0009054 | 0.028802 | 0.425437 | 14.77113052 | 0.021505 |
| exo_circ_25447 | hsa_circ_0045534 | 0.055646 | 0.816679 | 14.67644315 | 0.001708 |
| exo_circ_34792 | hsa_circ_0011547 | 0.045326 | 0.664986 | 14.67124315 | 0.005687 |
| exo_circ_20494 | hsa_circ_0038367 | 0.039283 | 0.572455 | 14.57254686 | 0.006359 |
| exo_circ_29419 | hsa_circ_0000885 | 0.046544 | 0.673794 | 14.4766274 | 0.002091 |
| exo_circ_43894 | hsa_circ_0002141 | 0.178921 | 2.585671 | 14.45148768 | 2.54E-11 |
| exo_circ_74282 | hsa_circ_0088398 | 0.024947 | 0.359726 | 14.41968199 | 0.022406 |
| exo_circ_40882 | hsa_circ_0052615 | 0.03721 | 0.533384 | 14.33456393 | 0.006548 |
| exo_circ_70437 | hsa_circ_0085112 | 0.062732 | 0.899085 | 14.33222237 | 0.000683 |
| exo_circ_64816 | hsa_circ_0076172 | 0.034569 | 0.495382 | 14.33030065 | 0.022181 |
| exo_circ_63865 | hsa_circ_0078363 | 0.046894 | 0.66859 | 14.2575236 | 0.00628 |
| exo_circ_72494 | hsa_circ_0084431 | 0.033068 | 0.471361 | 14.25412154 | 0.023007 |
| exo_circ_48077 | hsa_circ_0066675 | 0.023246 | 0.329143 | 14.15881672 | 0.027788 |
| exo_circ_67427 | hsa_circ_0001755 | 0.052211 | 0.737136 | 14.11854058 | 0.001053 |
| exo_circ_31850 | hsa_circ_0006924 | 0.073224 | 1.030408 | 14.07201463 | 0.000304 |
| exo_circ_17664 | hsa_circ_0035086 | 0.147242 | 2.071725 | 14.07021092 | 4.55E-07 |
| exo_circ_66268 | hsa_circ_0007821 | 0.03721 | 0.521201 | 14.00715732 | 0.006673 |
| exo_circ_62323 | hsa_circ_0077496 | 0.061492 | 0.859632 | 13.97955436 | 0.002177 |
| exo_circ_15994 | hsa_circ_0000555 | 0.041127 | 0.56992 | 13.85767117 | 0.007309 |
| exo_circ_29101 | hsa_circ_0004720 | 0.081432 | 1.124288 | 13.806495 | 0.000148 |
| exo_circ_06914 | hsa_circ_0023901 | 0.031188 | 0.430451 | 13.80170206 | 0.023552 |
| exo_circ_25669 | hsa_circ_0005294 | 0.110568 | 1.524505 | 13.78789723 | 3.14E-05 |
| exo_circ_06926 | hsa_circ_0002264 | 0.132672 | 1.825154 | 13.75693991 | 9.01E-07 |
| exo_circ_37363 | hsa_circ_0059187 | 0.047136 | 0.648081 | 13.74922645 | 0.006497 |
| exo_circ_21455 | hsa_circ_0005943 | 0.044664 | 0.612988 | 13.72445918 | 0.00252 |
| exo_circ_13747 | hsa_circ_0033424 | 0.033974 | 0.464664 | 13.67684521 | 0.00683 |
| exo_circ_54512 | hsa_circ_0004731 | 0.080286 | 1.08578 | 13.52385362 | 0.000855 |
| exo_circ_39117 | hsa_circ_0061491 | 0.07068 | 0.954744 | 13.50790984 | 0.000804 |
| exo_circ_78596 | hsa_circ_0090879 | 0.058348 | 0.785216 | 13.45746716 | 0.002075 |
| exo_circ_23805 | hsa_circ_0004402 | 0.030917 | 0.413148 | 13.36302334 | 0.024606 |
| exo_circ_69012 | hsa_circ_0080210 | 0.036682 | 0.490168 | 13.36261791 | 0.008452 |
| exo_circ_39597 | hsa_circ_0007126 | 0.039854 | 0.530367 | 13.30759408 | 0.009388 |
| exo_circ_29489 | hsa_circ_0008990 | 0.039398 | 0.523917 | 13.29817562 | 0.008195 |
| exo_circ_06314 | hsa_circ_0006277 | 0.061246 | 0.812508 | 13.2663803 | 0.002236 |
| exo_circ_17287 | hsa_circ_0008926 | 0.095213 | 1.262495 | 13.25970042 | 9.81E-05 |
| exo_circ_23428 | hsa_circ_0043136 | 0.048596 | 0.643541 | 13.24279827 | 0.006791 |
| exo_circ_62633 | hsa_circ_0077657 | 0.069006 | 0.908814 | 13.1700711 | 0.001027 |
| exo_circ_40216 | hsa_circ_0002063 | 0.039116 | 0.512485 | 13.10178491 | 0.010488 |
| exo_circ_67402 | hsa_circ_0082654 | 0.077685 | 1.015979 | 13.07817632 | 0.000426 |
| exo_circ_43315 | hsa_circ_0057611 | 0.051051 | 0.665888 | 13.04350941 | 0.007065 |
| exo_circ_02873 | hsa_circ_0018544 | 0.034777 | 0.453334 | 13.03551181 | 0.009261 |
| exo_circ_13077 | hsa_circ_0030345 | 0.024999 | 0.322838 | 12.91390754 | 0.024846 |
| exo_circ_01025 | hsa_circ_0020457 | 0.056389 | 0.726571 | 12.88504531 | 0.00262 |
| exo_circ_15048 | hsa_circ_0002012 | 0.065308 | 0.838893 | 12.84519683 | 0.002346 |
| exo_circ_22023 | hsa_circ_0003857 | 0.047382 | 0.60825 | 12.83720795 | 0.007233 |
| exo_circ_39565 | hsa_circ_0061938 | 0.057247 | 0.732076 | 12.78812762 | 0.00273 |
| exo_circ_55719 | hsa_circ_0002785 | 0.03246 | 0.41213 | 12.69663053 | 0.027304 |
| exo_circ_04584 | hsa_circ_0024466 | 0.028845 | 0.364317 | 12.63027262 | 0.03216 |
| exo_circ_48349 | hsa_circ_0066825 | 0.032106 | 0.403671 | 12.57317632 | 0.025655 |
| exo_circ_68002 | hsa_circ_0004755 | 0.051051 | 0.64031 | 12.54248973 | 0.007546 |
| exo_circ_32206 | hsa_circ_0006337 | 0.047183 | 0.591753 | 12.54170479 | 0.001209 |
| exo_circ_47891 | hsa_circ_0055798 | 0.07137 | 0.888717 | 12.4522637 | 0.002872 |
| exo_circ_57821 | hsa_circ_0073497 | 0.082019 | 1.018776 | 12.42120209 | 0.000186 |
| exo_circ_70400 | hsa_circ_0007940 | 0.070342 | 0.87282 | 12.40830434 | 0.000746 |
| exo_circ_66078 | hsa_circ_0077291 | 0.048768 | 0.604536 | 12.39617869 | 0.008284 |
| exo_circ_50452 | hsa_circ_0002726 | 0.05994 | 0.742945 | 12.39473398 | 0.001546 |
| exo_circ_41510 | hsa_circ_0056488 | 0.062047 | 0.768505 | 12.38582494 | 0.00087 |
| exo_circ_45730 | hsa_circ_0002348 | 0.059435 | 0.735087 | 12.36793098 | 0.003843 |
| exo_circ_34312 | hsa_circ_0010927 | 0.064264 | 0.793353 | 12.3452959 | 0.001279 |
| exo_circ_27167 | hsa_circ_0047807 | 0.034653 | 0.427435 | 12.33466315 | 0.026268 |
| exo_circ_19806 | hsa_circ_0036950 | 0.05331 | 0.657405 | 12.33164504 | 0.007792 |
| exo_circ_78195 | hsa_circ_0090135 | 0.058467 | 0.716889 | 12.26152308 | 0.002795 |
| exo_circ_14883 | hsa_circ_0031856 | 0.059092 | 0.72394 | 12.25112335 | 0.004205 |
| exo_circ_29084 | hsa_circ_0006699 | 0.073061 | 0.891765 | 12.20573382 | 0.000257 |
| exo_circ_60156 | hsa_circ_0002292 | 0.064381 | 0.783715 | 12.1731323 | 0.00171 |
| exo_circ_72202 | hsa_circ_0084026 | 0.031188 | 0.378494 | 12.13579077 | 0.029446 |
| exo_circ_59886 | hsa_circ_0075291 | 0.031171 | 0.378177 | 12.13232588 | 0.034535 |
| exo_circ_03506 | hsa_circ_0002281 | 0.031269 | 0.378834 | 12.11529139 | 0.027011 |
| exo_circ_38738 | hsa_circ_0061132 | 0.102178 | 1.234869 | 12.08548398 | 3.34E-05 |
| exo_circ_54878 | hsa_circ_0071113 | 0.068942 | 0.833068 | 12.08357146 | 0.003052 |
| exo_circ_17538 | hsa_circ_0034970 | 0.159731 | 1.920876 | 12.02569899 | 4.8E-07 |
| exo_circ_45308 | hsa_circ_0006348 | 0.083235 | 0.998345 | 11.99425631 | 7.66E-05 |
| exo_circ_05963 | hsa_circ_0002904 | 0.061487 | 0.736453 | 11.97746605 | 0.004282 |
| exo_circ_57003 | hsa_circ_0008578 | 0.028845 | 0.345003 | 11.96069027 | 0.027779 |
| exo_circ_40444 | hsa_circ_0009079 | 0.148166 | 1.76717 | 11.92692079 | 7.55E-06 |
| exo_circ_36394 | hsa_circ_0000088 | 0.051621 | 0.614587 | 11.90579166 | 0.005546 |
| exo_circ_19196 | hsa_circ_0005470 | 0.104539 | 1.242927 | 11.88954709 | 4.82E-05 |
| exo_circ_41301 | hsa_circ_0056258 | 0.036267 | 0.429165 | 11.83357628 | 0.028875 |
| exo_circ_38374 | hsa_circ_0060665 | 0.040126 | 0.471613 | 11.75315507 | 0.011518 |
| exo_circ_30990 | hsa_circ_0010064 | 0.054476 | 0.638273 | 11.71663747 | 0.008839 |
| exo_circ_29098 | hsa_circ_0048687 | 0.04013 | 0.468145 | 11.66567522 | 0.010116 |
| exo_circ_48266 | hsa_circ_0066803 | 0.037599 | 0.438345 | 11.65839792 | 0.02842 |
| exo_circ_00643 | hsa_circ_0017701 | 0.047863 | 0.556492 | 11.62677182 | 0.00913 |
| exo_circ_75552 | hsa_circ_0006728 | 0.059913 | 0.691141 | 11.53582317 | 0.001547 |
| exo_circ_69336 | hsa_circ_0005610 | 0.034643 | 0.398584 | 11.50542813 | 0.02982 |
| exo_circ_06023 | hsa_circ_0002489 | 0.11531 | 1.319513 | 11.44319145 | 2.55E-05 |
| exo_circ_35677 | hsa_circ_0006426 | 0.055323 | 0.632543 | 11.43368416 | 0.009691 |
| exo_circ_22147 | hsa_circ_0005483 | 0.072044 | 0.822429 | 11.41571847 | 0.001126 |
| exo_circ_78974 | hsa_circ_0092238 | 0.031449 | 0.358926 | 11.4130667 | 0.029038 |
| exo_circ_37980 | hsa_circ_0007673 | 0.055764 | 0.634351 | 11.37564394 | 0.004346 |
| exo_circ_03773 | hsa_circ_0019201 | 0.071898 | 0.8149 | 11.33409449 | 0.00356 |
| exo_circ_14152 | hsa_circ_0031482 | 0.124083 | 1.404481 | 11.31887897 | 3.17E-05 |
| exo_circ_30792 | hsa_circ_0008161 | 0.063603 | 0.719071 | 11.30552781 | 0.005532 |
| exo_circ_30527 | hsa_circ_0005995 | 0.045942 | 0.51689 | 11.25086003 | 0.009965 |
| exo_circ_35283 | hsa_circ_0005361 | 0.055646 | 0.624378 | 11.22062272 | 0.005758 |
| exo_circ_38407 | hsa_circ_0060733 | 0.033734 | 0.378503 | 11.22033543 | 0.029797 |
| exo_circ_74751 | hsa_circ_0006074 | 0.035741 | 0.400977 | 11.2189004 | 0.029648 |
| exo_circ_33840 | hsa_circ_0004595 | 0.065081 | 0.728778 | 11.19807667 | 0.003731 |
| exo_circ_60324 | hsa_circ_0072339 | 0.034758 | 0.388831 | 11.18693785 | 0.029893 |
| exo_circ_23719 | hsa_circ_0043523 | 0.063282 | 0.707113 | 11.17401423 | 0.004054 |
| exo_circ_39071 | hsa_circ_0061405 | 0.048768 | 0.544201 | 11.15897929 | 0.011545 |
| exo_circ_59898 | hsa_circ_0005778 | 0.072858 | 0.812475 | 11.15153786 | 0.001888 |
| exo_circ_72482 | hsa_circ_0084327 | 0.058607 | 0.653242 | 11.14616351 | 0.00639 |
| exo_circ_55871 | hsa_circ_0001399 | 0.046544 | 0.518478 | 11.13961676 | 0.030591 |
| exo_circ_48829 | hsa_circ_0067204 | 0.071892 | 0.8 | 11.12776526 | 0.00382 |
| exo_circ_25618 | hsa_circ_0045864 | 0.108293 | 1.201231 | 11.09245615 | 0.00024 |
| exo_circ_28939 | hsa_circ_0002105 | 0.039942 | 0.441855 | 11.06245167 | 0.004412 |
| exo_circ_78442 | hsa_circ_0090637 | 0.04289 | 0.473129 | 11.03117626 | 0.012467 |
| exo_circ_16180 | hsa_circ_0032826 | 0.054219 | 0.598036 | 11.03007147 | 0.010891 |
| exo_circ_69193 | hsa_circ_0079335 | 0.074052 | 0.816089 | 11.02047083 | 0.003973 |
| exo_circ_73449 | hsa_circ_0084964 | 0.03113 | 0.342271 | 10.99493435 | 0.043043 |
| exo_circ_20503 | hsa_circ_0038375 | 0.095163 | 1.045683 | 10.98833441 | 0.000756 |
| exo_circ_74007 | hsa_circ_0088080 | 0.031401 | 0.345032 | 10.98792819 | 0.049994 |
| exo_circ_07364 | hsa_circ_0027862 | 0.058058 | 0.637657 | 10.98319632 | 0.010239 |
| exo_circ_05376 | hsa_circ_0003712 | 0.074616 | 0.819374 | 10.98127425 | 0.004118 |
| exo_circ_15414 | hsa_circ_0032125 | 0.13207 | 1.441193 | 10.91238144 | 3.04E-05 |
| exo_circ_30011 | hsa_circ_0013573 | 0.05415 | 0.589913 | 10.89414239 | 0.010575 |
| exo_circ_72882 | hsa_circ_0084677 | 0.027325 | 0.29743 | 10.88489141 | 0.034176 |
| exo_circ_66437 | hsa_circ_0081808 | 0.058744 | 0.638741 | 10.87333456 | 0.011267 |
| exo_circ_67646 | hsa_circ_0082822 | 0.096056 | 1.043383 | 10.86220512 | 0.001953 |
| exo_circ_67757 | hsa_circ_0082951 | 0.032171 | 0.348717 | 10.83947795 | 0.03343 |
| exo_circ_18881 | hsa_circ_0003973 | 0.06437 | 0.697287 | 10.83246062 | 0.002771 |
| exo_circ_70440 | hsa_circ_0085114 | 0.057071 | 0.617424 | 10.81846536 | 0.010844 |
| exo_circ_52714 | hsa_circ_0064086 | 0.033622 | 0.362876 | 10.79292327 | 0.03559 |
| exo_circ_22317 | hsa_circ_0037127 | 0.052289 | 0.564267 | 10.79127403 | 0.012941 |
| exo_circ_53949 | hsa_circ_0070655 | 0.031286 | 0.337117 | 10.77528868 | 0.047293 |
| exo_circ_78964 | hsa_circ_0009019 | 0.113067 | 1.218024 | 10.77263244 | 0.000118 |
| exo_circ_19066 | hsa_circ_0004249 | 0.066794 | 0.717715 | 10.74515912 | 0.004503 |
| exo_circ_28278 | hsa_circ_0002596 | 0.059916 | 0.641986 | 10.71476693 | 0.005333 |
| exo_circ_20564 | hsa_circ_0038439 | 0.04013 | 0.429063 | 10.69178425 | 0.011399 |
| exo_circ_23780 | hsa_circ_0043692 | 0.033915 | 0.361926 | 10.67152757 | 0.015703 |
| exo_circ_30278 | hsa_circ_0009972 | 0.031401 | 0.334859 | 10.66395653 | 0.032959 |
| exo_circ_31596 | hsa_circ_0015315 | 0.034777 | 0.37045 | 10.65221043 | 0.032322 |
| exo_circ_64176 | hsa_circ_0004032 | 0.05957 | 0.63433 | 10.64855816 | 0.002911 |
| exo_circ_72966 | hsa_circ_0084714 | 0.081087 | 0.861902 | 10.62936299 | 0.002424 |
| exo_circ_49117 | hsa_circ_0067478 | 0.149313 | 1.586646 | 10.62632595 | 5.5E-05 |
| exo_circ_07657 | hsa_circ_0028097 | 0.03216 | 0.341564 | 10.6206019 | 0.035687 |
| exo_circ_30692 | hsa_circ_0014394 | 0.036765 | 0.390157 | 10.61215876 | 0.015053 |
| exo_circ_09601 | hsa_circ_0025836 | 0.041245 | 0.437651 | 10.61111384 | 0.012106 |
| exo_circ_74671 | hsa_circ_0089075 | 0.033377 | 0.353685 | 10.59670727 | 0.012292 |
| exo_circ_78559 | hsa_circ_0090868 | 0.060477 | 0.640638 | 10.59312069 | 0.011047 |
| exo_circ_29619 | hsa_circ_0009685 | 0.035741 | 0.378343 | 10.58563333 | 0.035467 |
| exo_circ_48728 | hsa_circ_0006617 | 0.031171 | 0.32991 | 10.58384953 | 0.03417 |
| exo_circ_30889 | hsa_circ_0014632 | 0.063287 | 0.669462 | 10.57816248 | 0.011101 |
| exo_circ_37648 | hsa_circ_0059951 | 0.043681 | 0.461601 | 10.56759996 | 0.014176 |
| exo_circ_13233 | hsa_circ_0005783 | 0.173651 | 1.834447 | 10.56399689 | 8.81E-06 |
| exo_circ_22236 | hsa_circ_0004203 | 0.023131 | 0.243763 | 10.53841383 | 0.034322 |
| exo_circ_69249 | hsa_circ_0003847 | 0.047416 | 0.498939 | 10.5224801 | 0.013053 |
| exo_circ_74804 | hsa_circ_0089280 | 0.058437 | 0.614011 | 10.5072643 | 0.01152 |
| exo_circ_25018 | hsa_circ_0045202 | 0.063509 | 0.667161 | 10.50497164 | 0.003761 |
| exo_circ_38146 | hsa_circ_0006167 | 0.041127 | 0.431731 | 10.49759562 | 0.032751 |
| exo_circ_23865 | hsa_circ_0006823 | 0.064614 | 0.677795 | 10.4899593 | 0.00544 |
| exo_circ_29841 | hsa_circ_0013431 | 0.04334 | 0.454457 | 10.4857874 | 0.033492 |
| exo_circ_08428 | hsa_circ_0005305 | 0.047509 | 0.49793 | 10.48064325 | 0.011749 |
| exo_circ_23399 | hsa_circ_0043108 | 0.131117 | 1.373845 | 10.47802813 | 0.000119 |
| exo_circ_10353 | hsa_circ_0004843 | 0.033467 | 0.350468 | 10.47188859 | 0.034299 |
| exo_circ_39458 | hsa_circ_0061748 | 0.032656 | 0.341773 | 10.46587478 | 0.035199 |
| exo_circ_56086 | hsa_circ_0069444 | 0.055114 | 0.576155 | 10.45380757 | 0.011648 |
| exo_circ_07912 | hsa_circ_0003197 | 0.120481 | 1.257409 | 10.43655841 | 0.000189 |
| exo_circ_37546 | hsa_circ_0059858 | 0.06496 | 0.677907 | 10.43583191 | 0.002529 |
| exo_circ_00837 | hsa_circ_0020254 | 0.08124 | 0.846632 | 10.42133424 | 0.003018 |
| exo_circ_00141 | hsa_circ_0019658 | 0.087672 | 0.913604 | 10.42065037 | 0.001184 |
| exo_circ_19252 | hsa_circ_0036405 | 0.08333 | 0.867417 | 10.40942871 | 0.00359 |
| exo_circ_50387 | hsa_circ_0002422 | 0.065884 | 0.681349 | 10.34160487 | 0.006071 |
| exo_circ_34323 | hsa_circ_0010937 | 0.080409 | 0.830037 | 10.32272845 | 0.00491 |
| exo_circ_64033 | hsa_circ_0078486 | 0.068747 | 0.707737 | 10.29479307 | 0.011849 |
| exo_circ_01904 | hsa_circ_0018111 | 0.032774 | 0.336607 | 10.27063666 | 0.034626 |
| exo_circ_23582 | hsa_circ_0043318 | 0.052254 | 0.535466 | 10.2473282 | 0.012406 |
| exo_circ_69415 | hsa_circ_0079400 | 0.133708 | 1.368424 | 10.23442749 | 8.11E-05 |
| exo_circ_35293 | hsa_circ_0012270 | 0.075463 | 0.77022 | 10.20656697 | 0.002669 |
| exo_circ_25842 | hsa_circ_0046557 | 0.056201 | 0.572717 | 10.19053211 | 0.012842 |
| exo_circ_27284 | hsa_circ_0046759 | 0.056063 | 0.569877 | 10.16500559 | 0.006242 |
| exo_circ_37612 | hsa_circ_0059935 | 0.080398 | 0.816987 | 10.16180555 | 0.005733 |
| exo_circ_59273 | hsa_circ_0007069 | 0.036493 | 0.370708 | 10.15828902 | 0.036657 |
| exo_circ_06705 | hsa_circ_0023684 | 0.072015 | 0.731048 | 10.15137095 | 0.005464 |
| exo_circ_57486 | hsa_circ_0008412 | 0.061271 | 0.621691 | 10.14656088 | 0.013658 |
| exo_circ_37553 | hsa_circ_0008532 | 0.112667 | 1.139572 | 10.11448395 | 0.000326 |
| exo_circ_27625 | hsa_circ_0004428 | 0.06352 | 0.641679 | 10.10199029 | 0.007431 |
| exo_circ_39348 | hsa_circ_0006075 | 0.149101 | 1.504124 | 10.08794909 | 2.13E-05 |
| exo_circ_34724 | hsa_circ_0004013 | 0.075243 | 0.757904 | 10.07269389 | 0.006198 |
| exo_circ_73305 | hsa_circ_0004394 | 0.07287 | 0.733447 | 10.06512713 | 0.006515 |
| exo_circ_03806 | hsa_circ_0019223 | 0.058176 | 0.58225 | 10.0084346 | 0.014337 |
| exo_circ_21887 | hsa_circ_0007511 | 0.060591 | 0.6063 | 10.00638729 | 0.01363 |
| exo_circ_46426 | hsa_circ_0004772 | 0.039078 | 0.390989 | 10.00540991 | 0.037964 |
| exo_circ_76228 | hsa_circ_0087223 | 0.102183 | 1.017454 | 9.957136267 | 0.000994 |
| exo_circ_35465 | hsa_circ_0008090 | 0.03721 | 0.370202 | 9.949088487 | 0.036443 |
| exo_circ_18384 | hsa_circ_0005402 | 0.050649 | 0.503408 | 9.939203144 | 0.017594 |
| exo_circ_75421 | hsa_circ_0086603 | 0.066174 | 0.657273 | 9.932472409 | 0.00625 |
| exo_circ_10247 | hsa_circ_0026622 | 0.074717 | 0.741786 | 9.927985552 | 0.005778 |
| exo_circ_33750 | hsa_circ_0006021 | 0.051879 | 0.514112 | 9.909759565 | 0.013818 |
| exo_circ_15821 | hsa_circ_0032471 | 0.105546 | 1.042466 | 9.876891426 | 0.000494 |
| exo_circ_57613 | hsa_circ_0001426 | 0.115017 | 1.127944 | 9.806754944 | 0.000469 |
| exo_circ_30276 | hsa_circ_0007677 | 0.119963 | 1.176381 | 9.806230675 | 0.000409 |
| exo_circ_63707 | hsa_circ_0078264 | 0.060401 | 0.591016 | 9.784877265 | 0.013719 |
| exo_circ_35115 | hsa_circ_0005303 | 0.136043 | 1.330877 | 9.782740548 | 0.000182 |
| exo_circ_53425 | hsa_circ_0002453 | 0.038894 | 0.380085 | 9.772267952 | 0.01361 |
| exo_circ_49326 | hsa_circ_0002516 | 0.041446 | 0.40293 | 9.721874301 | 0.020947 |
| exo_circ_28292 | hsa_circ_0050286 | 0.027907 | 0.271142 | 9.715756018 | 0.045908 |
| exo_circ_18486 | hsa_circ_0035653 | 0.070782 | 0.679746 | 9.603353551 | 0.006811 |
| exo_circ_34693 | hsa_circ_0011422 | 0.060785 | 0.582242 | 9.578666136 | 0.008719 |
| exo_circ_01156 | hsa_circ_0004183 | 0.041367 | 0.396024 | 9.573390448 | 0.018137 |
| exo_circ_75562 | hsa_circ_0003624 | 0.069006 | 0.660223 | 9.567624461 | 0.005101 |
| exo_circ_34393 | hsa_circ_0011075 | 0.04636 | 0.442682 | 9.548796432 | 0.038478 |
| exo_circ_74692 | hsa_circ_0002543 | 0.03877 | 0.369152 | 9.521653238 | 0.03875 |
| exo_circ_70218 | hsa_circ_0081009 | 0.085187 | 0.808671 | 9.492944295 | 0.006569 |
| exo_circ_74365 | hsa_circ_0088446 | 0.061844 | 0.586258 | 9.479604544 | 0.017678 |
| exo_circ_75167 | hsa_circ_0086445 | 0.08417 | 0.796306 | 9.460670321 | 0.003173 |
| exo_circ_39435 | hsa_circ_0061739 | 0.035247 | 0.332694 | 9.438920122 | 0.040161 |
| exo_circ_23389 | hsa_circ_0043082 | 0.088408 | 0.834158 | 9.435318535 | 0.000767 |
| exo_circ_07523 | hsa_circ_0027966 | 0.045005 | 0.424153 | 9.424570743 | 0.038339 |
| exo_circ_10092 | hsa_circ_0026230 | 0.080782 | 0.761074 | 9.421278279 | 0.006788 |
| exo_circ_47921 | hsa_circ_0004147 | 0.03983 | 0.37495 | 9.413879101 | 0.038704 |
| exo_circ_70387 | hsa_circ_0002974 | 0.074614 | 0.70233 | 9.412800253 | 0.00416 |
| exo_circ_19881 | hsa_circ_0036990 | 0.048808 | 0.458757 | 9.399217533 | 0.016507 |
| exo_circ_73616 | hsa_circ_0085082 | 0.040263 | 0.37815 | 9.39189937 | 0.039397 |
| exo_circ_28980 | hsa_circ_0002444 | 0.051672 | 0.484648 | 9.379372896 | 0.016871 |
| exo_circ_39792 | hsa_circ_0062559 | 0.096458 | 0.903116 | 9.362789448 | 0.001463 |
| exo_circ_66841 | hsa_circ_0003773 | 0.12563 | 1.173846 | 9.34365057 | 0.000504 |
| exo_circ_65167 | hsa_circ_0003563 | 0.049766 | 0.464504 | 9.333772306 | 0.039137 |
| exo_circ_03061 | hsa_circ_0017627 | 0.063354 | 0.591282 | 9.332929023 | 0.016276 |
| exo_circ_39692 | hsa_circ_0062239 | 0.062769 | 0.585078 | 9.32106861 | 0.016075 |
| exo_circ_47249 | hsa_circ_0008220 | 0.065125 | 0.607013 | 9.320742099 | 0.007958 |
| exo_circ_65716 | hsa_circ_0077101 | 0.090157 | 0.838751 | 9.303230795 | 0.007374 |
| exo_circ_59955 | hsa_circ_0075364 | 0.072095 | 0.66997 | 9.292819314 | 0.016133 |
| exo_circ_11672 | hsa_circ_0030885 | 0.069221 | 0.641041 | 9.260789987 | 0.015576 |
| exo_circ_32929 | hsa_circ_0000183 | 0.059831 | 0.552114 | 9.227967672 | 0.018668 |
| exo_circ_12075 | hsa_circ_0003040 | 0.081152 | 0.748449 | 9.22280824 | 0.007632 |
| exo_circ_68903 | hsa_circ_0005369 | 0.139453 | 1.285342 | 9.217009435 | 0.000375 |
| exo_circ_27076 | hsa_circ_0047744 | 0.1206 | 1.108125 | 9.188459544 | 0.000196 |
| exo_circ_30575 | hsa_circ_0008018 | 0.073721 | 0.676996 | 9.183233726 | 0.004022 |
| exo_circ_17558 | hsa_circ_0034993 | 0.115193 | 1.057677 | 9.181745966 | 0.001405 |
| exo_circ_74037 | hsa_circ_0088111 | 0.040478 | 0.371506 | 9.178047243 | 0.040025 |
| exo_circ_21892 | hsa_circ_0007963 | 0.133213 | 1.216182 | 9.129613012 | 0.000564 |
| exo_circ_26157 | hsa_circ_0047063 | 0.091375 | 0.832444 | 9.110171084 | 0.004352 |
| exo_circ_61133 | hsa_circ_0006411 | 0.293394 | 2.668804 | 9.096308458 | 1.48E-08 |
| exo_circ_63658 | hsa_circ_0078214 | 0.054273 | 0.492356 | 9.071780998 | 0.019866 |
| exo_circ_39430 | hsa_circ_0001194 | 0.033068 | 0.298726 | 9.033574182 | 0.041558 |
| exo_circ_70823 | hsa_circ_0004116 | 0.126849 | 1.14532 | 9.028993952 | 0.000303 |
| exo_circ_43691 | hsa_circ_0057832 | 0.096292 | 0.868318 | 9.017552146 | 0.003363 |
| exo_circ_20211 | hsa_circ_0037941 | 0.051362 | 0.460924 | 8.973992321 | 0.041599 |
| exo_circ_56292 | hsa_circ_0069589 | 0.06365 | 0.571083 | 8.972222349 | 0.01718 |
| exo_circ_05019 | hsa_circ_0000278 | 0.096915 | 0.86915 | 8.968208115 | 0.00189 |
| exo_circ_71941 | hsa_circ_0003885 | 0.111767 | 1.001587 | 8.9613822 | 0.001972 |
| exo_circ_52899 | hsa_circ_0066231 | 0.041779 | 0.373625 | 8.942860675 | 0.042432 |
| exo_circ_67155 | hsa_circ_0082423 | 0.075327 | 0.673107 | 8.935814713 | 0.017194 |
| exo_circ_71216 | hsa_circ_0083383 | 0.056056 | 0.500619 | 8.930749244 | 0.02082 |
| exo_circ_13150 | hsa_circ_0030380 | 0.052171 | 0.464861 | 8.910272823 | 0.042539 |
| exo_circ_37705 | hsa_circ_0060035 | 0.061758 | 0.550186 | 8.908761143 | 0.01343 |
| exo_circ_63476 | hsa_circ_0006754 | 0.069293 | 0.616567 | 8.897912765 | 0.017324 |
| exo_circ_04476 | hsa_circ_0008103 | 0.083375 | 0.740533 | 8.881908609 | 0.002394 |
| exo_circ_20961 | hsa_circ_0007175 | 0.036081 | 0.320421 | 8.880645179 | 0.042908 |
| exo_circ_52790 | hsa_circ_0066115 | 0.039078 | 0.345922 | 8.852148023 | 0.04335 |
| exo_circ_48286 | hsa_circ_0064307 | 0.078082 | 0.691103 | 8.850934609 | 0.005821 |
| exo_circ_74899 | hsa_circ_0089513 | 0.056789 | 0.502148 | 8.84241028 | 0.021349 |
| exo_circ_24854 | hsa_circ_0044974 | 0.093631 | 0.82501 | 8.811335817 | 0.004048 |
| exo_circ_32427 | hsa_circ_0015928 | 0.053252 | 0.468629 | 8.800184424 | 0.01989 |
| exo_circ_73897 | hsa_circ_0008043 | 0.059051 | 0.518908 | 8.787398856 | 0.013776 |
| exo_circ_12587 | hsa_circ_0030065 | 0.06598 | 0.579711 | 8.78610231 | 0.018286 |
| exo_circ_47911 | hsa_circ_0055811 | 0.054858 | 0.481932 | 8.785000493 | 0.009344 |
| exo_circ_16007 | hsa_circ_0003957 | 0.092936 | 0.816098 | 8.781298976 | 0.001257 |
| exo_circ_65286 | hsa_circ_0005893 | 0.041237 | 0.361516 | 8.766694538 | 0.043829 |
| exo_circ_31915 | hsa_circ_0015529 | 0.081462 | 0.712944 | 8.751875006 | 0.009979 |
| exo_circ_56980 | hsa_circ_0069942 | 0.059322 | 0.519045 | 8.749593241 | 0.010835 |
| exo_circ_02717 | hsa_circ_0018485 | 0.072917 | 0.637518 | 8.743008192 | 0.020601 |
| exo_circ_12203 | hsa_circ_0029836 | 0.048369 | 0.422837 | 8.74183865 | 0.04827 |
| exo_circ_13593 | hsa_circ_0030714 | 0.066328 | 0.579365 | 8.73478785 | 0.019188 |
| exo_circ_39548 | hsa_circ_0061920 | 0.161434 | 1.404657 | 8.70111039 | 5.86E-05 |
| exo_circ_12634 | hsa_circ_0008232 | 0.070198 | 0.609899 | 8.688330346 | 0.019011 |
| exo_circ_70216 | hsa_circ_0002497 | 0.07163 | 0.621904 | 8.682150497 | 0.018677 |
| exo_circ_78390 | hsa_circ_0090434 | 0.121952 | 1.058004 | 8.675601464 | 0.000782 |
| exo_circ_35443 | hsa_circ_0012409 | 0.043867 | 0.38025 | 8.668290383 | 0.044159 |
| exo_circ_67743 | hsa_circ_0003027 | 0.076442 | 0.662526 | 8.667041651 | 0.010299 |
| exo_circ_74095 | hsa_circ_0088281 | 0.115156 | 0.997623 | 8.663259077 | 0.000992 |
| exo_circ_08284 | hsa_circ_0028705 | 0.034653 | 0.299667 | 8.647596428 | 0.046944 |
| exo_circ_34618 | hsa_circ_0000045 | 0.062243 | 0.537773 | 8.639952848 | 0.006073 |
| exo_circ_69627 | hsa_circ_0080712 | 0.078578 | 0.678836 | 8.639010044 | 0.011273 |
| exo_circ_32864 | hsa_circ_0007655 | 0.079942 | 0.689893 | 8.629954773 | 0.009701 |
| exo_circ_66494 | hsa_circ_0003656 | 0.101621 | 0.876951 | 8.629598907 | 0.001844 |
| exo_circ_31732 | hsa_circ_0002846 | 0.083734 | 0.721959 | 8.622005192 | 0.010611 |
| exo_circ_04475 | hsa_circ_0024323 | 0.145276 | 1.247713 | 8.588577264 | 0.000872 |
| exo_circ_66810 | hsa_circ_0082045 | 0.046496 | 0.399156 | 8.58475796 | 0.045042 |
| exo_circ_52334 | hsa_circ_0065163 | 0.067157 | 0.575789 | 8.57382972 | 0.014272 |
| exo_circ_05458 | hsa_circ_0021640 | 0.04289 | 0.367639 | 8.571638977 | 0.044941 |
| exo_circ_71720 | hsa_circ_0083493 | 0.048727 | 0.417133 | 8.560599046 | 0.04533 |
| exo_circ_37734 | hsa_circ_0060078 | 0.287183 | 2.448239 | 8.525016147 | 4.01E-07 |
| exo_circ_57816 | hsa_circ_0073491 | 0.043281 | 0.368926 | 8.523925472 | 0.045357 |
| exo_circ_21708 | hsa_circ_0002889 | 0.055759 | 0.475163 | 8.521778323 | 0.014075 |
| exo_circ_08645 | hsa_circ_0007552 | 0.258536 | 2.199634 | 8.508035582 | 8.97E-07 |
| exo_circ_62669 | hsa_circ_0005156 | 0.048954 | 0.416101 | 8.499912004 | 0.045623 |
| exo_circ_77889 | hsa_circ_0092043 | 0.059421 | 0.50492 | 8.497353712 | 0.021801 |
| exo_circ_39083 | hsa_circ_0061421 | 0.047597 | 0.40441 | 8.496622753 | 0.024994 |
| exo_circ_04579 | hsa_circ_0024457 | 0.077253 | 0.656375 | 8.49641363 | 0.010494 |
| exo_circ_51840 | hsa_circ_0064772 | 0.046774 | 0.396909 | 8.485695675 | 0.045966 |
| exo_circ_71206 | hsa_circ_0003691 | 0.060104 | 0.509803 | 8.482060887 | 0.020799 |
| exo_circ_39320 | hsa_circ_0008572 | 0.094968 | 0.804201 | 8.46815096 | 0.002906 |
| exo_circ_27538 | hsa_circ_0048006 | 0.113196 | 0.957867 | 8.462041037 | 0.002072 |
| exo_circ_31655 | hsa_circ_0015335 | 0.063354 | 0.535919 | 8.459069988 | 0.02288 |
| exo_circ_62421 | hsa_circ_0008925 | 0.144581 | 1.22227 | 8.453852222 | 0.000232 |
| exo_circ_40781 | hsa_circ_0055906 | 0.183309 | 1.547435 | 8.441672186 | 7.22E-07 |
| exo_circ_64255 | hsa_circ_0078786 | 0.066926 | 0.564682 | 8.437386631 | 0.012483 |
| exo_circ_50877 | hsa_circ_0068404 | 0.068267 | 0.575691 | 8.432962327 | 0.020481 |
| exo_circ_03532 | hsa_circ_0002934 | 0.053962 | 0.454756 | 8.427297819 | 0.046738 |
| exo_circ_77900 | hsa_circ_0092136 | 0.119239 | 0.999948 | 8.386077772 | 0.005434 |
| exo_circ_37637 | hsa_circ_0059945 | 0.083257 | 0.697538 | 8.378117982 | 0.012981 |
| exo_circ_11231 | hsa_circ_0027703 | 0.057719 | 0.483057 | 8.369046596 | 0.021682 |
| exo_circ_35539 | hsa_circ_0008811 | 0.063479 | 0.530329 | 8.354435296 | 0.020731 |
| exo_circ_76269 | hsa_circ_0087236 | 0.041765 | 0.348525 | 8.344903552 | 0.049638 |
| exo_circ_34837 | hsa_circ_0003986 | 0.072086 | 0.600349 | 8.328245718 | 0.020965 |
| exo_circ_23751 | hsa_circ_0041498 | 0.094364 | 0.783167 | 8.299438444 | 0.005947 |
| exo_circ_15864 | hsa_circ_0032491 | 0.060442 | 0.50155 | 8.298089684 | 0.021926 |
| exo_circ_67947 | hsa_circ_0005006 | 0.062345 | 0.516517 | 8.284746812 | 0.024416 |
| exo_circ_60669 | hsa_circ_0072497 | 0.079265 | 0.655377 | 8.268206668 | 0.012431 |
| exo_circ_71076 | hsa_circ_0001821 | 0.385231 | 3.182866 | 8.262222614 | 8.96E-10 |
| exo_circ_63608 | hsa_circ_0078189 | 0.074261 | 0.612738 | 8.251117242 | 0.013695 |
| exo_circ_44997 | hsa_circ_0059132 | 0.044504 | 0.365792 | 8.21939544 | 0.04925 |
| exo_circ_59217 | hsa_circ_0004104 | 0.106404 | 0.872435 | 8.199294404 | 0.003005 |
| exo_circ_26975 | hsa_circ_0047705 | 0.055528 | 0.454942 | 8.19303959 | 0.024149 |
| exo_circ_19247 | hsa_circ_0036402 | 0.056646 | 0.463574 | 8.183719733 | 0.017971 |
| exo_circ_39955 | hsa_circ_0002180 | 0.142565 | 1.16427 | 8.166598269 | 0.000648 |
| exo_circ_50388 | hsa_circ_0067997 | 0.171056 | 1.394775 | 8.153901433 | 0.00015 |
| exo_circ_60239 | hsa_circ_0002432 | 0.065401 | 0.530474 | 8.111105137 | 0.027186 |
| exo_circ_26446 | hsa_circ_0046735 | 0.086771 | 0.703372 | 8.106022424 | 0.005498 |
| exo_circ_22219 | hsa_circ_0006958 | 0.204834 | 1.659568 | 8.10200131 | 5.2E-05 |
| exo_circ_74877 | hsa_circ_0007482 | 0.070863 | 0.57052 | 8.051068624 | 0.023888 |
| exo_circ_23816 | hsa_circ_0043785 | 0.073152 | 0.5878 | 8.035293759 | 0.024492 |
| exo_circ_11208 | hsa_circ_0002329 | 0.075187 | 0.602941 | 8.019179651 | 0.023908 |
| exo_circ_43674 | hsa_circ_0057829 | 0.169922 | 1.358062 | 7.992257598 | 0.000221 |
| exo_circ_48329 | hsa_circ_0008210 | 0.072609 | 0.58016 | 7.990167854 | 0.015437 |
| exo_circ_64809 | hsa_circ_0076165 | 0.068728 | 0.548834 | 7.985619309 | 0.025541 |
| exo_circ_39205 | hsa_circ_0061570 | 0.190312 | 1.518673 | 7.979912488 | 0.000124 |
| exo_circ_08435 | hsa_circ_0029062 | 0.151242 | 1.206649 | 7.978243246 | 0.000743 |
| exo_circ_75236 | hsa_circ_0086203 | 0.122056 | 0.97187 | 7.962511746 | 0.001198 |
| exo_circ_35722 | hsa_circ_0012633 | 0.063246 | 0.50255 | 7.945917702 | 0.023931 |
| exo_circ_22819 | hsa_circ_0042498 | 0.048373 | 0.384058 | 7.939492155 | 0.02587 |
| exo_circ_31086 | hsa_circ_0007360 | 0.11032 | 0.874166 | 7.923923524 | 0.002892 |
| exo_circ_49299 | hsa_circ_0067574 | 0.10416 | 0.823858 | 7.909532552 | 0.005415 |
| exo_circ_57711 | hsa_circ_0005770 | 0.051112 | 0.403943 | 7.90303106 | 0.026077 |
| exo_circ_44520 | hsa_circ_0058674 | 0.048855 | 0.3861 | 7.902977084 | 0.0244 |
| exo_circ_20969 | hsa_circ_0037655 | 0.070786 | 0.558779 | 7.893883775 | 0.018109 |
| exo_circ_75847 | hsa_circ_0087082 | 0.071774 | 0.566235 | 7.889105776 | 0.033321 |
| exo_circ_58540 | hsa_circ_0002602 | 0.083997 | 0.662014 | 7.881406281 | 0.013377 |
| exo_circ_50669 | hsa_circ_0001368 | 0.164134 | 1.288552 | 7.85059368 | 0.000338 |
| exo_circ_39615 | hsa_circ_0002715 | 0.17054 | 1.336918 | 7.839300519 | 0.000294 |
| exo_circ_71942 | hsa_circ_0005411 | 0.067847 | 0.530966 | 7.82596755 | 0.025139 |
| exo_circ_19163 | hsa_circ_0036347 | 0.252713 | 1.976992 | 7.823081785 | 9.62E-06 |
| exo_circ_43954 | hsa_circ_0057958 | 0.070666 | 0.552816 | 7.822904433 | 0.02565 |
| exo_circ_05464 | hsa_circ_0000288 | 0.206842 | 1.61761 | 7.820525696 | 2.25E-05 |
| exo_circ_20470 | hsa_circ_0038342 | 0.073374 | 0.57155 | 7.789550646 | 0.028037 |
| exo_circ_16023 | hsa_circ_0003670 | 0.074545 | 0.580242 | 7.783791375 | 0.01547 |
| exo_circ_54390 | hsa_circ_0006563 | 0.184027 | 1.42673 | 7.752823939 | 4.62E-05 |
| exo_circ_14026 | hsa_circ_0005185 | 0.069424 | 0.537978 | 7.749168029 | 0.016898 |
| exo_circ_26923 | hsa_circ_0047652 | 0.062418 | 0.48098 | 7.705818841 | 0.026454 |
| exo_circ_12083 | hsa_circ_0029745 | 0.064826 | 0.49858 | 7.691105976 | 0.027731 |
| exo_circ_45351 | hsa_circ_0053393 | 0.141131 | 1.084217 | 7.68234499 | 0.00087 |
| exo_circ_25666 | hsa_circ_0002504 | 0.080219 | 0.615275 | 7.669925247 | 0.02754 |
| exo_circ_50582 | hsa_circ_0068129 | 0.043481 | 0.333468 | 7.669325894 | 0.027142 |
| exo_circ_30727 | hsa_circ_0010041 | 0.068597 | 0.526048 | 7.668658253 | 0.028734 |
| exo_circ_28851 | hsa_circ_0000876 | 0.109362 | 0.835968 | 7.644064247 | 0.003626 |
| exo_circ_14935 | hsa_circ_0009104 | 0.160339 | 1.224786 | 7.63872233 | 0.000577 |
| exo_circ_21298 | hsa_circ_0039373 | 0.131156 | 1.000009 | 7.624568977 | 0.001714 |
| exo_circ_73317 | hsa_circ_0007443 | 0.576601 | 4.393415 | 7.619510324 | 2.08E-14 |
| exo_circ_09408 | hsa_circ_0002009 | 0.10877 | 0.826884 | 7.60213469 | 0.006434 |
| exo_circ_31009 | hsa_circ_0002237 | 0.19976 | 1.506163 | 7.539858557 | 0.000197 |
| exo_circ_23500 | hsa_circ_0002404 | 0.070416 | 0.52993 | 7.525676119 | 0.030383 |
| exo_circ_46098 | hsa_circ_0054322 | 0.118895 | 0.894475 | 7.523256106 | 0.003977 |
| exo_circ_12852 | hsa_circ_0030225 | 0.112604 | 0.844885 | 7.503162921 | 0.006505 |
| exo_circ_66365 | hsa_circ_0008271 | 0.056851 | 0.426165 | 7.496197617 | 0.040161 |
| exo_circ_17790 | hsa_circ_0007487 | 0.125209 | 0.933835 | 7.458231463 | 0.005559 |
| exo_circ_35615 | hsa_circ_0003494 | 0.094765 | 0.705776 | 7.447618783 | 0.01755 |
| exo_circ_43864 | hsa_circ_0057908 | 0.20121 | 1.498356 | 7.44672198 | 9.45E-05 |
| exo_circ_08782 | hsa_circ_0029392 | 0.194509 | 1.448314 | 7.446008536 | 0.000161 |
| exo_circ_41335 | hsa_circ_0007186 | 0.142428 | 1.05875 | 7.433562509 | 0.000877 |
| exo_circ_39158 | hsa_circ_0008795 | 0.111006 | 0.822645 | 7.410808135 | 0.007788 |
| exo_circ_28543 | hsa_circ_0050518 | 0.067612 | 0.500828 | 7.40742938 | 0.032728 |
| exo_circ_49652 | hsa_circ_0067729 | 0.144865 | 1.072271 | 7.401881218 | 0.00114 |
| exo_circ_74519 | hsa_circ_0001891 | 0.190704 | 1.410215 | 7.394791455 | 0.000229 |
| exo_circ_37993 | hsa_circ_0060342 | 0.061534 | 0.454337 | 7.383536161 | 0.035234 |
| exo_circ_46378 | hsa_circ_0003959 | 0.238768 | 1.760864 | 7.374798375 | 3.48E-05 |
| exo_circ_00634 | hsa_circ_0007412 | 0.066146 | 0.487441 | 7.369134448 | 0.035348 |
| exo_circ_69248 | hsa_circ_0005419 | 0.101781 | 0.748627 | 7.355291357 | 0.0109 |
| exo_circ_26473 | hsa_circ_0046747 | 0.11379 | 0.831666 | 7.308780214 | 0.007406 |
| exo_circ_65965 | hsa_circ_0008991 | 0.115646 | 0.844336 | 7.301054919 | 0.008103 |
| exo_circ_22054 | hsa_circ_0000720 | 0.125105 | 0.912939 | 7.297405846 | 0.004368 |
| exo_circ_54255 | hsa_circ_0007149 | 0.265077 | 1.933069 | 7.292483415 | 2.29E-05 |
| exo_circ_49823 | hsa_circ_0008277 | 0.098969 | 0.720544 | 7.280515354 | 0.010125 |
| exo_circ_78953 | hsa_circ_0007873 | 0.128196 | 0.930992 | 7.262252131 | 0.003737 |
| exo_circ_32515 | hsa_circ_0016121 | 0.072467 | 0.525287 | 7.248684865 | 0.016653 |
| exo_circ_60974 | hsa_circ_0072714 | 0.072608 | 0.525033 | 7.231047003 | 0.037827 |
| exo_circ_56708 | hsa_circ_0069776 | 0.095629 | 0.690472 | 7.220350163 | 0.010288 |
| exo_circ_53168 | hsa_circ_0004113 | 0.194555 | 1.400336 | 7.197637551 | 0.000281 |
| exo_circ_43251 | hsa_circ_0057552 | 0.067723 | 0.487046 | 7.191782486 | 0.032468 |
| exo_circ_06377 | hsa_circ_0023233 | 0.248117 | 1.780596 | 7.176444436 | 6.02E-05 |
| exo_circ_28470 | hsa_circ_0006480 | 0.149653 | 1.07376 | 7.174985835 | 0.003861 |
| exo_circ_10398 | hsa_circ_0027118 | 0.126428 | 0.906884 | 7.17313794 | 0.004328 |
| exo_circ_21681 | hsa_circ_0006224 | 0.101486 | 0.727751 | 7.170950652 | 0.00828 |
| exo_circ_68871 | hsa_circ_0079948 | 0.121352 | 0.867237 | 7.146452476 | 0.002512 |
| exo_circ_72397 | hsa_circ_0005847 | 0.094413 | 0.674108 | 7.139972661 | 0.009547 |
| exo_circ_53858 | hsa_circ_0070585 | 0.047616 | 0.338766 | 7.114503182 | 0.049677 |
| exo_circ_73134 | hsa_circ_0005438 | 0.065191 | 0.462049 | 7.087572898 | 0.036914 |
| exo_circ_69269 | hsa_circ_0007622 | 0.117239 | 0.82945 | 7.074877973 | 0.003209 |
| exo_circ_04763 | hsa_circ_0024656 | 0.229549 | 1.621054 | 7.061900556 | 0.000152 |
| exo_circ_05169 | hsa_circ_0021424 | 0.156388 | 1.09812 | 7.021779001 | 0.00068 |
| exo_circ_12701 | hsa_circ_0030154 | 0.131267 | 0.921601 | 7.020819502 | 0.003178 |
| exo_circ_69265 | hsa_circ_0001712 | 0.068944 | 0.484035 | 7.020653469 | 0.035267 |
| exo_circ_05335 | hsa_circ_0020815 | 0.103209 | 0.724187 | 7.016731336 | 0.010779 |
| exo_circ_46692 | hsa_circ_0054716 | 0.150744 | 1.056714 | 7.009975879 | 0.004732 |
| exo_circ_56056 | hsa_circ_0007486 | 0.085794 | 0.600589 | 7.000388497 | 0.022704 |
| exo_circ_70395 | hsa_circ_0008163 | 0.060429 | 0.422929 | 6.99875619 | 0.022236 |
| exo_circ_46253 | hsa_circ_0003478 | 0.082949 | 0.579371 | 6.98466704 | 0.037159 |
| exo_circ_30462 | hsa_circ_0014013 | 0.149669 | 1.045243 | 6.98369939 | 0.005299 |
| exo_circ_21981 | hsa_circ_0008913 | 0.07339 | 0.512426 | 6.982215873 | 0.025618 |
| exo_circ_39459 | hsa_circ_0061750 | 0.054589 | 0.381071 | 6.980764814 | 0.047608 |
| exo_circ_13932 | hsa_circ_0031194 | 0.149546 | 1.04297 | 6.974249997 | 0.001314 |
| exo_circ_51873 | hsa_circ_0064794 | 0.168921 | 1.176541 | 6.965039478 | 0.000962 |
| exo_circ_74932 | hsa_circ_0002647 | 0.089702 | 0.624734 | 6.964558643 | 0.00818 |
| exo_circ_73385 | hsa_circ_0001813 | 0.091944 | 0.638651 | 6.946061549 | 0.035546 |
| exo_circ_55091 | hsa_circ_0006811 | 0.080249 | 0.556562 | 6.935463447 | 0.038709 |
| exo_circ_35848 | hsa_circ_0005719 | 0.143834 | 0.996965 | 6.931362 | 0.002453 |
| exo_circ_17384 | hsa_circ_0034815 | 0.081201 | 0.561314 | 6.912601534 | 0.03641 |
| exo_circ_19828 | hsa_circ_0036962 | 0.110263 | 0.761924 | 6.91004734 | 0.00916 |
| exo_circ_16911 | hsa_circ_0008987 | 0.081257 | 0.561009 | 6.904149225 | 0.021804 |
| exo_circ_43927 | hsa_circ_0052877 | 0.184758 | 1.27248 | 6.887265775 | 0.000499 |
| exo_circ_12586 | hsa_circ_0009152 | 0.071773 | 0.494312 | 6.887146237 | 0.023986 |
| exo_circ_27835 | hsa_circ_0049375 | 0.158313 | 1.089826 | 6.88401846 | 0.000837 |
| exo_circ_01474 | hsa_circ_0017956 | 0.153055 | 1.050292 | 6.862197074 | 0.004831 |
| exo_circ_66085 | hsa_circ_0077294 | 0.078203 | 0.536443 | 6.859658308 | 0.024915 |
| exo_circ_01554 | hsa_circ_0017974 | 0.30899 | 2.115076 | 6.845123288 | 1.44E-05 |
| exo_circ_28336 | hsa_circ_0008122 | 0.077134 | 0.52785 | 6.843326302 | 0.040339 |
| exo_circ_33996 | hsa_circ_0017239 | 0.130153 | 0.888672 | 6.827894584 | 0.009975 |
| exo_circ_66481 | hsa_circ_0081829 | 0.273861 | 1.86917 | 6.825244823 | 4.47E-05 |
| exo_circ_53956 | hsa_circ_0070660 | 0.235463 | 1.606566 | 6.823006736 | 0.000131 |
| exo_circ_00929 | hsa_circ_0008915 | 0.108302 | 0.73645 | 6.799962191 | 0.012246 |
| exo_circ_33632 | hsa_circ_0005223 | 0.162827 | 1.106357 | 6.794679856 | 0.001629 |
| exo_circ_68640 | hsa_circ_0004730 | 0.078155 | 0.530503 | 6.787835279 | 0.019358 |
| exo_circ_04346 | hsa_circ_0024251 | 0.450592 | 3.05057 | 6.770133676 | 2.48E-08 |
| exo_circ_72017 | hsa_circ_0083854 | 0.06342 | 0.428763 | 6.760735756 | 0.042044 |
| exo_circ_38451 | hsa_circ_0060773 | 0.084551 | 0.569695 | 6.737885259 | 0.039168 |
| exo_circ_75890 | hsa_circ_0007368 | 0.063429 | 0.426215 | 6.719583012 | 0.021363 |
| exo_circ_41298 | hsa_circ_0056257 | 0.095902 | 0.64437 | 6.719068833 | 0.023587 |
| exo_circ_06582 | hsa_circ_0023562 | 0.313615 | 2.10228 | 6.703380106 | 2.15E-05 |
| exo_circ_24055 | hsa_circ_0008436 | 0.121449 | 0.812308 | 6.688446191 | 0.005422 |
| exo_circ_60792 | hsa_circ_0071653 | 0.130809 | 0.872906 | 6.673160997 | 0.009996 |
| exo_circ_50997 | hsa_circ_0003759 | 0.553885 | 3.689093 | 6.660392649 | 8.48E-10 |
| exo_circ_39710 | hsa_circ_0062327 | 0.050094 | 0.333125 | 6.64999025 | 0.047808 |
| exo_circ_42793 | hsa_circ_0005775 | 0.282915 | 1.881219 | 6.64941007 | 1E-05 |
| exo_circ_45802 | hsa_circ_0054086 | 0.135073 | 0.897078 | 6.64143628 | 0.003421 |
| exo_circ_56215 | hsa_circ_0006567 | 0.130637 | 0.867289 | 6.638924699 | 0.010784 |
| exo_circ_02604 | hsa_circ_0003658 | 0.137568 | 0.911474 | 6.625607617 | 0.010714 |
| exo_circ_44608 | hsa_circ_0058754 | 0.130929 | 0.867141 | 6.623005647 | 0.005576 |
| exo_circ_43563 | hsa_circ_0057781 | 0.135461 | 0.896779 | 6.62018793 | 0.010715 |
| exo_circ_05964 | hsa_circ_0005795 | 0.067381 | 0.445542 | 6.612253994 | 0.047304 |
| exo_circ_78709 | hsa_circ_0091024 | 0.161007 | 1.063036 | 6.602404775 | 0.00103 |
| exo_circ_12326 | hsa_circ_0029926 | 0.118295 | 0.780194 | 6.59534848 | 0.010954 |
| exo_circ_30595 | hsa_circ_0014173 | 0.079111 | 0.521752 | 6.595195384 | 0.046034 |
| exo_circ_17447 | hsa_circ_0006297 | 0.142861 | 0.942024 | 6.594010094 | 0.004949 |
| exo_circ_38212 | hsa_circ_0060585 | 0.078184 | 0.513172 | 6.563626565 | 0.01367 |
| exo_circ_39000 | hsa_circ_0001966 | 0.166645 | 1.09365 | 6.562761314 | 0.003435 |
| exo_circ_59493 | hsa_circ_0074874 | 0.153817 | 1.008433 | 6.556052317 | 0.002995 |
| exo_circ_63057 | hsa_circ_0077844 | 0.142664 | 0.934328 | 6.549171702 | 0.006499 |
| exo_circ_35466 | hsa_circ_0012423 | 0.252748 | 1.654495 | 6.546032939 | 4.5E-05 |
| exo_circ_36259 | hsa_circ_0012947 | 0.085844 | 0.561882 | 6.545391329 | 0.021716 |
| exo_circ_65686 | hsa_circ_0077091 | 0.077701 | 0.508172 | 6.540134035 | 0.045891 |
| exo_circ_54552 | hsa_circ_0008825 | 0.226274 | 1.478605 | 6.534566536 | 0.00027 |
| exo_circ_37075 | hsa_circ_0059430 | 0.083321 | 0.543965 | 6.528561064 | 0.042928 |
| exo_circ_44428 | hsa_circ_0058586 | 0.065325 | 0.425281 | 6.510241232 | 0.039669 |
| exo_circ_75818 | hsa_circ_0087051 | 0.136496 | 0.885411 | 6.486737456 | 0.007472 |
| exo_circ_53518 | hsa_circ_0006630 | 0.085729 | 0.55604 | 6.485999267 | 0.049704 |
| exo_circ_34515 | hsa_circ_0000039 | 0.21384 | 1.386318 | 6.482980182 | 0.000409 |
| exo_circ_21930 | hsa_circ_0040487 | 0.139999 | 0.906343 | 6.473935247 | 0.003856 |
| exo_circ_04332 | hsa_circ_0024235 | 0.455575 | 2.947574 | 6.470007275 | 4.59E-07 |
| exo_circ_42630 | hsa_circ_0057051 | 0.182029 | 1.175157 | 6.455891551 | 0.001098 |
| exo_circ_43567 | hsa_circ_0001093 | 0.414785 | 2.673971 | 6.446645901 | 1.05E-06 |
| exo_circ_04580 | hsa_circ_0004195 | 0.297777 | 1.918914 | 6.444136036 | 8.34E-05 |
| exo_circ_37333 | hsa_circ_0005219 | 0.097121 | 0.625536 | 6.440808255 | 0.024581 |
| exo_circ_26458 | hsa_circ_0046741 | 0.153305 | 0.981016 | 6.399098734 | 0.004705 |
| exo_circ_60433 | hsa_circ_0004608 | 0.199248 | 1.274757 | 6.397831595 | 0.001981 |
| exo_circ_62347 | hsa_circ_0077520 | 0.170848 | 1.092655 | 6.395467355 | 0.00465 |
| exo_circ_14546 | hsa_circ_0031748 | 0.075672 | 0.483847 | 6.394002634 | 0.032837 |
| exo_circ_38126 | hsa_circ_0004218 | 0.070546 | 0.451065 | 6.393937218 | 0.047602 |
| exo_circ_76003 | hsa_circ_0086293 | 0.150645 | 0.962807 | 6.391234253 | 0.00488 |
| exo_circ_23904 | hsa_circ_0000771 | 0.124362 | 0.794471 | 6.388371934 | 0.007992 |
| exo_circ_39751 | hsa_circ_0062439 | 0.241031 | 1.538863 | 6.384505145 | 0.000219 |
| exo_circ_42111 | hsa_circ_0052802 | 0.15644 | 0.99847 | 6.382431316 | 0.002655 |
| exo_circ_26301 | hsa_circ_0047159 | 0.195333 | 1.245251 | 6.375002223 | 0.00112 |
| exo_circ_03216 | hsa_circ_0007221 | 0.063144 | 0.402368 | 6.372207794 | 0.047814 |
| exo_circ_47250 | hsa_circ_0055113 | 0.25142 | 1.598769 | 6.35895908 | 0.000107 |
| exo_circ_24874 | hsa_circ_0045006 | 0.151152 | 0.959601 | 6.348594211 | 0.005081 |
| exo_circ_53149 | hsa_circ_0006039 | 0.161017 | 1.021667 | 6.345102222 | 0.006764 |
| exo_circ_13750 | hsa_circ_0033426 | 0.121768 | 0.771753 | 6.337901165 | 0.012698 |
| exo_circ_17501 | hsa_circ_0034928 | 0.097016 | 0.614642 | 6.335453059 | 0.029435 |
| exo_circ_28777 | hsa_circ_0003079 | 0.115574 | 0.731676 | 6.33082287 | 0.013467 |
| exo_circ_39249 | hsa_circ_0007193 | 0.395044 | 2.499249 | 6.326506749 | 1.57E-06 |
| exo_circ_20274 | hsa_circ_0002771 | 0.101345 | 0.640208 | 6.31711902 | 0.016782 |
| exo_circ_67516 | hsa_circ_0004684 | 0.196995 | 1.243726 | 6.313502787 | 0.001962 |
| exo_circ_23981 | hsa_circ_0044025 | 0.173785 | 1.09688 | 6.311706435 | 0.00262 |
| exo_circ_72470 | hsa_circ_0084269 | 0.074715 | 0.470503 | 6.297308712 | 0.027749 |
| exo_circ_41418 | hsa_circ_0007176 | 0.123315 | 0.774971 | 6.284496321 | 0.013401 |
| exo_circ_26406 | hsa_circ_0047323 | 0.083985 | 0.527369 | 6.279355829 | 0.025123 |
| exo_circ_71932 | hsa_circ_0008761 | 0.381281 | 2.392159 | 6.274000933 | 4.5E-06 |
| exo_circ_04429 | hsa_circ_0024296 | 0.233644 | 1.464863 | 6.269628783 | 0.000348 |
| exo_circ_05578 | hsa_circ_0007605 | 0.094109 | 0.589453 | 6.263550631 | 0.019729 |
| exo_circ_27600 | hsa_circ_0003063 | 0.192921 | 1.207524 | 6.259157646 | 0.00254 |
| exo_circ_04288 | hsa_circ_0000356 | 0.369769 | 2.307595 | 6.240639976 | 6.96E-06 |
| exo_circ_59685 | hsa_circ_0008140 | 0.091432 | 0.568077 | 6.213139689 | 0.025853 |
| exo_circ_25651 | hsa_circ_0045920 | 0.120447 | 0.748221 | 6.21202508 | 0.013276 |
| exo_circ_58577 | hsa_circ_0073983 | 0.264724 | 1.641366 | 6.200292645 | 9.26E-05 |
| exo_circ_54268 | hsa_circ_0006362 | 0.07914 | 0.490622 | 6.199448201 | 0.026308 |
| exo_circ_57808 | hsa_circ_0003698 | 0.154482 | 0.957308 | 6.196870092 | 0.005307 |
| exo_circ_45137 | hsa_circ_0053036 | 0.104909 | 0.649952 | 6.195395811 | 0.026258 |
| exo_circ_52451 | hsa_circ_0065287 | 0.105625 | 0.654124 | 6.192898885 | 0.01701 |
| exo_circ_66290 | hsa_circ_0081534 | 0.250365 | 1.550282 | 6.192091894 | 0.000278 |
| exo_circ_28515 | hsa_circ_0006392 | 0.126328 | 0.781811 | 6.188739601 | 0.014846 |
| exo_circ_24633 | hsa_circ_0044796 | 0.146415 | 0.90538 | 6.18364887 | 0.0094 |
| exo_circ_53298 | hsa_circ_0066527 | 0.211691 | 1.308493 | 6.181147916 | 0.002441 |
| exo_circ_24868 | hsa_circ_0044999 | 0.129639 | 0.801182 | 6.180108971 | 0.014119 |
| exo_circ_75359 | hsa_circ_0008796 | 0.188122 | 1.157382 | 6.152283919 | 0.00159 |
| exo_circ_08010 | hsa_circ_0028343 | 0.144943 | 0.89068 | 6.145051303 | 0.004831 |
| exo_circ_28000 | hsa_circ_0004853 | 0.667793 | 4.085859 | 6.118449709 | 1.03E-10 |
| exo_circ_60463 | hsa_circ_0008621 | 0.092563 | 0.56517 | 6.105810918 | 0.027063 |
| exo_circ_25956 | hsa_circ_0046977 | 0.119695 | 0.729522 | 6.09484698 | 0.014658 |
| exo_circ_43126 | hsa_circ_0007794 | 0.128552 | 0.783394 | 6.093987517 | 0.014049 |
| exo_circ_30014 | hsa_circ_0013584 | 0.112155 | 0.682431 | 6.084699615 | 0.017762 |
| exo_circ_46547 | hsa_circ_0001007 | 0.167873 | 1.020172 | 6.077030499 | 0.004684 |
| exo_circ_30341 | hsa_circ_0000125 | 0.081827 | 0.496994 | 6.073696872 | 0.02744 |
| exo_circ_38894 | hsa_circ_0061279 | 0.235786 | 1.431883 | 6.072806229 | 0.000378 |
| exo_circ_55674 | hsa_circ_0004156 | 0.172493 | 1.047037 | 6.070013732 | 0.0047 |
| exo_circ_40988 | hsa_circ_0008953 | 0.377785 | 2.28809 | 6.056594303 | 1.84E-05 |
| exo_circ_65234 | hsa_circ_0075522 | 0.13917 | 0.842061 | 6.050586699 | 0.010076 |
| exo_circ_60611 | hsa_circ_0007572 | 0.10514 | 0.635864 | 6.047771052 | 0.027916 |
| exo_circ_10559 | hsa_circ_0027364 | 0.20215 | 1.218069 | 6.025555555 | 0.002935 |
| exo_circ_00751 | hsa_circ_0004630 | 0.300905 | 1.811984 | 6.021777753 | 0.000226 |
| exo_circ_69076 | hsa_circ_0080251 | 0.090798 | 0.546652 | 6.020545256 | 0.028155 |
| exo_circ_49157 | hsa_circ_0067503 | 0.108332 | 0.650541 | 6.005062727 | 0.030966 |
| exo_circ_07609 | hsa_circ_0007468 | 0.078864 | 0.473288 | 6.001342107 | 0.029327 |
| exo_circ_64042 | hsa_circ_0003735 | 0.094982 | 0.569902 | 6.000103006 | 0.028966 |
| exo_circ_64267 | hsa_circ_0075709 | 0.100406 | 0.601522 | 5.990880089 | 0.029048 |
| exo_circ_76824 | hsa_circ_0003981 | 0.109611 | 0.653955 | 5.966121137 | 0.028839 |
| exo_circ_33915 | hsa_circ_0003757 | 0.464172 | 2.768551 | 5.964494771 | 6.6E-07 |
| exo_circ_55102 | hsa_circ_0071300 | 0.135112 | 0.80337 | 5.945963431 | 0.018154 |
| exo_circ_57215 | hsa_circ_0070197 | 0.108488 | 0.642652 | 5.923732607 | 0.019093 |
| exo_circ_27420 | hsa_circ_0047927 | 0.102954 | 0.608793 | 5.913234463 | 0.036637 |
| exo_circ_03028 | hsa_circ_0004977 | 0.34466 | 2.037026 | 5.91025149 | 7.06E-05 |
| exo_circ_33881 | hsa_circ_0007875 | 0.181222 | 1.069284 | 5.900412706 | 0.005167 |
| exo_circ_25647 | hsa_circ_0045916 | 0.116377 | 0.686384 | 5.897944467 | 0.018972 |
| exo_circ_18511 | hsa_circ_0035729 | 0.10997 | 0.647614 | 5.888992776 | 0.020389 |
| exo_circ_21605 | hsa_circ_0040000 | 0.335513 | 1.971491 | 5.87604676 | 0.0002 |
| exo_circ_74097 | hsa_circ_0088283 | 0.118311 | 0.694261 | 5.868106984 | 0.017483 |
| exo_circ_76825 | hsa_circ_0008577 | 0.193628 | 1.13527 | 5.863150512 | 0.005393 |
| exo_circ_69512 | hsa_circ_0080480 | 0.09826 | 0.575677 | 5.858738137 | 0.032247 |
| exo_circ_40623 | hsa_circ_0004243 | 0.278515 | 1.630652 | 5.854809618 | 0.000482 |
| exo_circ_58990 | hsa_circ_0008646 | 0.097146 | 0.564865 | 5.814569504 | 0.031316 |
| exo_circ_69311 | hsa_circ_0007502 | 0.132175 | 0.765861 | 5.794274401 | 0.020728 |
| exo_circ_28539 | hsa_circ_0008037 | 0.123251 | 0.713516 | 5.789134556 | 0.018357 |
| exo_circ_47953 | hsa_circ_0007900 | 0.232996 | 1.344023 | 5.768446518 | 0.001689 |
| exo_circ_02726 | hsa_circ_0004258 | 0.132245 | 0.762299 | 5.764300749 | 0.024893 |
| exo_circ_10537 | hsa_circ_0027356 | 0.19597 | 1.128813 | 5.7601269 | 0.007414 |
| exo_circ_40628 | hsa_circ_0063769 | 0.098866 | 0.567805 | 5.743195956 | 0.033329 |
| exo_circ_03124 | hsa_circ_0017641 | 0.11862 | 0.681177 | 5.742495601 | 0.018153 |
| exo_circ_05601 | hsa_circ_0004410 | 0.299582 | 1.719096 | 5.738317633 | 0.000199 |
| exo_circ_10642 | hsa_circ_0025148 | 0.105852 | 0.607069 | 5.735073466 | 0.033153 |
| exo_circ_23782 | hsa_circ_0043695 | 0.187165 | 1.072859 | 5.73216457 | 0.007142 |
| exo_circ_70396 | hsa_circ_0081342 | 0.102264 | 0.585972 | 5.729994489 | 0.032695 |
| exo_circ_54362 | hsa_circ_0070941 | 0.131924 | 0.755594 | 5.727499325 | 0.018653 |
| exo_circ_63764 | hsa_circ_0002894 | 0.223222 | 1.278196 | 5.726116568 | 0.003658 |
| exo_circ_03877 | hsa_circ_0019275 | 0.100138 | 0.573185 | 5.723973083 | 0.041298 |
| exo_circ_58499 | hsa_circ_0002633 | 0.198849 | 1.136202 | 5.713897623 | 0.004019 |
| exo_circ_30937 | hsa_circ_0010048 | 0.096793 | 0.552727 | 5.710380307 | 0.03278 |
| exo_circ_77064 | hsa_circ_0004663 | 0.298424 | 1.703493 | 5.708300924 | 0.000259 |
| exo_circ_67556 | hsa_circ_0007518 | 0.287416 | 1.637407 | 5.696999264 | 0.000878 |
| exo_circ_76544 | hsa_circ_0087385 | 0.199783 | 1.137695 | 5.694652925 | 0.001688 |
| exo_circ_43352 | hsa_circ_0057657 | 0.262985 | 1.497583 | 5.694559444 | 0.000581 |
| exo_circ_30293 | hsa_circ_0008063 | 0.139418 | 0.793709 | 5.693000329 | 0.013147 |
| exo_circ_29357 | hsa_circ_0005598 | 0.245118 | 1.39411 | 5.687497158 | 0.001093 |
| exo_circ_38333 | hsa_circ_0060641 | 0.159819 | 0.908114 | 5.682151099 | 0.016879 |
| exo_circ_31682 | hsa_circ_0015364 | 0.105418 | 0.598762 | 5.679888406 | 0.038924 |
| exo_circ_27810 | hsa_circ_0049329 | 0.097703 | 0.554836 | 5.678798125 | 0.035445 |
| exo_circ_07790 | hsa_circ_0028190 | 0.512521 | 2.904603 | 5.667290573 | 1.96E-06 |
| exo_circ_07524 | hsa_circ_0027969 | 0.097337 | 0.551231 | 5.663115058 | 0.037411 |
| exo_circ_08765 | hsa_circ_0003855 | 0.258622 | 1.463455 | 5.658673981 | 0.00046 |
| exo_circ_06939 | hsa_circ_0023920 | 0.33517 | 1.892348 | 5.645931231 | 7.85E-05 |
| exo_circ_64185 | hsa_circ_0004556 | 0.319161 | 1.801036 | 5.643030675 | 0.000144 |
| exo_circ_45427 | hsa_circ_0000989 | 0.345653 | 1.950082 | 5.641740396 | 6.12E-05 |
| exo_circ_08358 | hsa_circ_0004756 | 0.08797 | 0.494342 | 5.619452739 | 0.023903 |
| exo_circ_13982 | hsa_circ_0005663 | 0.134671 | 0.756772 | 5.619434335 | 0.020648 |
| exo_circ_22788 | hsa_circ_0041147 | 0.091492 | 0.513949 | 5.617449033 | 0.040902 |
| exo_circ_19614 | hsa_circ_0036610 | 0.200611 | 1.125515 | 5.610431465 | 0.004861 |
| exo_circ_63150 | hsa_circ_0075648 | 0.206587 | 1.159027 | 5.610367997 | 0.001843 |
| exo_circ_08116 | hsa_circ_0002748 | 0.146686 | 0.822325 | 5.606008645 | 0.007798 |
| exo_circ_45285 | hsa_circ_0002025 | 0.112394 | 0.629872 | 5.604160653 | 0.020577 |
| exo_circ_48319 | hsa_circ_0001959 | 0.128566 | 0.720013 | 5.600347579 | 0.021461 |
| exo_circ_30720 | hsa_circ_0014507 | 0.130298 | 0.729651 | 5.59987522 | 0.012584 |
| exo_circ_13646 | hsa_circ_0033191 | 0.151335 | 0.847177 | 5.598007783 | 0.0204 |
| exo_circ_30414 | hsa_circ_0003619 | 0.114822 | 0.64213 | 5.592385951 | 0.020436 |
| exo_circ_33647 | hsa_circ_0016991 | 0.110986 | 0.62025 | 5.588526638 | 0.038861 |
| exo_circ_67099 | hsa_circ_0082356 | 0.123146 | 0.686956 | 5.578391462 | 0.027418 |
| exo_circ_66605 | hsa_circ_0081888 | 0.120098 | 0.669803 | 5.577154978 | 0.040236 |
| exo_circ_50428 | hsa_circ_0064551 | 0.28129 | 1.566473 | 5.56889518 | 0.000828 |
| exo_circ_30747 | hsa_circ_0014558 | 0.084341 | 0.469411 | 5.565624556 | 0.0409 |
| exo_circ_47322 | hsa_circ_0004643 | 0.116765 | 0.649463 | 5.562160487 | 0.035615 |
| exo_circ_59959 | hsa_circ_0075402 | 0.228743 | 1.272115 | 5.561333833 | 0.002624 |
| exo_circ_26200 | hsa_circ_0047098 | 0.262702 | 1.459323 | 5.555049801 | 0.001336 |
| exo_circ_02845 | hsa_circ_0007778 | 0.205053 | 1.135357 | 5.536896591 | 0.00494 |
| exo_circ_72147 | hsa_circ_0083964 | 0.127655 | 0.705873 | 5.529549349 | 0.021747 |
| exo_circ_14322 | hsa_circ_0004047 | 0.168465 | 0.931242 | 5.527820333 | 0.008097 |
| exo_circ_32682 | hsa_circ_0016307 | 0.106932 | 0.59068 | 5.52389306 | 0.03679 |
| exo_circ_72791 | hsa_circ_0084642 | 0.332578 | 1.834869 | 5.517107889 | 0.000199 |
| exo_circ_37300 | hsa_circ_0007894 | 0.169611 | 0.935064 | 5.512999425 | 0.008426 |
| exo_circ_20958 | hsa_circ_0005410 | 0.166058 | 0.915146 | 5.510994182 | 0.009734 |
| exo_circ_67094 | hsa_circ_0002014 | 0.138248 | 0.761884 | 5.510991811 | 0.01894 |
| exo_circ_27845 | hsa_circ_0049398 | 0.145558 | 0.801782 | 5.508345312 | 0.007848 |
| exo_circ_14357 | hsa_circ_0031646 | 0.19385 | 1.067753 | 5.508145491 | 0.00767 |
| exo_circ_20475 | hsa_circ_0006797 | 0.659849 | 3.63285 | 5.505575281 | 1.83E-08 |
| exo_circ_24808 | hsa_circ_0000793 | 0.257194 | 1.415173 | 5.502360752 | 0.00179 |
| exo_circ_33763 | hsa_circ_0017067 | 0.153369 | 0.842293 | 5.491942763 | 0.022039 |
| exo_circ_66165 | hsa_circ_0077394 | 0.111132 | 0.609711 | 5.486386315 | 0.038792 |
| exo_circ_60153 | hsa_circ_0001471 | 0.140918 | 0.77176 | 5.476646877 | 0.013742 |
| exo_circ_35229 | hsa_circ_0005246 | 0.112179 | 0.614084 | 5.474161212 | 0.039715 |
| exo_circ_32420 | hsa_circ_0003115 | 0.193005 | 1.055403 | 5.468268649 | 0.004767 |
| exo_circ_71449 | hsa_circ_0007521 | 0.087895 | 0.480552 | 5.46734817 | 0.047198 |
| exo_circ_52650 | hsa_circ_0005545 | 0.138644 | 0.756036 | 5.453077405 | 0.015861 |
| exo_circ_58984 | hsa_circ_0074376 | 0.255984 | 1.395407 | 5.451159132 | 0.00156 |
| exo_circ_33164 | hsa_circ_0016536 | 0.175388 | 0.954975 | 5.444924273 | 0.013896 |
| exo_circ_43474 | hsa_circ_0003858 | 0.121968 | 0.662986 | 5.435744489 | 0.040479 |
| exo_circ_21725 | hsa_circ_0040185 | 0.122698 | 0.665892 | 5.427089606 | 0.036873 |
| exo_circ_29699 | hsa_circ_0009716 | 0.25516 | 1.383897 | 5.423647706 | 0.001887 |
| exo_circ_50425 | hsa_circ_0002047 | 0.112281 | 0.608239 | 5.417126363 | 0.037613 |
| exo_circ_70330 | hsa_circ_0081188 | 0.123254 | 0.66696 | 5.411285265 | 0.038091 |
| exo_circ_35083 | hsa_circ_0003276 | 0.307824 | 1.664575 | 5.407562148 | 0.000175 |
| exo_circ_19143 | hsa_circ_0007840 | 0.126702 | 0.685018 | 5.406543068 | 0.016911 |
| exo_circ_56157 | hsa_circ_0001404 | 0.204323 | 1.103736 | 5.401916344 | 0.005725 |
| exo_circ_11711 | hsa_circ_0007031 | 0.406694 | 2.19032 | 5.38567007 | 4.08E-05 |
| exo_circ_50785 | hsa_circ_0068360 | 0.162236 | 0.873745 | 5.385629154 | 0.016597 |
| exo_circ_34788 | hsa_circ_0000051 | 0.683966 | 3.683539 | 5.385555162 | 3.69E-08 |
| exo_circ_37724 | hsa_circ_0060067 | 0.10882 | 0.585865 | 5.383819765 | 0.039202 |
| exo_circ_76708 | hsa_circ_0005432 | 0.14993 | 0.805422 | 5.372004445 | 0.0103 |
| exo_circ_07047 | hsa_circ_0023982 | 0.152376 | 0.818523 | 5.3717395 | 0.007907 |
| exo_circ_22292 | hsa_circ_0041079 | 0.107059 | 0.574599 | 5.367114911 | 0.041126 |
| exo_circ_41504 | hsa_circ_0003524 | 0.280347 | 1.504403 | 5.366208576 | 0.001248 |
| exo_circ_30597 | hsa_circ_0014174 | 0.130702 | 0.701195 | 5.364834076 | 0.032306 |
| exo_circ_65250 | hsa_circ_0076742 | 0.590332 | 3.165861 | 5.362845411 | 3.47E-07 |
| exo_circ_78338 | hsa_circ_0001916 | 0.14545 | 0.779712 | 5.360680495 | 0.016307 |
| exo_circ_23574 | hsa_circ_0041462 | 0.198543 | 1.062758 | 5.352776205 | 0.005405 |
| exo_circ_19600 | hsa_circ_0006899 | 0.123116 | 0.658405 | 5.347825996 | 0.040677 |
| exo_circ_46058 | hsa_circ_0054263 | 0.138049 | 0.737589 | 5.342966781 | 0.024362 |
| exo_circ_23289 | hsa_circ_0041153 | 0.111996 | 0.59817 | 5.341000834 | 0.030134 |
| exo_circ_53024 | hsa_circ_0003006 | 0.230718 | 1.230304 | 5.33250273 | 0.002973 |
| exo_circ_06078 | hsa_circ_0006748 | 0.164651 | 0.877616 | 5.33015781 | 0.00997 |
| exo_circ_52709 | hsa_circ_0001263 | 0.30142 | 1.605571 | 5.32669353 | 0.001093 |
| exo_circ_24174 | hsa_circ_0044261 | 0.153639 | 0.81661 | 5.315131119 | 0.015811 |
| exo_circ_07944 | hsa_circ_0028279 | 0.138909 | 0.736567 | 5.302522222 | 0.043703 |
| exo_circ_30299 | hsa_circ_0010024 | 0.11983 | 0.634533 | 5.295264169 | 0.042419 |
| exo_circ_79082 | hsa_circ_0006322 | 0.660238 | 3.494339 | 5.292541727 | 2.35E-07 |
| exo_circ_21839 | hsa_circ_0005016 | 0.519717 | 2.749472 | 5.2903231 | 1.3E-06 |
| exo_circ_35299 | hsa_circ_0000068 | 0.133958 | 0.705277 | 5.264899779 | 0.029986 |
| exo_circ_43753 | hsa_circ_0004642 | 0.241361 | 1.266598 | 5.24773345 | 0.003626 |
| exo_circ_02339 | hsa_circ_0006243 | 0.178337 | 0.934225 | 5.23853548 | 0.008281 |
| exo_circ_18294 | hsa_circ_0035470 | 0.124663 | 0.652999 | 5.238117651 | 0.016986 |
| exo_circ_25696 | hsa_circ_0046013 | 0.545899 | 2.856941 | 5.233460549 | 8.34E-06 |
| exo_circ_76763 | hsa_circ_0087493 | 0.138366 | 0.723203 | 5.226751385 | 0.028602 |
| exo_circ_63460 | hsa_circ_0003803 | 0.381208 | 1.986577 | 5.211272549 | 0.00019 |
| exo_circ_22886 | hsa_circ_0042654 | 0.238934 | 1.243257 | 5.203358684 | 0.00231 |
| exo_circ_47359 | hsa_circ_0007273 | 0.153675 | 0.798665 | 5.197117799 | 0.01765 |
| exo_circ_52411 | hsa_circ_0003602 | 0.206475 | 1.072616 | 5.194898631 | 0.00299 |
| exo_circ_67056 | hsa_circ_0082304 | 0.098116 | 0.509588 | 5.193741636 | 0.043327 |
| exo_circ_75880 | hsa_circ_0086195 | 0.306728 | 1.592031 | 5.190373187 | 0.000765 |
| exo_circ_66744 | hsa_circ_0007720 | 0.478441 | 2.483087 | 5.189949486 | 1.59E-05 |
| exo_circ_06937 | hsa_circ_0002513 | 0.119075 | 0.617876 | 5.18898339 | 0.01086 |
| exo_circ_34862 | hsa_circ_0011635 | 0.125669 | 0.651931 | 5.187680963 | 0.031481 |
| exo_circ_58077 | hsa_circ_0002462 | 0.42158 | 2.186929 | 5.187461795 | 2.51E-05 |
| exo_circ_13865 | hsa_circ_0033530 | 0.133586 | 0.692871 | 5.186702444 | 0.042416 |
| exo_circ_20422 | hsa_circ_0038258 | 0.249544 | 1.293472 | 5.183336668 | 0.00232 |
| exo_circ_33627 | hsa_circ_0001958 | 0.215854 | 1.118822 | 5.183230409 | 0.006663 |
| exo_circ_48231 | hsa_circ_0001326 | 0.640635 | 3.317377 | 5.178268237 | 5.83E-07 |
| exo_circ_34659 | hsa_circ_0004289 | 0.336869 | 1.743698 | 5.176197243 | 0.000507 |
| exo_circ_77901 | hsa_circ_0092137 | 0.133309 | 0.689342 | 5.171001503 | 0.033359 |
| exo_circ_45728 | hsa_circ_0002346 | 0.737962 | 3.812698 | 5.166521098 | 6.87E-08 |
| exo_circ_37297 | hsa_circ_0059556 | 0.150814 | 0.778671 | 5.163132645 | 0.027611 |
| exo_circ_44431 | hsa_circ_0058587 | 0.360203 | 1.858984 | 5.160936946 | 9.31E-05 |
| exo_circ_03062 | hsa_circ_0017628 | 0.396495 | 2.042225 | 5.150696661 | 0.000105 |
| exo_circ_28150 | hsa_circ_0003253 | 0.416485 | 2.142161 | 5.143435453 | 4.66E-05 |
| exo_circ_55880 | hsa_circ_0068943 | 0.262584 | 1.349717 | 5.140132382 | 0.002752 |
| exo_circ_27057 | hsa_circ_0047731 | 0.207056 | 1.063705 | 5.137272752 | 0.007441 |
| exo_circ_51815 | hsa_circ_0064741 | 0.121645 | 0.62482 | 5.136431678 | 0.03108 |
| exo_circ_50348 | hsa_circ_0067960 | 0.179456 | 0.921588 | 5.135463882 | 0.00881 |
| exo_circ_07741 | hsa_circ_0008949 | 0.204066 | 1.047334 | 5.132337969 | 0.007032 |
| exo_circ_23775 | hsa_circ_0041530 | 0.126809 | 0.64942 | 5.121226801 | 0.045579 |
| exo_circ_05731 | hsa_circ_0007719 | 0.291463 | 1.492245 | 5.119840697 | 0.001582 |
| exo_circ_00331 | hsa_circ_0017465 | 0.154645 | 0.791115 | 5.115674819 | 0.028192 |
| exo_circ_10368 | hsa_circ_0027090 | 0.126863 | 0.64799 | 5.107789902 | 0.036305 |
| exo_circ_53667 | hsa_circ_0070532 | 0.118726 | 0.604303 | 5.089881789 | 0.045942 |
| exo_circ_09406 | hsa_circ_0006742 | 0.184647 | 0.939265 | 5.086827703 | 0.016783 |
| exo_circ_16193 | hsa_circ_0032829 | 0.3624 | 1.838988 | 5.074474618 | 0.000249 |
| exo_circ_47251 | hsa_circ_0000970 | 0.22048 | 1.118411 | 5.072626259 | 0.00722 |
| exo_circ_20514 | hsa_circ_0005648 | 0.363539 | 1.839254 | 5.059303076 | 0.000319 |
| exo_circ_14376 | hsa_circ_0031655 | 0.10888 | 0.549571 | 5.04747378 | 0.036918 |
| exo_circ_61763 | hsa_circ_0073177 | 0.177372 | 0.89488 | 5.045223009 | 0.006375 |
| exo_circ_44476 | hsa_circ_0005116 | 0.416261 | 2.098807 | 5.042042524 | 5.41E-05 |
| exo_circ_26760 | hsa_circ_0007095 | 0.122666 | 0.618451 | 5.041736232 | 0.045625 |
| exo_circ_25334 | hsa_circ_0045455 | 0.222645 | 1.122269 | 5.04062655 | 0.004874 |
| exo_circ_40127 | hsa_circ_0006064 | 0.203726 | 1.026731 | 5.039756566 | 0.006151 |
| exo_circ_20250 | hsa_circ_0037969 | 0.196752 | 0.991196 | 5.037781571 | 0.011021 |
| exo_circ_72307 | hsa_circ_0084135 | 0.179533 | 0.903113 | 5.030344872 | 0.0104 |
| exo_circ_23439 | hsa_circ_0043142 | 0.104708 | 0.526562 | 5.028860835 | 0.024519 |
| exo_circ_36063 | hsa_circ_0007009 | 0.157547 | 0.791582 | 5.024403896 | 0.02984 |
| exo_circ_15773 | hsa_circ_0004008 | 0.168395 | 0.843378 | 5.008336157 | 0.02311 |
| exo_circ_33538 | hsa_circ_0003889 | 0.127482 | 0.638415 | 5.007870534 | 0.041064 |
| exo_circ_77562 | hsa_circ_0091453 | 0.161283 | 0.807463 | 5.006495696 | 0.031787 |
| exo_circ_28147 | hsa_circ_0049965 | 0.267051 | 1.336871 | 5.006055753 | 0.002194 |
| exo_circ_47916 | hsa_circ_0055814 | 0.133724 | 0.667955 | 4.995026638 | 0.036862 |
| exo_circ_65844 | hsa_circ_0004908 | 0.330428 | 1.647709 | 4.986590576 | 0.000553 |
| exo_circ_00759 | hsa_circ_0007813 | 0.407261 | 2.030564 | 4.985901788 | 0.000201 |
| exo_circ_68586 | hsa_circ_0006010 | 0.34044 | 1.696795 | 4.984121885 | 0.000811 |
| exo_circ_47284 | hsa_circ_0055149 | 0.236257 | 1.176861 | 4.981273269 | 0.00211 |
| exo_circ_53676 | hsa_circ_0070533 | 0.148191 | 0.737823 | 4.978860853 | 0.02257 |
| exo_circ_52419 | hsa_circ_0001262 | 0.185019 | 0.921021 | 4.977988033 | 0.012541 |
| exo_circ_40443 | hsa_circ_0008174 | 0.25555 | 1.269264 | 4.966785845 | 0.005184 |
| exo_circ_52678 | hsa_circ_0065898 | 0.194636 | 0.966297 | 4.964633726 | 0.01033 |
| exo_circ_43288 | hsa_circ_0057590 | 0.226511 | 1.122042 | 4.95358333 | 0.004097 |
| exo_circ_24871 | hsa_circ_0045001 | 0.161331 | 0.799166 | 4.953576422 | 0.021839 |
| exo_circ_22637 | hsa_circ_0002307 | 0.17264 | 0.854839 | 4.951573723 | 0.021464 |
| exo_circ_51723 | hsa_circ_0008549 | 0.370073 | 1.829841 | 4.944544798 | 0.000753 |
| exo_circ_44397 | hsa_circ_0058539 | 0.137093 | 0.677095 | 4.938951134 | 0.037445 |
| exo_circ_46976 | hsa_circ_0054961 | 0.117663 | 0.580762 | 4.935802933 | 0.049479 |
| exo_circ_66057 | hsa_circ_0077280 | 0.226345 | 1.113886 | 4.92118772 | 0.004106 |
| exo_circ_46052 | hsa_circ_0054248 | 0.347425 | 1.706823 | 4.912786431 | 0.001152 |
| exo_circ_71306 | hsa_circ_0007209 | 0.398557 | 1.957662 | 4.911880816 | 0.000243 |
| exo_circ_63769 | hsa_circ_0078327 | 0.140309 | 0.688688 | 4.908353924 | 0.037155 |
| exo_circ_27834 | hsa_circ_0005973 | 0.561467 | 2.75262 | 4.902548063 | 9.12E-06 |
| exo_circ_16830 | hsa_circ_0034102 | 0.099714 | 0.488329 | 4.897302324 | 0.044997 |
| exo_circ_35362 | hsa_circ_0012378 | 0.135412 | 0.661369 | 4.884115051 | 0.022588 |
| exo_circ_23840 | hsa_circ_0043815 | 0.302479 | 1.476994 | 4.882970219 | 0.001304 |
| exo_circ_51620 | hsa_circ_0064622 | 0.316366 | 1.542401 | 4.875368824 | 0.001575 |
| exo_circ_66224 | hsa_circ_0077426 | 0.141662 | 0.690242 | 4.872441154 | 0.035147 |
| exo_circ_23148 | hsa_circ_0042835 | 0.120954 | 0.589295 | 4.872060055 | 0.037633 |
| exo_circ_64953 | hsa_circ_0076254 | 0.151885 | 0.73882 | 4.864340327 | 0.035134 |
| exo_circ_34783 | hsa_circ_0011537 | 0.126899 | 0.617197 | 4.863692414 | 0.034648 |
| exo_circ_04877 | hsa_circ_0002083 | 0.726472 | 3.52696 | 4.854918212 | 6.1E-07 |
| exo_circ_26201 | hsa_circ_0007504 | 0.286261 | 1.389046 | 4.852376667 | 0.002044 |
| exo_circ_52415 | hsa_circ_0065264 | 0.146345 | 0.709926 | 4.851038972 | 0.037032 |
| exo_circ_43680 | hsa_circ_0003493 | 0.265932 | 1.287091 | 4.839916933 | 0.00381 |
| exo_circ_19104 | hsa_circ_0005073 | 0.180792 | 0.874748 | 4.838426976 | 0.018501 |
| exo_circ_29099 | hsa_circ_0004272 | 0.163981 | 0.792519 | 4.832990628 | 0.026837 |
| exo_circ_05163 | hsa_circ_0021412 | 0.319822 | 1.545168 | 4.831339174 | 0.000385 |
| exo_circ_32850 | hsa_circ_0008745 | 0.284477 | 1.373478 | 4.828079671 | 0.000712 |
| exo_circ_46908 | hsa_circ_0003497 | 0.582504 | 2.811339 | 4.826300086 | 6.32E-06 |
| exo_circ_16892 | hsa_circ_0003398 | 0.193915 | 0.935833 | 4.825995618 | 0.010441 |
| exo_circ_27833 | hsa_circ_0005412 | 0.338022 | 1.631242 | 4.825844302 | 0.000517 |
| exo_circ_15213 | hsa_circ_0032082 | 0.219162 | 1.057481 | 4.825121365 | 0.014439 |
| exo_circ_54716 | hsa_circ_0071041 | 0.216297 | 1.043421 | 4.824020144 | 0.006152 |
| exo_circ_20583 | hsa_circ_0038487 | 0.311945 | 1.50305 | 4.818308564 | 0.00212 |
| exo_circ_39996 | hsa_circ_0003490 | 0.22049 | 1.061474 | 4.814149734 | 0.006534 |
| exo_circ_45732 | hsa_circ_0007386 | 0.149702 | 0.719624 | 4.807041669 | 0.016356 |
| exo_circ_21144 | hsa_circ_0037753 | 0.319024 | 1.531636 | 4.801001425 | 0.001915 |
| exo_circ_51329 | hsa_circ_0007203 | 0.226507 | 1.086952 | 4.798764555 | 0.010027 |
| exo_circ_44014 | hsa_circ_0007225 | 0.173908 | 0.83388 | 4.794939859 | 0.016806 |
| exo_circ_31758 | hsa_circ_0005977 | 0.181916 | 0.870838 | 4.787042066 | 0.012414 |
| exo_circ_05798 | hsa_circ_0000298 | 0.14518 | 0.694464 | 4.783478605 | 0.039449 |
| exo_circ_68399 | hsa_circ_0079651 | 0.238253 | 1.138715 | 4.779426671 | 0.004744 |
| exo_circ_02315 | hsa_circ_0018315 | 0.200968 | 0.960107 | 4.77741012 | 0.013852 |
| exo_circ_50381 | hsa_circ_0067990 | 0.194934 | 0.930689 | 4.774369338 | 0.014687 |
| exo_circ_01485 | hsa_circ_0004306 | 0.208835 | 0.995236 | 4.765652663 | 0.007736 |
| exo_circ_36933 | hsa_circ_0006427 | 0.140394 | 0.666122 | 4.744662121 | 0.041801 |
| exo_circ_49871 | hsa_circ_0001350 | 0.12772 | 0.605879 | 4.743823657 | 0.042297 |
| exo_circ_21603 | hsa_circ_0003220 | 0.125926 | 0.59711 | 4.741752915 | 0.038919 |
| exo_circ_77820 | hsa_circ_0001911 | 0.268208 | 1.271664 | 4.741328725 | 0.004597 |
| exo_circ_18174 | hsa_circ_0008004 | 0.338255 | 1.603446 | 4.740350716 | 0.00119 |
| exo_circ_76545 | hsa_circ_0087386 | 0.247636 | 1.173249 | 4.737801776 | 0.006681 |
| exo_circ_10054 | hsa_circ_0006238 | 0.139441 | 0.659903 | 4.732488363 | 0.042338 |
| exo_circ_35398 | hsa_circ_0012386 | 0.189427 | 0.892835 | 4.713353449 | 0.017972 |
| exo_circ_61373 | hsa_circ_0072979 | 0.385073 | 1.814557 | 4.712248068 | 0.000801 |
| exo_circ_73263 | hsa_circ_0084868 | 0.280526 | 1.32034 | 4.706657934 | 0.006035 |
| exo_circ_33283 | hsa_circ_0007418 | 0.206407 | 0.971195 | 4.705234051 | 0.005708 |
| exo_circ_18703 | hsa_circ_0007527 | 0.38011 | 1.785432 | 4.697146318 | 0.000728 |
| exo_circ_35796 | hsa_circ_0012721 | 0.576302 | 2.705727 | 4.69498423 | 1.26E-05 |
| exo_circ_46291 | hsa_circ_0054498 | 0.222059 | 1.042372 | 4.694113738 | 0.011621 |
| exo_circ_23331 | hsa_circ_0043004 | 0.201602 | 0.945869 | 4.691756266 | 0.015865 |
| exo_circ_11244 | hsa_circ_0027707 | 0.305639 | 1.432719 | 4.687622448 | 0.003607 |
| exo_circ_12330 | hsa_circ_0029930 | 0.26228 | 1.227417 | 4.679796288 | 0.004821 |
| exo_circ_14934 | hsa_circ_0031897 | 0.263009 | 1.230764 | 4.679542604 | 0.003537 |
| exo_circ_44684 | hsa_circ_0003272 | 0.397375 | 1.858842 | 4.677803389 | 0.000479 |
| exo_circ_31843 | hsa_circ_0015482 | 0.144697 | 0.676855 | 4.677743586 | 0.044251 |
| exo_circ_52609 | hsa_circ_0065773 | 0.279728 | 1.306158 | 4.669389303 | 0.004859 |
| exo_circ_78295 | hsa_circ_0006712 | 0.28675 | 1.338446 | 4.667643792 | 0.003085 |
| exo_circ_02285 | hsa_circ_0007016 | 0.3117 | 1.452683 | 4.660514193 | 0.000941 |
| exo_circ_23142 | hsa_circ_0042828 | 0.25228 | 1.17528 | 4.658632607 | 0.004874 |
| exo_circ_21457 | hsa_circ_0039714 | 0.177713 | 0.827647 | 4.657213813 | 0.026288 |
| exo_circ_32967 | hsa_circ_0007772 | 0.157707 | 0.733493 | 4.650990829 | 0.04542 |
| exo_circ_59370 | hsa_circ_0003868 | 0.134617 | 0.625219 | 4.644422523 | 0.045524 |
| exo_circ_77741 | hsa_circ_0002871 | 0.540212 | 2.508794 | 4.64409143 | 2.53E-05 |
| exo_circ_06905 | hsa_circ_0002998 | 0.198373 | 0.92114 | 4.643467775 | 0.013239 |
| exo_circ_61757 | hsa_circ_0003761 | 0.257887 | 1.197108 | 4.641990149 | 0.005644 |
| exo_circ_59215 | hsa_circ_0074623 | 0.198248 | 0.918853 | 4.634874166 | 0.016557 |
| exo_circ_24971 | hsa_circ_0008740 | 0.213286 | 0.988263 | 4.633511635 | 0.017576 |
| exo_circ_02305 | hsa_circ_0017523 | 0.156088 | 0.723001 | 4.632004153 | 0.017069 |
| exo_circ_48124 | hsa_circ_0066716 | 0.226489 | 1.046282 | 4.619576474 | 0.008582 |
| exo_circ_26405 | hsa_circ_0047322 | 0.247861 | 1.144339 | 4.616865526 | 0.005181 |
| exo_circ_11682 | hsa_circ_0000503 | 0.207739 | 0.959014 | 4.616448302 | 0.01561 |
| exo_circ_71491 | hsa_circ_0085779 | 0.16049 | 0.739664 | 4.608770979 | 0.032273 |
| exo_circ_55088 | hsa_circ_0005272 | 0.215479 | 0.992613 | 4.606551235 | 0.013545 |
| exo_circ_44878 | hsa_circ_0006808 | 0.214379 | 0.987166 | 4.604768472 | 0.0129 |
| exo_circ_34038 | hsa_circ_0006696 | 0.297578 | 1.368088 | 4.597401369 | 0.001537 |
| exo_circ_39851 | hsa_circ_0003102 | 0.348909 | 1.601811 | 4.590917916 | 0.002102 |
| exo_circ_62861 | hsa_circ_0077771 | 0.235469 | 1.078824 | 4.581598743 | 0.017134 |
| exo_circ_44405 | hsa_circ_0004554 | 0.306875 | 1.403218 | 4.572610928 | 0.003339 |
| exo_circ_59101 | hsa_circ_0005145 | 0.624661 | 2.851186 | 4.564373324 | 2.23E-06 |
| exo_circ_55615 | hsa_circ_0004874 | 0.922397 | 4.209919 | 4.564107261 | 6.38E-09 |
| exo_circ_14818 | hsa_circ_0004040 | 0.205124 | 0.935847 | 4.562338772 | 0.010907 |
| exo_circ_40569 | hsa_circ_0063679 | 0.214934 | 0.980506 | 4.561894705 | 0.013061 |
| exo_circ_54777 | hsa_circ_0007970 | 0.15336 | 0.699565 | 4.56159373 | 0.044837 |
| exo_circ_61396 | hsa_circ_0007559 | 0.650996 | 2.962323 | 4.550444815 | 9.16E-06 |
| exo_circ_44202 | hsa_circ_0058267 | 0.135297 | 0.615323 | 4.547951093 | 0.045461 |
| exo_circ_58256 | hsa_circ_0073716 | 0.182915 | 0.831121 | 4.543762862 | 0.020593 |
| exo_circ_21783 | hsa_circ_0009163 | 0.409449 | 1.860221 | 4.543225563 | 0.001139 |
| exo_circ_12932 | hsa_circ_0005263 | 0.560059 | 2.541502 | 4.537918891 | 6.6E-05 |
| exo_circ_00287 | hsa_circ_0017441 | 0.147551 | 0.667793 | 4.525846466 | 0.037108 |
| exo_circ_69606 | hsa_circ_0080650 | 0.150823 | 0.681356 | 4.517573575 | 0.023985 |
| exo_circ_72651 | hsa_circ_0005722 | 0.194774 | 0.87951 | 4.515542742 | 0.029102 |
| exo_circ_76221 | hsa_circ_0087219 | 0.292816 | 1.321087 | 4.511659331 | 0.006301 |
| exo_circ_30577 | hsa_circ_0005710 | 0.203616 | 0.91827 | 4.50981106 | 0.011357 |
| exo_circ_62112 | hsa_circ_0073378 | 0.180291 | 0.813066 | 4.509738166 | 0.028716 |
| exo_circ_13595 | hsa_circ_0030716 | 0.150087 | 0.676689 | 4.508652064 | 0.03382 |
| exo_circ_43488 | hsa_circ_0002860 | 0.377905 | 1.702873 | 4.506083236 | 0.000714 |
| exo_circ_51676 | hsa_circ_0064656 | 0.294891 | 1.32822 | 4.504097008 | 0.004457 |
| exo_circ_48519 | hsa_circ_0066965 | 0.233338 | 1.050577 | 4.502375192 | 0.013459 |
| exo_circ_03410 | hsa_circ_0003018 | 0.352305 | 1.578929 | 4.481707887 | 0.003358 |
| exo_circ_47919 | hsa_circ_0008609 | 0.555667 | 2.489792 | 4.480723755 | 4.24E-05 |
| exo_circ_05984 | hsa_circ_0020671 | 0.131019 | 0.585563 | 4.469294246 | 0.047965 |
| exo_circ_18932 | hsa_circ_0003916 | 0.575854 | 2.571581 | 4.46568558 | 1.67E-05 |
| exo_circ_13939 | hsa_circ_0005957 | 0.228317 | 1.018913 | 4.462709988 | 0.020076 |
| exo_circ_35709 | hsa_circ_0012618 | 0.19416 | 0.865179 | 4.456016818 | 0.029294 |
| exo_circ_58531 | hsa_circ_0005193 | 0.314717 | 1.400475 | 4.449951585 | 0.001611 |
| exo_circ_05261 | hsa_circ_0006467 | 0.228274 | 1.015275 | 4.44761611 | 0.01042 |
| exo_circ_24902 | hsa_circ_0045045 | 0.215786 | 0.959731 | 4.447602961 | 0.009664 |
| exo_circ_69257 | hsa_circ_0079377 | 0.225235 | 1.001743 | 4.447547013 | 0.022798 |
| exo_circ_01012 | hsa_circ_0003465 | 0.173764 | 0.772808 | 4.447452855 | 0.032148 |
| exo_circ_75594 | hsa_circ_0086715 | 0.251229 | 1.117002 | 4.446145894 | 0.008255 |
| exo_circ_55354 | hsa_circ_0071422 | 0.868198 | 3.857356 | 4.442944744 | 3.19E-07 |
| exo_circ_26752 | hsa_circ_0005942 | 0.168753 | 0.748427 | 4.435034789 | 0.023299 |
| exo_circ_78340 | hsa_circ_0001917 | 0.592476 | 2.624677 | 4.430012108 | 3.3E-05 |
| exo_circ_35500 | hsa_circ_0006125 | 0.57044 | 2.526968 | 4.429855763 | 4.89E-05 |
| exo_circ_22328 | hsa_circ_0007919 | 0.47569 | 2.107077 | 4.429520367 | 0.000241 |
| exo_circ_37315 | hsa_circ_0059567 | 0.178065 | 0.788431 | 4.427770412 | 0.022934 |
| exo_circ_19407 | hsa_circ_0036441 | 0.270911 | 1.199107 | 4.426205221 | 0.010172 |
| exo_circ_26318 | hsa_circ_0002403 | 0.179818 | 0.794964 | 4.42093826 | 0.030328 |
| exo_circ_70366 | hsa_circ_0081311 | 0.898373 | 3.967715 | 4.416555591 | 9.7E-08 |
| exo_circ_08841 | hsa_circ_0029461 | 0.197608 | 0.872083 | 4.413194197 | 0.022276 |
| exo_circ_35316 | hsa_circ_0012300 | 0.974527 | 4.300383 | 4.412787281 | 9.65E-09 |
| exo_circ_18021 | hsa_circ_0035294 | 0.297204 | 1.311274 | 4.412031584 | 0.004777 |
| exo_circ_54071 | hsa_circ_0008493 | 0.209703 | 0.923041 | 4.401660547 | 0.010537 |
| exo_circ_55276 | hsa_circ_0009006 | 0.545429 | 2.400789 | 4.401652585 | 0.000118 |
| exo_circ_48707 | hsa_circ_0006118 | 0.252415 | 1.110458 | 4.399342308 | 0.009188 |
| exo_circ_31795 | hsa_circ_0015446 | 0.25255 | 1.110377 | 4.396661679 | 0.014665 |
| exo_circ_54829 | hsa_circ_0009048 | 0.317532 | 1.395894 | 4.396068177 | 0.004038 |
| exo_circ_57556 | hsa_circ_0006866 | 0.244965 | 1.076277 | 4.393593979 | 0.010316 |
| exo_circ_39432 | hsa_circ_0061728 | 0.193484 | 0.849596 | 4.391032698 | 0.021627 |
| exo_circ_67826 | hsa_circ_0002810 | 0.514437 | 2.256541 | 4.386425062 | 7.06E-05 |
| exo_circ_42188 | hsa_circ_0056810 | 0.464109 | 2.034872 | 4.384473034 | 0.000211 |
| exo_circ_15983 | hsa_circ_0005030 | 0.68942 | 3.020243 | 4.380848176 | 8.56E-06 |
| exo_circ_43380 | hsa_circ_0003915 | 0.358237 | 1.56916 | 4.380231337 | 0.000798 |
| exo_circ_46910 | hsa_circ_0002370 | 0.167638 | 0.734183 | 4.379566706 | 0.042146 |
| exo_circ_39943 | hsa_circ_0005622 | 0.193477 | 0.847159 | 4.378611506 | 0.033779 |
| exo_circ_30617 | hsa_circ_0014191 | 0.199799 | 0.87467 | 4.377741285 | 0.031967 |
| exo_circ_38024 | hsa_circ_0006873 | 0.392616 | 1.717943 | 4.375630229 | 0.001419 |
| exo_circ_30633 | hsa_circ_0000133 | 0.247982 | 1.084369 | 4.372769617 | 0.01035 |
| exo_circ_51164 | hsa_circ_0008444 | 0.26615 | 1.163207 | 4.370500799 | 0.005386 |
| exo_circ_03730 | hsa_circ_0019170 | 0.350189 | 1.529781 | 4.368445092 | 0.000946 |
| exo_circ_71957 | hsa_circ_0083824 | 0.805544 | 3.514914 | 4.363404221 | 5.28E-07 |
| exo_circ_40637 | hsa_circ_0001247 | 1.055922 | 4.605914 | 4.361983125 | 1.19E-09 |
| exo_circ_27606 | hsa_circ_0002872 | 0.7148 | 3.108971 | 4.34942656 | 1.49E-05 |
| exo_circ_77066 | hsa_circ_0089865 | 0.1736 | 0.754846 | 4.348191592 | 0.02849 |
| exo_circ_47009 | hsa_circ_0008529 | 0.212877 | 0.925096 | 4.345674206 | 0.019744 |
| exo_circ_65834 | hsa_circ_0077183 | 0.156215 | 0.677989 | 4.340099191 | 0.039715 |
| exo_circ_26908 | hsa_circ_0007006 | 0.179215 | 0.777393 | 4.337768263 | 0.033307 |
| exo_circ_50639 | hsa_circ_0003085 | 0.184121 | 0.798648 | 4.337621449 | 0.028181 |
| exo_circ_18828 | hsa_circ_0035952 | 0.304254 | 1.319501 | 4.336845112 | 0.004508 |
| exo_circ_35921 | hsa_circ_0012782 | 0.532636 | 2.306156 | 4.329705386 | 0.000139 |
| exo_circ_67562 | hsa_circ_0005106 | 0.384867 | 1.660647 | 4.314860167 | 0.001638 |
| exo_circ_58679 | hsa_circ_0005222 | 0.25369 | 1.093987 | 4.312299869 | 0.012173 |
| exo_circ_04751 | hsa_circ_0002955 | 0.506631 | 2.181787 | 4.306463879 | 0.000205 |
| exo_circ_02946 | hsa_circ_0007097 | 0.171143 | 0.735565 | 4.297945762 | 0.024283 |
| exo_circ_43274 | hsa_circ_0057569 | 0.349392 | 1.501201 | 4.296612274 | 0.003578 |
| exo_circ_27688 | hsa_circ_0007808 | 0.238469 | 1.024203 | 4.29491228 | 0.024512 |
| exo_circ_01963 | hsa_circ_0002351 | 0.258927 | 1.11201 | 4.294691337 | 0.011386 |
| exo_circ_40881 | hsa_circ_0000977 | 0.337438 | 1.449054 | 4.294277543 | 0.002442 |
| exo_circ_25821 | hsa_circ_0005281 | 0.301681 | 1.294504 | 4.290965035 | 0.005392 |
| exo_circ_73332 | hsa_circ_0084889 | 0.134464 | 0.574904 | 4.275532353 | 0.044091 |
| exo_circ_37684 | hsa_circ_0008213 | 0.305027 | 1.30227 | 4.269362778 | 0.007869 |
| exo_circ_70976 | hsa_circ_0085456 | 0.174406 | 0.74421 | 4.267123931 | 0.035531 |
| exo_circ_06667 | hsa_circ_0023653 | 0.177412 | 0.756751 | 4.265494533 | 0.038239 |
| exo_circ_08511 | hsa_circ_0029111 | 0.194503 | 0.82866 | 4.260386954 | 0.027415 |
| exo_circ_00059 | hsa_circ_0005741 | 0.45627 | 1.935605 | 4.242232793 | 0.00055 |
| exo_circ_34789 | hsa_circ_0011542 | 0.598678 | 2.536703 | 4.237176462 | 8.96E-05 |
| exo_circ_55749 | hsa_circ_0069338 | 0.488775 | 2.070759 | 4.23663158 | 0.000481 |
| exo_circ_34863 | hsa_circ_0011636 | 0.177841 | 0.752793 | 4.232964417 | 0.037801 |
| exo_circ_74346 | hsa_circ_0001887 | 0.234521 | 0.990939 | 4.225377963 | 0.019607 |
| exo_circ_07528 | hsa_circ_0003012 | 0.268788 | 1.133549 | 4.217255421 | 0.013245 |
| exo_circ_22638 | hsa_circ_0004488 | 0.297661 | 1.254139 | 4.213311443 | 0.006694 |
| exo_circ_61412 | hsa_circ_0073027 | 0.482421 | 2.031251 | 4.210537594 | 0.000615 |
| exo_circ_02723 | hsa_circ_0004472 | 0.176728 | 0.743955 | 4.209616281 | 0.036885 |
| exo_circ_28537 | hsa_circ_0050511 | 0.15388 | 0.647691 | 4.209065657 | 0.0377 |
| exo_circ_35319 | hsa_circ_0012301 | 0.274571 | 1.155381 | 4.207942463 | 0.012399 |
| exo_circ_11980 | hsa_circ_0003852 | 0.256647 | 1.079439 | 4.205928858 | 0.017386 |
| exo_circ_16901 | hsa_circ_0005387 | 0.535137 | 2.250247 | 4.204991653 | 0.000345 |
| exo_circ_53066 | hsa_circ_0002134 | 0.269742 | 1.133065 | 4.200550967 | 0.012472 |
| exo_circ_61438 | hsa_circ_0007350 | 0.231295 | 0.971099 | 4.198531621 | 0.027352 |
| exo_circ_74242 | hsa_circ_0002812 | 0.230615 | 0.96817 | 4.198201238 | 0.028806 |
| exo_circ_35239 | hsa_circ_0002563 | 0.17563 | 0.736573 | 4.193882676 | 0.038895 |
| exo_circ_08127 | hsa_circ_0002175 | 0.438678 | 1.839611 | 4.193529908 | 0.001213 |
| exo_circ_28458 | hsa_circ_0000925 | 0.627612 | 2.628579 | 4.188225413 | 5.83E-05 |
| exo_circ_39736 | hsa_circ_0062426 | 0.322467 | 1.34929 | 4.184267829 | 0.002918 |
| exo_circ_04675 | hsa_circ_0000362 | 0.687513 | 2.866266 | 4.169035988 | 2.54E-05 |
| exo_circ_41185 | hsa_circ_0052428 | 0.245912 | 1.02488 | 4.167667736 | 0.018997 |
| exo_circ_59796 | hsa_circ_0004138 | 0.497101 | 2.06974 | 4.163617613 | 0.000262 |
| exo_circ_44901 | hsa_circ_0059007 | 0.16669 | 0.693531 | 4.16060127 | 0.049425 |
| exo_circ_03079 | hsa_circ_0017636 | 1.02504 | 4.263013 | 4.15887394 | 9.07E-08 |
| exo_circ_01182 | hsa_circ_0017829 | 0.257706 | 1.071252 | 4.156875124 | 0.010006 |
| exo_circ_13640 | hsa_circ_0033180 | 0.463946 | 1.92788 | 4.155398886 | 0.000678 |
| exo_circ_61331 | hsa_circ_0072940 | 0.190539 | 0.791697 | 4.155041578 | 0.039487 |
| exo_circ_37353 | hsa_circ_0006756 | 0.294754 | 1.224696 | 4.154969428 | 0.013335 |
| exo_circ_31999 | hsa_circ_0006507 | 0.304873 | 1.264463 | 4.147512318 | 0.00918 |
| exo_circ_58663 | hsa_circ_0074063 | 0.22156 | 0.918728 | 4.146630282 | 0.019254 |
| exo_circ_73310 | hsa_circ_0084878 | 0.340092 | 1.410118 | 4.146289071 | 0.005452 |
| exo_circ_53300 | hsa_circ_0066529 | 0.323284 | 1.337759 | 4.138026483 | 0.005259 |
| exo_circ_16951 | hsa_circ_0034294 | 0.378708 | 1.566266 | 4.135811421 | 0.002585 |
| exo_circ_28381 | hsa_circ_0050334 | 0.71485 | 2.956369 | 4.135649128 | 8.36E-06 |
| exo_circ_43816 | hsa_circ_0005613 | 0.202239 | 0.836334 | 4.13537164 | 0.040741 |
| exo_circ_62229 | hsa_circ_0001513 | 0.307588 | 1.269527 | 4.127357562 | 0.003923 |
| exo_circ_33063 | hsa_circ_0002843 | 0.389872 | 1.607562 | 4.123305792 | 0.001165 |
| exo_circ_23139 | hsa_circ_0041443 | 0.194472 | 0.801486 | 4.121343738 | 0.029631 |
| exo_circ_04330 | hsa_circ_0024234 | 0.663453 | 2.732978 | 4.119324155 | 1.71E-05 |
| exo_circ_47564 | hsa_circ_0055517 | 0.23125 | 0.952337 | 4.118206812 | 0.015891 |
| exo_circ_20501 | hsa_circ_0006795 | 0.543689 | 2.237206 | 4.114864499 | 0.000227 |
| exo_circ_45435 | hsa_circ_0053535 | 0.297885 | 1.224092 | 4.109279636 | 0.011182 |
| exo_circ_33916 | hsa_circ_0010902 | 0.162774 | 0.668454 | 4.106628245 | 0.048378 |
| exo_circ_25988 | hsa_circ_0046999 | 0.626105 | 2.565888 | 4.098177397 | 0.00019 |
| exo_circ_22298 | hsa_circ_0002631 | 0.320281 | 1.310484 | 4.091664303 | 0.007371 |
| exo_circ_51021 | hsa_circ_0008124 | 0.792582 | 3.240582 | 4.088636888 | 1.88E-06 |
| exo_circ_64795 | hsa_circ_0001599 | 0.994319 | 4.0646 | 4.087823218 | 3.84E-07 |
| exo_circ_35105 | hsa_circ_0011938 | 0.315254 | 1.288513 | 4.087218136 | 0.006907 |
| exo_circ_72976 | hsa_circ_0008736 | 0.360565 | 1.472098 | 4.082749127 | 0.002012 |
| exo_circ_32490 | hsa_circ_0007167 | 0.874214 | 3.566492 | 4.079656071 | 1.18E-06 |
| exo_circ_46616 | hsa_circ_0003165 | 0.509368 | 2.077373 | 4.078331393 | 0.000523 |
| exo_circ_21342 | hsa_circ_0039389 | 0.190983 | 0.776603 | 4.066346847 | 0.049805 |
| exo_circ_73613 | hsa_circ_0085081 | 0.285718 | 1.16045 | 4.06152836 | 0.011509 |
| exo_circ_71990 | hsa_circ_0004283 | 0.423993 | 1.721657 | 4.060574657 | 0.002487 |
| exo_circ_75588 | hsa_circ_0002976 | 0.369723 | 1.501209 | 4.060367268 | 0.002496 |
| exo_circ_12497 | hsa_circ_0030042 | 0.75991 | 3.080639 | 4.053951631 | 1.68E-05 |
| exo_circ_50698 | hsa_circ_0009131 | 0.599693 | 2.429545 | 4.051317078 | 9.76E-05 |
| exo_circ_29709 | hsa_circ_0008936 | 0.265541 | 1.075255 | 4.049295591 | 0.016125 |
| exo_circ_07792 | hsa_circ_0028196 | 0.284038 | 1.149902 | 4.048414573 | 0.001198 |
| exo_circ_71630 | hsa_circ_0002887 | 0.653802 | 2.646774 | 4.048278391 | 4.89E-05 |
| exo_circ_74018 | hsa_circ_0088087 | 0.239671 | 0.966759 | 4.033686396 | 0.021479 |
| exo_circ_74964 | hsa_circ_0001905 | 0.207239 | 0.83514 | 4.029831563 | 0.033459 |
| exo_circ_00747 | hsa_circ_0017724 | 0.375172 | 1.508421 | 4.02061535 | 0.003361 |
| exo_circ_68528 | hsa_circ_0079701 | 0.247706 | 0.994327 | 4.014140752 | 0.022389 |
| exo_circ_20643 | hsa_circ_0008867 | 0.233528 | 0.935757 | 4.007048133 | 0.034574 |
| exo_circ_19829 | hsa_circ_0036963 | 0.663805 | 2.659271 | 4.006102257 | 7.75E-05 |
| exo_circ_19803 | hsa_circ_0003252 | 0.208033 | 0.832394 | 4.001261383 | 0.039368 |
| exo_circ_55298 | hsa_circ_0004303 | 0.447777 | 1.791554 | 4.000995702 | 0.001414 |
| exo_circ_76180 | hsa_circ_0087196 | 0.238691 | 0.954962 | 4.000823046 | 0.044494 |
| exo_circ_58674 | hsa_circ_0074066 | 0.184095 | 0.735243 | 3.993821933 | 0.045145 |
| exo_circ_27602 | hsa_circ_0007144 | 0.396121 | 1.58158 | 3.992663991 | 0.003571 |
| exo_circ_78711 | hsa_circ_0091025 | 0.349102 | 1.393634 | 3.992058105 | 0.004971 |
| exo_circ_09004 | hsa_circ_0025006 | 0.278974 | 1.113489 | 3.991378346 | 0.010215 |
| exo_circ_50202 | hsa_circ_0001274 | 0.692862 | 2.764556 | 3.990053748 | 8.08E-05 |
| exo_circ_34256 | hsa_circ_0002802 | 0.23694 | 0.94461 | 3.986708361 | 0.010536 |
| exo_circ_29235 | hsa_circ_0052165 | 0.760814 | 3.033079 | 3.986621652 | 1.03E-05 |
| exo_circ_20804 | hsa_circ_0007768 | 0.205614 | 0.819238 | 3.984343306 | 0.028624 |
| exo_circ_15784 | hsa_circ_0001997 | 0.387269 | 1.542286 | 3.982466722 | 0.00418 |
| exo_circ_69186 | hsa_circ_0007823 | 0.582745 | 2.320545 | 3.982093839 | 0.000787 |
| exo_circ_48850 | hsa_circ_0001337 | 0.682126 | 2.71322 | 3.977592636 | 4.34E-05 |
| exo_circ_48105 | hsa_circ_0004385 | 0.317195 | 1.260495 | 3.97387557 | 0.010856 |
| exo_circ_38639 | hsa_circ_0060989 | 0.929959 | 3.691353 | 3.969373089 | 1.22E-06 |
| exo_circ_77726 | hsa_circ_0008579 | 0.238024 | 0.944416 | 3.967742757 | 0.024775 |
| exo_circ_18512 | hsa_circ_0035731 | 0.296853 | 1.177151 | 3.96544043 | 0.013358 |
| exo_circ_36621 | hsa_circ_0013083 | 0.246068 | 0.974743 | 3.961272635 | 0.023825 |
| exo_circ_30525 | hsa_circ_0006352 | 0.262342 | 1.037003 | 3.952873107 | 0.02353 |
| exo_circ_77334 | hsa_circ_0003300 | 0.259937 | 1.027329 | 3.952223913 | 0.024198 |
| exo_circ_13390 | hsa_circ_0030537 | 0.443116 | 1.749642 | 3.948493576 | 0.002165 |
| exo_circ_34492 | hsa_circ_0011162 | 0.315118 | 1.243988 | 3.947689708 | 0.004952 |
| exo_circ_60507 | hsa_circ_0072428 | 0.871405 | 3.436301 | 3.943401954 | 3.45E-06 |
| exo_circ_38136 | hsa_circ_0060484 | 0.407724 | 1.60737 | 3.942294785 | 0.003982 |
| exo_circ_11566 | hsa_circ_0030763 | 0.254734 | 1.003439 | 3.939162678 | 0.01649 |
| exo_circ_52492 | hsa_circ_0065336 | 0.577488 | 2.274553 | 3.938702033 | 0.000293 |
| exo_circ_27955 | hsa_circ_0005660 | 0.277099 | 1.09006 | 3.933827684 | 0.022407 |
| exo_circ_21572 | hsa_circ_0000712 | 0.234429 | 0.921395 | 3.930372078 | 0.020578 |
| exo_circ_03644 | hsa_circ_0004224 | 0.39668 | 1.558104 | 3.927862283 | 0.00323 |
| exo_circ_16037 | hsa_circ_0032682 | 0.344919 | 1.354288 | 3.92639185 | 0.008496 |
| exo_circ_61445 | hsa_circ_0073049 | 0.226846 | 0.889885 | 3.922861315 | 0.037936 |
| exo_circ_11406 | hsa_circ_0027771 | 0.202916 | 0.795374 | 3.919716458 | 0.036389 |
| exo_circ_18697 | hsa_circ_0035897 | 0.263811 | 1.033793 | 3.918682466 | 0.028739 |
| exo_circ_73851 | hsa_circ_0008706 | 0.393973 | 1.54344 | 3.917623813 | 0.00444 |
| exo_circ_15780 | hsa_circ_0007996 | 0.213002 | 0.833639 | 3.913756754 | 0.037294 |
| exo_circ_26170 | hsa_circ_0008520 | 0.376146 | 1.471444 | 3.911900819 | 0.007229 |
| exo_circ_29008 | hsa_circ_0005261 | 0.385093 | 1.50615 | 3.911132356 | 0.002092 |
| exo_circ_29707 | hsa_circ_0000015 | 0.268014 | 1.048184 | 3.910930052 | 0.020412 |
| exo_circ_72950 | hsa_circ_0084708 | 0.904586 | 3.536594 | 3.909628601 | 6.66E-06 |
| exo_circ_69312 | hsa_circ_0006562 | 0.18283 | 0.714712 | 3.909163487 | 0.038953 |
| exo_circ_40564 | hsa_circ_0002610 | 0.320248 | 1.25097 | 3.906253722 | 0.013435 |
| exo_circ_72859 | hsa_circ_0084668 | 0.291376 | 1.137937 | 3.905389929 | 0.023253 |
| exo_circ_25833 | hsa_circ_0046524 | 0.26473 | 1.032101 | 3.898692039 | 0.026278 |
| exo_circ_25301 | hsa_circ_0045431 | 0.233358 | 0.909551 | 3.897657859 | 0.026303 |
| exo_circ_11578 | hsa_circ_0030766 | 0.327876 | 1.277089 | 3.895030715 | 0.010327 |
| exo_circ_34399 | hsa_circ_0006585 | 0.247651 | 0.963595 | 3.890934745 | 0.028406 |
| exo_circ_07735 | hsa_circ_0028160 | 0.440334 | 1.712834 | 3.889849262 | 0.001756 |
| exo_circ_04878 | hsa_circ_0004296 | 1.514616 | 5.890844 | 3.889331706 | 2.26E-11 |
| exo_circ_67518 | hsa_circ_0082689 | 0.470512 | 1.82882 | 3.88686999 | 0.002039 |
| exo_circ_25729 | hsa_circ_0046085 | 0.358151 | 1.39138 | 3.884894523 | 0.005906 |
| exo_circ_78726 | hsa_circ_0008930 | 0.507445 | 1.969864 | 3.88192532 | 0.001849 |
| exo_circ_77789 | hsa_circ_0091652 | 0.249915 | 0.969436 | 3.87906181 | 0.025976 |
| exo_circ_42394 | hsa_circ_0002249 | 0.329559 | 1.277925 | 3.877688105 | 0.014696 |
| exo_circ_04508 | hsa_circ_0000357 | 0.410122 | 1.589799 | 3.876410444 | 0.003026 |
| exo_circ_34881 | hsa_circ_0004093 | 0.686461 | 2.659473 | 3.874182418 | 0.000101 |
| exo_circ_04975 | hsa_circ_0024920 | 0.432166 | 1.673462 | 3.872270476 | 0.003525 |
| exo_circ_00935 | hsa_circ_0005418 | 0.547931 | 2.121129 | 3.871160588 | 0.000731 |
| exo_circ_28902 | hsa_circ_0051449 | 0.734734 | 2.840596 | 3.866155637 | 6.09E-05 |
| exo_circ_48590 | hsa_circ_0006132 | 0.267967 | 1.035423 | 3.863992346 | 0.013112 |
| exo_circ_44388 | hsa_circ_0058514 | 0.433456 | 1.673607 | 3.861076983 | 0.002599 |
| exo_circ_66611 | hsa_circ_0081892 | 0.38588 | 1.489904 | 3.861055444 | 0.002254 |
| exo_circ_42182 | hsa_circ_0005102 | 0.330401 | 1.27401 | 3.855955467 | 0.004396 |
| exo_circ_60526 | hsa_circ_0072440 | 0.763416 | 2.942082 | 3.85383625 | 9.53E-05 |
| exo_circ_03250 | hsa_circ_0018884 | 0.356673 | 1.374103 | 3.852560101 | 0.008473 |
| exo_circ_58982 | hsa_circ_0074373 | 0.394789 | 1.520867 | 3.852350829 | 0.003535 |
| exo_circ_65771 | hsa_circ_0077142 | 0.23805 | 0.916198 | 3.848765776 | 0.028504 |
| exo_circ_79080 | hsa_circ_0092231 | 0.241317 | 0.928431 | 3.847352423 | 0.02978 |
| exo_circ_48888 | hsa_circ_0067323 | 0.826155 | 3.174709 | 3.842754017 | 5.33E-06 |
| exo_circ_55763 | hsa_circ_0001398 | 1.179713 | 4.520568 | 3.83192252 | 8.9E-08 |
| exo_circ_23161 | hsa_circ_0042843 | 0.26653 | 1.019605 | 3.825481536 | 0.028658 |
| exo_circ_22141 | hsa_circ_0000724 | 1.427398 | 5.454921 | 3.821583237 | 6.87E-10 |
| exo_circ_13850 | hsa_circ_0000572 | 0.916526 | 3.493614 | 3.811799115 | 5.75E-06 |
| exo_circ_74382 | hsa_circ_0008383 | 0.200564 | 0.763984 | 3.809171252 | 0.041417 |
| exo_circ_04589 | hsa_circ_0024470 | 0.173078 | 0.658996 | 3.807506009 | 0.046023 |
| exo_circ_17779 | hsa_circ_0035185 | 0.201814 | 0.767793 | 3.804467918 | 0.042002 |
| exo_circ_40525 | hsa_circ_0008806 | 0.648555 | 2.464368 | 3.799783866 | 0.000174 |
| exo_circ_64814 | hsa_circ_0076169 | 0.226056 | 0.858029 | 3.795644762 | 0.029548 |
| exo_circ_52907 | hsa_circ_0066241 | 0.492245 | 1.865719 | 3.790226321 | 0.000831 |
| exo_circ_74518 | hsa_circ_0008240 | 0.811261 | 3.073776 | 3.788887349 | 2.03E-05 |
| exo_circ_38140 | hsa_circ_0006484 | 0.224181 | 0.848122 | 3.783204601 | 0.037489 |
| exo_circ_23889 | hsa_circ_0000770 | 0.18504 | 0.698355 | 3.774080219 | 0.024285 |
| exo_circ_78693 | hsa_circ_0091017 | 0.268704 | 1.01144 | 3.764134096 | 0.024209 |
| exo_circ_50810 | hsa_circ_0068372 | 0.297867 | 1.121147 | 3.763914301 | 0.017036 |
| exo_circ_09143 | hsa_circ_0025608 | 0.21952 | 0.826112 | 3.763259072 | 0.034596 |
| exo_circ_66131 | hsa_circ_0003814 | 0.433373 | 1.630654 | 3.762706458 | 0.007185 |
| exo_circ_12229 | hsa_circ_0000469 | 0.379602 | 1.428273 | 3.762553333 | 0.008455 |
| exo_circ_66080 | hsa_circ_0077292 | 0.605856 | 2.27431 | 3.753878284 | 0.000495 |
| exo_circ_06029 | hsa_circ_0003900 | 0.460192 | 1.727019 | 3.752826247 | 0.003013 |
| exo_circ_22109 | hsa_circ_0040738 | 0.360896 | 1.354136 | 3.752147339 | 0.005604 |
| exo_circ_74959 | hsa_circ_0089728 | 0.719704 | 2.700234 | 3.751867964 | 6.52E-05 |
| exo_circ_33986 | hsa_circ_0017237 | 0.282027 | 1.057221 | 3.74864682 | 0.016777 |
| exo_circ_77677 | hsa_circ_0001944 | 0.654048 | 2.451673 | 3.748459692 | 0.000255 |
| exo_circ_46080 | hsa_circ_0054303 | 0.361099 | 1.352351 | 3.745094295 | 0.00864 |
| exo_circ_78966 | hsa_circ_0006301 | 0.297189 | 1.112398 | 3.743064389 | 0.008545 |
| exo_circ_41362 | hsa_circ_0002374 | 0.242357 | 0.907123 | 3.742916007 | 0.033804 |
| exo_circ_03089 | hsa_circ_0017639 | 0.459332 | 1.716165 | 3.7362188 | 0.004303 |
| exo_circ_00757 | hsa_circ_0017731 | 0.262034 | 0.97863 | 3.734742559 | 0.026574 |
| exo_circ_32893 | hsa_circ_0016394 | 0.404862 | 1.511358 | 3.733021627 | 0.009366 |
| exo_circ_43842 | hsa_circ_0004575 | 0.380056 | 1.418707 | 3.732888208 | 0.009759 |
| exo_circ_31108 | hsa_circ_0003567 | 0.305109 | 1.138808 | 3.732461548 | 0.016274 |
| exo_circ_20476 | hsa_circ_0005699 | 0.41998 | 1.565267 | 3.72699997 | 0.003719 |
| exo_circ_27813 | hsa_circ_0049336 | 0.609607 | 2.270093 | 3.723863946 | 0.000803 |
| exo_circ_69819 | hsa_circ_0080847 | 0.328036 | 1.221368 | 3.723272474 | 0.017527 |
| exo_circ_03633 | hsa_circ_0008148 | 0.817511 | 3.043614 | 3.723025436 | 4.75E-05 |
| exo_circ_52656 | hsa_circ_0002479 | 0.960589 | 3.576044 | 3.722762631 | 3.64E-06 |
| exo_circ_38332 | hsa_circ_0060640 | 1.013818 | 3.772464 | 3.721048635 | 3.26E-06 |
| exo_circ_37975 | hsa_circ_0008006 | 0.268051 | 0.99731 | 3.720604082 | 0.02177 |
| exo_circ_00888 | hsa_circ_0000264 | 0.87618 | 3.259722 | 3.720377963 | 1.81E-05 |
| exo_circ_39055 | hsa_circ_0061395 | 0.762943 | 2.836934 | 3.718409388 | 8.4E-05 |
| exo_circ_65688 | hsa_circ_0077093 | 0.312919 | 1.163292 | 3.717545445 | 0.024212 |
| exo_circ_50375 | hsa_circ_0001362 | 0.500436 | 1.86038 | 3.717517617 | 0.000777 |
| exo_circ_21953 | hsa_circ_0040528 | 0.359236 | 1.331388 | 3.706169385 | 0.016621 |
| exo_circ_27872 | hsa_circ_0006471 | 0.476278 | 1.763957 | 3.703629869 | 0.002311 |
| exo_circ_41429 | hsa_circ_0004645 | 0.248761 | 0.920166 | 3.698990326 | 0.030885 |
| exo_circ_64251 | hsa_circ_0003016 | 0.464849 | 1.719141 | 3.698280633 | 0.002767 |
| exo_circ_70635 | hsa_circ_0008320 | 0.53055 | 1.961719 | 3.69751984 | 0.000104 |
| exo_circ_74598 | hsa_circ_0088816 | 0.475754 | 1.758911 | 3.69710229 | 0.002494 |
| exo_circ_16657 | hsa_circ_0033130 | 0.25469 | 0.940994 | 3.694662748 | 0.033669 |
| exo_circ_28491 | hsa_circ_0006446 | 0.451351 | 1.667417 | 3.694277971 | 0.004775 |
| exo_circ_61853 | hsa_circ_0073236 | 0.5542 | 2.046178 | 3.692125462 | 0.000765 |
| exo_circ_16865 | hsa_circ_0006140 | 0.416664 | 1.538211 | 3.691731203 | 0.006452 |
| exo_circ_20594 | hsa_circ_0007275 | 0.290148 | 1.070928 | 3.690969379 | 0.017548 |
| exo_circ_20351 | hsa_circ_0038076 | 0.448493 | 1.653943 | 3.687774778 | 0.001418 |
| exo_circ_30322 | hsa_circ_0010029 | 0.929202 | 3.425927 | 3.686956181 | 3.32E-06 |
| exo_circ_01341 | hsa_circ_0017359 | 0.24727 | 0.908652 | 3.674729067 | 0.03747 |
| exo_circ_09575 | hsa_circ_0008445 | 0.563111 | 2.064333 | 3.665941055 | 0.001431 |
| exo_circ_11259 | hsa_circ_0007621 | 0.604552 | 2.216076 | 3.665647922 | 0.000629 |
| exo_circ_62134 | hsa_circ_0006639 | 0.774625 | 2.838602 | 3.66448484 | 1.41E-05 |
| exo_circ_64295 | hsa_circ_0075740 | 0.390001 | 1.427309 | 3.659752589 | 0.007899 |
| exo_circ_33518 | hsa_circ_0007120 | 0.341313 | 1.247918 | 3.656230125 | 0.014336 |
| exo_circ_28177 | hsa_circ_0050063 | 0.28472 | 1.040153 | 3.653251086 | 0.021421 |
| exo_circ_36568 | hsa_circ_0009590 | 0.249614 | 0.910746 | 3.648617708 | 0.039271 |
| exo_circ_08740 | hsa_circ_0029311 | 0.45193 | 1.648502 | 3.647696382 | 0.004824 |
| exo_circ_62726 | hsa_circ_0077712 | 0.638343 | 2.328067 | 3.647048627 | 0.00055 |
| exo_circ_29899 | hsa_circ_0000017 | 0.223645 | 0.815517 | 3.646480822 | 0.041344 |
| exo_circ_06570 | hsa_circ_0001981 | 0.283827 | 1.034283 | 3.644064037 | 0.015456 |
| exo_circ_39463 | hsa_circ_0061757 | 0.384509 | 1.40089 | 3.643324847 | 0.015851 |
| exo_circ_32313 | hsa_circ_0000169 | 0.44785 | 1.631388 | 3.642709758 | 0.005932 |
| exo_circ_19480 | hsa_circ_0002255 | 0.323483 | 1.178258 | 3.642406871 | 0.010175 |
| exo_circ_72133 | hsa_circ_0083953 | 0.288968 | 1.050837 | 3.636518199 | 0.019099 |
| exo_circ_34657 | hsa_circ_0003446 | 0.369877 | 1.344715 | 3.635572055 | 0.011363 |
| exo_circ_68238 | hsa_circ_0005860 | 0.314885 | 1.144666 | 3.635187291 | 0.015065 |
| exo_circ_29896 | hsa_circ_0000105 | 0.939242 | 3.414088 | 3.63493805 | 1.69E-05 |
| exo_circ_05392 | hsa_circ_0021570 | 0.670923 | 2.437789 | 3.63348393 | 0.000368 |
| exo_circ_05005 | hsa_circ_0000277 | 0.779469 | 2.831805 | 3.632994617 | 9.92E-05 |
| exo_circ_04747 | hsa_circ_0024648 | 0.614939 | 2.230466 | 3.627133706 | 0.000918 |
| exo_circ_73798 | hsa_circ_0087859 | 0.297247 | 1.077666 | 3.625491498 | 0.022007 |
| exo_circ_63151 | hsa_circ_0004712 | 0.288802 | 1.045556 | 3.6203206 | 0.025091 |
| exo_circ_74539 | hsa_circ_0006984 | 0.742111 | 2.685785 | 3.619114718 | 5.02E-05 |
| exo_circ_27938 | hsa_circ_0000894 | 0.5642 | 2.03845 | 3.612993715 | 0.001517 |
| exo_circ_39860 | hsa_circ_0062647 | 0.361307 | 1.302925 | 3.606140039 | 0.008675 |
| exo_circ_53855 | hsa_circ_0007540 | 0.264241 | 0.95263 | 3.605156156 | 0.015283 |
| exo_circ_20978 | hsa_circ_0037658 | 0.942266 | 3.391863 | 3.59968867 | 5.31E-06 |
| exo_circ_26084 | hsa_circ_0007706 | 0.563939 | 2.029996 | 3.599671331 | 0.001516 |
| exo_circ_04328 | hsa_circ_0024233 | 0.388698 | 1.396342 | 3.592361713 | 0.009446 |
| exo_circ_01225 | hsa_circ_0007671 | 0.39015 | 1.399858 | 3.588000989 | 0.006292 |
| exo_circ_42766 | hsa_circ_0057144 | 0.576684 | 2.06615 | 3.582810826 | 0.001138 |
| exo_circ_72628 | hsa_circ_0007920 | 0.713721 | 2.556785 | 3.582333011 | 0.000164 |
| exo_circ_02478 | hsa_circ_0003755 | 0.554014 | 1.984258 | 3.581601708 | 0.001392 |
| exo_circ_18313 | hsa_circ_0001982 | 0.931002 | 3.328039 | 3.574684241 | 1.51E-05 |
| exo_circ_15897 | hsa_circ_0002289 | 0.376245 | 1.3445 | 3.573468718 | 0.011293 |
| exo_circ_43194 | hsa_circ_0008961 | 0.346563 | 1.238112 | 3.572543627 | 0.002365 |
| exo_circ_09230 | hsa_circ_0025638 | 0.266869 | 0.953348 | 3.572341186 | 0.038356 |
| exo_circ_45779 | hsa_circ_0054055 | 0.269235 | 0.961368 | 3.570736387 | 0.024568 |
| exo_circ_69433 | hsa_circ_0003943 | 0.538848 | 1.924062 | 3.570696324 | 0.00207 |
| exo_circ_00448 | hsa_circ_0017469 | 0.198189 | 0.70667 | 3.565633624 | 0.046998 |
| exo_circ_41320 | hsa_circ_0008278 | 0.457479 | 1.63119 | 3.565608297 | 0.008471 |
| exo_circ_07087 | hsa_circ_0004099 | 0.332534 | 1.185608 | 3.565369432 | 0.017095 |
| exo_circ_59792 | hsa_circ_0007611 | 0.284344 | 1.013389 | 3.56395893 | 0.033004 |
| exo_circ_63727 | hsa_circ_0078299 | 0.408425 | 1.455329 | 3.563270578 | 0.004802 |
| exo_circ_59609 | hsa_circ_0075000 | 0.610986 | 2.176649 | 3.562517475 | 0.000635 |
| exo_circ_49613 | hsa_circ_0003502 | 0.732228 | 2.605858 | 3.558808128 | 0.000243 |
| exo_circ_39409 | hsa_circ_0001192 | 0.864469 | 3.076045 | 3.558306053 | 6.23E-05 |
| exo_circ_21571 | hsa_circ_0039933 | 0.305271 | 1.085582 | 3.55612491 | 0.023802 |
| exo_circ_70397 | hsa_circ_0081343 | 0.239544 | 0.851761 | 3.555757321 | 0.041794 |
| exo_circ_43284 | hsa_circ_0057582 | 1.571196 | 5.577868 | 3.550077913 | 5.85E-09 |
| exo_circ_53182 | hsa_circ_0005688 | 0.581114 | 2.059458 | 3.543981601 | 0.001567 |
| exo_circ_58559 | hsa_circ_0003319 | 0.386821 | 1.370554 | 3.543122167 | 0.013629 |
| exo_circ_11040 | hsa_circ_0002419 | 0.420997 | 1.491443 | 3.54264337 | 0.006294 |
| exo_circ_09745 | hsa_circ_0025887 | 0.630607 | 2.230028 | 3.536321386 | 0.000888 |
| exo_circ_23802 | hsa_circ_0003530 | 0.267259 | 0.94476 | 3.534996696 | 0.015419 |
| exo_circ_43492 | hsa_circ_0007530 | 0.396642 | 1.400339 | 3.530481733 | 0.012355 |
| exo_circ_74105 | hsa_circ_0088284 | 0.244097 | 0.860409 | 3.524868176 | 0.032674 |
| exo_circ_31835 | hsa_circ_0015475 | 0.287792 | 1.014163 | 3.523946585 | 0.030017 |
| exo_circ_18334 | hsa_circ_0035505 | 0.26434 | 0.931343 | 3.523272886 | 0.042961 |
| exo_circ_64341 | hsa_circ_0001581 | 0.636996 | 2.243014 | 3.521237339 | 0.000784 |
| exo_circ_49382 | hsa_circ_0008304 | 0.447001 | 1.569942 | 3.512169357 | 0.002847 |
| exo_circ_30225 | hsa_circ_0013755 | 0.35004 | 1.229342 | 3.51200897 | 0.019943 |
| exo_circ_24294 | hsa_circ_0041638 | 1.039539 | 3.649977 | 3.511149339 | 1.5E-05 |
| exo_circ_78910 | hsa_circ_0091187 | 0.547684 | 1.922846 | 3.510867975 | 0.003058 |
| exo_circ_39144 | hsa_circ_0008725 | 0.629859 | 2.210085 | 3.508854414 | 0.001158 |
| exo_circ_43891 | hsa_circ_0008400 | 0.272683 | 0.956241 | 3.506780148 | 0.03482 |
| exo_circ_21575 | hsa_circ_0039935 | 0.238223 | 0.835144 | 3.505723523 | 0.03571 |
| exo_circ_62765 | hsa_circ_0077736 | 1.178951 | 4.124943 | 3.498823613 | 1.02E-06 |
| exo_circ_75901 | hsa_circ_0002874 | 0.387013 | 1.35399 | 3.498560231 | 0.012912 |
| exo_circ_75680 | hsa_circ_0002702 | 0.462339 | 1.617118 | 3.497688789 | 0.004548 |
| exo_circ_36948 | hsa_circ_0003310 | 0.820866 | 2.870553 | 3.496980496 | 0.000193 |
| exo_circ_41444 | hsa_circ_0056402 | 0.401383 | 1.403525 | 3.49672205 | 0.009871 |
| exo_circ_23245 | hsa_circ_0003586 | 0.81714 | 2.855923 | 3.495022039 | 0.000148 |
| exo_circ_21582 | hsa_circ_0005623 | 0.3237 | 1.131251 | 3.494753021 | 0.02463 |
| exo_circ_28547 | hsa_circ_0050532 | 0.2886 | 1.008 | 3.49272549 | 0.036743 |
| exo_circ_74929 | hsa_circ_0009011 | 0.305246 | 1.065069 | 3.489219571 | 0.029416 |
| exo_circ_68839 | hsa_circ_0079929 | 1.078533 | 3.761067 | 3.487206934 | 1.24E-05 |
| exo_circ_21503 | hsa_circ_0000707 | 0.414688 | 1.444959 | 3.484451939 | 0.005354 |
| exo_circ_26022 | hsa_circ_0047021 | 0.38309 | 1.334498 | 3.483512764 | 0.010758 |
| exo_circ_26245 | hsa_circ_0000837 | 0.421145 | 1.466362 | 3.481842961 | 0.009002 |
| exo_circ_15426 | hsa_circ_0006393 | 0.317393 | 1.104864 | 3.481059473 | 0.020529 |
| exo_circ_40666 | hsa_circ_0001250 | 0.831089 | 2.892921 | 3.480879409 | 0.00022 |
| exo_circ_75726 | hsa_circ_0087014 | 0.35292 | 1.227311 | 3.477585894 | 0.01907 |
| exo_circ_30920 | hsa_circ_0005758 | 0.321729 | 1.118343 | 3.476035809 | 0.029115 |
| exo_circ_51628 | hsa_circ_0006215 | 0.690376 | 2.398897 | 3.474769756 | 0.000765 |
| exo_circ_30707 | hsa_circ_0005423 | 0.404384 | 1.404547 | 3.473303089 | 0.012131 |
| exo_circ_06229 | hsa_circ_0004168 | 0.264365 | 0.917539 | 3.470731775 | 0.033966 |
| exo_circ_28994 | hsa_circ_0051680 | 0.467833 | 1.623255 | 3.46973122 | 0.007634 |
| exo_circ_04573 | hsa_circ_0024444 | 0.363213 | 1.259575 | 3.467867922 | 0.016237 |
| exo_circ_36243 | hsa_circ_0002231 | 0.748382 | 2.594601 | 3.466945442 | 0.000188 |
| exo_circ_42397 | hsa_circ_0003120 | 0.444655 | 1.540521 | 3.464531403 | 0.006898 |
| exo_circ_62210 | hsa_circ_0007003 | 0.671523 | 2.324711 | 3.461851474 | 0.001011 |
| exo_circ_15436 | hsa_circ_0032135 | 0.254444 | 0.880524 | 3.460579576 | 0.046764 |
| exo_circ_42844 | hsa_circ_0005490 | 0.295692 | 1.020572 | 3.451463809 | 0.026336 |
| exo_circ_62349 | hsa_circ_0005932 | 0.528406 | 1.823695 | 3.451312525 | 0.002445 |
| exo_circ_44269 | hsa_circ_0004821 | 0.677268 | 2.337137 | 3.450829415 | 0.000742 |
| exo_circ_20887 | hsa_circ_0039076 | 0.446498 | 1.540247 | 3.4496153 | 0.008231 |
| exo_circ_40982 | hsa_circ_0002076 | 0.545117 | 1.879101 | 3.447152613 | 0.001577 |
| exo_circ_71780 | hsa_circ_0006320 | 1.227767 | 4.226134 | 3.442129774 | 2.52E-06 |
| exo_circ_36598 | hsa_circ_0009596 | 0.368972 | 1.267213 | 3.434438431 | 0.014822 |
| exo_circ_66389 | hsa_circ_0081739 | 0.559998 | 1.920429 | 3.429349578 | 0.002552 |
| exo_circ_16300 | hsa_circ_0005987 | 1.41439 | 4.847615 | 3.427352608 | 2.08E-07 |
| exo_circ_59076 | hsa_circ_0074441 | 0.2701 | 0.924813 | 3.423960076 | 0.046725 |
| exo_circ_58081 | hsa_circ_0004629 | 0.374079 | 1.280221 | 3.422324117 | 0.02235 |
| exo_circ_34602 | hsa_circ_0002452 | 0.291272 | 0.996024 | 3.419564458 | 0.035847 |
| exo_circ_74885 | hsa_circ_0089489 | 0.72345 | 2.471285 | 3.415972999 | 0.000352 |
| exo_circ_05174 | hsa_circ_0000279 | 1.162395 | 3.959954 | 3.406719486 | 5.04E-06 |
| exo_circ_75472 | hsa_circ_0004389 | 0.323043 | 1.098868 | 3.401614311 | 0.032139 |
| exo_circ_65183 | hsa_circ_0001610 | 0.450051 | 1.529682 | 3.39891295 | 0.006377 |
| exo_circ_64209 | hsa_circ_0004342 | 0.543266 | 1.845309 | 3.396695672 | 0.005291 |
| exo_circ_74234 | hsa_circ_0003250 | 1.272708 | 4.321258 | 3.395326463 | 5.17E-07 |
| exo_circ_08470 | hsa_circ_0005465 | 0.871983 | 2.959503 | 3.393991558 | 8E-05 |
| exo_circ_04219 | hsa_circ_0005580 | 0.725237 | 2.460108 | 3.392141794 | 0.000946 |
| exo_circ_45482 | hsa_circ_0053786 | 0.371479 | 1.259852 | 3.391447204 | 0.017154 |
| exo_circ_26092 | hsa_circ_0047056 | 0.255212 | 0.865092 | 3.389702623 | 0.022903 |
| exo_circ_63154 | hsa_circ_0002198 | 0.627352 | 2.123189 | 3.384365105 | 0.001475 |
| exo_circ_56928 | hsa_circ_0002048 | 0.288318 | 0.975357 | 3.382924194 | 0.03193 |
| exo_circ_30277 | hsa_circ_0008275 | 0.411943 | 1.393334 | 3.382344199 | 0.015997 |
| exo_circ_78497 | hsa_circ_0002724 | 0.29233 | 0.987608 | 3.378401079 | 0.039541 |
| exo_circ_77705 | hsa_circ_0004549 | 0.672433 | 2.269564 | 3.375153917 | 0.000658 |
| exo_circ_24738 | hsa_circ_0003127 | 0.275316 | 0.928106 | 3.371056801 | 0.044257 |
| exo_circ_73217 | hsa_circ_0003175 | 0.291942 | 0.982892 | 3.36673595 | 0.035937 |
| exo_circ_62721 | hsa_circ_0077711 | 0.445477 | 1.499205 | 3.365391124 | 0.010447 |
| exo_circ_40976 | hsa_circ_0003523 | 0.480492 | 1.616059 | 3.363344129 | 0.010288 |
| exo_circ_34787 | hsa_circ_0002043 | 0.485297 | 1.630483 | 3.359764417 | 0.007622 |
| exo_circ_06777 | hsa_circ_0023748 | 0.29928 | 1.004419 | 3.356114082 | 0.034279 |
| exo_circ_25751 | hsa_circ_0000814 | 0.342206 | 1.147874 | 3.354331997 | 0.025198 |
| exo_circ_06936 | hsa_circ_0023918 | 1.210892 | 4.061097 | 3.353807117 | 6.12E-06 |
| exo_circ_46013 | hsa_circ_0054226 | 1.017212 | 3.409081 | 3.351396242 | 3.63E-05 |
| exo_circ_21834 | hsa_circ_0040400 | 0.522978 | 1.751979 | 3.350006989 | 0.004798 |
| exo_circ_71467 | hsa_circ_0007786 | 0.323632 | 1.084043 | 3.349614824 | 0.016878 |
| exo_circ_11188 | hsa_circ_0027689 | 0.229813 | 0.768916 | 3.345832432 | 0.048717 |
| exo_circ_07200 | hsa_circ_0024034 | 0.377925 | 1.26233 | 3.340161456 | 0.019261 |
| exo_circ_75417 | hsa_circ_0086188 | 0.895798 | 2.991126 | 3.339064732 | 0.000105 |
| exo_circ_77194 | hsa_circ_0002153 | 0.406324 | 1.354922 | 3.334587126 | 0.011758 |
| exo_circ_12019 | hsa_circ_0029705 | 0.41201 | 1.373567 | 3.333817472 | 0.018893 |
| exo_circ_30364 | hsa_circ_0007755 | 1.559744 | 5.18502 | 3.324275591 | 2.91E-08 |
| exo_circ_40685 | hsa_circ_0001252 | 0.485867 | 1.61461 | 3.323154298 | 0.006277 |
| exo_circ_05755 | hsa_circ_0021902 | 0.672357 | 2.231495 | 3.318916638 | 0.00176 |
| exo_circ_36983 | hsa_circ_0005720 | 0.897848 | 2.97725 | 3.315983886 | 0.000143 |
| exo_circ_35784 | hsa_circ_0006281 | 0.429298 | 1.423119 | 3.314990349 | 0.0129 |
| exo_circ_03734 | hsa_circ_0019172 | 0.681501 | 2.259101 | 3.314888409 | 0.002194 |
| exo_circ_48823 | hsa_circ_0007841 | 0.301453 | 0.99772 | 3.309703383 | 0.046512 |
| exo_circ_24572 | hsa_circ_0044709 | 0.499355 | 1.651933 | 3.30813377 | 0.00371 |
| exo_circ_29370 | hsa_circ_0000883 | 0.326082 | 1.07844 | 3.307266944 | 0.031972 |
| exo_circ_27808 | hsa_circ_0002438 | 0.426719 | 1.409191 | 3.302389978 | 0.014921 |
| exo_circ_44433 | hsa_circ_0003273 | 0.90342 | 2.982798 | 3.301672474 | 5.26E-05 |
| exo_circ_46068 | hsa_circ_0003990 | 0.469336 | 1.549552 | 3.301581947 | 0.004971 |
| exo_circ_40721 | hsa_circ_0001258 | 2.212121 | 7.299014 | 3.299554503 | 1.28E-12 |
| exo_circ_31901 | hsa_circ_0005909 | 0.322841 | 1.06445 | 3.297135307 | 0.03136 |
| exo_circ_56287 | hsa_circ_0004124 | 0.750783 | 2.472389 | 3.293080678 | 0.000994 |
| exo_circ_16029 | hsa_circ_0000557 | 0.629021 | 2.070677 | 3.291904609 | 0.000773 |
| exo_circ_61411 | hsa_circ_0004405 | 1.213171 | 3.993067 | 3.291428454 | 4.27E-06 |
| exo_circ_51241 | hsa_circ_0068648 | 0.351809 | 1.156819 | 3.288197351 | 0.032544 |
| exo_circ_09998 | hsa_circ_0006222 | 0.298313 | 0.980617 | 3.287203162 | 0.039382 |
| exo_circ_16603 | hsa_circ_0000563 | 0.356316 | 1.170008 | 3.283627938 | 0.033221 |
| exo_circ_12290 | hsa_circ_0002635 | 0.627041 | 2.058201 | 3.282401317 | 0.003926 |
| exo_circ_29474 | hsa_circ_0049072 | 0.535954 | 1.759153 | 3.28228427 | 0.005107 |
| exo_circ_12623 | hsa_circ_0000478 | 1.179734 | 3.872047 | 3.282137299 | 5.75E-06 |
| exo_circ_56013 | hsa_circ_0069402 | 0.388101 | 1.27277 | 3.279484775 | 0.021283 |
| exo_circ_21005 | hsa_circ_0007637 | 1.997851 | 6.551147 | 3.279096772 | 1.31E-11 |
| exo_circ_55202 | hsa_circ_0007121 | 0.472927 | 1.550088 | 3.277644708 | 0.013806 |
| exo_circ_36257 | hsa_circ_0003566 | 0.528728 | 1.732048 | 3.275879094 | 0.005482 |
| exo_circ_57040 | hsa_circ_0069982 | 0.350108 | 1.146188 | 3.273817432 | 0.003309 |
| exo_circ_07529 | hsa_circ_0006260 | 0.555816 | 1.818074 | 3.270999124 | 0.008979 |
| exo_circ_34849 | hsa_circ_0011625 | 0.312241 | 1.020808 | 3.269298348 | 0.047234 |
| exo_circ_43049 | hsa_circ_0004328 | 0.326805 | 1.068369 | 3.269130892 | 0.028573 |
| exo_circ_13976 | hsa_circ_0002928 | 1.256184 | 4.09698 | 3.261449379 | 6.54E-06 |
| exo_circ_40540 | hsa_circ_0008561 | 0.402028 | 1.310918 | 3.260763711 | 0.019604 |
| exo_circ_56609 | hsa_circ_0069725 | 0.423371 | 1.379537 | 3.258455639 | 0.020396 |
| exo_circ_55569 | hsa_circ_0071519 | 0.33979 | 1.105274 | 3.252819666 | 0.034133 |
| exo_circ_31870 | hsa_circ_0009175 | 0.733663 | 2.385694 | 3.251758445 | 0.000858 |
| exo_circ_08135 | hsa_circ_0008151 | 0.777723 | 2.527382 | 3.24971971 | 0.000593 |
| exo_circ_24571 | hsa_circ_0044708 | 0.943885 | 3.066581 | 3.248891888 | 6.47E-05 |
| exo_circ_33565 | hsa_circ_0004531 | 0.666262 | 2.164234 | 3.248321898 | 0.00269 |
| exo_circ_30360 | hsa_circ_0002679 | 0.351949 | 1.142344 | 3.245768689 | 0.022081 |
| exo_circ_04293 | hsa_circ_0007694 | 0.957448 | 3.106068 | 3.244111304 | 2.75E-05 |
| exo_circ_78061 | hsa_circ_0005574 | 0.473572 | 1.535991 | 3.243412768 | 0.010701 |
| exo_circ_71666 | hsa_circ_0005132 | 0.687316 | 2.229091 | 3.243180977 | 0.002513 |
| exo_circ_60165 | hsa_circ_0001472 | 1.526793 | 4.949163 | 3.241542465 | 1.28E-07 |
| exo_circ_21478 | hsa_circ_0008242 | 0.612826 | 1.986365 | 3.241319306 | 0.003 |
| exo_circ_37886 | hsa_circ_0060219 | 0.382855 | 1.240786 | 3.240879745 | 0.021634 |
| exo_circ_09427 | hsa_circ_0006584 | 0.425353 | 1.37804 | 3.239753115 | 0.014116 |
| exo_circ_61800 | hsa_circ_0008315 | 0.365278 | 1.182285 | 3.236671737 | 0.027337 |
| exo_circ_74965 | hsa_circ_0009134 | 0.621284 | 2.010826 | 3.236562882 | 0.001481 |
| exo_circ_76468 | hsa_circ_0008510 | 0.456259 | 1.475872 | 3.234728124 | 0.009823 |
| exo_circ_77910 | hsa_circ_0001952 | 0.58784 | 1.900574 | 3.233148382 | 0.004442 |
| exo_circ_33146 | hsa_circ_0005576 | 0.850282 | 2.748802 | 3.232810854 | 7.83E-05 |
| exo_circ_75252 | hsa_circ_0086492 | 1.414407 | 4.56979 | 3.230888197 | 2.03E-07 |
| exo_circ_68660 | hsa_circ_0079812 | 1.002909 | 3.233778 | 3.224397535 | 9E-05 |
| exo_circ_35795 | hsa_circ_0000073 | 0.614047 | 1.979062 | 3.222983234 | 0.003316 |
| exo_circ_22904 | hsa_circ_0003481 | 0.374678 | 1.20601 | 3.218789838 | 0.027315 |
| exo_circ_42041 | hsa_circ_0052728 | 0.306027 | 0.984176 | 3.215980691 | 0.036016 |
| exo_circ_04327 | hsa_circ_0005612 | 0.443928 | 1.427155 | 3.214833945 | 0.018295 |
| exo_circ_30616 | hsa_circ_0002394 | 0.871152 | 2.794361 | 3.207662994 | 0.000369 |
| exo_circ_24052 | hsa_circ_0005455 | 2.292857 | 7.353032 | 3.206929482 | 3.65E-12 |
| exo_circ_63517 | hsa_circ_0006713 | 0.612994 | 1.96366 | 3.203389755 | 0.004555 |
| exo_circ_52826 | hsa_circ_0002554 | 0.463534 | 1.48344 | 3.200280288 | 0.013717 |
| exo_circ_23308 | hsa_circ_0042987 | 0.423816 | 1.355884 | 3.199229418 | 0.023189 |
| exo_circ_20942 | hsa_circ_0005838 | 1.3623 | 4.357528 | 3.198655638 | 3.79E-06 |
| exo_circ_31790 | hsa_circ_0007700 | 0.370952 | 1.185341 | 3.195401344 | 0.019344 |
| exo_circ_06000 | hsa_circ_0004988 | 0.418338 | 1.336361 | 3.194455741 | 0.018442 |
| exo_circ_79068 | hsa_circ_0007907 | 0.975276 | 3.112531 | 3.191438179 | 0.000308 |
| exo_circ_51405 | hsa_circ_0068738 | 0.536158 | 1.710802 | 3.190856428 | 0.005993 |
| exo_circ_23035 | hsa_circ_0004800 | 0.755656 | 2.408029 | 3.186673059 | 0.001356 |
| exo_circ_21025 | hsa_circ_0007788 | 1.224644 | 3.897851 | 3.1828447 | 4.13E-06 |
| exo_circ_29569 | hsa_circ_0009682 | 0.71441 | 2.270636 | 3.178337103 | 0.001492 |
| exo_circ_57516 | hsa_circ_0007948 | 0.573868 | 1.8237 | 3.177907946 | 0.008166 |
| exo_circ_55560 | hsa_circ_0071510 | 0.475328 | 1.510358 | 3.177504075 | 0.011474 |
| exo_circ_18931 | hsa_circ_0008378 | 1.741115 | 5.531988 | 3.177267502 | 8.56E-08 |
| exo_circ_23756 | hsa_circ_0041501 | 0.377207 | 1.197022 | 3.173383074 | 0.031327 |
| exo_circ_29632 | hsa_circ_0005199 | 1.125912 | 3.571304 | 3.171921428 | 8.87E-05 |
| exo_circ_11273 | hsa_circ_0024986 | 0.379377 | 1.202662 | 3.170094858 | 0.025029 |
| exo_circ_43247 | hsa_circ_0057550 | 0.635628 | 2.013547 | 3.167808857 | 0.003009 |
| exo_circ_21231 | hsa_circ_0039346 | 0.374427 | 1.185857 | 3.167126818 | 0.027176 |
| exo_circ_43676 | hsa_circ_0003747 | 0.413316 | 1.30874 | 3.166442063 | 0.020555 |
| exo_circ_24818 | hsa_circ_0044956 | 0.377106 | 1.193374 | 3.164555226 | 0.033205 |
| exo_circ_10105 | hsa_circ_0003248 | 0.58268 | 1.843147 | 3.163222096 | 0.000805 |
| exo_circ_28063 | hsa_circ_0004518 | 0.407078 | 1.286814 | 3.161101852 | 0.011977 |
| exo_circ_18885 | hsa_circ_0000620 | 1.271922 | 4.020326 | 3.160827884 | 6.36E-06 |
| exo_circ_19936 | hsa_circ_0000659 | 1.173962 | 3.709345 | 3.159681004 | 2.66E-05 |
| exo_circ_72632 | hsa_circ_0084552 | 0.530104 | 1.674021 | 3.157909199 | 0.008494 |
| exo_circ_33960 | hsa_circ_0007543 | 0.365414 | 1.153816 | 3.157556351 | 0.039695 |
| exo_circ_30992 | hsa_circ_0002733 | 0.893525 | 2.818284 | 3.154117296 | 0.000286 |
| exo_circ_70339 | hsa_circ_0001725 | 0.804977 | 2.535153 | 3.149349079 | 0.000687 |
| exo_circ_02842 | hsa_circ_0003459 | 0.712398 | 2.243292 | 3.14893038 | 0.002072 |
| exo_circ_15908 | hsa_circ_0008406 | 0.394448 | 1.240703 | 3.145413326 | 0.024359 |
| exo_circ_65841 | hsa_circ_0008236 | 0.340476 | 1.070921 | 3.14536282 | 0.034915 |
| exo_circ_35965 | hsa_circ_0000079 | 0.316692 | 0.995712 | 3.14409791 | 0.028501 |
| exo_circ_25677 | hsa_circ_0008677 | 0.393456 | 1.236062 | 3.141550149 | 0.033486 |
| exo_circ_21532 | hsa_circ_0000710 | 0.439989 | 1.382124 | 3.141267673 | 0.015908 |
| exo_circ_61856 | hsa_circ_0073239 | 0.993326 | 3.116395 | 3.137332199 | 0.000119 |
| exo_circ_06091 | hsa_circ_0022601 | 0.401122 | 1.25793 | 3.136024843 | 0.031546 |
| exo_circ_38740 | hsa_circ_0006458 | 1.295025 | 4.056712 | 3.132534879 | 9.49E-06 |
| exo_circ_69546 | hsa_circ_0005588 | 1.316261 | 4.12315 | 3.1324717 | 8.78E-06 |
| exo_circ_44092 | hsa_circ_0058050 | 0.399353 | 1.250596 | 3.131553747 | 0.033629 |
| exo_circ_58836 | hsa_circ_0007452 | 0.627367 | 1.963939 | 3.130447599 | 0.004869 |
| exo_circ_37833 | hsa_circ_0008695 | 0.424281 | 1.327123 | 3.127932156 | 0.015352 |
| exo_circ_41043 | hsa_circ_0056121 | 0.914264 | 2.859386 | 3.127525791 | 0.000629 |
| exo_circ_33674 | hsa_circ_0017041 | 0.605587 | 1.891742 | 3.123814623 | 0.006416 |
| exo_circ_63661 | hsa_circ_0001651 | 0.630796 | 1.96985 | 3.122799842 | 0.001332 |
| exo_circ_58254 | hsa_circ_0005949 | 0.736024 | 2.29791 | 3.122059805 | 0.001862 |
| exo_circ_59468 | hsa_circ_0074854 | 0.716794 | 2.237293 | 3.121251895 | 0.002661 |
| exo_circ_01154 | hsa_circ_0004281 | 0.929822 | 2.900773 | 3.119707732 | 0.000367 |
| exo_circ_47998 | hsa_circ_0005605 | 1.050162 | 3.273497 | 3.117136368 | 0.000251 |
| exo_circ_42914 | hsa_circ_0002087 | 0.374619 | 1.167218 | 3.115744416 | 0.029415 |
| exo_circ_00303 | hsa_circ_0000206 | 1.007379 | 3.137122 | 3.11414425 | 0.000195 |
| exo_circ_22055 | hsa_circ_0000721 | 0.604328 | 1.880525 | 3.111764052 | 0.004124 |
| exo_circ_50667 | hsa_circ_0001367 | 1.002023 | 3.112898 | 3.106613557 | 0.000203 |
| exo_circ_20078 | hsa_circ_0000672 | 1.672323 | 5.189608 | 3.103233074 | 1.13E-07 |
| exo_circ_44947 | hsa_circ_0004924 | 0.679666 | 2.108146 | 3.101738252 | 0.002564 |
| exo_circ_43021 | hsa_circ_0057276 | 0.319945 | 0.991849 | 3.100063884 | 0.046256 |
| exo_circ_61684 | hsa_circ_0001504 | 1.114357 | 3.448626 | 3.094722583 | 3.2E-05 |
| exo_circ_37672 | hsa_circ_0003073 | 0.750715 | 2.322301 | 3.093451832 | 0.001776 |
| exo_circ_25794 | hsa_circ_0008041 | 0.700081 | 2.16148 | 3.087471259 | 0.00329 |
| exo_circ_06677 | hsa_circ_0023659 | 0.826638 | 2.552031 | 3.087240931 | 0.001071 |
| exo_circ_09491 | hsa_circ_0025763 | 0.446893 | 1.378517 | 3.084671151 | 0.027235 |
| exo_circ_74744 | hsa_circ_0001895 | 1.100594 | 3.394733 | 3.084455954 | 5.11E-05 |
| exo_circ_70287 | hsa_circ_0003974 | 0.412948 | 1.271898 | 3.080041819 | 0.028755 |
| exo_circ_38605 | hsa_circ_0008777 | 0.624247 | 1.92254 | 3.079775734 | 0.00606 |
| exo_circ_31644 | hsa_circ_0000161 | 0.667877 | 2.055572 | 3.077769046 | 0.002684 |
| exo_circ_03606 | hsa_circ_0019120 | 0.518564 | 1.596002 | 3.077734511 | 0.014636 |
| exo_circ_18934 | hsa_circ_0007088 | 0.794357 | 2.444812 | 3.0777263 | 0.000993 |
| exo_circ_26984 | hsa_circ_0006952 | 0.859682 | 2.644866 | 3.076562666 | 0.000574 |
| exo_circ_30273 | hsa_circ_0009964 | 0.868227 | 2.664602 | 3.069014537 | 0.000299 |
| exo_circ_41442 | hsa_circ_0056401 | 0.466889 | 1.430586 | 3.064082005 | 0.020026 |
| exo_circ_39891 | hsa_circ_0062654 | 0.301488 | 0.923546 | 3.063297525 | 0.033356 |
| exo_circ_13828 | hsa_circ_0004965 | 0.766155 | 2.345915 | 3.061930202 | 0.001502 |
| exo_circ_00908 | hsa_circ_0008969 | 0.403481 | 1.235337 | 3.06170004 | 0.029806 |
| exo_circ_59939 | hsa_circ_0001566 | 1.049063 | 3.211722 | 3.061514906 | 0.000183 |
| exo_circ_20789 | hsa_circ_0007807 | 0.657494 | 2.011565 | 3.059441384 | 0.003855 |
| exo_circ_42074 | hsa_circ_0008083 | 0.555146 | 1.696752 | 3.056408773 | 0.006818 |
| exo_circ_75133 | hsa_circ_0086425 | 0.50959 | 1.556909 | 3.055217651 | 0.011652 |
| exo_circ_39777 | hsa_circ_0001211 | 0.358273 | 1.094299 | 3.05437017 | 0.037532 |
| exo_circ_69556 | hsa_circ_0080543 | 0.285442 | 0.871693 | 3.053833128 | 0.035254 |
| exo_circ_55817 | hsa_circ_0068925 | 0.767318 | 2.341397 | 3.051404441 | 0.002282 |
| exo_circ_44503 | hsa_circ_0003898 | 0.377585 | 1.15179 | 3.05041024 | 0.037641 |
| exo_circ_39141 | hsa_circ_0002660 | 0.506354 | 1.542968 | 3.047214479 | 0.012171 |
| exo_circ_19479 | hsa_circ_0005558 | 0.542802 | 1.65375 | 3.046692289 | 0.006855 |
| exo_circ_29630 | hsa_circ_0013353 | 0.400186 | 1.218988 | 3.046057056 | 0.032739 |
| exo_circ_21727 | hsa_circ_0040188 | 0.816691 | 2.486123 | 3.044140282 | 0.001517 |
| exo_circ_09433 | hsa_circ_0008250 | 1.147487 | 3.492007 | 3.043177809 | 9.75E-05 |
| exo_circ_61386 | hsa_circ_0005777 | 0.751843 | 2.284052 | 3.037938598 | 0.001912 |
| exo_circ_73244 | hsa_circ_0002576 | 0.376452 | 1.142714 | 3.035486566 | 0.039656 |
| exo_circ_51175 | hsa_circ_0008756 | 0.755174 | 2.292126 | 3.035228967 | 0.001907 |
| exo_circ_62641 | hsa_circ_0077660 | 0.64249 | 1.949875 | 3.034874607 | 0.005354 |
| exo_circ_34382 | hsa_circ_0005087 | 1.204706 | 3.655055 | 3.033980243 | 6.62E-06 |
| exo_circ_10300 | hsa_circ_0002737 | 0.393318 | 1.193249 | 3.033804206 | 0.035964 |
| exo_circ_74955 | hsa_circ_0001904 | 1.649777 | 4.993084 | 3.026520772 | 4.71E-07 |
| exo_circ_52638 | hsa_circ_0007799 | 0.549982 | 1.663678 | 3.024967455 | 0.012331 |
| exo_circ_13069 | hsa_circ_0008776 | 0.479656 | 1.44794 | 3.018704993 | 0.018954 |
| exo_circ_07226 | hsa_circ_0000350 | 0.349096 | 1.053641 | 3.018195484 | 0.044337 |
| exo_circ_34435 | hsa_circ_0008057 | 0.517115 | 1.560071 | 3.016874754 | 0.013189 |
| exo_circ_20091 | hsa_circ_0037858 | 0.495768 | 1.494857 | 3.015231457 | 0.017245 |
| exo_circ_22388 | hsa_circ_0004445 | 0.764422 | 2.30433 | 3.014474817 | 0.00243 |
| exo_circ_31670 | hsa_circ_0004722 | 0.539236 | 1.625017 | 3.01355534 | 0.019383 |
| exo_circ_76333 | hsa_circ_0087255 | 0.462266 | 1.39193 | 3.011100327 | 0.017192 |
| exo_circ_04587 | hsa_circ_0007372 | 0.935218 | 2.813303 | 3.008178368 | 0.000291 |
| exo_circ_42994 | hsa_circ_0002844 | 0.365955 | 1.099267 | 3.003834235 | 0.024397 |
| exo_circ_20761 | hsa_circ_0037558 | 0.361063 | 1.084119 | 3.002579228 | 0.040864 |
| exo_circ_13835 | hsa_circ_0007737 | 0.618046 | 1.855592 | 3.002350848 | 0.006322 |
| exo_circ_75112 | hsa_circ_0086421 | 0.548335 | 1.641872 | 2.994286351 | 0.010452 |
| exo_circ_64793 | hsa_circ_0076154 | 0.487979 | 1.460949 | 2.993877525 | 0.023746 |
| exo_circ_04764 | hsa_circ_0000365 | 1.454749 | 4.352042 | 2.991610344 | 8.24E-06 |
| exo_circ_13310 | hsa_circ_0030507 | 0.601207 | 1.798036 | 2.990710554 | 0.008955 |
| exo_circ_62734 | hsa_circ_0006146 | 0.650286 | 1.944659 | 2.990465405 | 0.005962 |
| exo_circ_68343 | hsa_circ_0001687 | 0.505763 | 1.511839 | 2.989222518 | 0.013675 |
| exo_circ_38356 | hsa_circ_0003539 | 0.513899 | 1.534873 | 2.986722967 | 0.010956 |
| exo_circ_36790 | hsa_circ_0013162 | 0.474234 | 1.414778 | 2.983288836 | 0.020515 |
| exo_circ_57473 | hsa_circ_0006938 | 0.416485 | 1.241313 | 2.980451238 | 0.037029 |
| exo_circ_25187 | hsa_circ_0002879 | 0.405946 | 1.209363 | 2.979123041 | 0.036446 |
| exo_circ_44147 | hsa_circ_0058213 | 0.408844 | 1.216246 | 2.974842031 | 0.021344 |
| exo_circ_18240 | hsa_circ_0035443 | 0.497757 | 1.479847 | 2.9730323 | 0.016672 |
| exo_circ_72176 | hsa_circ_0083995 | 0.541884 | 1.61092 | 2.972814297 | 0.012152 |
| exo_circ_53186 | hsa_circ_0003060 | 1.066733 | 3.170262 | 2.97193635 | 0.000247 |
| exo_circ_25470 | hsa_circ_0006942 | 0.872015 | 2.589486 | 2.969541806 | 0.000964 |
| exo_circ_63454 | hsa_circ_0004327 | 0.61075 | 1.812694 | 2.967981183 | 0.009342 |
| exo_circ_40073 | hsa_circ_0009055 | 0.365178 | 1.083105 | 2.965962817 | 0.035918 |
| exo_circ_61938 | hsa_circ_0005476 | 0.501925 | 1.487401 | 2.963392197 | 0.029001 |
| exo_circ_31084 | hsa_circ_0003039 | 0.747567 | 2.210285 | 2.956636409 | 0.004179 |
| exo_circ_24855 | hsa_circ_0007682 | 0.470746 | 1.391694 | 2.956355286 | 0.027653 |
| exo_circ_50961 | hsa_circ_0068472 | 0.479897 | 1.418621 | 2.95609161 | 0.019103 |
| exo_circ_73461 | hsa_circ_0004762 | 0.898639 | 2.656095 | 2.955686849 | 0.002123 |
| exo_circ_17434 | hsa_circ_0002466 | 0.694341 | 2.051677 | 2.954853505 | 0.004286 |
| exo_circ_59771 | hsa_circ_0075161 | 0.511054 | 1.508685 | 2.952105281 | 0.014033 |
| exo_circ_46223 | hsa_circ_0005377 | 1.095015 | 3.2322 | 2.951739258 | 0.000136 |
| exo_circ_72861 | hsa_circ_0084669 | 0.540333 | 1.593135 | 2.948433822 | 0.015001 |
| exo_circ_36113 | hsa_circ_0008433 | 1.282115 | 3.779921 | 2.9481917 | 8.16E-05 |
| exo_circ_61006 | hsa_circ_0003830 | 1.053188 | 3.099296 | 2.942776633 | 0.00026 |
| exo_circ_37357 | hsa_circ_0003853 | 1.028986 | 3.021923 | 2.936797062 | 0.000651 |
| exo_circ_00897 | hsa_circ_0003570 | 1.029363 | 3.022653 | 2.936430139 | 0.000371 |
| exo_circ_74892 | hsa_circ_0006814 | 0.443383 | 1.301645 | 2.935709238 | 0.023776 |
| exo_circ_21577 | hsa_circ_0000713 | 0.627049 | 1.840779 | 2.935622091 | 0.003389 |
| exo_circ_27521 | hsa_circ_0048000 | 0.50663 | 1.485071 | 2.93127312 | 0.021997 |
| exo_circ_06949 | hsa_circ_0023928 | 0.755753 | 2.215213 | 2.931132196 | 0.004288 |
| exo_circ_60325 | hsa_circ_0001475 | 0.717266 | 2.102325 | 2.931025196 | 0.005126 |
| exo_circ_62279 | hsa_circ_0073486 | 0.712985 | 2.088806 | 2.92966332 | 0.003306 |
| exo_circ_19762 | hsa_circ_0007201 | 1.908689 | 5.588137 | 2.927735843 | 8.21E-07 |
| exo_circ_09609 | hsa_circ_0025839 | 1.227279 | 3.592309 | 2.927052624 | 6.76E-05 |
| exo_circ_29654 | hsa_circ_0009701 | 0.437962 | 1.280915 | 2.924715805 | 0.031637 |
| exo_circ_30538 | hsa_circ_0000126 | 0.490915 | 1.435507 | 2.924145997 | 0.008085 |
| exo_circ_21355 | hsa_circ_0039398 | 0.419813 | 1.226861 | 2.922402637 | 0.028455 |
| exo_circ_44531 | hsa_circ_0007321 | 0.855748 | 2.498303 | 2.919438331 | 0.002228 |
| exo_circ_30953 | hsa_circ_0014717 | 0.251926 | 0.735457 | 2.919344488 | 0.048754 |
| exo_circ_44296 | hsa_circ_0058463 | 0.488096 | 1.424539 | 2.918561935 | 0.030439 |
| exo_circ_56258 | hsa_circ_0008453 | 1.473856 | 4.299669 | 2.917292764 | 1.42E-05 |
| exo_circ_06867 | hsa_circ_0021089 | 0.511159 | 1.489216 | 2.913410876 | 0.027006 |
| exo_circ_26920 | hsa_circ_0047650 | 0.819748 | 2.387765 | 2.91280423 | 0.003183 |
| exo_circ_78434 | hsa_circ_0008983 | 0.795404 | 2.315668 | 2.911309047 | 0.002082 |
| exo_circ_16154 | hsa_circ_0032822 | 1.266525 | 3.686238 | 2.910513111 | 6.1E-05 |
| exo_circ_72454 | hsa_circ_0002003 | 0.596364 | 1.735248 | 2.909712858 | 0.018479 |
| exo_circ_32582 | hsa_circ_0016213 | 0.418812 | 1.21789 | 2.907964215 | 0.04937 |
| exo_circ_70982 | hsa_circ_0085460 | 0.429178 | 1.247144 | 2.905888949 | 0.028573 |
| exo_circ_32497 | hsa_circ_0016105 | 0.632904 | 1.838807 | 2.905348561 | 0.011946 |
| exo_circ_30600 | hsa_circ_0002520 | 0.499805 | 1.450738 | 2.902610793 | 0.018528 |
| exo_circ_01859 | hsa_circ_0004126 | 0.658663 | 1.911075 | 2.901446163 | 0.007539 |
| exo_circ_07231 | hsa_circ_0008648 | 0.630071 | 1.82422 | 2.89526087 | 0.00586 |
| exo_circ_01647 | hsa_circ_0018007 | 0.699957 | 2.024012 | 2.891621907 | 0.006404 |
| exo_circ_09772 | hsa_circ_0000392 | 1.310427 | 3.787327 | 2.890146169 | 3.61E-05 |
| exo_circ_39532 | hsa_circ_0008021 | 0.555963 | 1.605623 | 2.888003999 | 0.019461 |
| exo_circ_34871 | hsa_circ_0011644 | 0.565038 | 1.631066 | 2.886648655 | 0.013755 |
| exo_circ_52257 | hsa_circ_0065052 | 0.924689 | 2.667663 | 2.884929827 | 0.001062 |
| exo_circ_53926 | hsa_circ_0070635 | 0.448846 | 1.294357 | 2.883746642 | 0.034633 |
| exo_circ_52610 | hsa_circ_0065774 | 0.534218 | 1.538835 | 2.880537526 | 0.017772 |
| exo_circ_35025 | hsa_circ_0005788 | 0.551477 | 1.588043 | 2.879618078 | 0.017643 |
| exo_circ_63299 | hsa_circ_0078019 | 0.456684 | 1.314949 | 2.879337347 | 0.033785 |
| exo_circ_35644 | hsa_circ_0003632 | 1.49334 | 4.299538 | 2.879141803 | 1.75E-05 |
| exo_circ_29467 | hsa_circ_0049069 | 0.675375 | 1.939942 | 2.872392825 | 0.006572 |
| exo_circ_38990 | hsa_circ_0061327 | 1.021478 | 2.933959 | 2.872267885 | 0.000745 |
| exo_circ_67363 | hsa_circ_0082633 | 0.543653 | 1.560745 | 2.87084807 | 0.013726 |
| exo_circ_50274 | hsa_circ_0067918 | 1.068035 | 3.066144 | 2.870826451 | 0.000794 |
| exo_circ_00083 | hsa_circ_0019612 | 0.577959 | 1.658537 | 2.869645505 | 0.018409 |
| exo_circ_44765 | hsa_circ_0058876 | 0.46088 | 1.322153 | 2.868755416 | 0.032744 |
| exo_circ_18999 | hsa_circ_0036103 | 0.420695 | 1.205539 | 2.865589882 | 0.026086 |
| exo_circ_62333 | hsa_circ_0006993 | 0.545238 | 1.562392 | 2.865520917 | 0.019287 |
| exo_circ_28540 | hsa_circ_0004956 | 0.382102 | 1.094551 | 2.864549615 | 0.04372 |
| exo_circ_06395 | hsa_circ_0008391 | 0.72252 | 2.069586 | 2.864399751 | 0.00791 |
| exo_circ_00017 | hsa_circ_0006718 | 0.402303 | 1.15118 | 2.861475892 | 0.037594 |
| exo_circ_78986 | hsa_circ_0005757 | 0.577769 | 1.652457 | 2.860065204 | 0.017177 |
| exo_circ_60427 | hsa_circ_0004873 | 1.696798 | 4.852472 | 2.859782309 | 3.92E-06 |
| exo_circ_43903 | hsa_circ_0008544 | 0.65357 | 1.868635 | 2.859118163 | 0.011469 |
| exo_circ_50931 | hsa_circ_0005286 | 0.423492 | 1.207731 | 2.851839545 | 0.032249 |
| exo_circ_75600 | hsa_circ_0086719 | 0.521078 | 1.485668 | 2.851146703 | 0.027319 |
| exo_circ_19630 | hsa_circ_0036629 | 1.914018 | 5.456403 | 2.850757875 | 5.87E-07 |
| exo_circ_19861 | hsa_circ_0036984 | 1.057089 | 3.012664 | 2.849963489 | 0.000489 |
| exo_circ_00242 | hsa_circ_0003366 | 1.463757 | 4.171318 | 2.849734075 | 4.07E-05 |
| exo_circ_73085 | hsa_circ_0006915 | 0.532607 | 1.517497 | 2.849186777 | 0.016757 |
| exo_circ_73805 | hsa_circ_0001880 | 0.478049 | 1.361315 | 2.847644628 | 0.027379 |
| exo_circ_17683 | hsa_circ_0007457 | 0.356762 | 1.015573 | 2.8466404 | 0.042377 |
| exo_circ_76461 | hsa_circ_0008038 | 0.412015 | 1.17237 | 2.845457094 | 0.034836 |
| exo_circ_68641 | hsa_circ_0003162 | 1.867913 | 5.309404 | 2.842425235 | 8.1E-07 |
| exo_circ_60298 | hsa_circ_0072309 | 2.30398 | 6.544939 | 2.840709949 | 8.72E-09 |
| exo_circ_19248 | hsa_circ_0005036 | 0.714233 | 2.028363 | 2.839915787 | 0.00579 |
| exo_circ_05418 | hsa_circ_0000283 | 0.520006 | 1.476272 | 2.838953546 | 0.026791 |
| exo_circ_03068 | hsa_circ_0008662 | 1.94366 | 5.516765 | 2.838338868 | 2.06E-07 |
| exo_circ_10885 | hsa_circ_0007127 | 1.242219 | 3.52408 | 2.83692296 | 0.000228 |
| exo_circ_04309 | hsa_circ_0005583 | 1.843409 | 5.229406 | 2.836813137 | 1.18E-06 |
| exo_circ_46116 | hsa_circ_0054326 | 0.547995 | 1.553811 | 2.835447078 | 0.022326 |
| exo_circ_39143 | hsa_circ_0002113 | 1.110924 | 3.149745 | 2.835247534 | 0.000612 |
| exo_circ_61598 | hsa_circ_0008164 | 0.910054 | 2.579089 | 2.833995127 | 0.002048 |
| exo_circ_46022 | hsa_circ_0005232 | 1.981099 | 5.586162 | 2.819728624 | 3.81E-07 |
| exo_circ_31679 | hsa_circ_0005357 | 0.576773 | 1.623604 | 2.814978181 | 0.0163 |
| exo_circ_46974 | hsa_circ_0001021 | 1.222384 | 3.439575 | 2.813826009 | 0.000159 |
| exo_circ_22815 | hsa_circ_0042496 | 0.789392 | 2.217388 | 2.808982001 | 0.005866 |
| exo_circ_20563 | hsa_circ_0002696 | 0.450275 | 1.264767 | 2.808877944 | 0.032901 |
| exo_circ_70428 | hsa_circ_0007544 | 1.055393 | 2.962378 | 2.806895331 | 0.000607 |
| exo_circ_40632 | hsa_circ_0003303 | 0.438859 | 1.230818 | 2.804583922 | 0.037392 |
| exo_circ_55821 | hsa_circ_0068926 | 1.19179 | 3.342135 | 2.804298231 | 0.000245 |
| exo_circ_37151 | hsa_circ_0059481 | 0.563184 | 1.579117 | 2.803910383 | 0.025535 |
| exo_circ_06904 | hsa_circ_0006629 | 1.923624 | 5.389984 | 2.801994958 | 3.61E-07 |
| exo_circ_38621 | hsa_circ_0001172 | 0.43045 | 1.202064 | 2.792575297 | 0.046791 |
| exo_circ_67911 | hsa_circ_0083092 | 0.608587 | 1.699019 | 2.791743183 | 0.015696 |
| exo_circ_16432 | hsa_circ_0003045 | 1.54597 | 4.306388 | 2.785558138 | 1.86E-05 |
| exo_circ_74942 | hsa_circ_0089715 | 1.512404 | 4.210974 | 2.784292481 | 4.08E-05 |
| exo_circ_44858 | hsa_circ_0003947 | 0.600761 | 1.672141 | 2.783371628 | 0.014386 |
| exo_circ_52368 | hsa_circ_0007291 | 1.66652 | 4.638076 | 2.783091278 | 9.79E-06 |
| exo_circ_26086 | hsa_circ_0005653 | 0.70434 | 1.960228 | 2.783071425 | 0.009549 |
| exo_circ_20040 | hsa_circ_0037806 | 0.745115 | 2.069792 | 2.777813774 | 0.003362 |
| exo_circ_19297 | hsa_circ_0000640 | 1.2241 | 3.398814 | 2.776581072 | 0.000448 |
| exo_circ_34610 | hsa_circ_0000044 | 1.195195 | 3.316436 | 2.774808023 | 0.000478 |
| exo_circ_38408 | hsa_circ_0060734 | 0.600569 | 1.664769 | 2.771986464 | 0.023522 |
| exo_circ_76550 | hsa_circ_0007162 | 0.595677 | 1.650598 | 2.770959482 | 0.016766 |
| exo_circ_27180 | hsa_circ_0003423 | 1.76499 | 4.890664 | 2.770930611 | 3.65E-06 |
| exo_circ_28792 | hsa_circ_0051220 | 0.46066 | 1.276101 | 2.770158182 | 0.020979 |
| exo_circ_56951 | hsa_circ_0069922 | 0.503352 | 1.394127 | 2.76968813 | 0.032807 |
| exo_circ_17288 | hsa_circ_0008346 | 0.679731 | 1.882239 | 2.76909452 | 0.010994 |
| exo_circ_74526 | hsa_circ_0088614 | 1.005079 | 2.783056 | 2.76899357 | 0.001892 |
| exo_circ_70285 | hsa_circ_0002620 | 0.991909 | 2.745929 | 2.768327351 | 0.001688 |
| exo_circ_09403 | hsa_circ_0000374 | 1.044212 | 2.889593 | 2.767249205 | 0.000965 |
| exo_circ_32787 | hsa_circ_0010487 | 0.436078 | 1.20549 | 2.764389818 | 0.048189 |
| exo_circ_23062 | hsa_circ_0042799 | 1.92782 | 5.325459 | 2.762424942 | 1.73E-06 |
| exo_circ_19690 | hsa_circ_0036683 | 1.266459 | 3.49492 | 2.759598529 | 0.000135 |
| exo_circ_69824 | hsa_circ_0080849 | 0.562688 | 1.552301 | 2.758726068 | 0.016404 |
| exo_circ_59364 | hsa_circ_0003506 | 0.831507 | 2.290718 | 2.754899172 | 0.004871 |
| exo_circ_40900 | hsa_circ_0052621 | 0.536179 | 1.476002 | 2.752815833 | 0.046669 |
| exo_circ_60790 | hsa_circ_0006381 | 0.366251 | 1.007925 | 2.75200167 | 0.044445 |
| exo_circ_34262 | hsa_circ_0005667 | 1.330159 | 3.659398 | 2.751097629 | 0.000176 |
| exo_circ_51813 | hsa_circ_0064735 | 0.915637 | 2.51733 | 2.749267069 | 0.003546 |
| exo_circ_42704 | hsa_circ_0057105 | 0.961656 | 2.643203 | 2.748594719 | 0.001682 |
| exo_circ_65532 | hsa_circ_0075551 | 0.494197 | 1.357137 | 2.746148251 | 0.048471 |
| exo_circ_52385 | hsa_circ_0065244 | 1.974614 | 5.420914 | 2.745302507 | 1.01E-06 |
| exo_circ_25822 | hsa_circ_0000818 | 1.199912 | 3.288308 | 2.740458467 | 0.000274 |
| exo_circ_60663 | hsa_circ_0008731 | 0.77169 | 2.113952 | 2.739381474 | 0.007825 |
| exo_circ_19778 | hsa_circ_0003010 | 1.062057 | 2.905779 | 2.735991475 | 0.000789 |
| exo_circ_30609 | hsa_circ_0000131 | 0.631493 | 1.727697 | 2.735891917 | 0.019098 |
| exo_circ_19514 | hsa_circ_0005745 | 0.689927 | 1.886967 | 2.735023801 | 0.01027 |
| exo_circ_16292 | hsa_circ_0000558 | 1.977123 | 5.399818 | 2.731149513 | 5.4E-07 |
| exo_circ_12745 | hsa_circ_0003401 | 1.071256 | 2.921672 | 2.727333833 | 0.001234 |
| exo_circ_18200 | hsa_circ_0006380 | 0.961948 | 2.62294 | 2.726696755 | 0.001736 |
| exo_circ_44083 | hsa_circ_0058042 | 0.816327 | 2.225323 | 2.726018632 | 0.005677 |
| exo_circ_34728 | hsa_circ_0011462 | 0.607258 | 1.654413 | 2.724399996 | 0.009939 |
| exo_circ_19167 | hsa_circ_0036348 | 0.725027 | 1.974129 | 2.722834434 | 0.008456 |
| exo_circ_09681 | hsa_circ_0002881 | 1.049436 | 2.855847 | 2.721317046 | 0.001372 |
| exo_circ_01854 | hsa_circ_0000228 | 0.941072 | 2.560952 | 2.721313319 | 0.00183 |
| exo_circ_31789 | hsa_circ_0015438 | 0.812828 | 2.210108 | 2.71903641 | 0.004854 |
| exo_circ_22635 | hsa_circ_0042356 | 0.705459 | 1.91376 | 2.712786966 | 0.011193 |
| exo_circ_37587 | hsa_circ_0001140 | 0.60448 | 1.639411 | 2.712102726 | 0.016871 |
| exo_circ_37154 | hsa_circ_0059482 | 0.47023 | 1.27474 | 2.710886514 | 0.049662 |
| exo_circ_16277 | hsa_circ_0032877 | 0.797838 | 2.162085 | 2.709929849 | 0.007752 |
| exo_circ_76464 | hsa_circ_0007698 | 1.46417 | 3.965357 | 2.708263352 | 9.84E-05 |
| exo_circ_00750 | hsa_circ_0003074 | 1.093922 | 2.961733 | 2.707443424 | 0.000961 |
| exo_circ_62113 | hsa_circ_0073379 | 0.887217 | 2.401672 | 2.706972696 | 0.004697 |
| exo_circ_08124 | hsa_circ_0003059 | 0.69742 | 1.887867 | 2.706928889 | 0.015081 |
| exo_circ_13746 | hsa_circ_0004166 | 0.81923 | 2.216299 | 2.705344645 | 0.005793 |
| exo_circ_28494 | hsa_circ_0008030 | 0.680306 | 1.839566 | 2.704026389 | 0.004797 |
| exo_circ_18597 | hsa_circ_0035818 | 0.427813 | 1.154835 | 2.699390951 | 0.041285 |
| exo_circ_18933 | hsa_circ_0036044 | 1.094738 | 2.955023 | 2.699297362 | 0.001403 |
| exo_circ_20893 | hsa_circ_0006127 | 1.653702 | 4.452849 | 2.6926544 | 6.83E-07 |
| exo_circ_33814 | hsa_circ_0017092 | 1.004986 | 2.70557 | 2.692147328 | 0.003316 |
| exo_circ_34385 | hsa_circ_0007283 | 0.707723 | 1.898689 | 2.682813162 | 0.012036 |
| exo_circ_53915 | hsa_circ_0003875 | 0.75023 | 2.008021 | 2.676539159 | 0.008178 |
| exo_circ_57327 | hsa_circ_0001421 | 0.711131 | 1.899727 | 2.671417761 | 0.012991 |
| exo_circ_25930 | hsa_circ_0006400 | 0.484986 | 1.295153 | 2.670496713 | 0.047247 |
| exo_circ_31495 | hsa_circ_0005230 | 0.759501 | 2.028182 | 2.670413579 | 0.008018 |
| exo_circ_77823 | hsa_circ_0006971 | 0.507301 | 1.352463 | 2.665995934 | 0.042417 |
| exo_circ_71933 | hsa_circ_0001784 | 0.557456 | 1.485325 | 2.664470494 | 0.035494 |
| exo_circ_01850 | hsa_circ_0003519 | 0.559819 | 1.491561 | 2.66436372 | 0.02966 |
| exo_circ_41367 | hsa_circ_0007052 | 1.576566 | 4.197972 | 2.662732023 | 6.34E-05 |
| exo_circ_41329 | hsa_circ_0056285 | 0.857281 | 2.280051 | 2.659631025 | 0.006155 |
| exo_circ_06733 | hsa_circ_0023700 | 0.724775 | 1.927585 | 2.659564218 | 0.017408 |
| exo_circ_02715 | hsa_circ_0018484 | 0.82502 | 2.194008 | 2.659339678 | 0.00766 |
| exo_circ_60530 | hsa_circ_0004840 | 0.702795 | 1.868657 | 2.658894612 | 0.013641 |
| exo_circ_25586 | hsa_circ_0005320 | 0.625469 | 1.66288 | 2.658611216 | 0.013034 |
| exo_circ_00879 | hsa_circ_0000215 | 0.544618 | 1.447643 | 2.658091097 | 0.03728 |
| exo_circ_50229 | hsa_circ_0006893 | 2.117047 | 5.625439 | 2.657210914 | 9.88E-07 |
| exo_circ_12270 | hsa_circ_0008651 | 0.937155 | 2.487082 | 2.653862621 | 0.004631 |
| exo_circ_35723 | hsa_circ_0012634 | 0.76212 | 2.022222 | 2.65341733 | 0.012331 |
| exo_circ_47514 | hsa_circ_0004726 | 2.237731 | 5.928202 | 2.649202525 | 3.25E-07 |
| exo_circ_02892 | hsa_circ_0000240 | 0.578056 | 1.531079 | 2.64866823 | 0.025139 |
| exo_circ_09170 | hsa_circ_0008664 | 0.535188 | 1.416357 | 2.646465841 | 0.045866 |
| exo_circ_21989 | hsa_circ_0040559 | 0.584021 | 1.545288 | 2.645944856 | 0.034746 |
| exo_circ_56263 | hsa_circ_0005991 | 2.103841 | 5.554267 | 2.640059882 | 1.96E-06 |
| exo_circ_65594 | hsa_circ_0007874 | 1.076845 | 2.842276 | 2.639448193 | 0.002065 |
| exo_circ_30815 | hsa_circ_0014592 | 2.210107 | 5.824371 | 2.635334705 | 3.8E-07 |
| exo_circ_23906 | hsa_circ_0007635 | 0.844591 | 2.225542 | 2.635051993 | 0.00552 |
| exo_circ_19606 | hsa_circ_0036598 | 0.469437 | 1.236638 | 2.634300169 | 0.044166 |
| exo_circ_00364 | hsa_circ_0020024 | 1.630574 | 4.295093 | 2.634098572 | 2.68E-05 |
| exo_circ_30690 | hsa_circ_0004717 | 1.058809 | 2.788911 | 2.634007683 | 0.002122 |
| exo_circ_25799 | hsa_circ_0046419 | 0.608456 | 1.599794 | 2.629268999 | 0.026022 |
| exo_circ_48128 | hsa_circ_0066719 | 0.536406 | 1.408642 | 2.626071587 | 0.033447 |
| exo_circ_47131 | hsa_circ_0005504 | 0.594945 | 1.561224 | 2.624150062 | 0.027884 |
| exo_circ_00922 | hsa_circ_0006545 | 1.362222 | 3.574188 | 2.62379231 | 0.000352 |
| exo_circ_07496 | hsa_circ_0027944 | 0.972215 | 2.550157 | 2.623038854 | 0.005317 |
| exo_circ_05943 | hsa_circ_0005204 | 1.085769 | 2.845333 | 2.620570176 | 0.002783 |
| exo_circ_00193 | hsa_circ_0019773 | 0.696439 | 1.824606 | 2.619906243 | 0.016118 |
| exo_circ_45328 | hsa_circ_0000987 | 1.04798 | 2.744568 | 2.618912078 | 0.002571 |
| exo_circ_44867 | hsa_circ_0058919 | 0.837731 | 2.193451 | 2.618322444 | 0.009068 |
| exo_circ_49799 | hsa_circ_0007630 | 1.312905 | 3.42719 | 2.610387209 | 0.000569 |
| exo_circ_24024 | hsa_circ_0044177 | 1.385636 | 3.616495 | 2.609989904 | 0.000138 |
| exo_circ_65190 | hsa_circ_0076704 | 0.990866 | 2.584177 | 2.607998889 | 0.004847 |
| exo_circ_74008 | hsa_circ_0008192 | 0.955581 | 2.489241 | 2.604950242 | 0.006358 |
| exo_circ_78397 | hsa_circ_0006364 | 1.325802 | 3.44997 | 2.60217555 | 0.000211 |
| exo_circ_73834 | hsa_circ_0004109 | 0.913234 | 2.376055 | 2.601802949 | 0.006758 |
| exo_circ_50246 | hsa_circ_0007854 | 0.599435 | 1.558364 | 2.599723523 | 0.023523 |
| exo_circ_50391 | hsa_circ_0068013 | 0.707732 | 1.839451 | 2.59907892 | 0.01824 |
| exo_circ_42398 | hsa_circ_0002029 | 0.823774 | 2.13952 | 2.59721594 | 0.006907 |
| exo_circ_22814 | hsa_circ_0007716 | 1.066128 | 2.76676 | 2.595148626 | 0.003714 |
| exo_circ_48122 | hsa_circ_0066714 | 1.06162 | 2.75372 | 2.593885532 | 0.002927 |
| exo_circ_67517 | hsa_circ_0082688 | 0.813967 | 2.107427 | 2.589082229 | 0.009451 |
| exo_circ_65670 | hsa_circ_0077084 | 0.651006 | 1.682031 | 2.583741259 | 0.02499 |
| exo_circ_63866 | hsa_circ_0078364 | 0.510215 | 1.318213 | 2.583642012 | 0.04672 |
| exo_circ_72796 | hsa_circ_0005883 | 1.572018 | 4.059376 | 2.582270971 | 0.000183 |
| exo_circ_14015 | hsa_circ_0007750 | 0.350573 | 0.90519 | 2.582034752 | 0.044562 |
| exo_circ_03505 | hsa_circ_0007229 | 0.473694 | 1.222891 | 2.581603606 | 0.049557 |
| exo_circ_62139 | hsa_circ_0001512 | 1.80753 | 4.66381 | 2.580210749 | 1.6E-05 |
| exo_circ_26512 | hsa_circ_0003805 | 1.293139 | 3.336061 | 2.579817115 | 0.0008 |
| exo_circ_39052 | hsa_circ_0001181 | 1.640308 | 4.229867 | 2.578703027 | 9.93E-05 |
| exo_circ_33977 | hsa_circ_0007442 | 0.698613 | 1.801338 | 2.578449108 | 0.018716 |
| exo_circ_68667 | hsa_circ_0079813 | 1.843349 | 4.74986 | 2.576755815 | 3.98E-05 |
| exo_circ_34894 | hsa_circ_0006578 | 1.251166 | 3.222256 | 2.57540357 | 0.001015 |
| exo_circ_05736 | hsa_circ_0007001 | 0.490231 | 1.261962 | 2.574219996 | 0.043945 |
| exo_circ_50234 | hsa_circ_0067897 | 0.87356 | 2.248019 | 2.573399427 | 0.008631 |
| exo_circ_68670 | hsa_circ_0008336 | 1.682635 | 4.324985 | 2.57036516 | 6.96E-05 |
| exo_circ_24967 | hsa_circ_0007736 | 1.021132 | 2.621064 | 2.566822255 | 0.004151 |
| exo_circ_10720 | hsa_circ_0025244 | 0.622179 | 1.596847 | 2.56653923 | 0.033422 |
| exo_circ_33076 | hsa_circ_0004314 | 1.14558 | 2.935426 | 2.562391832 | 0.002127 |
| exo_circ_58783 | hsa_circ_0003280 | 1.302251 | 3.331043 | 2.557911049 | 0.000306 |
| exo_circ_05617 | hsa_circ_0007401 | 0.919699 | 2.352447 | 2.557843074 | 0.007984 |
| exo_circ_33813 | hsa_circ_0004550 | 0.669925 | 1.713116 | 2.557176247 | 0.021669 |
| exo_circ_10070 | hsa_circ_0006535 | 0.741838 | 1.896864 | 2.556977985 | 0.012483 |
| exo_circ_25175 | hsa_circ_0045334 | 0.539512 | 1.376065 | 2.550573036 | 0.048273 |
| exo_circ_77932 | hsa_circ_0006355 | 1.577095 | 4.020374 | 2.549228012 | 0.000244 |
| exo_circ_52825 | hsa_circ_0066187 | 0.804771 | 2.04923 | 2.546351615 | 0.016399 |
| exo_circ_03960 | hsa_circ_0006371 | 0.820159 | 2.087562 | 2.545313667 | 0.014753 |
| exo_circ_08297 | hsa_circ_0000448 | 2.072668 | 5.270055 | 2.542642744 | 5.1E-06 |
| exo_circ_40387 | hsa_circ_0005206 | 0.677196 | 1.719793 | 2.539579902 | 0.020159 |
| exo_circ_55457 | hsa_circ_0001458 | 1.214994 | 3.083388 | 2.537780299 | 0.00123 |
| exo_circ_60113 | hsa_circ_0072132 | 0.866224 | 2.197778 | 2.537194409 | 0.008767 |
| exo_circ_50399 | hsa_circ_0007042 | 0.535335 | 1.357465 | 2.535731615 | 0.046502 |
| exo_circ_53690 | hsa_circ_0006007 | 0.795888 | 2.016701 | 2.533899874 | 0.010721 |
| exo_circ_73790 | hsa_circ_0009029 | 0.714863 | 1.808775 | 2.530241046 | 0.025531 |
| exo_circ_73534 | hsa_circ_0002798 | 1.146822 | 2.901359 | 2.529913121 | 0.001761 |
| exo_circ_48534 | hsa_circ_0066971 | 1.136292 | 2.867331 | 2.52340958 | 0.003589 |
| exo_circ_40331 | hsa_circ_0063331 | 1.159067 | 2.924468 | 2.523122738 | 0.002038 |
| exo_circ_65075 | hsa_circ_0076410 | 0.645446 | 1.627446 | 2.521426606 | 0.037794 |
| exo_circ_25665 | hsa_circ_0008114 | 1.513159 | 3.810979 | 2.518557806 | 0.000332 |
| exo_circ_17368 | hsa_circ_0034803 | 1.343343 | 3.38296 | 2.518314381 | 0.000826 |
| exo_circ_10788 | hsa_circ_0003599 | 0.491228 | 1.235818 | 2.515770372 | 0.032554 |
| exo_circ_22186 | hsa_circ_0004640 | 1.565053 | 3.934906 | 2.514231929 | 0.000182 |
| exo_circ_01804 | hsa_circ_0018069 | 1.073975 | 2.698638 | 2.512757653 | 0.003853 |
| exo_circ_34734 | hsa_circ_0011471 | 2.534552 | 6.366432 | 2.511856529 | 2.05E-07 |
| exo_circ_14249 | hsa_circ_0031583 | 0.750436 | 1.88372 | 2.510166547 | 0.02214 |
| exo_circ_30455 | hsa_circ_0004437 | 0.86495 | 2.169428 | 2.508155551 | 0.015032 |
| exo_circ_25845 | hsa_circ_0005245 | 1.110117 | 2.784076 | 2.507912896 | 0.001871 |
| exo_circ_27472 | hsa_circ_0000857 | 1.211653 | 3.038194 | 2.507479189 | 0.00118 |
| exo_circ_67609 | hsa_circ_0002980 | 0.759279 | 1.902301 | 2.50540557 | 0.011883 |
| exo_circ_27705 | hsa_circ_0046882 | 0.734654 | 1.840516 | 2.505282572 | 0.024341 |
| exo_circ_36338 | hsa_circ_0006395 | 1.128983 | 2.826543 | 2.503619314 | 0.003291 |
| exo_circ_13313 | hsa_circ_0030509 | 1.513104 | 3.787235 | 2.502957016 | 0.000486 |
| exo_circ_31036 | hsa_circ_0004617 | 0.695237 | 1.739906 | 2.502609575 | 0.013172 |
| exo_circ_35423 | hsa_circ_0008197 | 0.607882 | 1.520907 | 2.501978312 | 0.035622 |
| exo_circ_18711 | hsa_circ_0035901 | 1.068758 | 2.671264 | 2.499409406 | 0.00477 |
| exo_circ_49109 | hsa_circ_0067474 | 0.575372 | 1.437161 | 2.497794028 | 0.039586 |
| exo_circ_00152 | hsa_circ_0000257 | 1.01264 | 2.527164 | 2.49561979 | 0.007356 |
| exo_circ_30753 | hsa_circ_0014566 | 0.761619 | 1.900244 | 2.495006258 | 0.020594 |
| exo_circ_10172 | hsa_circ_0004313 | 1.779592 | 4.436631 | 2.493060526 | 7.32E-05 |
| exo_circ_19627 | hsa_circ_0036627 | 0.631568 | 1.570149 | 2.486112205 | 0.046369 |
| exo_circ_18860 | hsa_circ_0008798 | 0.839038 | 2.084593 | 2.484503479 | 0.01497 |
| exo_circ_37020 | hsa_circ_0004161 | 0.742981 | 1.845192 | 2.483497243 | 0.010044 |
| exo_circ_75601 | hsa_circ_0086720 | 2.663738 | 6.614989 | 2.483348072 | 1.52E-07 |
| exo_circ_21178 | hsa_circ_0005813 | 0.843705 | 2.093776 | 2.48164551 | 0.014697 |
| exo_circ_74858 | hsa_circ_0089371 | 1.675159 | 4.156639 | 2.481339638 | 7.78E-05 |
| exo_circ_06738 | hsa_circ_0023701 | 0.973638 | 2.413939 | 2.479298074 | 0.005603 |
| exo_circ_54550 | hsa_circ_0001441 | 0.900639 | 2.231756 | 2.477969136 | 0.011561 |
| exo_circ_26648 | hsa_circ_0047476 | 0.696083 | 1.724242 | 2.477064887 | 0.024225 |
| exo_circ_32174 | hsa_circ_0015733 | 0.702191 | 1.739147 | 2.476742046 | 0.024921 |
| exo_circ_09683 | hsa_circ_0024946 | 1.120923 | 2.769504 | 2.470735872 | 0.003015 |
| exo_circ_33635 | hsa_circ_0000198 | 0.74368 | 1.835428 | 2.468033604 | 0.029042 |
| exo_circ_42393 | hsa_circ_0003296 | 1.294654 | 3.192496 | 2.465907352 | 0.001728 |
| exo_circ_20575 | hsa_circ_0005690 | 0.633162 | 1.560878 | 2.465213209 | 0.037045 |
| exo_circ_60385 | hsa_circ_0007795 | 1.246477 | 3.069726 | 2.462722118 | 0.002838 |
| exo_circ_02700 | hsa_circ_0018478 | 0.957061 | 2.355923 | 2.461622054 | 0.007853 |
| exo_circ_63775 | hsa_circ_0078328 | 2.150629 | 5.285189 | 2.457509059 | 2.74E-05 |
| exo_circ_42186 | hsa_circ_0008010 | 0.834128 | 2.045698 | 2.452500451 | 0.024672 |
| exo_circ_34538 | hsa_circ_0000041 | 0.849816 | 2.077016 | 2.444078151 | 0.012664 |
| exo_circ_25468 | hsa_circ_0000741 | 1.791302 | 4.377805 | 2.443923081 | 0.000165 |
| exo_circ_77827 | hsa_circ_0091669 | 1.231723 | 3.001832 | 2.43710009 | 0.004779 |
| exo_circ_32523 | hsa_circ_0016123 | 0.855368 | 2.08307 | 2.435291585 | 0.021395 |
| exo_circ_74780 | hsa_circ_0089254 | 0.838628 | 2.041899 | 2.434808451 | 0.021658 |
| exo_circ_28192 | hsa_circ_0050119 | 0.988275 | 2.405161 | 2.43369584 | 0.005997 |
| exo_circ_55673 | hsa_circ_0008460 | 0.591714 | 1.437693 | 2.4297093 | 0.048384 |
| exo_circ_01235 | hsa_circ_0017854 | 1.127344 | 2.736654 | 2.427524752 | 0.005286 |
| exo_circ_60525 | hsa_circ_0072437 | 1.390036 | 3.367431 | 2.422549136 | 0.001224 |
| exo_circ_19065 | hsa_circ_0006509 | 0.915843 | 2.217094 | 2.420821541 | 0.015958 |
| exo_circ_52028 | hsa_circ_0003910 | 2.407399 | 5.82444 | 2.419390754 | 5.03E-06 |
| exo_circ_22244 | hsa_circ_0040929 | 1.673584 | 4.04834 | 2.418963401 | 0.000317 |
| exo_circ_29695 | hsa_circ_0000099 | 2.443174 | 5.903018 | 2.416126175 | 1.17E-06 |
| exo_circ_52553 | hsa_circ_0008343 | 0.887703 | 2.138167 | 2.408650356 | 0.017718 |
| exo_circ_36542 | hsa_circ_0009117 | 0.645709 | 1.553185 | 2.405396389 | 0.048944 |
| exo_circ_58274 | hsa_circ_0007726 | 0.707454 | 1.700699 | 2.403969354 | 0.034162 |
| exo_circ_07284 | hsa_circ_0024085 | 1.667727 | 4.0087 | 2.403690601 | 0.000509 |
| exo_circ_30077 | hsa_circ_0000111 | 2.841516 | 6.826326 | 2.402353545 | 4.4E-07 |
| exo_circ_24206 | hsa_circ_0002854 | 0.780416 | 1.8748 | 2.402307055 | 0.027301 |
| exo_circ_35276 | hsa_circ_0002053 | 0.81814 | 1.964618 | 2.401323379 | 0.021728 |
| exo_circ_28901 | hsa_circ_0000940 | 2.477205 | 5.945695 | 2.400163019 | 2.41E-06 |
| exo_circ_72883 | hsa_circ_0084678 | 1.373218 | 3.295853 | 2.400094941 | 0.001652 |
| exo_circ_56361 | hsa_circ_0069615 | 1.524293 | 3.658132 | 2.399887809 | 0.00108 |
| exo_circ_75478 | hsa_circ_0086190 | 1.147817 | 2.752808 | 2.398298559 | 0.005201 |
| exo_circ_53371 | hsa_circ_0004340 | 1.098821 | 2.634976 | 2.398003243 | 0.006955 |
| exo_circ_77096 | hsa_circ_0005992 | 0.776114 | 1.860309 | 2.396952472 | 0.018969 |
| exo_circ_22166 | hsa_circ_0040819 | 0.561453 | 1.345528 | 2.396508565 | 0.049366 |
| exo_circ_50614 | hsa_circ_0068142 | 1.443492 | 3.459006 | 2.396277421 | 0.001385 |
| exo_circ_28594 | hsa_circ_0002356 | 0.7351 | 1.759418 | 2.39344086 | 0.038606 |
| exo_circ_79066 | hsa_circ_0001953 | 3.92727 | 9.391625 | 2.391388031 | 1.96E-10 |
| exo_circ_71008 | hsa_circ_0085496 | 0.848701 | 2.028341 | 2.389936431 | 0.021334 |
| exo_circ_06628 | hsa_circ_0003307 | 0.84702 | 2.02319 | 2.388598465 | 0.010028 |
| exo_circ_55277 | hsa_circ_0069244 | 0.762086 | 1.819862 | 2.3880008 | 0.033372 |
| exo_circ_21482 | hsa_circ_0000706 | 1.825321 | 4.35798 | 2.387514033 | 0.000202 |
| exo_circ_53337 | hsa_circ_0001322 | 2.669256 | 6.372113 | 2.387224859 | 2.68E-06 |
| exo_circ_74070 | hsa_circ_0088179 | 1.151139 | 2.746858 | 2.386208858 | 0.004253 |
| exo_circ_59404 | hsa_circ_0074828 | 0.634501 | 1.512854 | 2.384321643 | 0.031004 |
| exo_circ_14908 | hsa_circ_0007101 | 0.906532 | 2.160053 | 2.382764304 | 0.022842 |
| exo_circ_58790 | hsa_circ_0007440 | 0.880489 | 2.097177 | 2.381833123 | 0.02197 |
| exo_circ_75674 | hsa_circ_0008518 | 1.774683 | 4.216757 | 2.376061883 | 0.000146 |
| exo_circ_47241 | hsa_circ_0052490 | 0.919111 | 2.182403 | 2.374473321 | 0.016627 |
| exo_circ_49317 | hsa_circ_0006347 | 1.556816 | 3.696549 | 2.374428433 | 0.000639 |
| exo_circ_42721 | hsa_circ_0002642 | 1.368097 | 3.245138 | 2.372008462 | 0.001175 |
| exo_circ_72841 | hsa_circ_0084663 | 0.62008 | 1.469068 | 2.369159065 | 0.031418 |
| exo_circ_72183 | hsa_circ_0003316 | 0.829225 | 1.964014 | 2.368492941 | 0.025647 |
| exo_circ_13878 | hsa_circ_0006153 | 1.040402 | 2.46349 | 2.367824658 | 0.006452 |
| exo_circ_10686 | hsa_circ_0006550 | 0.711465 | 1.683797 | 2.366660629 | 0.039254 |
| exo_circ_66445 | hsa_circ_0081813 | 0.923628 | 2.183265 | 2.363792542 | 0.01318 |
| exo_circ_52595 | hsa_circ_0065769 | 0.909397 | 2.149607 | 2.363772293 | 0.016106 |
| exo_circ_75729 | hsa_circ_0006515 | 1.853761 | 4.381191 | 2.36340631 | 0.000248 |
| exo_circ_06723 | hsa_circ_0008790 | 0.590214 | 1.392764 | 2.359761942 | 0.041717 |
| exo_circ_61268 | hsa_circ_0072897 | 1.721675 | 4.060097 | 2.358225063 | 0.000581 |
| exo_circ_59710 | hsa_circ_0075092 | 1.016085 | 2.39383 | 2.355934727 | 0.011832 |
| exo_circ_12198 | hsa_circ_0008760 | 0.718449 | 1.690563 | 2.353072996 | 0.038741 |
| exo_circ_40871 | hsa_circ_0055947 | 1.212684 | 2.852693 | 2.352379985 | 0.005822 |
| exo_circ_04591 | hsa_circ_0006374 | 1.639329 | 3.85601 | 2.352187635 | 0.000593 |
| exo_circ_30249 | hsa_circ_0006470 | 2.015458 | 4.740053 | 2.351848614 | 6.73E-05 |
| exo_circ_65539 | hsa_circ_0007429 | 1.897592 | 4.455992 | 2.34823497 | 0.000167 |
| exo_circ_33597 | hsa_circ_0007848 | 1.830597 | 4.293853 | 2.34560212 | 7.64E-05 |
| exo_circ_12746 | hsa_circ_0008937 | 1.308396 | 3.068814 | 2.345478643 | 0.004116 |
| exo_circ_50260 | hsa_circ_0067906 | 0.922337 | 2.163134 | 2.345275814 | 0.019476 |
| exo_circ_36540 | hsa_circ_0006837 | 1.243374 | 2.9144 | 2.343945897 | 0.004246 |
| exo_circ_28005 | hsa_circ_0008950 | 0.803636 | 1.882354 | 2.342297009 | 0.017838 |
| exo_circ_07053 | hsa_circ_0023984 | 0.920751 | 2.154501 | 2.339937814 | 0.020122 |
| exo_circ_22256 | hsa_circ_0000727 | 0.738338 | 1.727337 | 2.33949547 | 0.037361 |
| exo_circ_26751 | hsa_circ_0006491 | 1.532374 | 3.581123 | 2.33697633 | 0.002288 |
| exo_circ_02655 | hsa_circ_0007626 | 0.792708 | 1.850425 | 2.334307736 | 0.032458 |
| exo_circ_22176 | hsa_circ_0000669 | 1.50397 | 3.507343 | 2.332056507 | 0.001426 |
| exo_circ_05350 | hsa_circ_0004132 | 0.48998 | 1.141964 | 2.330634631 | 0.049381 |
| exo_circ_75008 | hsa_circ_0008952 | 2.958624 | 6.876408 | 2.32419168 | 8.4E-07 |
| exo_circ_18471 | hsa_circ_0008104 | 0.743496 | 1.722981 | 2.317405597 | 0.030998 |
| exo_circ_74444 | hsa_circ_0009007 | 0.679387 | 1.574242 | 2.317149684 | 0.047709 |
| exo_circ_74887 | hsa_circ_0004338 | 0.766779 | 1.774914 | 2.31476415 | 0.03625 |
| exo_circ_20739 | hsa_circ_0005642 | 1.714664 | 3.968698 | 2.314563343 | 0.000629 |
| exo_circ_11145 | hsa_circ_0002886 | 1.80989 | 4.18884 | 2.314416694 | 0.000402 |
| exo_circ_53828 | hsa_circ_0007477 | 1.463215 | 3.386464 | 2.314400273 | 0.001469 |
| exo_circ_08367 | hsa_circ_0004513 | 1.142573 | 2.643745 | 2.31385227 | 0.006838 |
| exo_circ_01857 | hsa_circ_0000230 | 2.607457 | 6.022556 | 2.309743319 | 1.14E-05 |
| exo_circ_07905 | hsa_circ_0000438 | 1.268067 | 2.92677 | 2.308057173 | 0.004798 |
| exo_circ_55837 | hsa_circ_0003869 | 0.724785 | 1.672822 | 2.308024605 | 0.047072 |
| exo_circ_72800 | hsa_circ_0004863 | 0.664156 | 1.532685 | 2.307716849 | 0.033291 |
| exo_circ_15043 | hsa_circ_0004846 | 1.353886 | 3.123412 | 2.306997868 | 0.004343 |
| exo_circ_01864 | hsa_circ_0002140 | 0.920688 | 2.121287 | 2.304023156 | 0.022793 |
| exo_circ_64748 | hsa_circ_0006757 | 1.753399 | 4.038804 | 2.303414697 | 0.000442 |
| exo_circ_76918 | hsa_circ_0087631 | 2.63153 | 6.060911 | 2.303188556 | 2.79E-06 |
| exo_circ_19140 | hsa_circ_0000633 | 0.801082 | 1.844417 | 2.302406103 | 0.032977 |
| exo_circ_35227 | hsa_circ_0007693 | 1.121532 | 2.578588 | 2.299165196 | 0.008603 |
| exo_circ_64317 | hsa_circ_0001580 | 1.162509 | 2.67228 | 2.29871847 | 0.003409 |
| exo_circ_05113 | hsa_circ_0021396 | 1.945213 | 4.46334 | 2.294524741 | 0.000351 |
| exo_circ_69432 | hsa_circ_0008970 | 1.484782 | 3.406789 | 2.29447047 | 0.002614 |
| exo_circ_51140 | hsa_circ_0007331 | 1.186789 | 2.7203 | 2.292152042 | 0.008529 |
| exo_circ_20909 | hsa_circ_0006719 | 1.689927 | 3.871446 | 2.29089522 | 0.000914 |
| exo_circ_31872 | hsa_circ_0000164 | 1.025436 | 2.346127 | 2.287931503 | 0.013827 |
| exo_circ_71249 | hsa_circ_0006050 | 1.801931 | 4.119653 | 2.286243216 | 0.000841 |
| exo_circ_77485 | hsa_circ_0091427 | 0.796445 | 1.820715 | 2.28605137 | 0.038154 |
| exo_circ_40272 | hsa_circ_0006385 | 1.12167 | 2.563204 | 2.285168056 | 0.010084 |
| exo_circ_03732 | hsa_circ_0019171 | 1.128091 | 2.57679 | 2.284203906 | 0.014841 |
| exo_circ_12280 | hsa_circ_0005874 | 1.156357 | 2.641131 | 2.284009262 | 0.013934 |
| exo_circ_26151 | hsa_circ_0006733 | 1.426287 | 3.256336 | 2.283086069 | 0.002955 |
| exo_circ_38092 | hsa_circ_0060420 | 1.577489 | 3.60104 | 2.282767989 | 0.002167 |
| exo_circ_39211 | hsa_circ_0005076 | 0.727204 | 1.659266 | 2.281705307 | 0.038046 |
| exo_circ_00730 | hsa_circ_0000213 | 1.84162 | 4.200648 | 2.280953024 | 0.000242 |
| exo_circ_13017 | hsa_circ_0000489 | 1.216485 | 2.771883 | 2.278599179 | 0.001025 |
| exo_circ_23318 | hsa_circ_0008604 | 2.019184 | 4.600258 | 2.278275463 | 4.78E-05 |
| exo_circ_71963 | hsa_circ_0083826 | 1.424285 | 3.244194 | 2.277769641 | 0.003166 |
| exo_circ_04886 | hsa_circ_0000368 | 1.320916 | 3.002201 | 2.272817585 | 0.004084 |
| exo_circ_40212 | hsa_circ_0001222 | 0.794118 | 1.804268 | 2.272040406 | 0.049813 |
| exo_circ_72267 | hsa_circ_0002754 | 1.262903 | 2.867818 | 2.270813366 | 0.007958 |
| exo_circ_51630 | hsa_circ_0001277 | 0.859987 | 1.952023 | 2.26982788 | 0.018818 |
| exo_circ_05538 | hsa_circ_0006988 | 2.71121 | 6.152156 | 2.269155311 | 4.03E-06 |
| exo_circ_38547 | hsa_circ_0001170 | 1.847101 | 4.191119 | 2.269025295 | 0.000786 |
| exo_circ_41309 | hsa_circ_0001064 | 0.895378 | 2.029243 | 2.266354039 | 0.031124 |
| exo_circ_31362 | hsa_circ_0003644 | 0.811454 | 1.838853 | 2.266120999 | 0.034587 |
| exo_circ_73483 | hsa_circ_0002114 | 1.062944 | 2.407812 | 2.265230028 | 0.017691 |
| exo_circ_20128 | hsa_circ_0006979 | 1.522689 | 3.448153 | 2.264516142 | 0.002617 |
| exo_circ_35236 | hsa_circ_0012152 | 1.378379 | 3.119537 | 2.263192058 | 0.003944 |
| exo_circ_13571 | hsa_circ_0005148 | 1.187447 | 2.687342 | 2.263126097 | 0.013683 |
| exo_circ_44298 | hsa_circ_0001102 | 1.011872 | 2.287901 | 2.261058665 | 0.016323 |
| exo_circ_08741 | hsa_circ_0000461 | 0.920687 | 2.080153 | 2.259348392 | 0.024895 |
| exo_circ_36617 | hsa_circ_0013081 | 0.972116 | 2.194392 | 2.257334556 | 0.019043 |
| exo_circ_71247 | hsa_circ_0008205 | 1.755912 | 3.960841 | 2.255716809 | 0.001455 |
| exo_circ_18094 | hsa_circ_0003863 | 1.713638 | 3.865144 | 2.255519109 | 0.001148 |
| exo_circ_60360 | hsa_circ_0072340 | 1.918497 | 4.321796 | 2.252698274 | 0.000529 |
| exo_circ_40737 | hsa_circ_0055855 | 0.832582 | 1.875357 | 2.252458129 | 0.038506 |
| exo_circ_26202 | hsa_circ_0000836 | 1.780446 | 4.009275 | 2.251837859 | 0.001211 |
| exo_circ_25694 | hsa_circ_0006264 | 1.769067 | 3.977878 | 2.248573601 | 0.000842 |
| exo_circ_18480 | hsa_circ_0002138 | 2.235034 | 5.025559 | 2.248538565 | 7.23E-05 |
| exo_circ_66141 | hsa_circ_0001621 | 1.135402 | 2.552803 | 2.248368757 | 0.009893 |
| exo_circ_11893 | hsa_circ_0002673 | 1.886998 | 4.24216 | 2.24810044 | 0.000947 |
| exo_circ_43968 | hsa_circ_0001095 | 1.73929 | 3.908768 | 2.24733589 | 0.00067 |
| exo_circ_04499 | hsa_circ_0005032 | 1.692766 | 3.802691 | 2.246436417 | 0.002052 |
| exo_circ_58871 | hsa_circ_0001542 | 0.874697 | 1.96474 | 2.246194077 | 0.031032 |
| exo_circ_70264 | hsa_circ_0081028 | 0.770678 | 1.728338 | 2.242619747 | 0.048512 |
| exo_circ_20922 | hsa_circ_0039161 | 1.524931 | 3.419437 | 2.242354707 | 0.003894 |
| exo_circ_70398 | hsa_circ_0004299 | 0.899335 | 2.016171 | 2.24184651 | 0.020692 |
| exo_circ_18219 | hsa_circ_0035435 | 1.127109 | 2.526044 | 2.241171326 | 0.015571 |
| exo_circ_70797 | hsa_circ_0005231 | 1.245267 | 2.785524 | 2.236889317 | 0.009421 |
| exo_circ_72138 | hsa_circ_0006302 | 2.333762 | 5.21492 | 2.234554798 | 4.12E-05 |
| exo_circ_65702 | hsa_circ_0077096 | 1.28082 | 2.860478 | 2.233317914 | 0.009083 |
| exo_circ_22087 | hsa_circ_0003026 | 0.781882 | 1.745911 | 2.23295874 | 0.04443 |
| exo_circ_51170 | hsa_circ_0001377 | 1.580586 | 3.528962 | 2.232691987 | 0.002369 |
| exo_circ_15168 | hsa_circ_0032047 | 1.132601 | 2.527851 | 2.231898056 | 0.018302 |
| exo_circ_13904 | hsa_circ_0005721 | 0.895225 | 1.997261 | 2.231016387 | 0.037772 |
| exo_circ_50597 | hsa_circ_0068135 | 2.353543 | 5.25055 | 2.230913434 | 9.72E-05 |
| exo_circ_47831 | hsa_circ_0001048 | 1.20859 | 2.694827 | 2.229728649 | 0.009434 |
| exo_circ_59254 | hsa_circ_0007773 | 2.32966 | 5.19395 | 2.229488649 | 0.000115 |
| exo_circ_58714 | hsa_circ_0004894 | 0.958153 | 2.135954 | 2.229241382 | 0.024206 |
| exo_circ_00157 | hsa_circ_0019701 | 0.66427 | 1.479933 | 2.227907262 | 0.047234 |
| exo_circ_37840 | hsa_circ_0060158 | 1.218568 | 2.709989 | 2.223913506 | 0.008781 |
| exo_circ_13853 | hsa_circ_0005791 | 1.605727 | 3.566464 | 2.221090139 | 0.002491 |
| exo_circ_18024 | hsa_circ_0035296 | 2.37784 | 5.279354 | 2.220231092 | 8.63E-05 |
| exo_circ_22360 | hsa_circ_0008996 | 1.026331 | 2.272586 | 2.214282437 | 0.022326 |
| exo_circ_32625 | hsa_circ_0000023 | 1.543075 | 3.411244 | 2.210679254 | 0.001212 |
| exo_circ_03686 | hsa_circ_0000251 | 1.211918 | 2.677667 | 2.209445352 | 0.014702 |
| exo_circ_75225 | hsa_circ_0006556 | 1.663059 | 3.67179 | 2.207853878 | 0.001496 |
| exo_circ_47913 | hsa_circ_0001051 | 2.529721 | 5.582303 | 2.206687581 | 1.05E-05 |
| exo_circ_42801 | hsa_circ_0006449 | 1.134273 | 2.501225 | 2.205135237 | 0.017981 |
| exo_circ_00282 | hsa_circ_0017438 | 1.669778 | 3.680205 | 2.204007858 | 0.002096 |
| exo_circ_53187 | hsa_circ_0004276 | 2.617766 | 5.758279 | 2.199692293 | 1.63E-05 |
| exo_circ_33642 | hsa_circ_0003314 | 0.888188 | 1.953117 | 2.19899101 | 0.049726 |
| exo_circ_36041 | hsa_circ_0004586 | 0.851622 | 1.870063 | 2.19588395 | 0.034032 |
| exo_circ_16950 | hsa_circ_0034293 | 2.524743 | 5.541234 | 2.194771644 | 5.83E-05 |
| exo_circ_76915 | hsa_circ_0087630 | 3.102291 | 6.807257 | 2.194268198 | 1.11E-06 |
| exo_circ_74817 | hsa_circ_0089282 | 1.131842 | 2.482717 | 2.193519132 | 0.018386 |
| exo_circ_15024 | hsa_circ_0006378 | 0.862566 | 1.891995 | 2.193450621 | 0.044663 |
| exo_circ_76249 | hsa_circ_0087234 | 1.318058 | 2.890057 | 2.192662125 | 0.012936 |
| exo_circ_46675 | hsa_circ_0001013 | 1.300679 | 2.851855 | 2.192588879 | 0.010792 |
| exo_circ_40609 | hsa_circ_0002077 | 2.739345 | 5.998037 | 2.189588178 | 1.01E-05 |
| exo_circ_62381 | hsa_circ_0005917 | 1.122184 | 2.457043 | 2.189518633 | 0.019642 |
| exo_circ_71935 | hsa_circ_0001785 | 3.906153 | 8.534967 | 2.185006077 | 5.48E-09 |
| exo_circ_06370 | hsa_circ_0002038 | 1.439085 | 3.143482 | 2.184362275 | 0.005426 |
| exo_circ_01095 | hsa_circ_0020523 | 0.81943 | 1.788113 | 2.182142173 | 0.048909 |
| exo_circ_71372 | hsa_circ_0007313 | 2.43561 | 5.304305 | 2.177813524 | 7.62E-05 |
| exo_circ_68842 | hsa_circ_0001699 | 2.389929 | 5.203288 | 2.177172765 | 0.000122 |
| exo_circ_04588 | hsa_circ_0008157 | 1.083768 | 2.359272 | 2.176915896 | 0.028628 |
| exo_circ_08734 | hsa_circ_0029308 | 2.788693 | 6.066894 | 2.175533256 | 8.44E-06 |
| exo_circ_51820 | hsa_circ_0007147 | 1.471126 | 3.197909 | 2.173783898 | 0.008082 |
| exo_circ_33526 | hsa_circ_0007822 | 1.823306 | 3.961908 | 2.172925594 | 0.001604 |
| exo_circ_05503 | hsa_circ_0003768 | 1.473964 | 3.201326 | 2.171915609 | 0.007081 |
| exo_circ_00080 | hsa_circ_0019611 | 1.739413 | 3.774748 | 2.170128096 | 0.0031 |
| exo_circ_44294 | hsa_circ_0005853 | 1.046527 | 2.270139 | 2.169213237 | 0.029123 |
| exo_circ_10619 | hsa_circ_0000413 | 0.900173 | 1.948882 | 2.165008999 | 0.041691 |
| exo_circ_40268 | hsa_circ_0004470 | 1.234708 | 2.671743 | 2.16386569 | 0.015126 |
| exo_circ_00071 | hsa_circ_0019607 | 2.906361 | 6.287558 | 2.163378438 | 5.39E-06 |
| exo_circ_38162 | hsa_circ_0060521 | 1.04888 | 2.264699 | 2.15916038 | 0.030665 |
| exo_circ_21050 | hsa_circ_0002500 | 1.080968 | 2.333828 | 2.159016921 | 0.019396 |
| exo_circ_31030 | hsa_circ_0010117 | 1.017523 | 2.196477 | 2.158651054 | 0.018829 |
| exo_circ_57604 | hsa_circ_0001425 | 2.572308 | 5.54835 | 2.156954508 | 9.21E-05 |
| exo_circ_63538 | hsa_circ_0078130 | 1.955976 | 4.218531 | 2.156739805 | 0.000775 |
| exo_circ_34821 | hsa_circ_0011571 | 1.321835 | 2.850401 | 2.156396348 | 0.011455 |
| exo_circ_59494 | hsa_circ_0001551 | 2.170561 | 4.677304 | 2.15488207 | 0.000266 |
| exo_circ_30991 | hsa_circ_0000021 | 3.05385 | 6.574485 | 2.152851436 | 4.13E-06 |
| exo_circ_16742 | hsa_circ_0005019 | 0.780053 | 1.675052 | 2.147357186 | 0.044317 |
| exo_circ_19743 | hsa_circ_0000651 | 3.132727 | 6.72458 | 2.146558153 | 2.56E-06 |
| exo_circ_77799 | hsa_circ_0001946 | 0.969627 | 2.078448 | 2.143553344 | 0.025049 |
| exo_circ_48471 | hsa_circ_0006498 | 0.982292 | 2.105305 | 2.14325819 | 0.041015 |
| exo_circ_40125 | hsa_circ_0062912 | 1.253707 | 2.686089 | 2.142516705 | 0.017095 |
| exo_circ_38757 | hsa_circ_0007609 | 2.719874 | 5.8227 | 2.140797623 | 2.23E-05 |
| exo_circ_75233 | hsa_circ_0086483 | 0.939195 | 2.009853 | 2.139974545 | 0.042489 |
| exo_circ_28110 | hsa_circ_0000907 | 3.164885 | 6.764278 | 2.137290157 | 1.75E-06 |
| exo_circ_33128 | hsa_circ_0016508 | 1.22316 | 2.614017 | 2.137101841 | 0.016715 |
| exo_circ_40542 | hsa_circ_0001240 | 1.622384 | 3.464138 | 2.135214209 | 0.00472 |
| exo_circ_56166 | hsa_circ_0006865 | 0.846645 | 1.806152 | 2.133304965 | 0.046004 |
| exo_circ_38093 | hsa_circ_0004359 | 1.737543 | 3.703503 | 2.131460279 | 0.003825 |
| exo_circ_60516 | hsa_circ_0005809 | 1.488364 | 3.170439 | 2.130150538 | 0.00762 |
| exo_circ_08316 | hsa_circ_0028899 | 3.534972 | 7.509484 | 2.124340159 | 7.35E-07 |
| exo_circ_16433 | hsa_circ_0032965 | 1.427028 | 3.029257 | 2.122773685 | 0.0118 |
| exo_circ_07454 | hsa_circ_0000373 | 1.669526 | 3.539592 | 2.12011782 | 0.004623 |
| exo_circ_23481 | hsa_circ_0004751 | 3.825002 | 8.109356 | 2.120091897 | 7.72E-08 |
| exo_circ_05461 | hsa_circ_0000287 | 1.422021 | 3.009329 | 2.116233301 | 0.011243 |
| exo_circ_55943 | hsa_circ_0001392 | 3.157105 | 6.681131 | 2.116220645 | 3.41E-06 |
| exo_circ_31261 | hsa_circ_0000155 | 1.822821 | 3.856624 | 2.115744787 | 0.002833 |
| exo_circ_51101 | hsa_circ_0002382 | 1.216645 | 2.571655 | 2.113726544 | 0.02377 |
| exo_circ_53509 | hsa_circ_0008027 | 1.424013 | 3.007757 | 2.112169244 | 0.006433 |
| exo_circ_74760 | hsa_circ_0007613 | 1.606433 | 3.39061 | 2.110645534 | 0.005204 |
| exo_circ_26922 | hsa_circ_0002501 | 1.701147 | 3.584366 | 2.107028166 | 0.005717 |
| exo_circ_74376 | hsa_circ_0002544 | 1.118381 | 2.356232 | 2.106823428 | 0.031676 |
| exo_circ_43714 | hsa_circ_0052867 | 0.949729 | 1.999456 | 2.105290387 | 0.040802 |
| exo_circ_24219 | hsa_circ_0044328 | 2.61072 | 5.495956 | 2.105149731 | 5.61E-05 |
| exo_circ_59539 | hsa_circ_0074944 | 1.974857 | 4.156806 | 2.104864091 | 0.002201 |
| exo_circ_08064 | hsa_circ_0002584 | 1.046075 | 2.201822 | 2.104842385 | 0.033648 |
| exo_circ_34520 | hsa_circ_0011168 | 1.036873 | 2.181492 | 2.103913889 | 0.040097 |
| exo_circ_74492 | hsa_circ_0001890 | 2.852177 | 5.99734 | 2.10272365 | 1.67E-05 |
| exo_circ_20742 | hsa_circ_0038704 | 2.990815 | 6.286699 | 2.102001971 | 1.38E-05 |
| exo_circ_40129 | hsa_circ_0062913 | 1.033135 | 2.168527 | 2.098976903 | 0.030232 |
| exo_circ_46310 | hsa_circ_0001002 | 1.049303 | 2.200654 | 2.097253596 | 0.038999 |
| exo_circ_16250 | hsa_circ_0003164 | 2.180499 | 4.571483 | 2.096530542 | 0.000616 |
| exo_circ_68273 | hsa_circ_0005251 | 2.906683 | 6.078239 | 2.091125415 | 5.81E-05 |
| exo_circ_40309 | hsa_circ_0005243 | 1.879396 | 3.928277 | 2.090180665 | 0.002867 |
| exo_circ_02520 | hsa_circ_0003351 | 1.33684 | 2.793 | 2.089255119 | 0.015381 |
| exo_circ_02560 | hsa_circ_0007403 | 1.165845 | 2.4344 | 2.088100155 | 0.023371 |
| exo_circ_51385 | hsa_circ_0002266 | 1.915457 | 3.997436 | 2.086935443 | 0.001186 |
| exo_circ_47604 | hsa_circ_0005315 | 2.635142 | 5.491268 | 2.083860321 | 0.000137 |
| exo_circ_59540 | hsa_circ_0074945 | 1.248113 | 2.600858 | 2.08383225 | 0.021249 |
| exo_circ_27585 | hsa_circ_0008435 | 1.922261 | 4.002115 | 2.081982712 | 0.001486 |
| exo_circ_26315 | hsa_circ_0047270 | 1.911417 | 3.978994 | 2.081698156 | 0.001978 |
| exo_circ_77743 | hsa_circ_0007717 | 2.635462 | 5.480422 | 2.079492224 | 0.000152 |
| exo_circ_77828 | hsa_circ_0001947 | 3.608138 | 7.491547 | 2.076291753 | 8.6E-07 |
| exo_circ_67573 | hsa_circ_0007178 | 2.599889 | 5.397755 | 2.076148491 | 0.000158 |
| exo_circ_52428 | hsa_circ_0008639 | 0.846781 | 1.757929 | 2.076013148 | 0.04763 |
| exo_circ_13322 | hsa_circ_0004217 | 2.240398 | 4.647044 | 2.074205097 | 0.00097 |
| exo_circ_41630 | hsa_circ_0001069 | 1.417707 | 2.940567 | 2.074171326 | 0.003405 |
| exo_circ_35142 | hsa_circ_0006848 | 1.599154 | 3.307894 | 2.068527088 | 0.004891 |
| exo_circ_38752 | hsa_circ_0002805 | 3.866251 | 7.99673 | 2.068342152 | 5.2E-07 |
| exo_circ_45233 | hsa_circ_0000985 | 3.258354 | 6.733621 | 2.066571176 | 9.56E-06 |
| exo_circ_08465 | hsa_circ_0029069 | 2.83483 | 5.850648 | 2.063844234 | 9.82E-05 |
| exo_circ_67132 | hsa_circ_0001746 | 2.2581 | 4.660268 | 2.063800451 | 0.000799 |
| exo_circ_72178 | hsa_circ_0083996 | 2.451896 | 5.058527 | 2.063108339 | 7.88E-05 |
| exo_circ_25985 | hsa_circ_0000831 | 3.721508 | 7.674064 | 2.062084584 | 7.34E-07 |
| exo_circ_27568 | hsa_circ_0048023 | 1.083208 | 2.233448 | 2.061882332 | 0.030133 |
| exo_circ_62376 | hsa_circ_0008514 | 1.238847 | 2.553529 | 2.061214717 | 0.026787 |
| exo_circ_60521 | hsa_circ_0006787 | 2.599501 | 5.356917 | 2.060748692 | 0.000187 |
| exo_circ_77896 | hsa_circ_0092135 | 2.096074 | 4.315291 | 2.058749694 | 0.001115 |
| exo_circ_32777 | hsa_circ_0000025 | 1.667308 | 3.431065 | 2.057847858 | 0.003173 |
| exo_circ_18544 | hsa_circ_0035803 | 0.994142 | 2.04487 | 2.056918897 | 0.040158 |
| exo_circ_20044 | hsa_circ_0037807 | 1.588683 | 3.26 | 2.052013789 | 0.006383 |
| exo_circ_20855 | hsa_circ_0037158 | 2.664581 | 5.461077 | 2.049506379 | 0.000186 |
| exo_circ_47538 | hsa_circ_0055451 | 1.014464 | 2.077449 | 2.047830025 | 0.044341 |
| exo_circ_24098 | hsa_circ_0002502 | 1.092383 | 2.236989 | 2.047807399 | 0.033895 |
| exo_circ_28913 | hsa_circ_0006698 | 1.196238 | 2.445951 | 2.044703122 | 0.028222 |
| exo_circ_00205 | hsa_circ_0019779 | 0.974182 | 1.991008 | 2.04377299 | 0.049946 |
| exo_circ_25803 | hsa_circ_0000817 | 1.511903 | 3.089955 | 2.043752626 | 0.009609 |
| exo_circ_52580 | hsa_circ_0004609 | 1.464638 | 2.991804 | 2.042691188 | 0.007855 |
| exo_circ_18762 | hsa_circ_0035923 | 1.184463 | 2.417302 | 2.040841306 | 0.03827 |
| exo_circ_03416 | hsa_circ_0018998 | 2.259288 | 4.608652 | 2.039869539 | 0.001305 |
| exo_circ_45048 | hsa_circ_0006934 | 1.221071 | 2.486845 | 2.036610054 | 0.036503 |
| exo_circ_30363 | hsa_circ_0003693 | 1.878477 | 3.824654 | 2.036039206 | 0.003654 |
| exo_circ_30864 | hsa_circ_0014611 | 1.090107 | 2.216813 | 2.033574212 | 0.037632 |
| exo_circ_07884 | hsa_circ_0002273 | 2.223443 | 4.520913 | 2.033293449 | 0.000844 |
| exo_circ_29485 | hsa_circ_0000886 | 2.69091 | 5.461144 | 2.029478718 | 4.82E-05 |
| exo_circ_54306 | hsa_circ_0009133 | 2.525608 | 5.120477 | 2.02742341 | 0.000417 |
| exo_circ_02266 | hsa_circ_0004915 | 2.091303 | 4.238006 | 2.026490942 | 0.003056 |
| exo_circ_35828 | hsa_circ_0000075 | 1.887227 | 3.820036 | 2.024152928 | 0.004105 |
| exo_circ_14149 | hsa_circ_0002301 | 1.266707 | 2.563228 | 2.02353573 | 0.026542 |
| exo_circ_76557 | hsa_circ_0001868 | 3.009215 | 6.084686 | 2.022017676 | 4.3E-05 |
| exo_circ_79050 | hsa_circ_0009024 | 4.667711 | 9.433432 | 2.020997529 | 2.19E-07 |
| exo_circ_73487 | hsa_circ_0002961 | 2.192516 | 4.43079 | 2.020870464 | 0.001745 |
| exo_circ_37354 | hsa_circ_0003298 | 2.840856 | 5.733051 | 2.018071847 | 0.00016 |
| exo_circ_33401 | hsa_circ_0016733 | 2.022745 | 4.077914 | 2.01602964 | 0.003432 |
| exo_circ_21015 | hsa_circ_0002439 | 3.041967 | 6.132522 | 2.015972263 | 2.61E-05 |
| exo_circ_03223 | hsa_circ_0017648 | 1.254261 | 2.527054 | 2.014775622 | 0.023615 |
| exo_circ_54254 | hsa_circ_0001386 | 3.030936 | 6.102437 | 2.013383828 | 9.33E-05 |
| exo_circ_31646 | hsa_circ_0005089 | 1.106376 | 2.225961 | 2.011939406 | 0.030865 |
| exo_circ_10823 | hsa_circ_0000417 | 2.521721 | 5.072259 | 2.01142715 | 0.000302 |
| exo_circ_63525 | hsa_circ_0001648 | 2.898732 | 5.828814 | 2.01081481 | 0.000101 |
| exo_circ_30383 | hsa_circ_0004220 | 1.838078 | 3.692232 | 2.008745706 | 0.006038 |
| exo_circ_08472 | hsa_circ_0000378 | 2.486301 | 4.98925 | 2.006695576 | 0.001002 |
| exo_circ_42417 | hsa_circ_0007024 | 1.176817 | 2.359122 | 2.004664143 | 0.034828 |
| exo_circ_44759 | hsa_circ_0006014 | 1.142402 | 2.288903 | 2.003589082 | 0.040724 |
| exo_circ_19171 | hsa_circ_0036351 | 1.383304 | 2.771403 | 2.003465931 | 0.02173 |
| exo_circ_38549 | hsa_circ_0060849 | 4.356363 | 8.71877 | 2.00138753 | 6.37E-08 |
| exo_circ_63388 | hsa_circ_0007769 | 1.260702 | 2.522375 | 2.000770789 | 0.032208 |
| exo_circ_39091 | hsa_circ_0007207 | 1.345264 | 2.689076 | 1.998920825 | 0.031043 |
| exo_circ_20079 | hsa_circ_0007846 | 2.088704 | 4.174291 | 1.99850722 | 0.003491 |
| exo_circ_52720 | hsa_circ_0002338 | 1.8596 | 3.710993 | 1.9955861 | 0.006951 |
| exo_circ_04965 | hsa_circ_0008967 | 2.278968 | 4.546642 | 1.995044021 | 0.001713 |
| exo_circ_74569 | hsa_circ_0088744 | 1.738633 | 3.468537 | 1.994979526 | 0.00213 |
| exo_circ_20755 | hsa_circ_0038728 | 1.667056 | 3.322478 | 1.993021272 | 0.009385 |
| exo_circ_71473 | hsa_circ_0003221 | 1.275089 | 2.541265 | 1.993009546 | 0.016234 |
| exo_circ_46795 | hsa_circ_0054853 | 1.757121 | 3.495175 | 1.989148341 | 0.01279 |
| exo_circ_43196 | hsa_circ_0002024 | 2.834822 | 5.636616 | 1.988348786 | 5.56E-05 |
| exo_circ_18026 | hsa_circ_0004773 | 1.619702 | 3.220292 | 1.988200582 | 0.014972 |
| exo_circ_21017 | hsa_circ_0005692 | 1.115404 | 2.216982 | 1.987605509 | 0.045242 |
| exo_circ_06448 | hsa_circ_0000337 | 2.029993 | 4.031793 | 1.986112439 | 0.002219 |
| exo_circ_03277 | hsa_circ_0009121 | 1.788236 | 3.549619 | 1.984983363 | 0.009234 |
| exo_circ_61340 | hsa_circ_0072954 | 2.68852 | 5.326957 | 1.98137187 | 0.000406 |
| exo_circ_72581 | hsa_circ_0084522 | 1.216504 | 2.408717 | 1.980031851 | 0.030019 |
| exo_circ_32779 | hsa_circ_0005604 | 2.282958 | 4.51942 | 1.979633028 | 0.001584 |
| exo_circ_55113 | hsa_circ_0071311 | 1.283031 | 2.539707 | 1.979458339 | 0.032979 |
| exo_circ_64254 | hsa_circ_0006107 | 1.452617 | 2.871374 | 1.976690864 | 0.021657 |
| exo_circ_11714 | hsa_circ_0000504 | 2.992587 | 5.915162 | 1.976604668 | 0.000114 |
| exo_circ_16414 | hsa_circ_0032959 | 0.904932 | 1.788083 | 1.975930037 | 0.034566 |
| exo_circ_29747 | hsa_circ_0004270 | 3.722397 | 7.351399 | 1.974910022 | 3.53E-06 |
| exo_circ_44660 | hsa_circ_0001115 | 2.327595 | 4.595403 | 1.974313544 | 0.002069 |
| exo_circ_62083 | hsa_circ_0073371 | 1.203972 | 2.375991 | 1.973460419 | 0.023671 |
| exo_circ_08571 | hsa_circ_0006615 | 1.399656 | 2.757567 | 1.970174903 | 0.029014 |
| exo_circ_35786 | hsa_circ_0000072 | 3.26875 | 6.439711 | 1.970083256 | 3.3E-05 |
| exo_circ_49462 | hsa_circ_0067620 | 2.4125 | 4.751001 | 1.969326509 | 0.001031 |
| exo_circ_61025 | hsa_circ_0001466 | 1.876462 | 3.692802 | 1.967960633 | 0.009761 |
| exo_circ_63115 | hsa_circ_0001641 | 1.613505 | 3.170572 | 1.965021219 | 0.010885 |
| exo_circ_60949 | hsa_circ_0072697 | 2.157761 | 4.236276 | 1.963273882 | 0.005104 |
| exo_circ_28755 | hsa_circ_0000936 | 1.907206 | 3.741994 | 1.962029061 | 0.003376 |
| exo_circ_27721 | hsa_circ_0006990 | 1.403667 | 2.754021 | 1.962018444 | 0.026477 |
| exo_circ_25342 | hsa_circ_0004392 | 2.421235 | 4.748911 | 1.961359051 | 0.001235 |
| exo_circ_39472 | hsa_circ_0061774 | 1.49485 | 2.928586 | 1.959116329 | 0.022862 |
| exo_circ_49874 | hsa_circ_0067774 | 1.350966 | 2.645763 | 1.958423395 | 0.029279 |
| exo_circ_10089 | hsa_circ_0026228 | 1.755785 | 3.437498 | 1.957812275 | 0.012445 |
| exo_circ_17693 | hsa_circ_0003784 | 1.918366 | 3.75557 | 1.957692508 | 0.005766 |
| exo_circ_22045 | hsa_circ_0040609 | 1.311716 | 2.565694 | 1.955982215 | 0.035179 |
| exo_circ_71478 | hsa_circ_0002483 | 3.963024 | 7.748857 | 1.955288825 | 2.29E-06 |
| exo_circ_05806 | hsa_circ_0000302 | 2.250827 | 4.399051 | 1.954415383 | 0.002814 |
| exo_circ_22190 | hsa_circ_0040831 | 1.748765 | 3.413235 | 1.951796981 | 0.006201 |
| exo_circ_21512 | hsa_circ_0002122 | 1.28255 | 2.503191 | 1.951729858 | 0.030539 |
| exo_circ_29514 | hsa_circ_0049083 | 1.684299 | 3.286916 | 1.951503379 | 0.015406 |
| exo_circ_11549 | hsa_circ_0000500 | 2.180823 | 4.252131 | 1.94978273 | 0.002413 |
| exo_circ_00896 | hsa_circ_0000267 | 3.908436 | 7.614114 | 1.948122677 | 2.01E-06 |
| exo_circ_61854 | hsa_circ_0073237 | 2.370075 | 4.610542 | 1.945314736 | 0.00272 |
| exo_circ_00286 | hsa_circ_0000205 | 2.810646 | 5.465705 | 1.944643972 | 0.000306 |
| exo_circ_57321 | hsa_circ_0003549 | 2.164311 | 4.202645 | 1.941793402 | 0.004044 |
| exo_circ_78730 | hsa_circ_0091072 | 2.351858 | 4.56667 | 1.941728672 | 0.001959 |
| exo_circ_20398 | hsa_circ_0003559 | 3.786524 | 7.350013 | 1.941097638 | 4.36E-06 |
| exo_circ_51283 | hsa_circ_0003036 | 2.279577 | 4.421449 | 1.939591867 | 0.001869 |
| exo_circ_00926 | hsa_circ_0000268 | 3.689151 | 7.145443 | 1.936880313 | 2.43E-05 |
| exo_circ_62426 | hsa_circ_0009144 | 1.447269 | 2.802205 | 1.93620235 | 0.027361 |
| exo_circ_24957 | hsa_circ_0045096 | 2.424551 | 4.687172 | 1.933212857 | 0.002118 |
| exo_circ_39145 | hsa_circ_0001185 | 1.871279 | 3.613864 | 1.931226688 | 0.010999 |
| exo_circ_23490 | hsa_circ_0006692 | 1.918672 | 3.703711 | 1.930351503 | 0.01051 |
| exo_circ_13942 | hsa_circ_0000523 | 3.401912 | 6.564432 | 1.929630028 | 2.64E-05 |
| exo_circ_68977 | hsa_circ_0001707 | 3.982198 | 7.672562 | 1.926715097 | 1.05E-05 |
| exo_circ_58942 | hsa_circ_0074362 | 1.989818 | 3.831454 | 1.925529812 | 0.007304 |
| exo_circ_04750 | hsa_circ_0002100 | 2.518415 | 4.845493 | 1.924024749 | 0.000445 |
| exo_circ_78984 | hsa_circ_0008297 | 2.917635 | 5.613349 | 1.923937867 | 0.000792 |
| exo_circ_68008 | hsa_circ_0083176 | 1.638178 | 3.146833 | 1.920934213 | 0.016422 |
| exo_circ_28886 | hsa_circ_0008590 | 2.020029 | 3.875611 | 1.918592237 | 0.00799 |
| exo_circ_09932 | hsa_circ_0000397 | 2.4275 | 4.65531 | 1.917738813 | 0.001999 |
| exo_circ_24733 | hsa_circ_0005077 | 2.273335 | 4.359013 | 1.917452966 | 0.0038 |
| exo_circ_64779 | hsa_circ_0076125 | 1.859209 | 3.561973 | 1.915854673 | 0.005055 |
| exo_circ_50720 | hsa_circ_0064555 | 3.337772 | 6.385297 | 1.913041843 | 0.000112 |
| exo_circ_10677 | hsa_circ_0008381 | 3.122853 | 5.974108 | 1.913028608 | 0.000227 |
| exo_circ_35829 | hsa_circ_0005354 | 1.740226 | 3.327688 | 1.912216522 | 0.01509 |
| exo_circ_24588 | hsa_circ_0000787 | 1.667065 | 3.187233 | 1.911883421 | 0.022035 |
| exo_circ_22243 | hsa_circ_0003846 | 1.405911 | 2.687503 | 1.911574279 | 0.02906 |
| exo_circ_24197 | hsa_circ_0004622 | 2.015948 | 3.853013 | 1.91126626 | 0.007636 |
| exo_circ_72123 | hsa_circ_0005630 | 1.810578 | 3.459299 | 1.910604943 | 0.012681 |
| exo_circ_27657 | hsa_circ_0003652 | 2.7515 | 5.256133 | 1.910279394 | 0.000353 |
| exo_circ_64337 | hsa_circ_0075792 | 2.312071 | 4.416098 | 1.910018307 | 0.00346 |
| exo_circ_74257 | hsa_circ_0004688 | 1.718002 | 3.27818 | 1.908135039 | 0.019122 |
| exo_circ_39442 | hsa_circ_0008452 | 1.271414 | 2.425818 | 1.907969447 | 0.043777 |
| exo_circ_67156 | hsa_circ_0001973 | 1.37451 | 2.621082 | 1.906920799 | 0.03788 |
| exo_circ_27599 | hsa_circ_0006114 | 3.53164 | 6.732672 | 1.906386993 | 4.72E-05 |
| exo_circ_52383 | hsa_circ_0065243 | 3.230285 | 6.157506 | 1.906180317 | 5.91E-05 |
| exo_circ_33099 | hsa_circ_0007739 | 1.787897 | 3.407274 | 1.905743822 | 0.019505 |
| exo_circ_06394 | hsa_circ_0001968 | 2.416037 | 4.597452 | 1.902889714 | 0.003167 |
| exo_circ_60321 | hsa_circ_0006308 | 2.408041 | 4.57191 | 1.898601111 | 0.003352 |
| exo_circ_70928 | hsa_circ_0085438 | 1.713788 | 3.253095 | 1.89819062 | 0.015716 |
| exo_circ_17109 | hsa_circ_0034414 | 2.617357 | 4.965513 | 1.897147677 | 0.001464 |
| exo_circ_09912 | hsa_circ_0025967 | 3.346034 | 6.335732 | 1.893504909 | 0.000187 |
| exo_circ_12096 | hsa_circ_0029762 | 2.322729 | 4.395857 | 1.892539323 | 0.003323 |
| exo_circ_30472 | hsa_circ_0003952 | 1.950661 | 3.691319 | 1.892342184 | 0.011814 |
| exo_circ_34018 | hsa_circ_0000199 | 2.126437 | 4.023716 | 1.892233636 | 0.008164 |
| exo_circ_65968 | hsa_circ_0001617 | 1.606577 | 3.038235 | 1.891123645 | 0.03009 |
| exo_circ_61677 | hsa_circ_0001503 | 2.369235 | 4.47841 | 1.890234661 | 0.004112 |
| exo_circ_36293 | hsa_circ_0008129 | 2.332122 | 4.405276 | 1.888955862 | 0.003448 |
| exo_circ_35788 | hsa_circ_0002316 | 4.017661 | 7.58181 | 1.887120262 | 1.22E-05 |
| exo_circ_46691 | hsa_circ_0005338 | 1.321091 | 2.489768 | 1.884629529 | 0.045774 |
| exo_circ_04979 | hsa_circ_0004429 | 2.040916 | 3.846035 | 1.884465171 | 0.011952 |
| exo_circ_40513 | hsa_circ_0001236 | 3.004356 | 5.659537 | 1.883777144 | 0.000412 |
| exo_circ_01609 | hsa_circ_0006063 | 3.242783 | 6.107317 | 1.883356676 | 0.000252 |
| exo_circ_40938 | hsa_circ_0006965 | 4.020114 | 7.564543 | 1.88167395 | 9.02E-06 |
| exo_circ_76038 | hsa_circ_0002359 | 3.033742 | 5.702258 | 1.879612071 | 0.000358 |
| exo_circ_51402 | hsa_circ_0006040 | 2.761625 | 5.185305 | 1.877628332 | 0.001334 |
| exo_circ_15894 | hsa_circ_0002564 | 2.757446 | 5.174697 | 1.876626824 | 0.001178 |
| exo_circ_42776 | hsa_circ_0007967 | 2.644321 | 4.953365 | 1.8732092 | 0.001344 |
| exo_circ_25061 | hsa_circ_0045272 | 2.449589 | 4.585479 | 1.871938002 | 0.003849 |
| exo_circ_76474 | hsa_circ_0001865 | 3.378315 | 6.32088 | 1.871015856 | 8.13E-05 |
| exo_circ_59652 | hsa_circ_0001555 | 3.008021 | 5.626413 | 1.870470219 | 0.000795 |
| exo_circ_53093 | hsa_circ_0007761 | 3.864999 | 7.229293 | 1.870451626 | 1.79E-05 |
| exo_circ_72672 | hsa_circ_0084582 | 2.466928 | 4.608113 | 1.86795628 | 0.004583 |
| exo_circ_38123 | hsa_circ_0006332 | 1.670151 | 3.119399 | 1.867734285 | 0.024117 |
| exo_circ_28193 | hsa_circ_0000914 | 3.716679 | 6.924673 | 1.863134348 | 3.02E-05 |
| exo_circ_23548 | hsa_circ_0043297 | 2.087455 | 3.888391 | 1.862742227 | 0.009013 |
| exo_circ_68447 | hsa_circ_0006773 | 2.086414 | 3.884184 | 1.861655399 | 0.009484 |
| exo_circ_58151 | hsa_circ_0004509 | 1.527377 | 2.838673 | 1.858527503 | 0.043683 |
| exo_circ_04888 | hsa_circ_0000370 | 2.320517 | 4.309546 | 1.857148982 | 0.004475 |
| exo_circ_44614 | hsa_circ_0003585 | 1.635225 | 3.036571 | 1.856974083 | 0.02735 |
| exo_circ_68342 | hsa_circ_0001686 | 2.107682 | 3.912504 | 1.856306413 | 0.008982 |
| exo_circ_53684 | hsa_circ_0001432 | 2.859978 | 5.304993 | 1.85490724 | 0.001086 |
| exo_circ_57292 | hsa_circ_0003451 | 2.403326 | 4.455611 | 1.853935742 | 0.005166 |
| exo_circ_69885 | hsa_circ_0001676 | 3.751246 | 6.95176 | 1.853186702 | 7.22E-05 |
| exo_circ_05465 | hsa_circ_0004851 | 2.12991 | 3.946442 | 1.85286803 | 0.008769 |
| exo_circ_37752 | hsa_circ_0001146 | 1.429879 | 2.647891 | 1.851828622 | 0.044595 |
| exo_circ_61663 | hsa_circ_0006916 | 1.590643 | 2.943405 | 1.850450161 | 0.035778 |
| exo_circ_32646 | hsa_circ_0002238 | 1.697462 | 3.139459 | 1.849502145 | 0.024321 |
| exo_circ_64187 | hsa_circ_0004558 | 1.916843 | 3.540624 | 1.847111891 | 0.013584 |
| exo_circ_52474 | hsa_circ_0065301 | 1.69393 | 3.127903 | 1.84653626 | 0.02456 |
| exo_circ_19938 | hsa_circ_0000660 | 2.262691 | 4.168982 | 1.842488454 | 0.007985 |
| exo_circ_64658 | hsa_circ_0001571 | 3.42022 | 6.301156 | 1.842324901 | 0.000135 |
| exo_circ_74196 | hsa_circ_0001885 | 3.503214 | 6.439288 | 1.838108719 | 0.000248 |
| exo_circ_52299 | hsa_circ_0004692 | 1.796899 | 3.291671 | 1.83186162 | 0.010749 |
| exo_circ_75586 | hsa_circ_0001846 | 2.332781 | 4.268444 | 1.829766001 | 0.006672 |
| exo_circ_00324 | hsa_circ_0003357 | 3.008188 | 5.502186 | 1.829069566 | 0.00113 |
| exo_circ_15095 | hsa_circ_0032029 | 1.491904 | 2.727774 | 1.828384471 | 0.043169 |
| exo_circ_00063 | hsa_circ_0000255 | 1.829447 | 3.341568 | 1.826545254 | 0.018761 |
| exo_circ_61365 | hsa_circ_0003571 | 3.544639 | 6.472343 | 1.82595241 | 0.000263 |
| exo_circ_16148 | hsa_circ_0007332 | 1.67735 | 3.060162 | 1.824402453 | 0.037344 |
| exo_circ_26314 | hsa_circ_0000839 | 3.51965 | 6.417377 | 1.823299844 | 0.000233 |
| exo_circ_32834 | hsa_circ_0007234 | 1.664189 | 3.033727 | 1.822945681 | 0.035638 |
| exo_circ_30223 | hsa_circ_0006188 | 1.841108 | 3.354654 | 1.822084714 | 0.029474 |
| exo_circ_31881 | hsa_circ_0005907 | 1.985315 | 3.614006 | 1.820369175 | 0.012345 |
| exo_circ_45192 | hsa_circ_0008257 | 2.019147 | 3.669506 | 1.81735436 | 0.014883 |
| exo_circ_31363 | hsa_circ_0015211 | 2.769252 | 5.030913 | 1.816704816 | 0.001436 |
| exo_circ_50917 | hsa_circ_0006248 | 3.096871 | 5.622944 | 1.815685231 | 0.001257 |
| exo_circ_01230 | hsa_circ_0006665 | 3.662751 | 6.639813 | 1.812794101 | 7.83E-05 |
| exo_circ_75619 | hsa_circ_0086724 | 2.882013 | 5.219614 | 1.811100089 | 0.002301 |
| exo_circ_58857 | hsa_circ_0001540 | 2.621854 | 4.74657 | 1.810386494 | 0.005855 |
| exo_circ_68269 | hsa_circ_0008951 | 3.006789 | 5.428317 | 1.805353428 | 0.001969 |
| exo_circ_43621 | hsa_circ_0005078 | 2.798 | 5.04089 | 1.801605058 | 0.003645 |
| exo_circ_24232 | hsa_circ_0002069 | 2.893045 | 5.207797 | 1.800109314 | 0.00236 |
| exo_circ_04887 | hsa_circ_0000369 | 3.162499 | 5.686059 | 1.797963616 | 0.000484 |
| exo_circ_27453 | hsa_circ_0002895 | 2.165983 | 3.890069 | 1.795983487 | 0.01483 |
| exo_circ_13685 | hsa_circ_0003831 | 1.618451 | 2.906343 | 1.795755896 | 0.035562 |
| exo_circ_04161 | hsa_circ_0006868 | 3.068818 | 5.507217 | 1.794572987 | 0.001562 |
| exo_circ_37835 | hsa_circ_0003322 | 2.532586 | 4.541917 | 1.79339068 | 0.005916 |
| exo_circ_08784 | hsa_circ_0006689 | 2.310709 | 4.135676 | 1.789786349 | 0.009967 |
| exo_circ_36360 | hsa_circ_0000087 | 2.236774 | 3.998785 | 1.78774626 | 0.014696 |
| exo_circ_33877 | hsa_circ_0004039 | 1.872476 | 3.343524 | 1.78561633 | 0.02836 |
| exo_circ_66681 | hsa_circ_0079440 | 1.727539 | 3.078904 | 1.782248115 | 0.040515 |
| exo_circ_34460 | hsa_circ_0011137 | 1.548959 | 2.760293 | 1.78203094 | 0.043739 |
| exo_circ_77862 | hsa_circ_0001948 | 1.855461 | 3.305999 | 1.781766783 | 0.02433 |
| exo_circ_52313 | hsa_circ_0002569 | 3.21345 | 5.725341 | 1.781680629 | 0.000891 |
| exo_circ_12219 | hsa_circ_0008902 | 3.617119 | 6.437821 | 1.779820354 | 0.000308 |
| exo_circ_65919 | hsa_circ_0008093 | 1.723219 | 3.063616 | 1.777844575 | 0.039577 |
| exo_circ_28911 | hsa_circ_0004440 | 2.259339 | 4.014786 | 1.776973519 | 0.011914 |
| exo_circ_12397 | hsa_circ_0004494 | 1.745897 | 3.100553 | 1.775908508 | 0.036192 |
| exo_circ_39379 | hsa_circ_0006062 | 3.951965 | 7.013903 | 1.774788703 | 4.47E-05 |
| exo_circ_62149 | hsa_circ_0008417 | 2.893834 | 5.118733 | 1.768840977 | 0.001196 |
| exo_circ_59793 | hsa_circ_0002972 | 2.820694 | 4.986003 | 1.767651261 | 0.00225 |
| exo_circ_49922 | hsa_circ_0006999 | 1.837805 | 3.245002 | 1.765694509 | 0.043565 |
| exo_circ_57103 | hsa_circ_0070039 | 4.224342 | 7.454894 | 1.764746558 | 7.67E-05 |
| exo_circ_59492 | hsa_circ_0001550 | 4.351792 | 7.67976 | 1.764734834 | 1.39E-05 |
| exo_circ_73089 | hsa_circ_0084781 | 2.879164 | 5.080297 | 1.764504128 | 0.002636 |
| exo_circ_34699 | hsa_circ_0011424 | 2.079028 | 3.66655 | 1.763588984 | 0.01772 |
| exo_circ_08785 | hsa_circ_0000462 | 2.839456 | 5.00671 | 1.76326397 | 0.003626 |
| exo_circ_27079 | hsa_circ_0000852 | 3.245301 | 5.718386 | 1.762050664 | 0.00114 |
| exo_circ_64646 | hsa_circ_0006109 | 3.493731 | 6.153569 | 1.76131766 | 0.000356 |
| exo_circ_04525 | hsa_circ_0007827 | 4.01498 | 7.066035 | 1.759917841 | 0.000138 |
| exo_circ_64032 | hsa_circ_0004587 | 2.664201 | 4.688008 | 1.759630398 | 0.00545 |
| exo_circ_45750 | hsa_circ_0054034 | 1.188802 | 2.091297 | 1.759163748 | 0.048569 |
| exo_circ_03408 | hsa_circ_0006956 | 2.077158 | 3.652409 | 1.758368643 | 0.025958 |
| exo_circ_50554 | hsa_circ_0001364 | 2.074813 | 3.646185 | 1.757356009 | 0.026567 |
| exo_circ_51162 | hsa_circ_0008267 | 3.862653 | 6.786001 | 1.756823937 | 0.000134 |
| exo_circ_06727 | hsa_circ_0004593 | 3.127561 | 5.491951 | 1.755985391 | 0.001443 |
| exo_circ_66435 | hsa_circ_0007395 | 2.575861 | 4.518785 | 1.754281355 | 0.009652 |
| exo_circ_68989 | hsa_circ_0001708 | 2.914266 | 5.109762 | 1.753361518 | 0.004139 |
| exo_circ_17328 | hsa_circ_0000592 | 3.580265 | 6.273039 | 1.752116176 | 0.000953 |
| exo_circ_49132 | hsa_circ_0067480 | 1.940921 | 3.399304 | 1.751387273 | 0.032605 |
| exo_circ_05550 | hsa_circ_0005044 | 3.052398 | 5.340338 | 1.749555023 | 0.003049 |
| exo_circ_34685 | hsa_circ_0011399 | 4.189186 | 7.327181 | 1.749070285 | 8.71E-05 |
| exo_circ_50066 | hsa_circ_0067841 | 1.718715 | 3.003453 | 1.747499303 | 0.042964 |
| exo_circ_53641 | hsa_circ_0002782 | 1.834719 | 3.201793 | 1.745113794 | 0.040217 |
| exo_circ_48151 | hsa_circ_0001325 | 1.669305 | 2.912016 | 1.744447534 | 0.03634 |
| exo_circ_04766 | hsa_circ_0003302 | 5.089278 | 8.860565 | 1.741025965 | 1.54E-06 |
| exo_circ_04748 | hsa_circ_0009021 | 3.132577 | 5.453462 | 1.740887196 | 0.002812 |
| exo_circ_58967 | hsa_circ_0074368 | 2.131791 | 3.708378 | 1.739559542 | 0.028284 |
| exo_circ_03405 | hsa_circ_0018992 | 2.134706 | 3.712005 | 1.738883601 | 0.030945 |
| exo_circ_14153 | hsa_circ_0031485 | 2.826149 | 4.913955 | 1.738745959 | 0.002934 |
| exo_circ_20715 | hsa_circ_0004260 | 2.06055 | 3.581643 | 1.73819716 | 0.028521 |
| exo_circ_65998 | hsa_circ_0004058 | 4.025568 | 6.994686 | 1.737564994 | 0.0002 |
| exo_circ_68141 | hsa_circ_0005535 | 1.768677 | 3.073156 | 1.737544839 | 0.039888 |
| exo_circ_21912 | hsa_circ_0003315 | 2.349572 | 4.07685 | 1.735145843 | 0.016125 |
| exo_circ_28945 | hsa_circ_0003356 | 3.273422 | 5.676244 | 1.734039624 | 0.001278 |
| exo_circ_72422 | hsa_circ_0084188 | 1.80438 | 3.128567 | 1.733874052 | 0.043789 |
| exo_circ_57328 | hsa_circ_0006618 | 2.03364 | 3.515521 | 1.728683742 | 0.024427 |
| exo_circ_68309 | hsa_circ_0001684 | 3.729868 | 6.443943 | 1.727659875 | 0.000531 |
| exo_circ_03440 | hsa_circ_0019005 | 2.373083 | 4.083711 | 1.720846102 | 0.020459 |
| exo_circ_74938 | hsa_circ_0004101 | 2.91067 | 5.006473 | 1.720041638 | 0.006896 |
| exo_circ_72921 | hsa_circ_0001807 | 2.389525 | 4.109477 | 1.71978859 | 0.015243 |
| exo_circ_56594 | hsa_circ_0001410 | 3.173861 | 5.454157 | 1.718461271 | 0.004273 |
| exo_circ_49609 | hsa_circ_0067716 | 1.971264 | 3.386063 | 1.717711269 | 0.040938 |
| exo_circ_72667 | hsa_circ_0007581 | 2.584249 | 4.437696 | 1.717209094 | 0.00938 |
| exo_circ_63005 | hsa_circ_0008833 | 3.911168 | 6.71162 | 1.716014262 | 0.00033 |
| exo_circ_51177 | hsa_circ_0002174 | 2.100061 | 3.602599 | 1.715473874 | 0.031016 |
| exo_circ_61697 | hsa_circ_0002260 | 2.557284 | 4.384874 | 1.714660443 | 0.014437 |
| exo_circ_27009 | hsa_circ_0007180 | 2.536994 | 4.344372 | 1.712409243 | 0.019071 |
| exo_circ_13912 | hsa_circ_0003643 | 2.953478 | 5.056843 | 1.712165317 | 0.003332 |
| exo_circ_04595 | hsa_circ_0007383 | 2.558767 | 4.378052 | 1.711000291 | 0.016742 |
| exo_circ_18669 | hsa_circ_0003526 | 2.288384 | 3.913431 | 1.710128716 | 0.021217 |
| exo_circ_78189 | hsa_circ_0007108 | 2.002942 | 3.419 | 1.706988886 | 0.038763 |
| exo_circ_18017 | hsa_circ_0035292 | 3.157022 | 5.388825 | 1.706933062 | 0.001688 |
| exo_circ_50331 | hsa_circ_0002387 | 3.42567 | 5.845369 | 1.706343011 | 0.002898 |
| exo_circ_25314 | hsa_circ_0005347 | 1.940198 | 3.302839 | 1.702320731 | 0.035739 |
| exo_circ_56655 | hsa_circ_0005912 | 2.659332 | 4.526595 | 1.702154888 | 0.005823 |
| exo_circ_43999 | hsa_circ_0001097 | 3.488642 | 5.93805 | 1.702109076 | 0.001243 |
| exo_circ_49226 | hsa_circ_0003823 | 2.600506 | 4.421865 | 1.70038617 | 0.010208 |
| exo_circ_09905 | hsa_circ_0004985 | 2.167083 | 3.683571 | 1.699782921 | 0.03159 |
| exo_circ_14977 | hsa_circ_0031939 | 2.393216 | 4.056611 | 1.695045848 | 0.022195 |
| exo_circ_63428 | hsa_circ_0009096 | 3.491128 | 5.909667 | 1.692767164 | 0.001538 |
| exo_circ_34942 | hsa_circ_0005807 | 1.969545 | 3.333245 | 1.692392916 | 0.043469 |
| exo_circ_49440 | hsa_circ_0067619 | 3.035173 | 5.133064 | 1.691193065 | 0.005302 |
| exo_circ_21729 | hsa_circ_0003438 | 2.359462 | 3.989539 | 1.690867671 | 0.02493 |
| exo_circ_25481 | hsa_circ_0003684 | 3.479179 | 5.86859 | 1.686774347 | 0.002432 |
| exo_circ_48431 | hsa_circ_0006884 | 3.197376 | 5.381299 | 1.683036351 | 0.004266 |
| exo_circ_55992 | hsa_circ_0069399 | 5.224232 | 8.790161 | 1.68257481 | 1.45E-05 |
| exo_circ_52904 | hsa_circ_0001315 | 4.559068 | 7.6707 | 1.682514901 | 6.77E-05 |
| exo_circ_63867 | hsa_circ_0001654 | 4.888278 | 8.217684 | 1.681099968 | 2.31E-05 |
| exo_circ_31880 | hsa_circ_0006869 | 2.588103 | 4.346719 | 1.679500328 | 0.016212 |
| exo_circ_12945 | hsa_circ_0030253 | 2.984269 | 5.010239 | 1.678883417 | 0.008791 |
| exo_circ_34345 | hsa_circ_0000033 | 2.437175 | 4.087196 | 1.677021568 | 0.018137 |
| exo_circ_24932 | hsa_circ_0002220 | 3.38998 | 5.684501 | 1.67685359 | 0.00236 |
| exo_circ_49316 | hsa_circ_0001344 | 2.154329 | 3.610282 | 1.675826887 | 0.035716 |
| exo_circ_28187 | hsa_circ_0005571 | 3.244365 | 5.434833 | 1.675160627 | 0.006075 |
| exo_circ_52176 | hsa_circ_0004089 | 4.789656 | 8.02032 | 1.67450856 | 1.42E-05 |
| exo_circ_27699 | hsa_circ_0007509 | 3.3948 | 5.681572 | 1.673610282 | 0.004229 |
| exo_circ_19924 | hsa_circ_0000657 | 2.175735 | 3.640778 | 1.673355261 | 0.033813 |
| exo_circ_13358 | hsa_circ_0000497 | 3.045016 | 5.092578 | 1.6724308 | 0.006717 |
| exo_circ_56217 | hsa_circ_0007308 | 2.092334 | 3.490064 | 1.668024091 | 0.014271 |
| exo_circ_33583 | hsa_circ_0000196 | 3.158463 | 5.26482 | 1.666893251 | 0.007523 |
| exo_circ_64041 | hsa_circ_0001663 | 4.590006 | 7.644542 | 1.665475124 | 0.000207 |
| exo_circ_37473 | hsa_circ_0002302 | 4.403321 | 7.332353 | 1.665187005 | 0.00019 |
| exo_circ_50272 | hsa_circ_0005228 | 3.240382 | 5.391157 | 1.663741353 | 0.006552 |
| exo_circ_17233 | hsa_circ_0004942 | 4.75221 | 7.894156 | 1.661154861 | 9.94E-05 |
| exo_circ_52688 | hsa_circ_0001307 | 2.434327 | 4.04049 | 1.659797517 | 0.020781 |
| exo_circ_67571 | hsa_circ_0006460 | 2.62892 | 4.356743 | 1.657237063 | 0.018787 |
| exo_circ_44432 | hsa_circ_0005689 | 2.23873 | 3.709633 | 1.65702556 | 0.034793 |
| exo_circ_05175 | hsa_circ_0000280 | 3.422281 | 5.668807 | 1.656440984 | 0.004743 |
| exo_circ_29292 | hsa_circ_0000881 | 4.636737 | 7.672111 | 1.654635812 | 0.000132 |
| exo_circ_09405 | hsa_circ_0000386 | 2.546805 | 4.207487 | 1.65206479 | 0.017026 |
| exo_circ_69497 | hsa_circ_0003261 | 2.513136 | 4.151734 | 1.652013713 | 0.013739 |
| exo_circ_63806 | hsa_circ_0003068 | 2.174441 | 3.592018 | 1.651926743 | 0.044103 |
| exo_circ_00061 | hsa_circ_0008362 | 3.65944 | 6.021021 | 1.645339583 | 0.002818 |
| exo_circ_71992 | hsa_circ_0007353 | 1.842128 | 3.029105 | 1.644350656 | 0.044854 |
| exo_circ_75831 | hsa_circ_0001861 | 3.709888 | 6.096628 | 1.64334552 | 0.001699 |
| exo_circ_05501 | hsa_circ_0021712 | 4.673567 | 7.66675 | 1.640449237 | 0.000142 |
| exo_circ_68271 | hsa_circ_0001971 | 3.368519 | 5.52158 | 1.639171459 | 0.00536 |
| exo_circ_08129 | hsa_circ_0000442 | 3.169384 | 5.193273 | 1.638574837 | 0.009284 |
| exo_circ_66462 | hsa_circ_0081819 | 2.320641 | 3.799721 | 1.637358333 | 0.019914 |
| exo_circ_46214 | hsa_circ_0000997 | 2.777173 | 4.541762 | 1.635390158 | 0.020564 |
| exo_circ_30305 | hsa_circ_0005986 | 2.505667 | 4.096529 | 1.634905567 | 0.026053 |
| exo_circ_18286 | hsa_circ_0000604 | 2.840123 | 4.642808 | 1.634720753 | 0.013174 |
| exo_circ_37654 | hsa_circ_0059957 | 2.842764 | 4.642035 | 1.632930098 | 0.019852 |
| exo_circ_55903 | hsa_circ_0006737 | 4.060012 | 6.61617 | 1.629593711 | 0.001524 |
| exo_circ_46212 | hsa_circ_0005542 | 2.74883 | 4.47893 | 1.629394953 | 0.018332 |
| exo_circ_06033 | hsa_circ_0002058 | 2.721245 | 4.427507 | 1.627014975 | 0.023189 |
| exo_circ_50640 | hsa_circ_0008550 | 2.325607 | 3.782563 | 1.626483918 | 0.034624 |
| exo_circ_43161 | hsa_circ_0001083 | 2.754036 | 4.474964 | 1.624874815 | 0.019199 |
| exo_circ_02316 | hsa_circ_0002968 | 2.817263 | 4.56835 | 1.621555932 | 0.016466 |
| exo_circ_01081 | hsa_circ_0007242 | 2.974053 | 4.822279 | 1.621450371 | 0.013564 |
| exo_circ_58325 | hsa_circ_0007067 | 2.254925 | 3.646538 | 1.617143774 | 0.044788 |
| exo_circ_63310 | hsa_circ_0004368 | 4.69872 | 7.588895 | 1.615098176 | 0.000287 |
| exo_circ_05444 | hsa_circ_0005974 | 3.751169 | 6.055523 | 1.614303079 | 0.002308 |
| exo_circ_27116 | hsa_circ_0047769 | 2.803676 | 4.525238 | 1.614037529 | 0.016058 |
| exo_circ_48434 | hsa_circ_0066875 | 2.287113 | 3.690733 | 1.613708291 | 0.038684 |
| exo_circ_39572 | hsa_circ_0001200 | 3.428443 | 5.518862 | 1.609728369 | 0.001664 |
| exo_circ_47085 | hsa_circ_0001023 | 2.551582 | 4.105244 | 1.608901528 | 0.031952 |
| exo_circ_12920 | hsa_circ_0004024 | 2.788321 | 4.484517 | 1.608322002 | 0.027201 |
| exo_circ_26196 | hsa_circ_0000835 | 5.890364 | 9.466462 | 1.607109979 | 9.78E-07 |
| exo_circ_07948 | hsa_circ_0006396 | 2.961941 | 4.754774 | 1.605289972 | 0.017669 |
| exo_circ_65700 | hsa_circ_0001614 | 2.993607 | 4.804463 | 1.604907793 | 0.018569 |
| exo_circ_65012 | hsa_circ_0002732 | 2.392067 | 3.839027 | 1.604899595 | 0.049416 |
| exo_circ_77422 | hsa_circ_0091382 | 4.113111 | 6.594655 | 1.603325147 | 0.000948 |
| exo_circ_36137 | hsa_circ_0006677 | 3.194162 | 5.119276 | 1.602697544 | 0.013097 |
| exo_circ_22354 | hsa_circ_0005603 | 2.122683 | 3.399125 | 1.601334226 | 0.045628 |
| exo_circ_21431 | hsa_circ_0000705 | 3.553394 | 5.688434 | 1.600844972 | 0.006272 |
| exo_circ_40868 | hsa_circ_0055945 | 2.903748 | 4.646131 | 1.600046393 | 0.020738 |
| exo_circ_58978 | hsa_circ_0074371 | 3.812502 | 6.090845 | 1.597597954 | 0.005217 |
| exo_circ_36246 | hsa_circ_0000085 | 3.979066 | 6.339939 | 1.593323171 | 0.002851 |
| exo_circ_38713 | hsa_circ_0003456 | 4.8782 | 7.769775 | 1.592754734 | 0.000333 |
| exo_circ_53347 | hsa_circ_0066536 | 3.93611 | 6.264598 | 1.591570881 | 0.003916 |
| exo_circ_67922 | hsa_circ_0002094 | 2.896924 | 4.599456 | 1.587703142 | 0.016806 |
| exo_circ_31910 | hsa_circ_0007905 | 4.439019 | 7.047156 | 1.58754802 | 0.0009 |
| exo_circ_60490 | hsa_circ_0005585 | 2.693684 | 4.270602 | 1.585412986 | 0.032189 |
| exo_circ_06827 | hsa_circ_0000346 | 2.781188 | 4.406531 | 1.58440584 | 0.028401 |
| exo_circ_21551 | hsa_circ_0005615 | 6.336017 | 10.03796 | 1.584269702 | 1.47E-06 |
| exo_circ_18536 | hsa_circ_0035796 | 5.174948 | 8.195703 | 1.583726805 | 8.85E-05 |
| exo_circ_08213 | hsa_circ_0000446 | 2.623628 | 4.151808 | 1.582468359 | 0.039372 |
| exo_circ_76805 | hsa_circ_0008193 | 2.40776 | 3.808136 | 1.581609372 | 0.038488 |
| exo_circ_72427 | hsa_circ_0002157 | 3.008583 | 4.757948 | 1.581458011 | 0.025747 |
| exo_circ_29907 | hsa_circ_0000106 | 5.299716 | 8.35825 | 1.577112638 | 8.84E-05 |
| exo_circ_68147 | hsa_circ_0005519 | 2.639057 | 4.158361 | 1.57569946 | 0.037425 |
| exo_circ_19897 | hsa_circ_0036997 | 3.452398 | 5.430983 | 1.573104758 | 0.011304 |
| exo_circ_70980 | hsa_circ_0085458 | 2.81768 | 4.429188 | 1.571927253 | 0.032673 |
| exo_circ_58592 | hsa_circ_0003154 | 2.285659 | 3.591008 | 1.571103747 | 0.029764 |
| exo_circ_74893 | hsa_circ_0001900 | 3.681063 | 5.76679 | 1.566609742 | 0.005123 |
| exo_circ_35650 | hsa_circ_0012553 | 4.636727 | 7.262809 | 1.566365369 | 0.000646 |
| exo_circ_32847 | hsa_circ_0002274 | 3.429319 | 5.368406 | 1.565443477 | 0.006382 |
| exo_circ_18543 | hsa_circ_0035801 | 3.260624 | 5.098437 | 1.563638366 | 0.017667 |
| exo_circ_59613 | hsa_circ_0002358 | 4.464634 | 6.972421 | 1.56170031 | 0.000931 |
| exo_circ_16647 | hsa_circ_0006717 | 2.851097 | 4.450852 | 1.561101552 | 0.035675 |
| exo_circ_01985 | hsa_circ_0000233 | 3.132199 | 4.879152 | 1.557740134 | 0.013832 |
| exo_circ_43215 | hsa_circ_0001085 | 3.78611 | 5.890156 | 1.555727459 | 0.007124 |
| exo_circ_48098 | hsa_circ_0003179 | 4.307291 | 6.689999 | 1.553180377 | 0.002327 |
| exo_circ_36653 | hsa_circ_0013093 | 2.826951 | 4.386423 | 1.551644069 | 0.041223 |
| exo_circ_71661 | hsa_circ_0002111 | 3.531858 | 5.478739 | 1.55123451 | 0.015115 |
| exo_circ_71637 | hsa_circ_0083465 | 4.494484 | 6.971526 | 1.551129529 | 0.002038 |
| exo_circ_28533 | hsa_circ_0005325 | 4.064481 | 6.281993 | 1.545582951 | 0.008596 |
| exo_circ_46888 | hsa_circ_0005552 | 2.880365 | 4.445636 | 1.543428108 | 0.042713 |
| exo_circ_04948 | hsa_circ_0002484 | 6.139626 | 9.475611 | 1.543353256 | 2.01E-05 |
| exo_circ_28245 | hsa_circ_0000918 | 5.432683 | 8.375179 | 1.541628579 | 0.000113 |
| exo_circ_32663 | hsa_circ_0005782 | 4.031115 | 6.213304 | 1.541336364 | 0.006128 |
| exo_circ_74467 | hsa_circ_0003270 | 4.735798 | 7.280885 | 1.537414701 | 0.001569 |
| exo_circ_05807 | hsa_circ_0000303 | 4.070159 | 6.255457 | 1.536907103 | 0.003562 |
| exo_circ_01901 | hsa_circ_0000231 | 3.294462 | 5.06138 | 1.536329842 | 0.014098 |
| exo_circ_12854 | hsa_circ_0004936 | 3.088637 | 4.74455 | 1.536130392 | 0.030968 |
| exo_circ_49328 | hsa_circ_0005372 | 2.970329 | 4.559752 | 1.535100042 | 0.040214 |
| exo_circ_26474 | hsa_circ_0004658 | 3.270513 | 5.018999 | 1.53462162 | 0.022232 |
| exo_circ_58858 | hsa_circ_0001541 | 3.615478 | 5.54705 | 1.534250666 | 0.013706 |
| exo_circ_57205 | hsa_circ_0070190 | 3.837764 | 5.873288 | 1.53039324 | 0.012477 |
| exo_circ_73793 | hsa_circ_0008812 | 4.114202 | 6.290201 | 1.528899366 | 0.005209 |
| exo_circ_78870 | hsa_circ_0001936 | 3.460608 | 5.290261 | 1.528708297 | 0.018667 |
| exo_circ_65005 | hsa_circ_0001603 | 4.137948 | 6.314893 | 1.526092761 | 0.008258 |
| exo_circ_73825 | hsa_circ_0087905 | 3.153118 | 4.810044 | 1.525488187 | 0.029814 |
| exo_circ_15220 | hsa_circ_0000542 | 3.313397 | 5.046708 | 1.52312208 | 0.02697 |
| exo_circ_37480 | hsa_circ_0001136 | 2.980965 | 4.538695 | 1.522558793 | 0.036086 |
| exo_circ_28194 | hsa_circ_0000915 | 3.054077 | 4.64696 | 1.521559605 | 0.029111 |
| exo_circ_60037 | hsa_circ_0001470 | 3.477311 | 5.290275 | 1.521369206 | 0.027442 |
| exo_circ_34458 | hsa_circ_0002909 | 5.39744 | 8.21073 | 1.521226745 | 0.000321 |
| exo_circ_04273 | hsa_circ_0003641 | 3.827787 | 5.819884 | 1.520430311 | 0.009711 |
| exo_circ_60502 | hsa_circ_0001481 | 6.0287 | 9.155188 | 1.518600672 | 7.4E-05 |
| exo_circ_07915 | hsa_circ_0002457 | 3.061279 | 4.634238 | 1.513823736 | 0.031914 |
| exo_circ_24282 | hsa_circ_0000782 | 3.941834 | 5.962418 | 1.512599859 | 0.011633 |
| exo_circ_07361 | hsa_circ_0006258 | 4.347482 | 6.573574 | 1.512041536 | 0.003716 |
| exo_circ_27698 | hsa_circ_0009022 | 4.725033 | 7.139374 | 1.510968149 | 0.002641 |
| exo_circ_09430 | hsa_circ_0007723 | 5.054016 | 7.633013 | 1.510286706 | 0.001562 |
| exo_circ_70415 | hsa_circ_0001726 | 3.112425 | 4.698735 | 1.509670203 | 0.035511 |
| exo_circ_65669 | hsa_circ_0077083 | 2.912325 | 4.394105 | 1.508796456 | 0.046829 |
| exo_circ_44762 | hsa_circ_0001116 | 5.359972 | 8.071339 | 1.505854626 | 0.000503 |
| exo_circ_76074 | hsa_circ_0001839 | 2.602654 | 3.914364 | 1.503989313 | 0.043328 |
| exo_circ_61562 | hsa_circ_0008620 | 4.904059 | 7.373705 | 1.503592339 | 0.00227 |
| exo_circ_13708 | hsa_circ_0002398 | 4.241994 | 6.369734 | 1.501589536 | 0.007713 |
