# Supplementary material for: CircVAPA promotes small cell lung cancer progression by modulating the miR-377-3p and miR-494-3p/IGF1R/AKT axis
Source: Mol Cancer. 2022 Jun 6;21:123. doi: 10.1186/s12943-022-01595-9 (PMC9172052; doi:10.1186/s12943-022-01595-9)
Supplement: Supplementary file 3 — Additional file 3: Table S2. The upregulated circRNAs previously reported based on circRNA profiling of six paired SCLC tissues. [file 12943_2022_1595_MOESM3_ESM.docx]

| **Table S2. The upregulated circRNAs previously reported based on circRNA profiling of six paired SCLC tissues.** | | | |
| --- | --- | --- | --- |
| **CircRNA ID** | **Gene** | **CircBase ID** | **Spliced length** |
| hsa_circRNA000750 | THRAP3 | None | chr1:36700678-36701591 |
| hsa_circRNA012605 | VAPA | hsa_circ_0006990 | chr18:9931806-9937063 |
| hsa_circRNA018206 | SATB1 | hsa_circ_0064557 | chr3:18456602-18462483 |
| hsa_circRNA018879 | STXBP5L | None | chr3:121097562-121100379 |
| hsa_circRNA020365 | SEC31A | hsa_circ_0070253 | chr4:83799882-83802075 |
