# Supplementary material for: CircVAPA promotes small cell lung cancer progression by modulating the miR-377-3p and miR-494-3p/IGF1R/AKT axis
Source: Mol Cancer. 2022 Jun 6;21:123. doi: 10.1186/s12943-022-01595-9 (PMC9172052; doi:10.1186/s12943-022-01595-9)
Supplement: Supplementary file 4 — Additional file 4: Table S3. The expression of circVAPA in serum samples. [file 12943_2022_1595_MOESM4_ESM.docx]

| **Table S3. The expression of circVAPA in serum samples.** | | | | |
| --- | --- | --- | --- | --- |
| **Sample** | **Type** | **Cohort** | **Sample size** | **Gene expression** |
| Benign001 | Blood | Benign | 130 | 0 |
| Benign002 | Blood | Benign | 130 | 0 |
| Benign003 | Blood | Benign | 130 | 0 |
| Benign004 | Blood | Benign | 130 | 0 |
| Benign005 | Blood | Benign | 130 | 254.966 |
| Benign006 | Blood | Benign | 130 | 0 |
| Benign007 | Blood | Benign | 130 | 0 |
| Benign008 | Blood | Benign | 130 | 0 |
| Benign009 | Blood | Benign | 130 | 0 |
| Benign010 | Blood | Benign | 130 | 190.684 |
| Benign011 | Blood | Benign | 130 | 0 |
| Benign012 | Blood | Benign | 130 | 0 |
| Benign013 | Blood | Benign | 130 | 0 |
| Benign014 | Blood | Benign | 130 | 0 |
| Benign015 | Blood | Benign | 130 | 0 |
| Benign016 | Blood | Benign | 130 | 7.045 |
| Benign017 | Blood | Benign | 130 | 0 |
| Benign018 | Blood | Benign | 130 | 13.025 |
| Benign019 | Blood | Benign | 130 | 18.56 |
| Benign020 | Blood | Benign | 130 | 79.333 |
| Benign021 | Blood | Benign | 130 | 0 |
| Benign022 | Blood | Benign | 130 | 619.446 |
| Benign023 | Blood | Benign | 130 | 0 |
| Benign024 | Blood | Benign | 130 | 97.179 |
| Benign025 | Blood | Benign | 130 | 77.899 |
| Benign026 | Blood | Benign | 130 | 111.899 |
| Benign027 | Blood | Benign | 130 | 32.503 |
| Benign028 | Blood | Benign | 130 | 0 |
| Benign029 | Blood | Benign | 130 | 0 |
| Benign030 | Blood | Benign | 130 | 119.576 |
| Benign031 | Blood | Benign | 130 | 0 |
| Benign032 | Blood | Benign | 130 | 0 |
| Benign033 | Blood | Benign | 130 | 0 |
| Benign034 | Blood | Benign | 130 | 0 |
| Benign035 | Blood | Benign | 130 | 0 |
| Benign036 | Blood | Benign | 130 | 0 |
| Benign037 | Blood | Benign | 130 | 0 |
| Benign038 | Blood | Benign | 130 | 0 |
| Benign039 | Blood | Benign | 130 | 0 |
| Benign040 | Blood | Benign | 130 | 0 |
| Benign041 | Blood | Benign | 130 | 0 |
| Benign042 | Blood | Benign | 130 | 0 |
| Benign043 | Blood | Benign | 130 | 0 |
| Benign044 | Blood | Benign | 130 | 0 |
| Benign045 | Blood | Benign | 130 | 0 |
| Benign046 | Blood | Benign | 130 | 0 |
| Benign047 | Blood | Benign | 130 | 0 |
| Benign048 | Blood | Benign | 130 | 32.296 |
| Benign049 | Blood | Benign | 130 | 0 |
| Benign050 | Blood | Benign | 130 | 0 |
| Benign051 | Blood | Benign | 130 | 0 |
| Benign052 | Blood | Benign | 130 | 0 |
| Benign053 | Blood | Benign | 130 | 0 |
| Benign054 | Blood | Benign | 130 | 0 |
| Benign055 | Blood | Benign | 130 | 0 |
| Benign056 | Blood | Benign | 130 | 0 |
| Benign057 | Blood | Benign | 130 | 14.22 |
| Benign058 | Blood | Benign | 130 | 0 |
| Benign059 | Blood | Benign | 130 | 0 |
| Benign060 | Blood | Benign | 130 | 0 |
| Benign061 | Blood | Benign | 130 | 0 |
| Benign062 | Blood | Benign | 130 | 0 |
| Benign063 | Blood | Benign | 130 | 0 |
| Benign064 | Blood | Benign | 130 | 0 |
| Benign065 | Blood | Benign | 130 | 0 |
| Benign066 | Blood | Benign | 130 | 0 |
| Benign067 | Blood | Benign | 130 | 0 |
| Benign068 | Blood | Benign | 130 | 0 |
| Benign069 | Blood | Benign | 130 | 0 |
| Benign070 | Blood | Benign | 130 | 0 |
| Benign071 | Blood | Benign | 130 | 651.903 |
| Benign072 | Blood | Benign | 130 | 0 |
| Benign073 | Blood | Benign | 130 | 0 |
| Benign074 | Blood | Benign | 130 | 0 |
| Benign075 | Blood | Benign | 130 | 0 |
| Benign076 | Blood | Benign | 130 | 0 |
| Benign077 | Blood | Benign | 130 | 0 |
| Benign078 | Blood | Benign | 130 | 55.012 |
| Benign079 | Blood | Benign | 130 | 0 |
| Benign080 | Blood | Benign | 130 | 0 |
| Benign081 | Blood | Benign | 130 | 0 |
| Benign082 | Blood | Benign | 130 | 0 |
| Benign083 | Blood | Benign | 130 | 0 |
| Benign084 | Blood | Benign | 130 | 0 |
| Benign085 | Blood | Benign | 130 | 0 |
| Benign086 | Blood | Benign | 130 | 0 |
| Benign087 | Blood | Benign | 130 | 0 |
| Benign088 | Blood | Benign | 130 | 0 |
| Benign089 | Blood | Benign | 130 | 0 |
| Benign090 | Blood | Benign | 130 | 0 |
| Benign091 | Blood | Benign | 130 | 0 |
| Benign092 | Blood | Benign | 130 | 0 |
| Benign093 | Blood | Benign | 130 | 563.863 |
| Benign094 | Blood | Benign | 130 | 0 |
| Benign095 | Blood | Benign | 130 | 0 |
| Benign096 | Blood | Benign | 130 | 0 |
| Benign097 | Blood | Benign | 130 | 0 |
| Benign098 | Blood | Benign | 130 | 0 |
| Benign099 | Blood | Benign | 130 | 0 |
| Benign100 | Blood | Benign | 130 | 0 |
| Benign101 | Blood | Benign | 130 | 0 |
| Benign102 | Blood | Benign | 130 | 0 |
| Benign103 | Blood | Benign | 130 | 0 |
| Benign104 | Blood | Benign | 130 | 227.505 |
| Benign105 | Blood | Benign | 130 | 0 |
| Benign106 | Blood | Benign | 130 | 0 |
| Benign107 | Blood | Benign | 130 | 0 |
| Benign108 | Blood | Benign | 130 | 0 |
| Benign109 | Blood | Benign | 130 | 0 |
| Benign110 | Blood | Benign | 130 | 14.183 |
| Benign111 | Blood | Benign | 130 | 0 |
| Benign112 | Blood | Benign | 130 | 0 |
| Benign113 | Blood | Benign | 130 | 0 |
| Benign114 | Blood | Benign | 130 | 0 |
| Benign115 | Blood | Benign | 130 | 0 |
| Benign116 | Blood | Benign | 130 | 0 |
| Benign117 | Blood | Benign | 130 | 21.235 |
| Benign118 | Blood | Benign | 130 | 0 |
| Benign119 | Blood | Benign | 130 | 0 |
| Benign120 | Blood | Benign | 130 | 0 |
| Benign121 | Blood | Benign | 130 | 0 |
| Benign122 | Blood | Benign | 130 | 0 |
| Benign123 | Blood | Benign | 130 | 0 |
| Benign124 | Blood | Benign | 130 | 210.416 |
| Benign125 | Blood | Benign | 130 | 0 |
| Benign126 | Blood | Benign | 130 | 0 |
| Benign127 | Blood | Benign | 130 | 0 |
| Benign128 | Blood | Benign | 130 | 0 |
| Benign129 | Blood | Benign | 130 | 0 |
| Benign130 | Blood | Benign | 130 | 0 |
| Bile01 | Bile | Bile | 15 | 0 |
| Bile02 | Bile | Bile | 15 | 0 |
| Bile03 | Bile | Bile | 15 | 0 |
| Bile04 | Bile | Bile | 15 | 0 |
| Bile05 | Bile | Bile | 15 | 0 |
| Bile06 | Bile | Bile | 15 | 0 |
| Bile07 | Bile | Bile | 15 | 0 |
| Bile08 | Bile | Bile | 15 | 0 |
| Bile09 | Bile | Bile | 15 | 0 |
| Bile10 | Bile | Bile | 15 | 0 |
| Bile11 | Bile | Bile | 15 | 0 |
| Bile12 | Bile | Bile | 15 | 0 |
| Bile13 | Bile | Bile | 15 | 0 |
| Bile14 | Bile | Bile | 15 | 0 |
| Bile15 | Bile | Bile | 15 | 0 |
| BRCA001 | Blood | BRCA | 140 | 0 |
| BRCA002 | Blood | BRCA | 140 | 0 |
| BRCA003 | Blood | BRCA | 140 | 0 |
| BRCA004 | Blood | BRCA | 140 | 0 |
| BRCA005 | Blood | BRCA | 140 | 0 |
| BRCA006 | Blood | BRCA | 140 | 0 |
| BRCA007 | Blood | BRCA | 140 | 0 |
| BRCA008 | Blood | BRCA | 140 | 0 |
| BRCA009 | Blood | BRCA | 140 | 172.698 |
| BRCA010 | Blood | BRCA | 140 | 417.611 |
| BRCA011 | Blood | BRCA | 140 | 321.52 |
| BRCA012 | Blood | BRCA | 140 | 0 |
| BRCA013 | Blood | BRCA | 140 | 20.662 |
| BRCA014 | Blood | BRCA | 140 | 0 |
| BRCA015 | Blood | BRCA | 140 | 0 |
| BRCA016 | Blood | BRCA | 140 | 0 |
| BRCA017 | Blood | BRCA | 140 | 391.321 |
| BRCA018 | Blood | BRCA | 140 | 0 |
| BRCA019 | Blood | BRCA | 140 | 0 |
| BRCA020 | Blood | BRCA | 140 | 178.606 |
| BRCA021 | Blood | BRCA | 140 | 0 |
| BRCA022 | Blood | BRCA | 140 | 9.501 |
| BRCA023 | Blood | BRCA | 140 | 0 |
| BRCA024 | Blood | BRCA | 140 | 0 |
| BRCA025 | Blood | BRCA | 140 | 0 |
| BRCA026 | Blood | BRCA | 140 | 461.882 |
| BRCA027 | Blood | BRCA | 140 | 0 |
| BRCA028 | Blood | BRCA | 140 | 361.297 |
| BRCA029 | Blood | BRCA | 140 | 0 |
| BRCA030 | Blood | BRCA | 140 | 0 |
| BRCA031 | Blood | BRCA | 140 | 6.089 |
| BRCA032 | Blood | BRCA | 140 | 0 |
| BRCA033 | Blood | BRCA | 140 | 0 |
| BRCA034 | Blood | BRCA | 140 | 0 |
| BRCA035 | Blood | BRCA | 140 | 0 |
| BRCA036 | Blood | BRCA | 140 | 0 |
| BRCA037 | Blood | BRCA | 140 | 4.851 |
| BRCA038 | Blood | BRCA | 140 | 0 |
| BRCA039 | Blood | BRCA | 140 | 0 |
| BRCA040 | Blood | BRCA | 140 | 0 |
| BRCA041 | Blood | BRCA | 140 | 4.022 |
| BRCA042 | Blood | BRCA | 140 | 115.343 |
| BRCA043 | Blood | BRCA | 140 | 137.237 |
| BRCA044 | Blood | BRCA | 140 | 0 |
| BRCA045 | Blood | BRCA | 140 | 182.041 |
| BRCA046 | Blood | BRCA | 140 | 0 |
| BRCA047 | Blood | BRCA | 140 | 0 |
| BRCA048 | Blood | BRCA | 140 | 307.164 |
| BRCA049 | Blood | BRCA | 140 | 0 |
| BRCA050 | Blood | BRCA | 140 | 7.028 |
| BRCA051 | Blood | BRCA | 140 | 0 |
| BRCA052 | Blood | BRCA | 140 | 0 |
| BRCA053 | Blood | BRCA | 140 | 0 |
| BRCA054 | Blood | BRCA | 140 | 86.395 |
| BRCA055 | Blood | BRCA | 140 | 687.149 |
| BRCA056 | Blood | BRCA | 140 | 0 |
| BRCA057 | Blood | BRCA | 140 | 0 |
| BRCA058 | Blood | BRCA | 140 | 44.241 |
| BRCA059 | Blood | BRCA | 140 | 0 |
| BRCA060 | Blood | BRCA | 140 | 0 |
| BRCA061 | Blood | BRCA | 140 | 0 |
| BRCA062 | Blood | BRCA | 140 | 0 |
| BRCA063 | Blood | BRCA | 140 | 0 |
| BRCA064 | Blood | BRCA | 140 | 0 |
| BRCA065 | Blood | BRCA | 140 | 0 |
| BRCA066 | Blood | BRCA | 140 | 0 |
| BRCA067 | Blood | BRCA | 140 | 0 |
| BRCA068 | Blood | BRCA | 140 | 0 |
| BRCA069 | Blood | BRCA | 140 | 0 |
| BRCA070 | Blood | BRCA | 140 | 175.431 |
| BRCA071 | Blood | BRCA | 140 | 0 |
| BRCA072 | Blood | BRCA | 140 | 0 |
| BRCA073 | Blood | BRCA | 140 | 0 |
| BRCA074 | Blood | BRCA | 140 | 0 |
| BRCA075 | Blood | BRCA | 140 | 78.704 |
| BRCA076 | Blood | BRCA | 140 | 0 |
| BRCA077 | Blood | BRCA | 140 | 202.382 |
| BRCA078 | Blood | BRCA | 140 | 0 |
| BRCA079 | Blood | BRCA | 140 | 0 |
| BRCA080 | Blood | BRCA | 140 | 151.525 |
| BRCA081 | Blood | BRCA | 140 | 0 |
| BRCA082 | Blood | BRCA | 140 | 415.864 |
| BRCA083 | Blood | BRCA | 140 | 78.101 |
| BRCA084 | Blood | BRCA | 140 | 0 |
| BRCA085 | Blood | BRCA | 140 | 0 |
| BRCA086 | Blood | BRCA | 140 | 0 |
| BRCA087 | Blood | BRCA | 140 | 0 |
| BRCA088 | Blood | BRCA | 140 | 135.205 |
| BRCA089 | Blood | BRCA | 140 | 0 |
| BRCA090 | Blood | BRCA | 140 | 103.842 |
| BRCA091 | Blood | BRCA | 140 | 0 |
| BRCA092 | Blood | BRCA | 140 | 59.715 |
| BRCA093 | Blood | BRCA | 140 | 0 |
| BRCA094 | Blood | BRCA | 140 | 12.039 |
| BRCA095 | Blood | BRCA | 140 | 0 |
| BRCA096 | Blood | BRCA | 140 | 0 |
| BRCA097 | Blood | BRCA | 140 | 17.429 |
| BRCA098 | Blood | BRCA | 140 | 0 |
| BRCA099 | Blood | BRCA | 140 | 0 |
| BRCA100 | Blood | BRCA | 140 | 0 |
| BRCA101 | Blood | BRCA | 140 | 0 |
| BRCA102 | Blood | BRCA | 140 | 160.393 |
| BRCA103 | Blood | BRCA | 140 | 0 |
| BRCA104 | Blood | BRCA | 140 | 0 |
| BRCA105 | Blood | BRCA | 140 | 0 |
| BRCA106 | Blood | BRCA | 140 | 0 |
| BRCA107 | Blood | BRCA | 140 | 0 |
| BRCA108 | Blood | BRCA | 140 | 307.093 |
| BRCA109 | Blood | BRCA | 140 | 16.962 |
| BRCA110 | Blood | BRCA | 140 | 0 |
| BRCA111 | Blood | BRCA | 140 | 7.301 |
| BRCA112 | Blood | BRCA | 140 | 608.735 |
| BRCA113 | Blood | BRCA | 140 | 0 |
| BRCA114 | Blood | BRCA | 140 | 0 |
| BRCA115 | Blood | BRCA | 140 | 0 |
| BRCA116 | Blood | BRCA | 140 | 0 |
| BRCA117 | Blood | BRCA | 140 | 23.575 |
| BRCA118 | Blood | BRCA | 140 | 158.054 |
| BRCA119 | Blood | BRCA | 140 | 965.65 |
| BRCA120 | Blood | BRCA | 140 | 216.921 |
| BRCA121 | Blood | BRCA | 140 | 0 |
| BRCA122 | Blood | BRCA | 140 | 212.589 |
| BRCA123 | Blood | BRCA | 140 | 0 |
| BRCA124 | Blood | BRCA | 140 | 339.053 |
| BRCA125 | Blood | BRCA | 140 | 0 |
| BRCA126 | Blood | BRCA | 140 | 0 |
| BRCA127 | Blood | BRCA | 140 | 0 |
| BRCA128 | Blood | BRCA | 140 | 0 |
| BRCA129 | Blood | BRCA | 140 | 0 |
| BRCA130 | Blood | BRCA | 140 | 0 |
| BRCA131 | Blood | BRCA | 140 | 0 |
| BRCA132 | Blood | BRCA | 140 | 0 |
| BRCA133 | Blood | BRCA | 140 | 0 |
| BRCA134 | Blood | BRCA | 140 | 0 |
| BRCA135 | Blood | BRCA | 140 | 0 |
| BRCA136 | Blood | BRCA | 140 | 0 |
| BRCA137 | Blood | BRCA | 140 | 0 |
| BRCA138 | Blood | BRCA | 140 | 56.097 |
| BRCA139 | Blood | BRCA | 140 | 0 |
| BRCA140 | Blood | BRCA | 140 | 0 |
| CHD01 | Blood | CHD | 12 | 0 |
| CHD02 | Blood | CHD | 12 | 87.462 |
| CHD03 | Blood | CHD | 12 | 0 |
| CHD04 | Blood | CHD | 12 | 0 |
| CHD05 | Blood | CHD | 12 | 0 |
| CHD06 | Blood | CHD | 12 | 0 |
| CHD07 | Blood | CHD | 12 | 0 |
| CHD08 | Blood | CHD | 12 | 0 |
| CHD09 | Blood | CHD | 12 | 0 |
| CHD10 | Blood | CHD | 12 | 0 |
| CHD11 | Blood | CHD | 12 | 0 |
| CHD12 | Blood | CHD | 12 | 0 |
| CRC01 | Blood | CRC | 35 | 0 |
| CRC02 | Blood | CRC | 35 | 39.892 |
| CRC03 | Blood | CRC | 35 | 0 |
| CRC04 | Blood | CRC | 35 | 48.296 |
| CRC05 | Blood | CRC | 35 | 0 |
| CRC06 | Blood | CRC | 35 | 50.225 |
| CRC07 | Blood | CRC | 35 | 11.985 |
| CRC08 | Blood | CRC | 35 | 322.606 |
| CRC09 | Blood | CRC | 35 | 0 |
| CRC10 | Blood | CRC | 35 | 6.983 |
| CRC11 | Blood | CRC | 35 | 338.458 |
| CRC12 | Blood | CRC | 35 | 8.681 |
| CRC13 | Blood | CRC | 35 | 144.623 |
| CRC14 | Blood | CRC | 35 | 0 |
| CRC15 | Blood | CRC | 35 | 343.868 |
| CRC16 | Blood | CRC | 35 | 0 |
| CRC17 | Blood | CRC | 35 | 0 |
| CRC18 | Blood | CRC | 35 | 149.03 |
| CRC19 | Blood | CRC | 35 | 179.5 |
| CRC20 | Blood | CRC | 35 | 609.411 |
| CRC21 | Blood | CRC | 35 | 65.433 |
| CRC22 | Blood | CRC | 35 | 0 |
| CRC23 | Blood | CRC | 35 | 0 |
| CRC24 | Blood | CRC | 35 | 0 |
| CRC25 | Blood | CRC | 35 | 71.454 |
| CRC26 | Blood | CRC | 35 | 0 |
| CRC27 | Blood | CRC | 35 | 0 |
| CRC28 | Blood | CRC | 35 | 0 |
| CRC29 | Blood | CRC | 35 | 0 |
| CRC30 | Blood | CRC | 35 | 53.221 |
| CRC31 | Blood | CRC | 35 | 198.397 |
| CRC32 | Blood | CRC | 35 | 0 |
| CRC33 | Blood | CRC | 35 | 0 |
| CRC34 | Blood | CRC | 35 | 19.215 |
| CRC35 | Blood | CRC | 35 | 0 |
| CSF1 | CSF | CSF | 5 | 0 |
| CSF2 | CSF | CSF | 5 | 0 |
| CSF3 | CSF | CSF | 5 | 0 |
| CSF4 | CSF | CSF | 5 | 0 |
| CSF5 | CSF | CSF | 5 | 0 |
| ESCC1 | Blood | ESCC | 6 | 0 |
| ESCC2 | Blood | ESCC | 6 | 0 |
| ESCC3 | Blood | ESCC | 6 | 0 |
| ESCC4 | Blood | ESCC | 6 | 0 |
| ESCC5 | Blood | ESCC | 6 | 0 |
| ESCC6 | Blood | ESCC | 6 | 1072.386 |
| GBM01 | Blood | GBM | 13 | 0 |
| GBM02 | Blood | GBM | 13 | 0 |
| GBM03 | Blood | GBM | 13 | 0 |
| GBM04 | Blood | GBM | 13 | 0 |
| GBM05 | Blood | GBM | 13 | 0 |
| GBM06 | Blood | GBM | 13 | 0 |
| GBM07 | Blood | GBM | 13 | 0 |
| GBM08 | Blood | GBM | 13 | 0 |
| GBM09 | Blood | GBM | 13 | 0 |
| GBM10 | Blood | GBM | 13 | 0 |
| GBM11 | Blood | GBM | 13 | 0 |
| GBM12 | Blood | GBM | 13 | 0 |
| GBM13 | Blood | GBM | 13 | 0 |
| GC1 | Blood | GC | 9 | 0 |
| GC2 | Blood | GC | 9 | 347.738 |
| GC3 | Blood | GC | 9 | 360.68 |
| GC4 | Blood | GC | 9 | 0 |
| GC5 | Blood | GC | 9 | 0 |
| GC6 | Blood | GC | 9 | 60.269 |
| GC7 | Blood | GC | 9 | 0 |
| GC8 | Blood | GC | 9 | 0 |
| GC9 | Blood | GC | 9 | 0 |
| HCC001 | Blood | HCC | 112 | 202.802 |
| HCC002 | Blood | HCC | 112 | 415.52 |
| HCC003 | Blood | HCC | 112 | 0 |
| HCC004 | Blood | HCC | 112 | 0 |
| HCC005 | Blood | HCC | 112 | 716.261 |
| HCC006 | Blood | HCC | 112 | 0 |
| HCC007 | Blood | HCC | 112 | 0 |
| HCC008 | Blood | HCC | 112 | 0 |
| HCC009 | Blood | HCC | 112 | 0 |
| HCC010 | Blood | HCC | 112 | 0 |
| HCC011 | Blood | HCC | 112 | 0 |
| HCC012 | Blood | HCC | 112 | 0 |
| HCC013 | Blood | HCC | 112 | 0 |
| HCC014 | Blood | HCC | 112 | 0 |
| HCC015 | Blood | HCC | 112 | 0 |
| HCC016 | Blood | HCC | 112 | 0 |
| HCC017 | Blood | HCC | 112 | 0 |
| HCC018 | Blood | HCC | 112 | 80.665 |
| HCC019 | Blood | HCC | 112 | 0 |
| HCC020 | Blood | HCC | 112 | 0 |
| HCC021 | Blood | HCC | 112 | 0 |
| HCC022 | Blood | HCC | 112 | 0 |
| HCC023 | Blood | HCC | 112 | 0 |
| HCC024 | Blood | HCC | 112 | 0 |
| HCC025 | Blood | HCC | 112 | 0 |
| HCC026 | Blood | HCC | 112 | 0 |
| HCC027 | Blood | HCC | 112 | 0 |
| HCC028 | Blood | HCC | 112 | 138.174 |
| HCC029 | Blood | HCC | 112 | 413.901 |
| HCC030 | Blood | HCC | 112 | 18.247 |
| HCC031 | Blood | HCC | 112 | 0 |
| HCC032 | Blood | HCC | 112 | 0 |
| HCC033 | Blood | HCC | 112 | 0 |
| HCC034 | Blood | HCC | 112 | 0 |
| HCC035 | Blood | HCC | 112 | 371.037 |
| HCC036 | Blood | HCC | 112 | 0 |
| HCC037 | Blood | HCC | 112 | 459.788 |
| HCC038 | Blood | HCC | 112 | 916.89 |
| HCC039 | Blood | HCC | 112 | 0 |
| HCC040 | Blood | HCC | 112 | 0 |
| HCC041 | Blood | HCC | 112 | 0 |
| HCC042 | Blood | HCC | 112 | 396.329 |
| HCC043 | Blood | HCC | 112 | 301.326 |
| HCC044 | Blood | HCC | 112 | 0 |
| HCC045 | Blood | HCC | 112 | 0 |
| HCC046 | Blood | HCC | 112 | 0 |
| HCC047 | Blood | HCC | 112 | 0 |
| HCC048 | Blood | HCC | 112 | 0 |
| HCC049 | Blood | HCC | 112 | 0 |
| HCC050 | Blood | HCC | 112 | 0 |
| HCC051 | Blood | HCC | 112 | 782.566 |
| HCC052 | Blood | HCC | 112 | 0 |
| HCC053 | Blood | HCC | 112 | 0 |
| HCC054 | Blood | HCC | 112 | 0 |
| HCC055 | Blood | HCC | 112 | 0 |
| HCC056 | Blood | HCC | 112 | 0 |
| HCC057 | Blood | HCC | 112 | 0 |
| HCC058 | Blood | HCC | 112 | 0 |
| HCC059 | Blood | HCC | 112 | 0 |
| HCC060 | Blood | HCC | 112 | 0 |
| HCC061 | Blood | HCC | 112 | 0 |
| HCC062 | Blood | HCC | 112 | 0 |
| HCC063 | Blood | HCC | 112 | 571.486 |
| HCC064 | Blood | HCC | 112 | 0 |
| HCC065 | Blood | HCC | 112 | 554.187 |
| HCC066 | Blood | HCC | 112 | 0 |
| HCC067 | Blood | HCC | 112 | 0 |
| HCC068 | Blood | HCC | 112 | 246.67 |
| HCC069 | Blood | HCC | 112 | 0 |
| HCC070 | Blood | HCC | 112 | 0 |
| HCC071 | Blood | HCC | 112 | 0 |
| HCC072 | Blood | HCC | 112 | 0 |
| HCC073 | Blood | HCC | 112 | 0 |
| HCC074 | Blood | HCC | 112 | 0 |
| HCC075 | Blood | HCC | 112 | 514.352 |
| HCC076 | Blood | HCC | 112 | 0 |
| HCC077 | Blood | HCC | 112 | 121.113 |
| HCC078 | Blood | HCC | 112 | 374.449 |
| HCC079 | Blood | HCC | 112 | 53.219 |
| HCC080 | Blood | HCC | 112 | 0 |
| HCC081 | Blood | HCC | 112 | 142.41 |
| HCC082 | Blood | HCC | 112 | 0 |
| HCC083 | Blood | HCC | 112 | 0 |
| HCC084 | Blood | HCC | 112 | 0 |
| HCC085 | Blood | HCC | 112 | 0 |
| HCC086 | Blood | HCC | 112 | 0 |
| HCC087 | Blood | HCC | 112 | 94.95 |
| HCC088 | Blood | HCC | 112 | 87.943 |
| HCC089 | Blood | HCC | 112 | 0 |
| HCC090 | Blood | HCC | 112 | 234.24 |
| HCC091 | Blood | HCC | 112 | 0 |
| HCC092 | Blood | HCC | 112 | 0 |
| HCC093 | Blood | HCC | 112 | 0 |
| HCC094 | Blood | HCC | 112 | 40.847 |
| HCC095 | Blood | HCC | 112 | 139.451 |
| HCC096 | Blood | HCC | 112 | 0 |
| HCC097 | Blood | HCC | 112 | 0 |
| HCC098 | Blood | HCC | 112 | 0 |
| HCC099 | Blood | HCC | 112 | 77.462 |
| HCC100 | Blood | HCC | 112 | 0 |
| HCC101 | Blood | HCC | 112 | 217.705 |
| HCC102 | Blood | HCC | 112 | 0 |
| HCC103 | Blood | HCC | 112 | 0 |
| HCC104 | Blood | HCC | 112 | 0 |
| HCC105 | Blood | HCC | 112 | 0 |
| HCC106 | Blood | HCC | 112 | 114.197 |
| HCC107 | Blood | HCC | 112 | 0 |
| HCC108 | Blood | HCC | 112 | 10.527 |
| HCC109 | Blood | HCC | 112 | 10.174 |
| HCC110 | Blood | HCC | 112 | 0 |
| HCC111 | Blood | HCC | 112 | 0 |
| HCC112 | Blood | HCC | 112 | 0 |
| Healthy001 | Blood | Healthy | 118 | 0 |
| Healthy002 | Blood | Healthy | 118 | 0 |
| Healthy003 | Blood | Healthy | 118 | 66.237 |
| Healthy004 | Blood | Healthy | 118 | 14.332 |
| Healthy005 | Blood | Healthy | 118 | 449.731 |
| Healthy006 | Blood | Healthy | 118 | 0 |
| Healthy007 | Blood | Healthy | 118 | 0 |
| Healthy008 | Blood | Healthy | 118 | 6.409 |
| Healthy009 | Blood | Healthy | 118 | 265.346 |
| Healthy010 | Blood | Healthy | 118 | 0 |
| Healthy011 | Blood | Healthy | 118 | 135.95 |
| Healthy012 | Blood | Healthy | 118 | 0 |
| Healthy013 | Blood | Healthy | 118 | 33.635 |
| Healthy014 | Blood | Healthy | 118 | 7.306 |
| Healthy015 | Blood | Healthy | 118 | 0 |
| Healthy016 | Blood | Healthy | 118 | 677.59 |
| Healthy017 | Blood | Healthy | 118 | 697.819 |
| Healthy018 | Blood | Healthy | 118 | 0 |
| Healthy019 | Blood | Healthy | 118 | 0 |
| Healthy020 | Blood | Healthy | 118 | 0 |
| Healthy021 | Blood | Healthy | 118 | 0 |
| Healthy022 | Blood | Healthy | 118 | 0 |
| Healthy023 | Blood | Healthy | 118 | 0 |
| Healthy024 | Blood | Healthy | 118 | 0 |
| Healthy025 | Blood | Healthy | 118 | 0 |
| Healthy026 | Blood | Healthy | 118 | 0 |
| Healthy027 | Blood | Healthy | 118 | 0 |
| Healthy028 | Blood | Healthy | 118 | 55.799 |
| Healthy029 | Blood | Healthy | 118 | 0 |
| Healthy030 | Blood | Healthy | 118 | 0 |
| Healthy031 | Blood | Healthy | 118 | 0 |
| Healthy032 | Blood | Healthy | 118 | 0 |
| Healthy033 | Blood | Healthy | 118 | 0 |
| Healthy034 | Blood | Healthy | 118 | 0 |
| Healthy035 | Blood | Healthy | 118 | 0 |
| Healthy036 | Blood | Healthy | 118 | 0 |
| Healthy037 | Blood | Healthy | 118 | 473.053 |
| Healthy038 | Blood | Healthy | 118 | 0 |
| Healthy039 | Blood | Healthy | 118 | 0 |
| Healthy040 | Blood | Healthy | 118 | 0 |
| Healthy041 | Blood | Healthy | 118 | 0 |
| Healthy042 | Blood | Healthy | 118 | 632.208 |
| Healthy043 | Blood | Healthy | 118 | 13.34 |
| Healthy044 | Blood | Healthy | 118 | 0 |
| Healthy045 | Blood | Healthy | 118 | 0 |
| Healthy046 | Blood | Healthy | 118 | 0 |
| Healthy047 | Blood | Healthy | 118 | 0 |
| Healthy048 | Blood | Healthy | 118 | 11.758 |
| Healthy049 | Blood | Healthy | 118 | 0 |
| Healthy050 | Blood | Healthy | 118 | 0 |
| Healthy051 | Blood | Healthy | 118 | 0 |
| Healthy052 | Blood | Healthy | 118 | 0 |
| Healthy053 | Blood | Healthy | 118 | 79.216 |
| Healthy054 | Blood | Healthy | 118 | 0 |
| Healthy055 | Blood | Healthy | 118 | 0 |
| Healthy056 | Blood | Healthy | 118 | 0 |
| Healthy057 | Blood | Healthy | 118 | 0 |
| Healthy058 | Blood | Healthy | 118 | 0 |
| Healthy059 | Blood | Healthy | 118 | 587.685 |
| Healthy060 | Blood | Healthy | 118 | 0 |
| Healthy061 | Blood | Healthy | 118 | 0 |
| Healthy062 | Blood | Healthy | 118 | 0 |
| Healthy063 | Blood | Healthy | 118 | 0 |
| Healthy064 | Blood | Healthy | 118 | 0 |
| Healthy065 | Blood | Healthy | 118 | 0 |
| Healthy066 | Blood | Healthy | 118 | 0 |
| Healthy067 | Blood | Healthy | 118 | 0 |
| Healthy068 | Blood | Healthy | 118 | 0 |
| Healthy069 | Blood | Healthy | 118 | 0 |
| Healthy070 | Blood | Healthy | 118 | 0 |
| Healthy071 | Blood | Healthy | 118 | 0 |
| Healthy072 | Blood | Healthy | 118 | 0 |
| Healthy073 | Blood | Healthy | 118 | 539.82 |
| Healthy074 | Blood | Healthy | 118 | 0 |
| Healthy075 | Blood | Healthy | 118 | 0 |
| Healthy076 | Blood | Healthy | 118 | 0 |
| Healthy077 | Blood | Healthy | 118 | 0 |
| Healthy078 | Blood | Healthy | 118 | 0 |
| Healthy079 | Blood | Healthy | 118 | 0 |
| Healthy080 | Blood | Healthy | 118 | 0 |
| Healthy081 | Blood | Healthy | 118 | 2092.506 |
| Healthy082 | Blood | Healthy | 118 | 0 |
| Healthy083 | Blood | Healthy | 118 | 0 |
| Healthy084 | Blood | Healthy | 118 | 0 |
| Healthy085 | Blood | Healthy | 118 | 0 |
| Healthy086 | Blood | Healthy | 118 | 0 |
| Healthy087 | Blood | Healthy | 118 | 0 |
| Healthy088 | Blood | Healthy | 118 | 0 |
| Healthy089 | Blood | Healthy | 118 | 0 |
| Healthy090 | Blood | Healthy | 118 | 639.659 |
| Healthy091 | Blood | Healthy | 118 | 0 |
| Healthy092 | Blood | Healthy | 118 | 0 |
| Healthy093 | Blood | Healthy | 118 | 0 |
| Healthy094 | Blood | Healthy | 118 | 0 |
| Healthy095 | Blood | Healthy | 118 | 0 |
| Healthy096 | Blood | Healthy | 118 | 7.554 |
| Healthy097 | Blood | Healthy | 118 | 0 |
| Healthy098 | Blood | Healthy | 118 | 0 |
| Healthy099 | Blood | Healthy | 118 | 0 |
| Healthy100 | Blood | Healthy | 118 | 0 |
| Healthy101 | Blood | Healthy | 118 | 0 |
| Healthy102 | Blood | Healthy | 118 | 0 |
| Healthy103 | Blood | Healthy | 118 | 0 |
| Healthy104 | Blood | Healthy | 118 | 0 |
| Healthy105 | Blood | Healthy | 118 | 0 |
| Healthy106 | Blood | Healthy | 118 | 0 |
| Healthy107 | Blood | Healthy | 118 | 0 |
| Healthy108 | Blood | Healthy | 118 | 0 |
| Healthy109 | Blood | Healthy | 118 | 0 |
| Healthy110 | Blood | Healthy | 118 | 0 |
| Healthy111 | Blood | Healthy | 118 | 0 |
| Healthy112 | Blood | Healthy | 118 | 0 |
| Healthy113 | Blood | Healthy | 118 | 0 |
| Healthy114 | Blood | Healthy | 118 | 46.521 |
| Healthy115 | Blood | Healthy | 118 | 148.367 |
| Healthy116 | Blood | Healthy | 118 | 0 |
| Healthy117 | Blood | Healthy | 118 | 0 |
| Healthy118 | Blood | Healthy | 118 | 651.536 |
| KIRC01 | Blood | KIRC | 15 | 12.144 |
| KIRC02 | Blood | KIRC | 15 | 320.544 |
| KIRC03 | Blood | KIRC | 15 | 0 |
| KIRC04 | Blood | KIRC | 15 | 0 |
| KIRC05 | Blood | KIRC | 15 | 0 |
| KIRC06 | Blood | KIRC | 15 | 0 |
| KIRC07 | Blood | KIRC | 15 | 0 |
| KIRC08 | Blood | KIRC | 15 | 1365.577 |
| KIRC09 | Blood | KIRC | 15 | 0 |
| KIRC10 | Blood | KIRC | 15 | 0 |
| KIRC11 | Blood | KIRC | 15 | 4.99 |
| KIRC12 | Blood | KIRC | 15 | 0 |
| KIRC13 | Blood | KIRC | 15 | 0 |
| KIRC14 | Blood | KIRC | 15 | 0 |
| KIRC15 | Blood | KIRC | 15 | 80.766 |
| MEL01 | Blood | MEL | 21 | 0 |
| MEL02 | Blood | MEL | 21 | 0 |
| MEL03 | Blood | MEL | 21 | 0 |
| MEL04 | Blood | MEL | 21 | 0 |
| MEL05 | Blood | MEL | 21 | 0 |
| MEL06 | Blood | MEL | 21 | 0 |
| MEL07 | Blood | MEL | 21 | 0 |
| MEL08 | Blood | MEL | 21 | 0 |
| MEL09 | Blood | MEL | 21 | 0 |
| MEL10 | Blood | MEL | 21 | 0 |
| MEL11 | Blood | MEL | 21 | 0 |
| MEL12 | Blood | MEL | 21 | 0 |
| MEL13 | Blood | MEL | 21 | 0 |
| MEL14 | Blood | MEL | 21 | 0 |
| MEL15 | Blood | MEL | 21 | 0 |
| MEL16 | Blood | MEL | 21 | 0 |
| MEL17 | Blood | MEL | 21 | 0 |
| MEL18 | Blood | MEL | 21 | 0 |
| MEL19 | Blood | MEL | 21 | 0 |
| MEL20 | Blood | MEL | 21 | 0 |
| MEL21 | Blood | MEL | 21 | 0 |
| ML01 | Blood | ML | 28 | 0 |
| ML02 | Blood | ML | 28 | 1372.477 |
| ML03 | Blood | ML | 28 | 0 |
| ML04 | Blood | ML | 28 | 0 |
| ML05 | Blood | ML | 28 | 0 |
| ML06 | Blood | ML | 28 | 0 |
| ML07 | Blood | ML | 28 | 0 |
| ML08 | Blood | ML | 28 | 0 |
| ML09 | Blood | ML | 28 | 0 |
| ML10 | Blood | ML | 28 | 0 |
| ML11 | Blood | ML | 28 | 0 |
| ML12 | Blood | ML | 28 | 74.933 |
| ML13 | Blood | ML | 28 | 0 |
| ML14 | Blood | ML | 28 | 0 |
| ML15 | Blood | ML | 28 | 0 |
| ML16 | Blood | ML | 28 | 0 |
| ML17 | Blood | ML | 28 | 0 |
| ML18 | Blood | ML | 28 | 0 |
| ML19 | Blood | ML | 28 | 0 |
| ML20 | Blood | ML | 28 | 0 |
| ML21 | Blood | ML | 28 | 0 |
| ML22 | Blood | ML | 28 | 0 |
| ML23 | Blood | ML | 28 | 0 |
| ML24 | Blood | ML | 28 | 73.939 |
| ML25 | Blood | ML | 28 | 0 |
| ML26 | Blood | ML | 28 | 0 |
| ML27 | Blood | ML | 28 | 137.782 |
| ML28 | Blood | ML | 28 | 14.256 |
| OV01 | Blood | OV | 30 | 55.625 |
| OV02 | Blood | OV | 30 | 0 |
| OV03 | Blood | OV | 30 | 230.684 |
| OV04 | Blood | OV | 30 | 0 |
| OV05 | Blood | OV | 30 | 57.033 |
| OV06 | Blood | OV | 30 | 135.701 |
| OV07 | Blood | OV | 30 | 0 |
| OV08 | Blood | OV | 30 | 0 |
| OV09 | Blood | OV | 30 | 0 |
| OV10 | Blood | OV | 30 | 0 |
| OV11 | Blood | OV | 30 | 157.458 |
| OV12 | Blood | OV | 30 | 0 |
| OV13 | Blood | OV | 30 | 0 |
| OV14 | Blood | OV | 30 | 0 |
| OV15 | Blood | OV | 30 | 0 |
| OV16 | Blood | OV | 30 | 0 |
| OV17 | Blood | OV | 30 | 0 |
| OV18 | Blood | OV | 30 | 11.913 |
| OV19 | Blood | OV | 30 | 0 |
| OV20 | Blood | OV | 30 | 0 |
| OV21 | Blood | OV | 30 | 725.845 |
| OV22 | Blood | OV | 30 | 0 |
| OV23 | Blood | OV | 30 | 214.382 |
| OV24 | Blood | OV | 30 | 0 |
| OV25 | Blood | OV | 30 | 0 |
| OV26 | Blood | OV | 30 | 0 |
| OV27 | Blood | OV | 30 | 0 |
| OV28 | Blood | OV | 30 | 334.988 |
| OV29 | Blood | OV | 30 | 30.329 |
| OV30 | Blood | OV | 30 | 0 |
| PAAD001 | Blood | PAAD | 164 | 0 |
| PAAD002 | Blood | PAAD | 164 | 0 |
| PAAD003 | Blood | PAAD | 164 | 0 |
| PAAD004 | Blood | PAAD | 164 | 0 |
| PAAD005 | Blood | PAAD | 164 | 0 |
| PAAD006 | Blood | PAAD | 164 | 0 |
| PAAD007 | Blood | PAAD | 164 | 500.1 |
| PAAD008 | Blood | PAAD | 164 | 0 |
| PAAD009 | Blood | PAAD | 164 | 0 |
| PAAD010 | Blood | PAAD | 164 | 0 |
| PAAD011 | Blood | PAAD | 164 | 0 |
| PAAD012 | Blood | PAAD | 164 | 0 |
| PAAD013 | Blood | PAAD | 164 | 0 |
| PAAD014 | Blood | PAAD | 164 | 31.894 |
| PAAD015 | Blood | PAAD | 164 | 0 |
| PAAD016 | Blood | PAAD | 164 | 212.168 |
| PAAD017 | Blood | PAAD | 164 | 0 |
| PAAD018 | Blood | PAAD | 164 | 0 |
| PAAD019 | Blood | PAAD | 164 | 0 |
| PAAD020 | Blood | PAAD | 164 | 33.693 |
| PAAD021 | Blood | PAAD | 164 | 0 |
| PAAD022 | Blood | PAAD | 164 | 549.652 |
| PAAD023 | Blood | PAAD | 164 | 673.994 |
| PAAD024 | Blood | PAAD | 164 | 0 |
| PAAD025 | Blood | PAAD | 164 | 0 |
| PAAD026 | Blood | PAAD | 164 | 0 |
| PAAD027 | Blood | PAAD | 164 | 0 |
| PAAD028 | Blood | PAAD | 164 | 49.171 |
| PAAD029 | Blood | PAAD | 164 | 0 |
| PAAD030 | Blood | PAAD | 164 | 0 |
| PAAD031 | Blood | PAAD | 164 | 27.83 |
| PAAD032 | Blood | PAAD | 164 | 0 |
| PAAD033 | Blood | PAAD | 164 | 0 |
| PAAD034 | Blood | PAAD | 164 | 0 |
| PAAD035 | Blood | PAAD | 164 | 0 |
| PAAD036 | Blood | PAAD | 164 | 0 |
| PAAD037 | Blood | PAAD | 164 | 0 |
| PAAD038 | Blood | PAAD | 164 | 0 |
| PAAD039 | Blood | PAAD | 164 | 0 |
| PAAD040 | Blood | PAAD | 164 | 0 |
| PAAD041 | Blood | PAAD | 164 | 0 |
| PAAD042 | Blood | PAAD | 164 | 0 |
| PAAD043 | Blood | PAAD | 164 | 0 |
| PAAD044 | Blood | PAAD | 164 | 0 |
| PAAD045 | Blood | PAAD | 164 | 0 |
| PAAD046 | Blood | PAAD | 164 | 0 |
| PAAD047 | Blood | PAAD | 164 | 0 |
| PAAD048 | Blood | PAAD | 164 | 0 |
| PAAD049 | Blood | PAAD | 164 | 0 |
| PAAD050 | Blood | PAAD | 164 | 0 |
| PAAD051 | Blood | PAAD | 164 | 0 |
| PAAD052 | Blood | PAAD | 164 | 0 |
| PAAD053 | Blood | PAAD | 164 | 0 |
| PAAD054 | Blood | PAAD | 164 | 337.218 |
| PAAD055 | Blood | PAAD | 164 | 0 |
| PAAD056 | Blood | PAAD | 164 | 0 |
| PAAD057 | Blood | PAAD | 164 | 78.633 |
| PAAD058 | Blood | PAAD | 164 | 0 |
| PAAD059 | Blood | PAAD | 164 | 395.691 |
| PAAD060 | Blood | PAAD | 164 | 280.747 |
| PAAD061 | Blood | PAAD | 164 | 0 |
| PAAD062 | Blood | PAAD | 164 | 364.426 |
| PAAD063 | Blood | PAAD | 164 | 0 |
| PAAD064 | Blood | PAAD | 164 | 440.941 |
| PAAD065 | Blood | PAAD | 164 | 0 |
| PAAD066 | Blood | PAAD | 164 | 0 |
| PAAD067 | Blood | PAAD | 164 | 167.953 |
| PAAD068 | Blood | PAAD | 164 | 0 |
| PAAD069 | Blood | PAAD | 164 | 0 |
| PAAD070 | Blood | PAAD | 164 | 0 |
| PAAD071 | Blood | PAAD | 164 | 62.323 |
| PAAD072 | Blood | PAAD | 164 | 0 |
| PAAD073 | Blood | PAAD | 164 | 0 |
| PAAD074 | Blood | PAAD | 164 | 0 |
| PAAD075 | Blood | PAAD | 164 | 0 |
| PAAD076 | Blood | PAAD | 164 | 0 |
| PAAD077 | Blood | PAAD | 164 | 0 |
| PAAD078 | Blood | PAAD | 164 | 0 |
| PAAD079 | Blood | PAAD | 164 | 0 |
| PAAD080 | Blood | PAAD | 164 | 0 |
| PAAD081 | Blood | PAAD | 164 | 0 |
| PAAD082 | Blood | PAAD | 164 | 0 |
| PAAD083 | Blood | PAAD | 164 | 0 |
| PAAD084 | Blood | PAAD | 164 | 0 |
| PAAD085 | Blood | PAAD | 164 | 43.209 |
| PAAD086 | Blood | PAAD | 164 | 0 |
| PAAD087 | Blood | PAAD | 164 | 0 |
| PAAD088 | Blood | PAAD | 164 | 0 |
| PAAD089 | Blood | PAAD | 164 | 0 |
| PAAD090 | Blood | PAAD | 164 | 0 |
| PAAD091 | Blood | PAAD | 164 | 0 |
| PAAD092 | Blood | PAAD | 164 | 0 |
| PAAD093 | Blood | PAAD | 164 | 0 |
| PAAD094 | Blood | PAAD | 164 | 0 |
| PAAD095 | Blood | PAAD | 164 | 0 |
| PAAD096 | Blood | PAAD | 164 | 0 |
| PAAD097 | Blood | PAAD | 164 | 19.097 |
| PAAD098 | Blood | PAAD | 164 | 0 |
| PAAD099 | Blood | PAAD | 164 | 0 |
| PAAD100 | Blood | PAAD | 164 | 0 |
| PAAD101 | Blood | PAAD | 164 | 0 |
| PAAD102 | Blood | PAAD | 164 | 0 |
| PAAD103 | Blood | PAAD | 164 | 0 |
| PAAD104 | Blood | PAAD | 164 | 0 |
| PAAD105 | Blood | PAAD | 164 | 0 |
| PAAD106 | Blood | PAAD | 164 | 0 |
| PAAD107 | Blood | PAAD | 164 | 0 |
| PAAD108 | Blood | PAAD | 164 | 6.929 |
| PAAD109 | Blood | PAAD | 164 | 0 |
| PAAD110 | Blood | PAAD | 164 | 0 |
| PAAD111 | Blood | PAAD | 164 | 6.783 |
| PAAD112 | Blood | PAAD | 164 | 0 |
| PAAD113 | Blood | PAAD | 164 | 0 |
| PAAD114 | Blood | PAAD | 164 | 0 |
| PAAD115 | Blood | PAAD | 164 | 0 |
| PAAD116 | Blood | PAAD | 164 | 0 |
| PAAD117 | Blood | PAAD | 164 | 0 |
| PAAD118 | Blood | PAAD | 164 | 0 |
| PAAD119 | Blood | PAAD | 164 | 316.879 |
| PAAD120 | Blood | PAAD | 164 | 0 |
| PAAD121 | Blood | PAAD | 164 | 0 |
| PAAD122 | Blood | PAAD | 164 | 0 |
| PAAD123 | Blood | PAAD | 164 | 0 |
| PAAD124 | Blood | PAAD | 164 | 0 |
| PAAD125 | Blood | PAAD | 164 | 0 |
| PAAD126 | Blood | PAAD | 164 | 0 |
| PAAD127 | Blood | PAAD | 164 | 0 |
| PAAD128 | Blood | PAAD | 164 | 0 |
| PAAD129 | Blood | PAAD | 164 | 0 |
| PAAD130 | Blood | PAAD | 164 | 0 |
| PAAD131 | Blood | PAAD | 164 | 0 |
| PAAD132 | Blood | PAAD | 164 | 23.46 |
| PAAD133 | Blood | PAAD | 164 | 0 |
| PAAD134 | Blood | PAAD | 164 | 7.909 |
| PAAD135 | Blood | PAAD | 164 | 0 |
| PAAD136 | Blood | PAAD | 164 | 0 |
| PAAD137 | Blood | PAAD | 164 | 0 |
| PAAD138 | Blood | PAAD | 164 | 0 |
| PAAD139 | Blood | PAAD | 164 | 6.898 |
| PAAD140 | Blood | PAAD | 164 | 0 |
| PAAD141 | Blood | PAAD | 164 | 0 |
| PAAD142 | Blood | PAAD | 164 | 0 |
| PAAD143 | Blood | PAAD | 164 | 0 |
| PAAD144 | Blood | PAAD | 164 | 0 |
| PAAD145 | Blood | PAAD | 164 | 0 |
| PAAD146 | Blood | PAAD | 164 | 67.881 |
| PAAD147 | Blood | PAAD | 164 | 0 |
| PAAD148 | Blood | PAAD | 164 | 16.863 |
| PAAD149 | Blood | PAAD | 164 | 0 |
| PAAD150 | Blood | PAAD | 164 | 0 |
| PAAD151 | Blood | PAAD | 164 | 0 |
| PAAD152 | Blood | PAAD | 164 | 0 |
| PAAD153 | Blood | PAAD | 164 | 0 |
| PAAD154 | Blood | PAAD | 164 | 7.707 |
| PAAD155 | Blood | PAAD | 164 | 163.23 |
| PAAD156 | Blood | PAAD | 164 | 0 |
| PAAD157 | Blood | PAAD | 164 | 0 |
| PAAD158 | Blood | PAAD | 164 | 0 |
| PAAD159 | Blood | PAAD | 164 | 553.96 |
| PAAD160 | Blood | PAAD | 164 | 0 |
| PAAD161 | Blood | PAAD | 164 | 0 |
| PAAD162 | Blood | PAAD | 164 | 0 |
| PAAD163 | Blood | PAAD | 164 | 0 |
| PAAD164 | Blood | PAAD | 164 | 29.303 |
| SCLC01 | Blood | SCLC | 36 | 0 |
| SCLC02 | Blood | SCLC | 36 | 0 |
| SCLC03 | Blood | SCLC | 36 | 0 |
| SCLC04 | Blood | SCLC | 36 | 0 |
| SCLC05 | Blood | SCLC | 36 | 0 |
| SCLC06 | Blood | SCLC | 36 | 0 |
| SCLC07 | Blood | SCLC | 36 | 0 |
| SCLC08 | Blood | SCLC | 36 | 0 |
| SCLC09 | Blood | SCLC | 36 | 8.972 |
| SCLC10 | Blood | SCLC | 36 | 0 |
| SCLC11 | Blood | SCLC | 36 | 69.623 |
| SCLC12 | Blood | SCLC | 36 | 0 |
| SCLC13 | Blood | SCLC | 36 | 337.107 |
| SCLC14 | Blood | SCLC | 36 | 66.4 |
| SCLC15 | Blood | SCLC | 36 | 0 |
| SCLC16 | Blood | SCLC | 36 | 644.392 |
| SCLC17 | Blood | SCLC | 36 | 100.139 |
| SCLC18 | Blood | SCLC | 36 | 44.476 |
| SCLC19 | Blood | SCLC | 36 | 0 |
| SCLC20 | Blood | SCLC | 36 | 252.601 |
| SCLC21 | Blood | SCLC | 36 | 0 |
| SCLC22 | Blood | SCLC | 36 | 0 |
| SCLC23 | Blood | SCLC | 36 | 154.609 |
| SCLC24 | Blood | SCLC | 36 | 596.141 |
| SCLC25 | Blood | SCLC | 36 | 0 |
| SCLC26 | Blood | SCLC | 36 | 50.029 |
| SCLC27 | Blood | SCLC | 36 | 0 |
| SCLC28 | Blood | SCLC | 36 | 173.639 |
| SCLC29 | Blood | SCLC | 36 | 0 |
| SCLC30 | Blood | SCLC | 36 | 308.604 |
| SCLC31 | Blood | SCLC | 36 | 0 |
| SCLC32 | Blood | SCLC | 36 | 0 |
| SCLC33 | Blood | SCLC | 36 | 0 |
| SCLC34 | Blood | SCLC | 36 | 0 |
| SCLC35 | Blood | SCLC | 36 | 0 |
| SCLC36 | Blood | SCLC | 36 | 225.233 |
| Urine01 | Urine | Urine | 16 | 0 |
| Urine02 | Urine | Urine | 16 | 25.145 |
| Urine03 | Urine | Urine | 16 | 0 |
| Urine04 | Urine | Urine | 16 | 77.676 |
| Urine05 | Urine | Urine | 16 | 0 |
| Urine06 | Urine | Urine | 16 | 39.442 |
| Urine07 | Urine | Urine | 16 | 36.71 |
| Urine08 | Urine | Urine | 16 | 41.503 |
| Urine09 | Urine | Urine | 16 | 17.931 |
| Urine10 | Urine | Urine | 16 | 0 |
| Urine11 | Urine | Urine | 16 | 0 |
| Urine12 | Urine | Urine | 16 | 0 |
| Urine13 | Urine | Urine | 16 | 0 |
| Urine14 | Urine | Urine | 16 | 0 |
| Urine15 | Urine | Urine | 16 | 62.606 |
| Urine16 | Urine | Urine | 16 | 0 |
