# Supplementary material for: CircVAPA promotes small cell lung cancer progression by modulating the miR-377-3p and miR-494-3p/IGF1R/AKT axis
Source: Mol Cancer. 2022 Jun 6;21:123. doi: 10.1186/s12943-022-01595-9 (PMC9172052; doi:10.1186/s12943-022-01595-9)
Supplement: Supplementary file 5 — Additional file 5: Table S4. Common candidate targets of miR-377-3p and miR-494-3p. [file 12943_2022_1595_MOESM5_ESM.docx]

**Table S4. Common candidate targets of miR-377-3p and miR-494-3p.**

|  |
| --- |
| AAK1 |
| ADARB1 |
| ANGEL2 |
| ANKRD13A |
| ATXN1 |
| CD59 |
| CMTM3 |
| DCP2 |
| DENND5B |
| DPY19L1 |
| FOXM1 |
| GNAS |
| GPR63 |
| IGF1R |
| KDM2B |
| LARP4 |
| LONRF2 |
| MTR |
| NAV2 |
| NCEH1 |
| NREP |
| NRXN1 |
| NSD1 |
| NUP210 |
| PCMTD2 |
| PDPR |
| PHLPP2 |
| PPARGC1B |
| PRRC2B |
| PURB |
| QKI |
| SLC38A2 |
| SOD2 |
| ULK2 |
| VPS13C |
| XIAP |
| XPO1 |
| ZC3H12C |
| ZFHX3 |
| ZFX |
| ZNF652 |
