# Supplementary material for: CircVAPA promotes small cell lung cancer progression by modulating the miR-377-3p and miR-494-3p/IGF1R/AKT axis
Source: Mol Cancer. 2022 Jun 6;21:123. doi: 10.1186/s12943-022-01595-9 (PMC9172052; doi:10.1186/s12943-022-01595-9)
Supplement: Supplementary file 6 — Additonal file 6: Table S5. Oligos used in the study. [file 12943_2022_1595_MOESM6_ESM.docx]

**Table S5. Oligos used in the study.**

| circVAPA-F | GGAAGCTGTGTGGAAAGAGG | For circVAPA qPCR detection |
| --- | --- | --- |
| circVAPA-R | GGCGAGGTGCTGTAGTCTTC |  |
| VAPA-F | CCTATGCCAAAACCACAC | For VAPA mRNA qPCR detection |
| VAPA-R | TGCCGATTTTCTTCTGATA |  |
| 18S rRNA-F | CGGCGACGACCCATTCGAAC | For 18S qPCR detection |
| 18S rRNA-R | GAATCGAACCCTGATTCCC |  |
| U6-F | CGCTTCGGCAGCACATATAC | For U6 qPCR detection |
| U6-R | TTCACGAATTTGCGTGTCAT |  |
| GAPDH-F | CTTCATTGACCTCAACTACATG | For GAPDH qPCR detection |
| GAPDH-R | CTCGCTCCTGGAAGATGGTGA |  |
| circHIPK3-F | ATGTTGGTGGATCCTGTTCG | For circHIPK3 qPCR detection |
| circHIPK3-R | GGGTAGACCAAGACTTGTGA |  |
| EIciPAIP2-F | AGCTCGAGATCTCCCACAAA | For EIciPAIP2 qPCR detection |
| EIciPAIP2-R | ACTGCTGCGACTTGGATCTT |  |
| si-SCR | UUCUCCGAACGUGUCACGU | Negative control of siRNAs |
| si-circVAPA#1 | UAAAUUGGCCCCUUCACAG | siRNAs of circVAPA |
| si-circVAPA#2 | AUGAUAAAUUGGCCCCUUC |  |
| si-IGF1R | CCUUUCUAGGGACAUGAAAUU | siRNAs of IGF1R |
| circVAPA shRNA-1F | CCGGATGATAAATTGGCCCCTTCCTCGA  GGAAGGGGCCAATTTATCATTTTTTG | shRNAs for knock down  circVAPA |
| circVAPA shRNA-1R | AATTCAAAAAATGATAAATTGGCCCCTTC CTCGAGGAAGGGGCCAATTTATCAT |  |
| OE circVAPA Fragment1-F1 | GCTTGGTACCGAGCTCGGATCCACCCCTCCATCCCTGAACTC | For circVAPA  overexpression |
| OE circVAPA Fragment1-R1 | TTGTAGAGACAGGGTCTTGT |  |
| OE circVAPA Fragmen2-F2 | ACAAGACCCTGTCTCTACAACCAACTCATTTTACCTTCCA |  |
| OE circVAPA Fragment2-R2 | GACTACATCTGTGAAGGGGCCTGTAAAGTTTAAAAATTAA |  |
| OE circVAPA Fragment3-F3 | GCCCCTTCACAGATGTAGTCAC |  |
| OE circVAPA Fragment3-R3 | CAATTTATCATTTTCATTGG |  |
| OE circVAPA Fragment4-F4 | CCAATGAAAATGATAAATTGGTAAGTAGGAGAATGTAAAAA |  |
| OE circVAPA Fragment4-R4 | GTGATGGATATCTGCAGAATTCCCTTACCTACCTCTCAGTTG |  |
| Scramble | Biotin-TTCTCCGAACGTGTCACGT | Control oligo |
| circVAPA-Probe | Biotin-CATCTGTGAAGGGGCCAATTTATCATTTTC | Antisense oligo of circVAPA |
| miR-377-3p RT-primer | GTCGTATCCAGTGCAGGGTCCGAGGTATTCGCAC  TGGATACGACACAAAA | For miR-377-3p reverse transcription |
| miR-142-3p RT-primer | GTCGTATCCAGTGCAGGGTCCGAGGTATTCGCAC  TGGATACGACTCCATA | For miR-142-3p reverse transcription |
| miR-494-3p RT-primer | GTCGTATCCAGTGCAGGGTCCGAGGTATTCGCAC  TGGATACGACGAGGTT | For miR-494-3p reverse transcription |
| miR-1298-5p RT-primer | GTCGTATCCAGTGCAGGGTCCGAGGTATTCGCAC  TGGATACGACTACATC | For miR-1298-5p reverse transcription |
| miR-1179 RT-primer | GTCGTATCCAGTGCAGGGTCCGAGGTATTCGCAC  TGGATACGACCCAACC | For miR-1179 reverse transcription |
| miR-1208 RT-primer | GTCGTATCCAGTGCAGGGTCCGAGGTATTCGCAC  TGGATACGACTCCGCC | For miR-1208 reverse transcription |
| miR-1231 RT-primer | GTCGTATCCAGTGCAGGGTCCGAGGTATTCGCAC  TGGATACGACGCAGCT | For miR-1231 reverse transcription |
| miR-1246 RT-primer | GTCGTATCCAGTGCAGGGTCCGAGGTATTCGCAC  TGGATACGACCCTGCT | For miR-1246 reverse transcription |
| miR-1290 RT-primer | GTCGTATCCAGTGCAGGGTCCGAGGTATTCGCAC  TGGATACGACTCCCTG | For miR-1290 reverse transcription |
| miR-548p RT-primer | GTCGTATCCAGTGCAGGGTCCGAGGTATTCGCAC  TGGATACGACAAAGTA | For miR-548p reverse transcription |
| miR-581 RT-primer | GTCGTATCCAGTGCAGGGTCCGAGGTATTCGCAC  TGGATACGACACTGAT | For miR-581 reverse transcription |
| miR-587 RT-primer | GTCGTATCCAGTGCAGGGTCCGAGGTATTCGCAC  TGGATACGACGTGACT | For miR-587 reverse transcription |
| miR-607 RT-primer | GTCGTATCCAGTGCAGGGTCCGAGGTATTCGCAC  TGGATACGACGTTATA | For miR-607 reverse transcription |
| miR-885-5p RT-primer | GTCGTATCCAGTGCAGGGTCCGAGGTATTCGCAC  TGGATACGACAGAGGC | For miR-885-5p reverse transcription |
| microRNAs reverse primer | ATCCAGTGCAGGGTCCGAGG | For microRNAs  qPCR detection |
| miR-377-3p forward primer | GGGCCATCACACAAAGGCAACTT | For miR-377-3p  qPCR detection |
| miR-142-3p forward primer | GCGGCTGTAGTGTTTCCTACTTTATG | For miR-142-3p  qPCR detection |
| miR-494-3p forward primer | GCCTGAAACATACACGGGAAACCT | For miR-494-3p  qPCR detection |
| miR-1298-5p forward primer | CCGCTTCATTCGGCTGTCCA | For miR-1298-5p  qPCR detection |
| miR-1179 forward primer | GGAGCATTCTTTCATTGGTTGG | For miR-1179  qPCR detection |
| miR-1208 forward primer | TCACTGTTCAGACAGGCGGA | For miR-1208  qPCR detection |
| miR-1231 forward primer | GTGTCTGGGCGGACAGCT | For miR-1231  qPCR detection |
| miR-1246 forward primer | GGCAATGGATTTTTGGAGCAGG | For miR-1246  qPCR detection |
| miR-1290 forward primer | CGTGGATTTTTGGATCAGGGA | For miR-1290  qPCR detection |
| miR-548p forward primer | GCCTAGCAAAAACTGCAGTTACTT | For miR-548p  qPCR detection |
| miR-581 forward primer | CGCTCTTGTGTTCTCTAGATCAG | For miR-581  qPCR detection |
| miR-587 forward primer | CGGTTTCCATAGGTGATGAGTC | For miR-587  qPCR detection |
| miR-607 forward primer | CGCGGTTCAAATCCAGATCTATA | For miR-607  qPCR detection |
| miR-885-5p forward primer | TCCATTACACTACCCTGCCTCT | For miR-885-5p  qPCR detection |
| β-actin-F | TGGCACCCAGCACAATGAA | For β-actin  qPCR detection |
| β-actin-R | CTAAGTCATAGTCCGCCTAGAAGCA |  |
| IGF1R-F | AATTGCCACAAGTCCAGCTG | For IGF1R qPCR detection |
| IGF1R-R | CAGCCTTGGATGAACGATGG |  |
| FOXM1-F | GCTCTGTGGTTTATTGGCGTC | For FOXM1  qPCR detection |
| FOXM1-R | TGCAGGACTAGCACTGGGAA |  |
| GNAS-F | TGCTCTGAGTTGTTTCGCCT | For GNAS  qPCR detection |
| GNAS-R | TAGTGCCGCTCTTTCTTGGC |  |
| GPR63-F | GGCCCCATTCACCACTTACA | For GPR63  qPCR detection |
| GPR63-R | GAGCTGCGGCAAAAACTTGA |  |
| KDM2B-F | GGGTTCCCCTGATATTTCGAGA | For KDM2B  qPCR detection |
| KDM2B-R | GCTCCCCACTAGGAGTTTGAC |  |
| LONRF2-F | GGAGCTGGCTCCTGATGATA | For LONRF2  qPCR detection |
| LONRF2-R | CCCAATCCAGAAAGAGCCTGA |  |
| NRXN1-F | AACACATCGCGGTGTACCTT | For NRXN1  qPCR detection |
| NRXN1-R | TCCATAAGATTGCTTTGGGAATGT |  |
| NUP210-F | GGGCGCACGATGTTCAGAA | For NUP210  qPCR detection |
| NUP210-R | CACCACCAGGTCGAAATGGG |  |
| PRRC2B-F | ATGTCTGGAGCAAGGTGACG | For PRRC2B  qPCR detection |
| PRRC2B-R | TCTTAAAACCCTGCTCCGCA |  |
| XPO1-F | CATGGGCTGAAAACTCAACCG | For XPO1  qPCR detection |
| XPO1-R | TTGCTGATGCTGTAGCTCCC |  |
| circVAPA-Luci-WT-F | AGATCGCCGTGTAATTCTAGACAGACCTCAAATTCAAAGGC | For Luci-circVAPA-WT construction |
| circVAPA-Luci-WT-R | GCCGGCCGCCCCGACTCTAGAATTCCCTGGTGGAGTTATAC |  |
| circVAPA-Luci-Mut-377-F | CAGCACCTCGCCGGTACTGACAGAGGCCCAACAGTGGAAT | For Luci-circVAPA-Mut-377 construction |
| circVAPA-Luci-Mut-377-R | CAGTACCGGCGAGGTGCTGTAG |  |
| circVAPA-Luci-Mut1(DM)-494-F | CATCGGATAGAAAAGTGTGAAACAAAGTGAAGACTACAGCAC | For Luci-circVAPA-Mut1-494 construction |
| circVAPA-Luci-Mut1(DM)-494-R | CACACTTTTCTATCCGATG |  |
| circVAPA-Luci-Mut2(DM)-494-F | CCAGGGTCAACTGTGACTGAAACAGTAATGCTACAGCCCTTTG | For Luci-circVAPA-Mut2-494 construction |
| circVAPA-Luci-Mut2(DM)-494-R | CAGTCACAGTTGACCCTGG |  |
| IGF1R-Luci-WT-F | AGATCGCCGTGTAATTCTAGAGTTCAGTGTTTCCACTCACC | For Luci-IGF1R-WT construction |
| IGF1R-Luci-WT-R | GCCGGCCGCCCCGACTCTAGAATGGCACTGTGCTGACAATG |  |
| IGF1R-Luci-Mut-377-F | GATGGTGCAGTCACTTTACTGG | For Luci-IGF1R-Mut-377 construction |
| IGF1R-Luci-Mut-377-R | CCAGTAAAGTGACTGCACCATCTGTCAAGCCAAGTTTTCTCTTTT |  |
| IGF1R-Luci-Mut1(DM)-494-F | CATTTTTAGCACTTCTCACC | For Luci-IGF1R-Mut1-494 construction |
| IGF1R-Luci-Mut1(DM)-494-R | GTGAGAAGTGCTAAAAATGTTTCAAGACAAGACCCCTGGG |  |
| IGF1R-Luci-Mut2(DM)-494-F | CATTGCATTTTTGTAAGAACAG | For Luci-IGF1R-Mut2-494 construction |
| IGF1R-Luci-Mut2(DM)-494-R | CTGTTCTTACAAAAATGCAATGTTTCATTTAATTAGTTCTTGTAAACC |  |
